# Trans-ancestry genome-wide study of depression identifies 697 associations implicating cell types and pharmacotherapies

**DOI:** 10.1016/j.cell.2024.12.002

**Published:** 2025-01-14

**Authors:** Major Depressive Disorder Working Group of the Psychiatric Genomics Consortium

## Abstract

In a genome-wide association study (GWAS) meta-analysis of 688,808 individuals with major depression (MD) and 4,364,225 controls from 29 countries across diverse and admixed ancestries, we identify 697 associations at 635 loci, 293 of which are novel. Using fine-mapping and functional tools, we find 308 high-confidence gene associations and enrichment of postsynaptic density and receptor clustering. A neural cell-type enrichment analysis utilizing single-cell data implicates excitatory, inhibitory, and medium spiny neurons and the involvement of amygdala neurons in both mouse and human single-cell analyses. The associations are enriched for antidepressant targets and provide potential repurposing opportunities. Polygenic scores trained using European or multi-ancestry data predicted MD status across all ancestries, explaining up to 5.8% of MD liability variance in Europeans. These findings advance our global understanding of MD and reveal biological targets that may be used to target and develop pharmacotherapies addressing the unmet need for effective treatment.

## In brief

Trans-ancestry GWAS of major depression identifies 697 genetic variants and 308 genes, implicating neural and molecular mechanisms and drug repurposing opportunities.

## Graphical Abstract

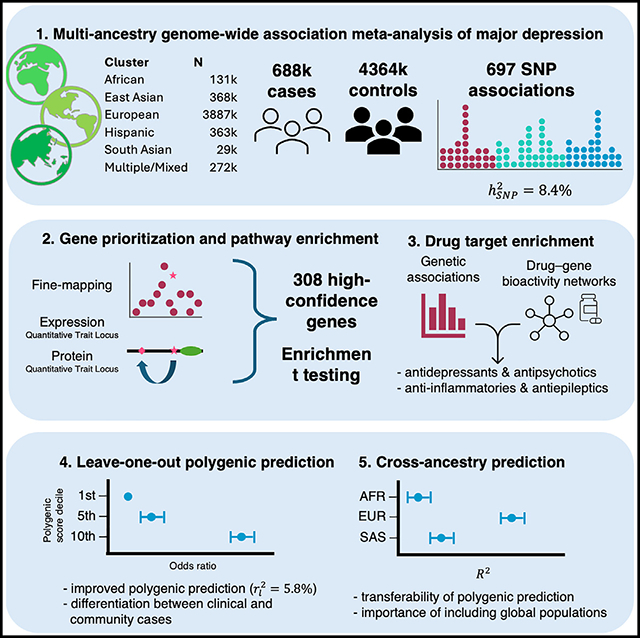

## INTRODUCTION

Major depression (MD) is a leading cause of worldwide disability and affects approximately 15% of the global population during their lifetime. The peak age of onset is in early adulthood, and the disorder is typically recurrent or chronic in nature, often with persisting disability despite pharmacological and psychological therapies. Twin and family-based studies provide evidence of a significant genetic contribution to its etiology, with a heritability of approximately 37%.^1^ Since 2013, genome-wide association studies (GWASs) have provided major insights into the polygenic nature of MD, its genetic risk factors, and underlying mechanisms.^2–9^ The largest study conducted to date reported 243 independent MD risk loci from a meta-analysis of the Million Veteran Program (MVP), 23andMe, UK Biobank, FinnGen, and iPSYCH, including 371K cases.^10^

Despite these efforts, the molecular, cellular, and neurobiological mechanisms of MD remain largely unidentified, limiting the development of disease models and mechanism-informed drug treatments.^11^ In this study, we report results from the Psychiatric Genomics Consortium (PGC) Major Depressive Disorder Working Group’s largest GWAS meta-analysis of MD to date (currently the largest GWAS study of any psychiatric disorder). We used strategies designed for the analysis of multi-ancestry and admixed populations to implement the largest, most inclusive study of MD genetics. These results substantially extend previous GWAS findings, implicating genes, cell types, and tissues in the etiology of MD, and demonstrate out-of-sample prediction across diverse ancestry groups.

## RESULTS

**Figure 1.**
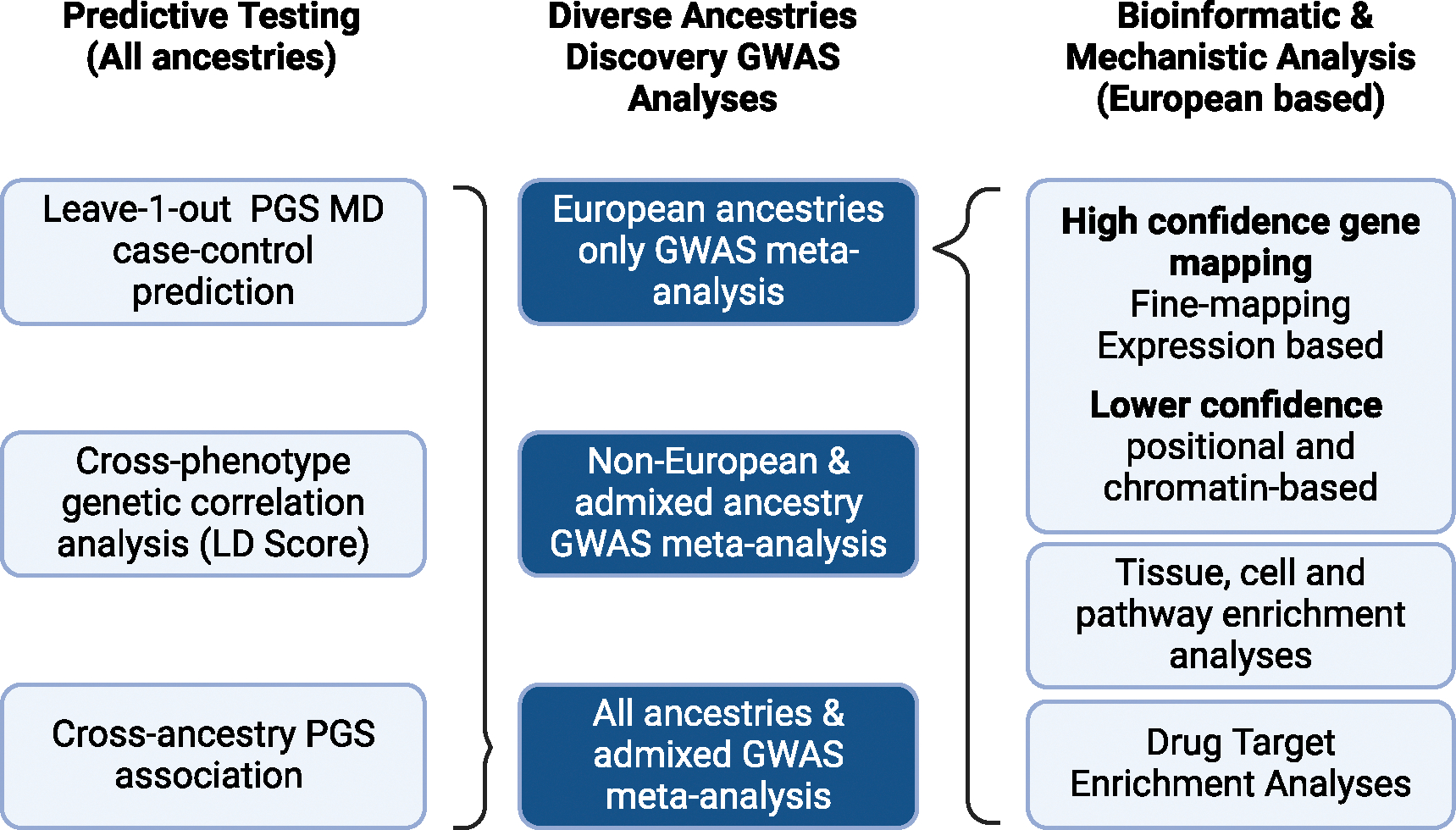
Overview of MD GWAS and downstream analyses Figure shows the 3 meta-analyses conducted (middle, deeper blue). Predictive testing using polygenic risk scores was conducted using both European and all ancestries GWAS summary statistics (left-hand side of the figure). Bioinformatic and mechanistic analyses were conducted using European-only GWAS summary statistics because many of the methods depend on a single suitable linkage equilibrium reference panel, and methods to generalize these approaches to trans-ancestry summary statistics were still in development at the time of submission.

The GWAS and subsequent downstream bioinformatic and predictive testing analyses are summarized in Figure 1.

### GWAS meta-analysis

**Figure 2.**
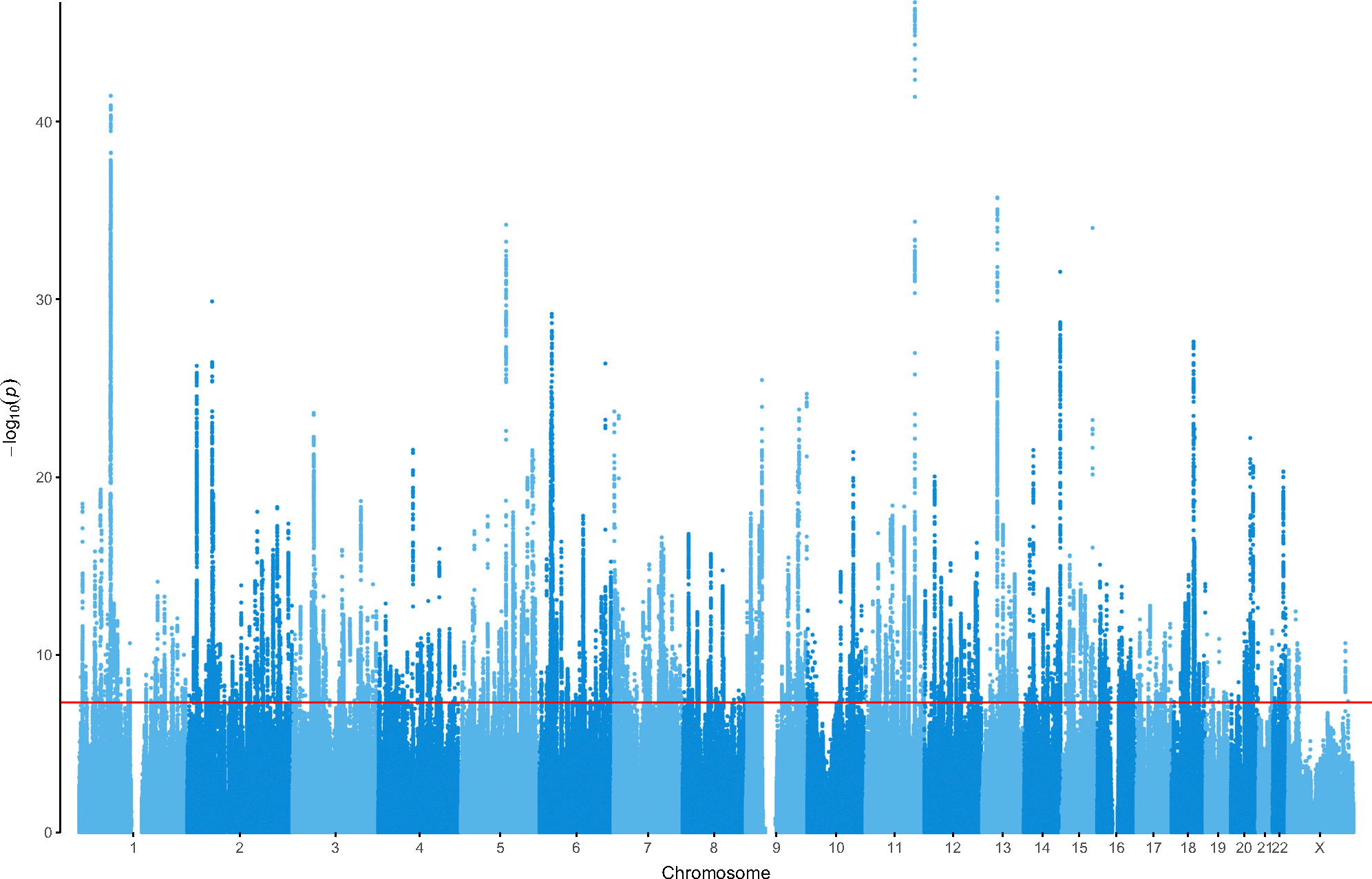
Manhattan plot of GWAS meta-analysis of 688,808 MD cases and 4,364,225 controls Manhattan plot displaying the significance of each SNP’s association with MD across the genome (vertical axis shows −log_10_ *p* value). Chromosomal position of each SNP is shown on the horizontal axis. The horizontal line at 7.3 (−log_10_(5 × 10^−8^)) indicates the genome-wide statistical significance threshold.

We meta-analyzed GWAS summary statistics from 109 ancestrally diverse cohort datasets with 688,808 MD cases and 4,364,225 controls (see STAR Methods, Table S1, Methods S1, and see key resources table). These studies had power equivalent to a case-control study of 1,004,459 cases and 1,004,459 controls, with 23% in diverse/non-European ancestries (Table 1). For cohorts with diverse ancestries, associations were assessed using tools that explicitly model population structure, admixture, and relatedness (GENESIS). For a subset of cohorts with ancestrally diverse samples, we compared the sample size using the commonly used strategy of assigning individuals into ancestry groups followed by logistic regression (*N* = 24,859) to our joint approach (*N* = 47,642) and found a 92% sample size increase. Our final sample size of 163,611 cases and 1,001,890 controls with diverse ancestries (Methods S1) led to an increase in the discovery of genome-wide significant loci compared with the European-only ancestry studies analysis. Using conditional-and-joint GCTA-COJO^12^ analysis with threshold *p* ≤ 5 × 10^−8^ within 10 Mb windows for the combined meta-analysis, we identified 697 significant independent single-nucleotide polymorphisms (SNPs) in 635 genomic regions. About half (293/635; 46%) of the discovered loci were novel MD associations (Figure 2; see key resources table) of which 100 were identified due to the inclusion of cohorts with ancestrally diverse samples. The European-only analysis identified 622 SNPs in 570 regions with a net change in the full meta-analysis of 65 (142 regions gained, 77 regions became non-significant).

In order to carry out downstream analyses, including heritability, gene prioritization, enrichment, and genetic correlation, we performed a fixed-effects meta-analysis for samples of European ancestries (525,197 cases and 3,362,335 controls), using a large single linkage disequilibrium (LD) reference dataset. The consequences of MD phenotyping on the meta-analyses were examined using genomic structural equation modeling (SEM) with a common-factor meta-analysis of the European ancestry summary statistics in genomic SEM^13^ (Figure S1). Cohorts were first meta-analyzed based on how the MD phenotype was determined: clinical/interview, electronic health record [EHR], questionnaire, or self-report of MD diagnosis. The proportion of total effective sample size contributed by each phenotype definition was 4% clinical/interview, 54% EHR, 14% questionnaire, and 27% self-report. The different phenotype definitions of MD had strong genetic correlations (LD score $r_{g}$ from 0.78 to 0.88). We fitted a common-factor model in genomic SEM and set the clinical/interview phenotype as the primary phenotype by fixing its factor loading to 1 and its residual variance to 0. This factor model was consistent with the data ($\chi_{3}^{2}=4.49$, *p* = 0.213); therefore, we could not reject the null hypothesis that a single factor capturing all the variance of the primary method explained the intercorrelations between the other depression phenotypes. Most MD phenotypes had strong positive loadings on the common factor (clinical/interview = 1.0 [reference], EHR = 0.92 ± 0.04, questionnaire = 0.95 ± 0.04), although the loading for self-reported diagnosis was lower (self-report loading = 0.85 ± 04). One locus showed significant SNP heterogeneity between phenotyping definitions (rs12124523 intronic variant in *NEGR1*, common factor association *p* = 8.4 × 10^−14^, Q heterogeneity *p* = 2.9 × 10^−10^, *I*^2^ = 0.71) with a stronger association found in self-reported depression studies (self-report odds ratio [OR] = 1.081, confidence interval [CI] = 1.065–1.098; other cohorts OR = 1.008, CI = 0.999–1.018). We found no evidence of heterogeneity at 569/570 loci, supporting the use of multiple phenotypes in genetic association studies of MD.

SNP-based heritability was estimated in European ancestries using SBayesS^14^ at 8.4% (SE 0.07%) on the liability scale (assuming lifetime MD risk of 15%) similar to prior estimates.^4,7^ Analyses of the genetic architecture using SBayesS estimated a polygenicity of 6% and selection parameter of −0.54. Compared with previously reported estimates for 155 traits, MD has a relatively higher polygenicity, but its associated variants are under weaker negative selection.^14^

### Gene prioritization and pathway enrichment analysis

We used a range of methods and functional genomic datasets to gain insight into the associated variants, genes, and pathways that may be dysregulated in MD. These included three rigorous “high-confidence” approaches: SNP-based fine-mapping of MD-associated loci and integration of expression and protein quantitative trait loci (eQTL and pQTL) to infer genetically driven MD case-control differences in RNA and protein expression. These are referred to as transcriptome- and proteome-wide association study approaches (TWAS and PWAS) and were reported when summary data-based Mendelian randomization (SMR), colocalization (COLOC), and expression-based fine-mapping (of eQTLs and pQTLs, in FOCUS) analyses all aligned to indicate a common gene. We also mapped associated loci to genes using standard gene-based association analysis in fastBAT, chromatin interaction datasets (Hi-C), and applied a gene prioritization package, psychiatric omnilocus prioritization score (PsyOPS) (see STAR Methods).

### SNP-based fine-mapping

We undertook functionally informed SNP-based fine-mapping analyses using the European ancestry GWAS findings, targeting all autosomal GWAS loci excluding the major histocompatibility complex (MHC) region. Twenty-four variants showed strong putative evidence of causality (posterior inclusion probability [PIP] > 0.95) at *IRF4*, *ESR1*, and *FURIN* (Table S2). Credible causal set sizes comprising ≤10 variants (cumulative PIP > 0.95) were identified at 224 loci, and 234/564 autosomal loci could be mapped to one or more genes (Table S2).

### RNA and protein-expression-based mapping

Both eQTL and pQTL data were used to infer upregulated or downregulated gene expression (TWAS) or protein (PWAS) levels associated with MD. Stringent criteria were used to identify high-confidence associations with MD (STAR Methods). MD genetic associations were found to correlate and colocalize with *cis*-regulated expression of 75 genes (Table S3) and *cis*-regulated levels of 10 proteins (Table S3). Results were only regarded as high-confidence when altered expression was supported by significant SMR and COLOC findings. No gene was identified as high confidence by both TWAS and PWAS analyses.

### Convergent high-confidence gene identification

In total, across SNP-based fine-mapping, eQTL, and pQTL analyses, 308 high-confidence associations were identified (Table S8). Fourteen eQTL genes and 1 pQTL gene also identified as high confidence by SNP-based fine-mapping. For example, SNP-based fine-mapping found all SNPs in one 95% credible set were within the cytochrome P450 gene *CYP7B1*, which was also inferred to have decreased expression in the dorsolateral prefrontal cortex of individuals with MD (TWAS *p* = 2.92 × 10^−15^, COLOC PP4 = 0.939, FOCUS PIP = 1). Additional signals identified by both fine-mapping and expression-based analyses included the genes *SP4*, *FURIN*, *DCC*, and the neurotrophin receptor kinase *NTRK3*.

### Other positional, chromatin-based, and bioinformatic approaches

Positional mapping approaches were used to identify additional genes that may be involved in MD etiology, including identification of the nearest gene to lead MD variants, aggregating genetic associations across gene regions using fastBAT (see key resources table), and linking associated loci to genes through Hi-C chromatin interactions using Hi-C coupled MAGMA (H-MAGMA) (see key resources table). Furthermore, the gene prioritization method PsyOPS was used to score genes based on prior information on mutational constraint, brain expression, and involvement in neurodevelopmental disorders (Table S4). Of the 18,737 genes assessed using fastBAT, 1,568 were associated with MD (*p* < 2.67 × 10^−6^) with the strongest evidence of association at the dopamine receptor D2 (*DRD2*) gene (*p* = 9.39 × 10^−47^). *DRD2* was also associated with MD by H-MAGMA in all four brain tissue profiles analyzed (*p* = 1 × 10^−10^ to 1 × 10^−15^). An additional 1,033 genes were also identified as associated with MD based on three-dimensional chromatin data using H-MAGMA. While PsyOPS prioritized a neighboring gene, *NCAM1* (PsyOPS score = 0.402), *DRD2* had an equivalent score (0.399). Other genes with high PsyOPS prioritization scores were *PTPRT*, *SLC12A5*, *RFX3*, *ELAVL2*, *HCN1*, *KIF5A*, and *SHANK3*.

### Synaptic gene set enrichment

We used the high-confidence gene list from SNP-based fine-mapping, TWAS, and PWAS (subset of Table S8) to identify enriched synapse functions using Synaptic Gene Ontologies (SynGO).^15^ The 43 genes from the high-confidence gene list with SynGO annotations were compared against a background of 18,035 brain-expressed genes. We replicated earlier findings from Howard et al.,^2^ showing enrichment of neuron differentiation processes and postsynaptic membrane components. The current GWAS provided greatly increased specificity, implicating the cytosol, active zone membrane, calcium levels, vesicle cycle, and presynaptic endocytosis. At the post-synapse, there was enrichment of synaptic specialization, density, and receptor clustering (Tables S5A and S5B).

### Tissue and cell-type enrichment analysis

**Figure 3.**
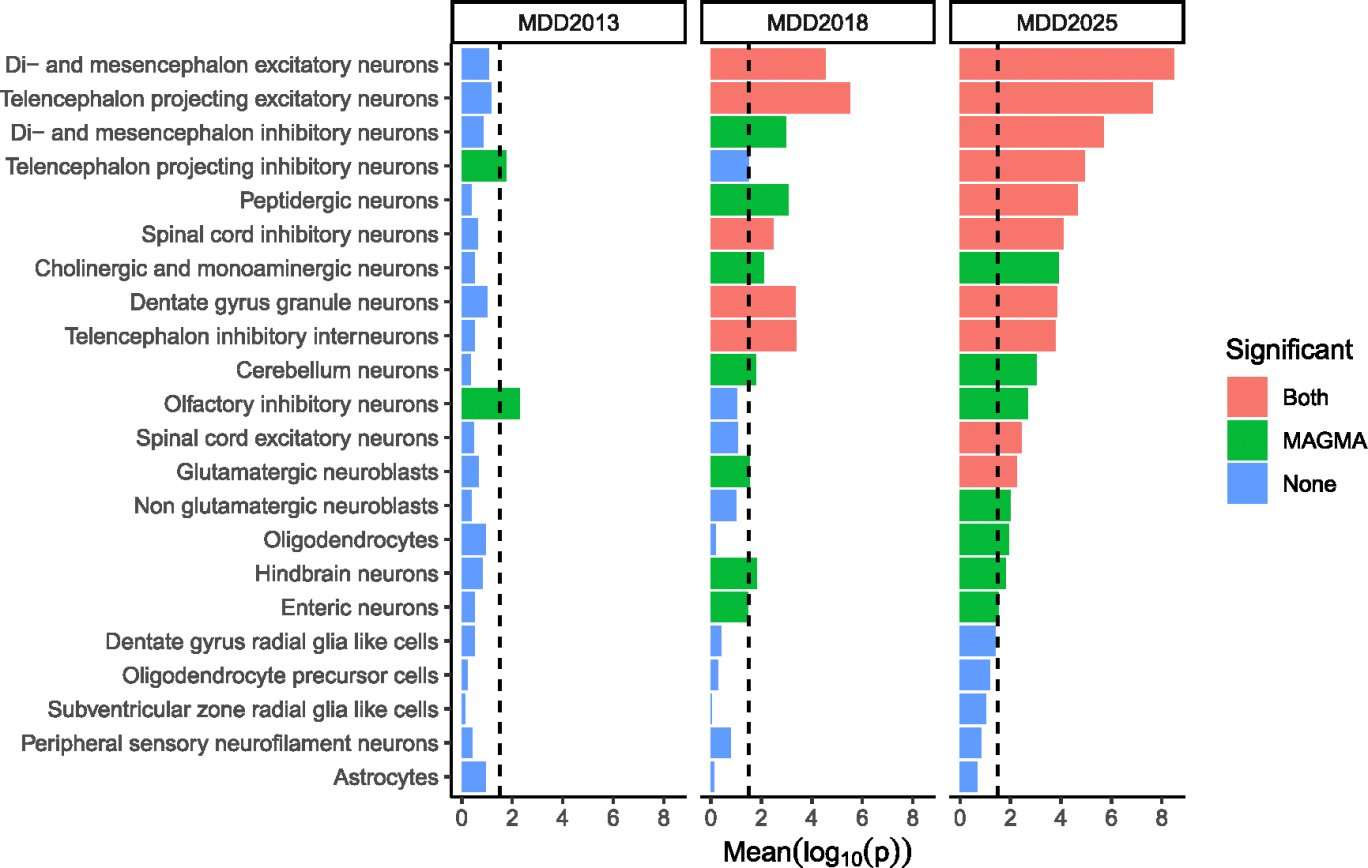
Broad brain cell category enrichment analysis Cell-type enrichment analysis. 20 categories of brain cell types are listed (from a total of 39 broad brain cell-type categories tested) along the vertical axis, and horizontal bar size represents the significance of the enrichment measured using MAGMA gene set enrichment test or partitioned LDSC. Color encodes results that were significant after false discovery rate correction. Bars in salmon color represent enrichments significant using both methods; green, MAGMA only; blue, partitioned LDSC only; and purple when neither method showed significant enrichment. 19 broad categories not displayed were not significant using either method. Columns represent the results of each test using summary statistics from MDD2013, MDD2018, and this study. The dotted line shows threshold of nominal (uncorrected) statistical significance.

We conducted tissue and cell-type enrichment analysis using published expression datasets including bulk RNA sequencing data from human tissues^16^ and single-cell RNA sequencing data from the adult mouse central and peripheral nervous system.^17^ Across human tissues, we found clearer enrichment patterns of MD SNP-heritability in neural tissues using the current GWAS association findings than those obtained from the previous two PGC Major Depressive Disorder (MDD) group analyses (Figure 3; Tables S5C–S5E). In the adult mouse central and peripheral nervous system, we found significant enrichment of MD SNP-heritability in 10 out of 39 cell types with two different methods (MAGMA and partitioned LD score regression [LDSC]; see key resources table). We confirmed all the cell types identified in the previous GWAS,^7^ including both excitatory and inhibitory neurons, but implicate multiple additional inhibitory neuron categories and peptidergic neurons.

Analysis at a more refined level of murine cell types further emphasized the enrichment in excitatory and inhibitory neurons in multiple brain regions (key resources table; Tables S5I–S5K). Associated cell types using both methods included midbrain (mouse atlas reference: MEGLU7, MEGLU8, MEGLU10, and MEGLU11), amygdala (TEGLU22), hippocampal (CA1 and TEG LU21), thalamic (DEGLU4), and cortical (TEGLU1, TEGLU4, TEGLU8, TEGLU8, TEGLU11, TEGLU13, and TEGLU20) excitatory neurons. We also found additional evidence for the involvement of D1/D2 midbrain and striatal medium spiny neurons (MSN2 by both methods and MSN1,3–4 by MAGMA only).

Furthermore, we performed cell-type enrichment analyses using a human brain single nucelli RNA sequencing dataset.^18^ We found enrichment of expression signals for amygdala excitatory neurons and of medial ganglionic eminence (MGE) and caudal ganglionic eminence (CGE) interneurons by both MAGMA and LDSC. MAGMA also implicated further neuronal cell clusters as well as oligodendrocyte precursors at the broader cell-type level (superclusters).

### Drug target enrichment analysis

Using Drug Targetor, we searched for therapeutic agents grouped according to organ and mode of action using their Anatomical Therapeutic Chemical (ATC) drug class and identified targets that were enriched in the association signals from the GWAS analysis.^19^ Drug Targetor harnesses drug bioactivity data to prioritize drugs and targets for a given phenotype. Replicating an earlier analysis, we found the gene targets of antidepressants (ATC class N06A) are significantly enriched (see key resources table) in our association findings. Other drug classes that were significantly enriched included antipsychotics (N05A), which include some medicines with antidepressant effects.

The gene targets of *specific drugs* were also enriched in genetic associations with MD, although the analysis does not infer whether the effects of these agents were more likely to be congruent or opposed to the effects of genetic risk. The identified drugs provide possible repurposing opportunities and examples included several anti-cancer therapies and the agents pregabalin (used in the management of pain and anxiety) and modafinil, which is used to treat daytime sleepiness caused by narcolepsy (Table 2; key resources table).

### Within- and cross-trait prediction

#### PGS prediction in European ancestry samples

**Figure 4.**
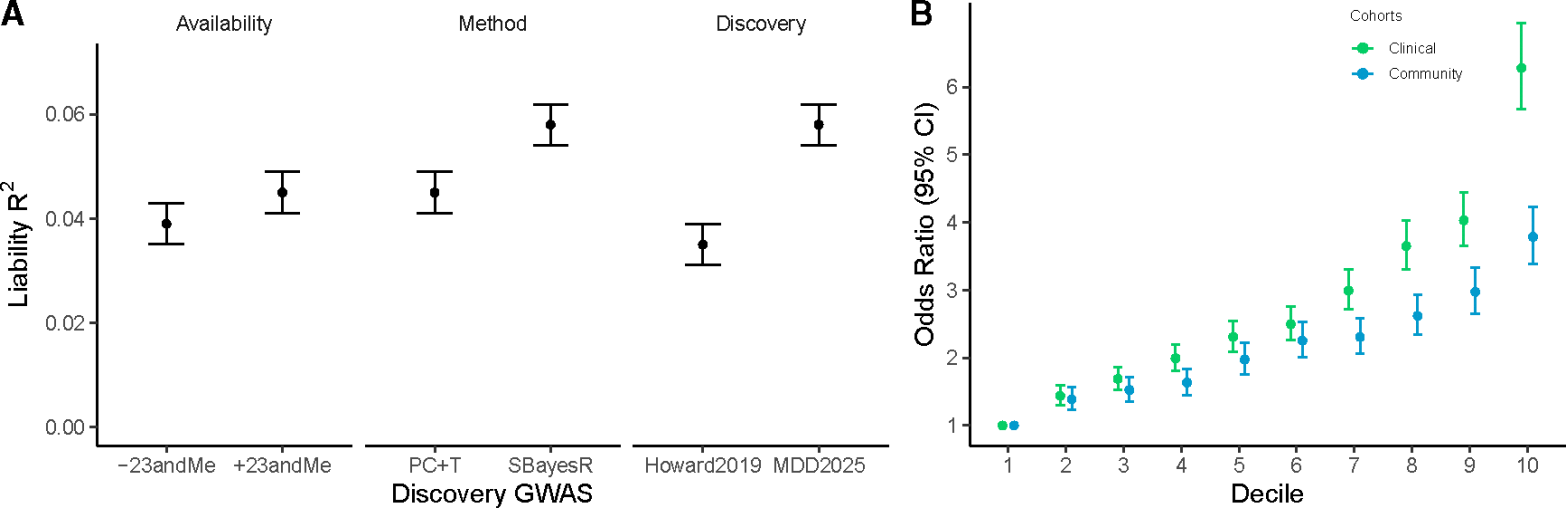
MD polygenic score prediction into European ancestry studies (A) Comparison of liability R^2^ by input summary statistics by availability (full dataset with 23andMe versus public dataset without 23andMe, using *p* value clumping + thresholding at *p* ≤ 0.05 [P+CT]), PGS method (P+CT versus SBayesR), and discovery dataset (previous Howard et al.^2^ versus current MDD2024 SBayesR). The R^2^ are estimated across 42 cohorts with individual-level data. For the discovery panel, the R^2^ are estimated from the 20 cohorts with individual-level data contributed to the PGC after the Howard et al.^2^ study. The $r_{l}^{2}$ was calculated using a lifetime prevalence of 0.15. (B) Odds ratio by decile, with reference to decile 1, for clinical and community-ascertained studies (SBayesR). Bars reflecting the 95% confidence interval (CI) are based on estimates from the logistic regression.

Using the case-control cohorts in the meta-analysis, we conducted a leave-one-cohort-out GWAS meta-analysis for 42 European ancestry cohorts that had provided individual-level data. Polygenic scores (PGSs) were generated in each cohort using SNP weights for the multi-ancestry and the European ancestry meta-analyses derived using SBayesR.^20^ Other PGS methods, including the standard *p* value clumping and thresholding, gave similar results (Table S6). Across all European ancestry cohorts, the variance explained on the liability scale $\left(r_{l}^{2}\right)$ was 5.8% (SE 0.2%) (see key resources table and Table S6), with an area under the receiver operating characteristic curve (AUC) statistic of 0.625 (see key resources table). Adding functional annotations into the algorithm to generate SNP weights for PGSs (SBayesR) increased prediction accuracy by 0.1% (i.e., $r_{l}^{2}$ of 5.9%). The $r_{l}^{2}$ was more than 1.4 times greater than that reported in the PGC MDD 2018 analysis^7,21^ (Figure 4). The OR for being a case per standard deviation (SD) increase in PGS was 1.57. The OR for being a case in the tenth compared with the first decile of PGSs was 4.92 (95% CI 4.57–5.29) (Figure 4), and the OR for the top versus bottom centiles was 11.8 (95% CI 8.4–15.2) (Figure 4). Heterogeneity in the out-of-sample prediction results could be partly explained by the recorded ascertainment type (Figure 4; key resources table), which we classified as “clinical” (12 cohorts; ascertained from in- or out-patient settings or EHR) or “community” ascertained (30 cohorts; interviews or questionnaires self-reporting on lifetime depression). The difference in mean PGS between clinical versus community cases was 0.131 (SE 0.012, *p* < 2 × 10^−16^) control sample SD units. The non-linear shape of these decile plots is expected under a polygenic architecture.^22^

#### Cross-ancestry prediction of MD

**Figure 5.**
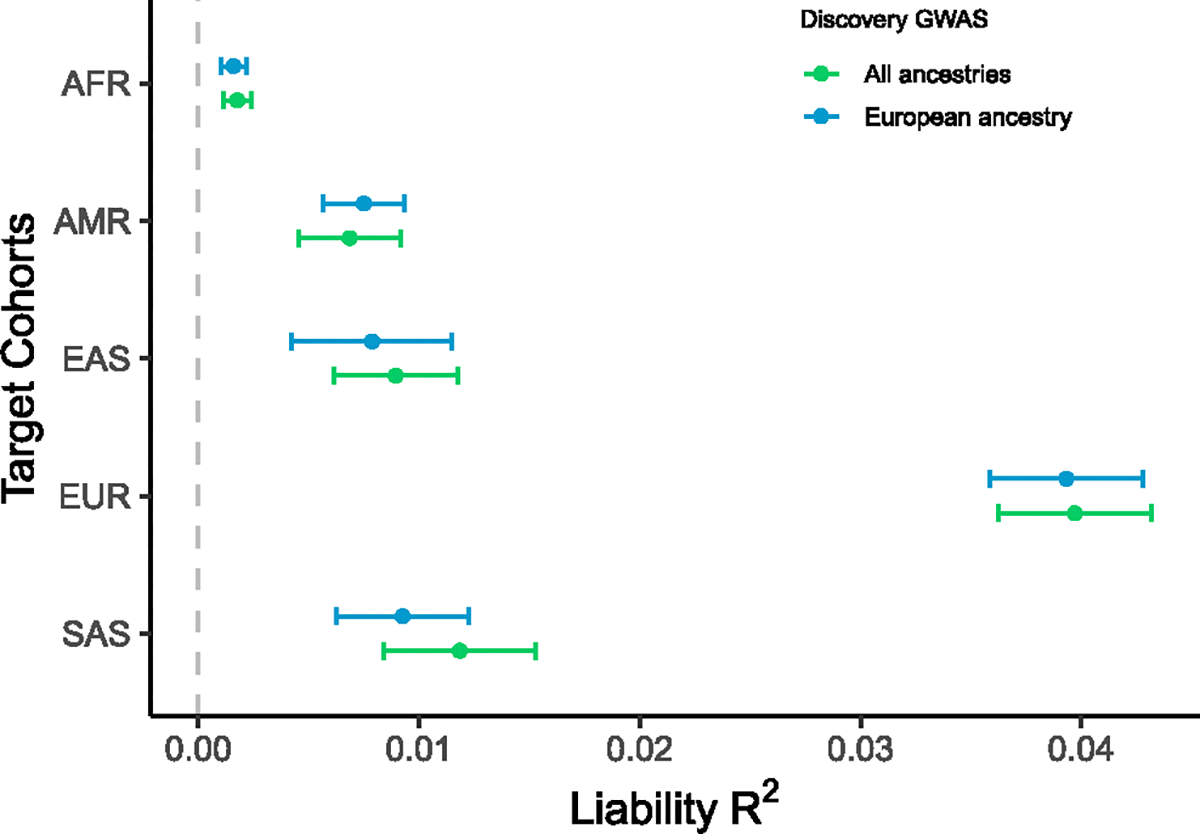
Polygenic prediction of MD status from European and multi-ancestry GWAS into ancestrally diverse non-European studies Details of cohorts found in Table S1. The $r_{l}^{2}$ was calculated using a prevalence of 0.15 with the P+CT method. The error bars are confidence intervals calculated using bootstrap. The training data did not include 23andMe because of access limitations. AFR, African ancestry; AMR, Hispanic and Latin American ethnicities; EAS, East Asian ancestries; EUR, European ancestries; SAS, South Asian ancestries.

We used data from 9 diverse ancestry studies to assess PGS transferability (Table S7A) using PGS derived from the clumping and thresholding approach. The PGSs were derived from the multi-ancestry and the European ancestry meta-analysis, excluding 23andMe ($N_{\text{effective}}=739,180$ and 576,327, respectively). In the diverse ancestry studies, the $r_{l}^{2}$, by the PGS based on the European ancestry training data ranged from ~0.6%–4.5%. *The $r_{l}^{2}$* values for prediction into European ancestry (excluding 23andMe) were 3.9% (SE 0.2%) using P_T_ = 0.05 (Figure 5; Table S7B). Values were lowest in studies with participants of African descent, and in the largest African ancestry study, the MVP, the PGS was not associated with MD $r_{l}^{2}=0.0018$). Results using the multi-ancestry summary statistics showed only minor and non-significant differences from European-only PGS GWAS-trained scores in all ancestry groups.

## DISCUSSION

This study represents the largest and most inclusive GWAS of MD to date, identifying 697 independent SNP associations located within 635 independent genetic loci and evidence that neuronal differentiation and receptor clustering are involved in the etiology of the disorder. 308 high-confidence gene associations were identified (summarized in Table S8) in European ancestries. There was convergent evidence from multiple approaches for 15 genes, such as *CYP7B1*, a gene encoding a cytochrome P450 enzyme involved in neurosteroid synthesis. However, the results of each gene prioritization approach were largely distinct, potentially representing the differential sensitivity of each approach to variants within (fine-mapping) or outside (regulatory) gene boundaries. Results from conventional gene-association and chromatin interaction mapping approaches also implicated DRD2 involvement in MD. Previous work has shown that DRD2 inhibition suppresses neuroinflammation in mice,^23^ supporting a potentially testable mechanism linking genetic variation to MD.

Our results confirm and extend previous findings showing the enrichment of expression signals in excitatory and inhibitory neurons. Importantly, the increased power in this genetic analysis provided additional evidence for involvement of amygdala and hippocampal excitatory neurons, including granule cells and medium spiny neurons. The amygdala and hippocampus have been previously implicated from a wide range of human imaging^24,25^ and animal studies of depression^26–28^ and medium spiny neurons have also been previously implicated in animal studies of reward and are linked to depressive behaviors.^29,30^ The enrichment of expression signals in granule cells is of particular interest given the renewal of this cell type throughout adult life in the dentate gyrus,^31^ its role in stress resilience,^32^ and the increased hippocampal granule cell expansion associated with antidepressant treatment.^33^ Together, these findings underline the mechanistic insights provided by the expansion of GWAS to over half a million depressed individuals.

Lack of ancestral and global diversity remains a significant concern for GWAS, with 86% of studies conducted in participants of European ancestry.^34^ Our study included data from 163,611 cases and 1,001,890 controls of non-European diverse ancestries. Unlike most other multi-ancestry GWAS, we used a joint analysis approach and did not exclude individuals with mixed ancestry or ancestry not represented in reference sets. This is becoming ever more important as the number of people with mixed ancestry is increasing in countries such as the USA and the UK.^35^ Overall, the additional ancestrally diverse participants helped identify 100 novel genetic associations and enabled us to demonstrate significant genetic risk prediction across diverse ancestry groups.

Using PGSs, the proportion of variation in liability to MD explained in European ancestry case-control studies also showed a considerable increase from an *R*^*2*^ of 3.2% in our previous analyses to 5.8% using SBayesR. We also show a significant MD prediction in diverse non-European and admixed ancestries. The SNP-*h*^2^ in this study of 8.4% implies that approximately 69% of the additive genetic variance for MD associated with common SNPs across studies can now be accounted for by PGSs. This study provides the first evidence of limited transferability of MD PGS to multiple diverse ancestries and further emphasizes the importance of conducting future GWAS studies across different global populations, especially in Africa, where transferability is poorest. While we did not find evidence for improved prediction based on multi-ancestry rather than European-only PGS, this may be due to the small proportion of participants *within* each individual ancestry group (23% of individuals of non-European ancestries were divided across 4 major ancestry and admixed groups) relative to the European ancestry group alone.

Genome-wide association signals for depression also showed enrichment for the targets of antidepressants, suggesting that they may also help to reveal other effective treatment targets and more effective interventions. Pregabalin^36–39^ and Modafinil^40^ are both supported by sparse non-randomized evidence supporting their efficacy in depression and related conditions. Our findings provide further proof of principle that GWAS is a useful means of identifying therapeutically relevant drug targets and treatments.

Together, these findings highlight the value of ancestrally diverse genetic studies to prioritize the study of pathophysiological processes in MD. The clearer association of genetic variants with altered gene expression and the enrichment of antidepressant targets provide confidence that genetic association findings will be relevant to the development, deployment, or repurposing of pharmacotherapies. Critically, these findings suggest genetic associations will point to new drug targets and more effective therapies that may reduce the considerable disability caused by depression.

### Limitations of the study

The current meta-analysis is limited by the low proportion of participants of non-European ancestries (76.6% of people with MD were of European ancestry) who were genotyped using arrays developed in European populations. This may reduce the power to discover or test the cross-ancestry transferability of genetic variants. Without larger and more globally representative samples, it is not clear whether (or to what extent) the genetic architecture of MD differs by ancestry or whether there are genetic differences between ancestral populations recruited from their regional origin versus those recruited from diasporas.

### RESOURCE AVAILABILITY

#### Lead contact

Andrew M. McIntosh, University of Edinburgh (andrew.mcintosh@ed.ac.uk).

#### Materials availability

This study did not generate new unique reagents. Samples analyzed as part of this published work may be available for further assays and, in some cases, upon application to the contributing study investigators. This information is available in the Methods S1 file.

#### Data and code availability

- Summary statistics are available from Figshare through the following link https://pgc.unc.edu/for-researchers/download-results/ (https://doi.org/10.6084/m9.figshare.27061255). These data are publicly available as of the date of publication.
- Individual data are made available following an approved application to the PGC Data Access Committee (https://pgc.unc.edu/for-researchers/data-access-committee/). These data are available as of the date of publication.
- Available summary statistics, including 23andMe data, require an approved application to 23andMe here: https://research.23andme.com/dataset-access/. These data are available as of the date of publication.
- Summary statistics for the Genetic Association Information Network (GAIN), NeuroGenetics Research Consortium (NGRC), Gene Environment Association Studies Initiative (GENEVA, Melanoma Study), and other studies are available from The Database of Genotypes and Phenotypes (dbGaP: https://dbgap.ncbi.nlm.nih.gov/). These data are available as of the date of publication. Instructions on how to access dbGap data are available here: https://www.ncbi.nlm.nih.gov/gap/docs/submissionguide/.
- Additional deposited reference dataset availability is here: Haplotype Reference Consortium (European Genome-Phenome Archive, https://ega-archive.org), GTEx v8 (GTEx Portal, https://gtexportal.org), Human Brain Cell Atlas (CELL×GENE Discover, https://cellxgene.cziscience.com/), eQTLGen (https://www.eqtlgen.org), MetaBrain (https://www.metabrain.nl), Brain pQTL (AD Knowledge Portal, https://adknowledgeportal.synapse.org), and SynGO (https://syngoportal.org/).
- Additional quality control information, gene-based association summary statistics in fastBAT (including figures), Hi-C, genetic correlation results, full drug target enrichment findings, single-cell enrichment figures, and PGS plots are also available for download from Figshare through the following link: https://pgc.unc.edu/for-researchers/download-results/ (https://doi.org/10.6084/m9.figshare.27089614). See STAR Methods for a key resources table. These data are publicly available as of the date of publication.
- Project code is available from https://github.com/psychiatric-genomics-consortium/mdd-wave3-meta.

## STAR★METHODS

Detailed methods are provided in the online version of this paper and include the following:

### EXPERIMENTAL MODEL AND STUDY PARTICIPANT DETAILS

#### Samples

##### Overview

Here, we report the third GWAS from the Major Depressive Disorder (MDD) Working Group of the Psychiatric Genomics Consortium (PGC MDD2023), We conducted a genome-wide association mega-analysis of major depression (MD) in 49 cohorts of European ancestry (“MDD49”) with combined 28,147 cases and 48,033 controls (defined by the original study authors, but defined as individuals without MD, schizophrenia or bipolar disorder). We then meta-analyzed the MDD49 results with summary statistics from 24 additional cohorts of European ancestry. We subsequently carried out a multi-ancestry meta-analysis, adding data from 160,611 cases and 1,001,890 controls with diverse ancestry, for a total sample size of 688,808 cases and 4,364,225 controls (the “discovery GWAS”). This sample size has the power equivalent to a balanced study (with equal numbers of cases and controls) with a total sample size of *N*_*eff*_ = 2* 1,000,101. For some cohorts, case status was based on self-report and did not reach the formal criteria of MDD. Hence, we use the term major depression (MD) to define case-ness.^54^

#### MDD49

The core PGC MDD49 cohort set builds on the MDD29 sample from Wray et al.^7^ These cohorts provided individual phenotype and genotype data for quality control, imputation, and analysis. The 49 cohort names, sample sizes, and inclusion/exclusion criteria are summarised in Table S1 and more details can be found in Methods S1. Most cohorts provided both cases and controls. For case-only cohorts, cohorts were either merged or matching controls were obtained from other PGC groups. Cases met international consensus criteria for a lifetime diagnosis of major depressive disorder (ICD9, ICD10, DSM-IV, or DSM-5). Cases were classified into *clinical studies* (where diagnoses were established using structured diagnostic instruments by clinicians, trained interviewers, or medical record review) and *community studies* (where structured diagnostic instruments were used for diagnosis of life-time major depression, or self-report of an MDD diagnosis). Controls were either screened for the absence of MDD and other mood disorders or selected randomly from the population.

#### Additional cohorts

Many cohorts cannot share individual phenotype and genotype data but can contribute case-control GWAS summary statistics. We incorporated summary statistics from 24 independent cohorts of European ancestry (496,710 cases and 3,010,973 controls) into the meta-analysis. Building on Meng et al.,^9^ we also incorporated data from ancestrally diverse cohorts, including 8 cohorts with participants of African ancestry (9,649 cases and 122,347 controls), 7 with East Asian ancestry (18,709 cases and 349,619 controls), 1 with South Asian ancestry (3,748 cases and 25,934 controls), 5 with Hispanic/Latin American ethnicity (19,927 cases and 340,403 controls). For the first time our analyses accommodated cohorts with participants of diverse and mixed ancestry by using a joint analysis approach (12 cohorts, 108,578 cases and 163,587 controls). We excluded participants of European ancestry from these studies where they had already been included in the analyses described above. The numbers of cases and controls, and MDD assessment methods are summarized in Table S1. Additional information, including genotyping, quality control and imputation are described in the Methods S1. Methods for determining MD status included clinical interviews, health register or medical records, self-reported questionnaires, and self-report of diagnosis.

### METHOD DETAILS

#### Genome-wide association study meta-analysis

##### Technical Quality Control (QC) of the 49 cohorts in the primary PGC sample

Technical QC was performed on single nucleotide polymorphisms (SNPs) in each core PGC study, applying standard PGC criteria including SNP missingness < 0.05 (before sample removal); sample missingness < 0.02; autosomal heterozygosity deviation (| Fhet | < 0.2); SNP missingness < 0.02 (after sample removal); difference in SNP missingness between cases and controls < 0.02; and SNP Hardy-Weinberg equilibrium (HWE: P > 10^−6^ in controls or P > 10^−10^ in cases). For chromosome X (chrX) genotypes, we applied the above QC to the males and females separately.^41^

##### Genomic Quality Control: Principal Component Analysis (PCA) and Relatedness Checking

Within all 49 cohorts we performed PCA using autosomal SNPs with high imputation quality (INFO >0.8), low missingness (<1%), MAF>0.05 and in relative linkage equilibrium after 2 iterations of linkage disequilibrium (LD) pruning (*r*^2^ < 0.2, 200 SNP windows), removing well known long-range-LD areas (MHC and chr8 inversion). 17,608 SNPs present in all 49 cohorts, followed by the above LD pruning were used for robust relatedness testing across cohorts using PLINK v1.9^55^; pairs of subjects with PIHAT > 0.2 were identified and one member of each pair removed at random, preferentially retaining cases over controls.

To control for false positive associations due to inflated test statistics we evaluated the effectiveness of the primary technical and genomic quality control parameters on the genome-wide inflation of test statistics using the lambda GC (median)^56^ and as necessary made the QC parameters more stringent until this value was between 0.981 and 1.173 (before inclusion of principal components as covariates) and/or between 0.977 and 1.068 after inclusion of PCA covariates. Additionally, we applied loose PCA filters for strongly stratified datasets even if we did not observe strong inflation of test statistics to retrieve reliable test statistics (Figure S1 shows PCA plots for all cohorts). Since the core PGC cohorts came from many distinct centres, countries, and continents, various measures (e.g., tightening of the technical QC parameters and/or genomic quality control) had to be taken in an iterative process to achieve this goal.

In summary we retained between 219K and 1.7M autosomal SNPs and 2476 to 28780 chromosome X SNPs in each cohort. For a detailed list of excluded individuals and SNPs at various QC steps described above see (Table S2B).

##### Imputation of the core PGC dataset

Genotype imputation of case-control cohorts was performed using the pre-phasing/imputation stepwise approach implemented in EAGLE 2 / MINIMAC3^57,58^ with 132 genomic windows of variable size and default parameters. The imputation reference consisted of 54,330 phased haplotypes with 36,678,882 variants from the publicly available HRC reference, release 1.1.^59^ Chromosome X imputation was conducted using individuals passing quality control for the autosomal analysis. Chromosome X imputation and association analysis was performed separately for males and females.

### QUANTIFICATION AND STATISTICAL ANALYSIS

#### Association / Meta-analysis in the core PGC dataset

In each cohort, association testing was based on an additive logistic regression model using PLINK.^55^ As covariates we used a subset of the first 20 principal components (PCs), derived within each cohort. By default, we included the first 4 PCs and thereafter every PC that was nominally significantly associated (p<0.05) with case-control status. We conducted a meta-analysis of the results using a standard error inverse-weighted fixed effects model. For chrX, gene dosages in males were scored 0 or 2, in females, 0/1/2, then association analysis was conducted separately for males and females and meta-analysed. We summarized the associations as number of independently associated index SNPs. Index SNPs were LD independent and had *r*^2^ < 0.1 within 3 Mb windows. We recorded the left and rightmost variant with *r*^2^ <0.1 to an index SNP to define an associated clump. To define loci, we added a 50kb window on each side of the LD clump and combined overlapping LD-clumps into a single locus. Due to the strong signal and high LD in the MHC region, only one SNP was kept from the extended MHC region (chr6:25–35Mb).

#### Joint analysis model for ancestrally diverse cohorts

We fitted ancestry-aware mixed models for 12 cohorts with ancestrally diverse and admixed participants. These were conducted using GENESIS Bioconductor package in R, which was developed for large-scale genetic analyses in samples with complex structure including relatedness, population structure and ancestry admixture.^60^ Genotyped variants for each study were first pruned, and the KING-robust method was used to estimate relatedness in the first instance. Subsequently, PC-Air was employed to calculate PCs using the kinship matrix derived from KING-robust method and the pruned variants. PC-Relate was used to re-estimate relatedness utilizing PCs from PC-Air. To enhance precision, a second iteration of PC-Air and PC-Relate was performed. Afterwards, we fitted a null model for MD case-control status, using sex, age, all 32 PCs from PC-Air, and the kinship matrix from PC-Relate. Finally, score tests were conducted using the null model and all imputed variants as predictors. Due to computational limitations, the Million Veterans Program was partitioned into 19 batches, which were then combined using an inverse variance weighted meta-analysis, implemented in METAL. To derive an estimate of the odds ratio (OR) and its standard error from the score test, the following conversion algorithm was applied: 1) logOR = Score/Variance, and 2) SE = 1/sqrt(Variance).^61^

#### Post-imputation quality control procedures

Summary statistics were aligned to chromosome-position scaffolds for Genome Reference Consortium Human Build 37 (GRCh37/hg19) and marker names were obtained from the Haplotype Reference Consortium (HRC) v1.1.^59^ Summary statistics on a different genome build were lifted over to GRCh37 using rtracklayer v1.48.0^62^ with UCSC Chain Files and removing positions that are not comparable between genome builds.^63^ We then used DENTIST^42^ to identify any heterogeneity between each set of summary statistics and the HRC LD reference. We calculated F_ST_ of SNP allele frequencies between each sample and the reference using the Hudson estimator,^64,65^ fit a beta distribution to the observed F_ST_ values, and identified outliers that exceeded an upper quantile in the fitted distribution of 0.05 divided by the number of variants tested in the summary statistics. We removed SNPs: DENTIST or F_ST_ outliers; minor allele frequency in cases and controls < 0.001; a minor allele count in cases and controls of < 20; imputation INFO score < 0.1; or that had alleles that were inconsistent with the reference sample. For each set of summary statistics we calculated the median odds ratio (OR) and standard error (SE) of the association statistics for SNPs with MAF > 0.01. We checked that median ORs were close to 1 and plotted median SEs against effective sample size to detect potential effect size scaling errors. We estimated pairwise LDSC genetic correlations^44^ among all cohorts and inspected genetic covariance intercepts for evidence of sample overlap. Pairs of studies with covariance intercepts > 0.025 were returned to cohort analysts for scrutiny to resolve potential sample overlap or close relatedness between sub-cohorts. For the diverse ancestry cohorts, only variants with an imputation information score of 0.7 or higher were deemed eligible. Furthermore, for studies with a sample size smaller than 10,000, a minor allele frequency (MAF) of at least 5% was required; for larger studies, we required a minimum effective sample size (N_eff_) of 50, calculated as N_eff_ = 2 × MAF × (1-MAF) × N × Info, where ‘Info’ represents the imputation quality score, ‘MAF’ is the minor allele frequency and ‘N’ is the actual sample size.

#### Genome-wide association and fixed effects meta-analysis

After quality control, we meta-analysed genotype and summary statistics samples together using Ricopili version 2019_Jun_18.001^41^ with HRC v1.1 as a reference panel. We identified genome-wide significant SNPs at *p* <= 5 × 10^−8^ and then identified independently associated index SNPs by clumping SNPs with *p* <= 1 × 10^−8^, INFO >= 0.6 that were r^2^ > 0.1 and were within 3 Mb windows of an index SNP. The extended MHC region was considered as a single region for clumping. We ran a conditional-and-joint (COJO) analysis^12^ on each region to identify SNPs that were associated after conditioning on the index SNP, using UK Biobank as the reference panel, and retained SNPs selected by COJO with joint *p* <= 5 × 10^−8^. To reduce variation in per-SNP effective sample size due to missingness across cohorts, we filtered SNPs to those with effective *N* >= 80% of the maximum effective *N* (calculated separately for autosomes and chrX). We examined discovery power in comparison to previous GWAS of depression using *genpwr*.^66^ To determine novelty of association findings, we looked for overlap in regions from recent MDD meta-analyses^2,6,7,54,67^ and associations for unipolar depression (EFO_0003761) in the GWAS Catalog.^68^

#### Common-factor meta-analysis

To examine the role of how MDD status was ascertained and phenotyped,^69^ we meta-analysed European ancestry studies together based on phenotyping approach (clinical, electronic health records, questionnaire, or self-reported diagnosis) and then analysed the grouped meta-analysis summary statistics in Genomic SEM.^13^ We estimated LD Score genetic correlations between each phenotype approach and conducted a common factor GWAS to test for heterogeneity in SNP effects across approaches. We fit a one-factor model where the loading factor on the Clinical/Interview phenotype was fixed to 1, so that the latent factor explains all the variance in clinical depression. We tested the hypothesis that the covariance matrix implied by the model differed from that observed in the input data.

#### SNP-based heritability and Genetic Correlation estimation

SNP-based heritability was estimated using SBayesS^14^ assuming lifetime risk of 15% for comparison with previous work. SBayesS was also used to provide estimates of polygenicity and selection. We estimated genetic correlations with other traits using LDSC. First, we searched for COJO-selected SNPs in the OpenGWAS catalogue^70^ at the standard lookup threshold (p < 1 × 10^−5^) and fetched full summary statistics for phenotypes returned by the query. We then estimated LD Score genetic correlations between each phenotype and MD,^44^ using false discovery rate (FDR) with *q* < 0.05 to correct for multiple testing. We then compared our results with previous MD GWAS analyses.

#### Polygenic analysis

##### Out of sample prediction

Of the case-control studies in the meta-analysis of European cohorts, 48 provided individual level data for analysis of which 43 were available for polygenic scoring. For these cohorts, we conducted a leave-one-cohort out GWAS meta-analysis to allow generation of polygenic scores (PGS) in the left-out target sample. Given a high variation in the effective sample size contributing to each SNP, we restricted to the set of SNPs with (*N*_*eff*_) ≥ max(Neff)*0.8, minor allele frequency > 0.05 and INFO > 0.75 in the full multi-ancestry analysis, resulting in 4.34 million SNPs. Preliminary analyses using the QC tool DENTIST^42^ justified this choice. We generated polygenic scores (PGS) on all individuals using two methods. A PGS is the sum of risk alleles weighted by the risk allele effect size; methods differ in the SNPs included and the effect sizes applied. To enable comparisons with previous publications, PGS were generated using the basic p-value clumping and thresholding (P+CT) method (LD clumping *r*^*2*^ threshold of 0.1, clump window of 500kb, 10 p-value thresholds). We also generated PGS using SBayesR,^20^ which is one of several methods that has been found to improve accuracy of PGS compared to C+T by better choice of the SNPs and their weights (derived from the GWAS effect sizes) through modelling of the genetic architecture. Of these methods we chose SBayesR because it ranked high in a study comparing methods,^21^ requires no tuning sample to estimate hyper-parameters and is computationally less demanding. We used the software recommended LD reference sample (*sbr_ldmatrix.band.mldm*) to infer the expected correlation structure between SNP association statistics. We also used the SBayesRC, which is an extension of SBayesR which uses functional information in the SNP weighting algorithm.^71^ For comparisons with the C+T PGS using genome-wide significant SNPs (p < 5×10^−8^) we also constructed a PGS based on the genome-wide significant SNPs and their weights (*b*_*J*_) estimated from a conditional/joint COJO analysis (that allows multiple SNPs within an LD block to be selected if they show association additional to the lead SNP).^12^ For benchmarking comparisons, we also calculated PGS in the 20 cohorts new in this PGC MDD wave 3 using SBayesR, SBayesRC and COJO derived from GWAS summary statistics from previously published PGC MDD GWAS studies in 2018 (N_case_=170,756, N_cont_ =329,443) by Wray^7^ and 2019 (PGC2+UKB + 23andMe-1stwave, N_case_=246,363, N_cont_=561,190) by Howard,^2^ omitting the individual-level genotyped studies from PGC MDD2.

The PGS were evaluated in each cohort. Logistic regression of case/control status on PGS standardised in each cohort so that the controls had PGS with mean zero and standard deviation of 1. Genetic principal components were included as covariates, but these explained very little variation. The performance of prediction in each target cohort was quantified by the following metrics:

- p-value of the PGS regression coefficient,
- Area under the receiver operating characteristic curve (AUC),
- Nagelkerke’s *R*^*2*^ (a pseudo-*R*^*2*^ statistic that depends on the proportion of cases in the sample),
- Variance explained by the PGS on the liability scale, $r_{l}^{2}$, derived from the transformation of the variance explained in a linear regression model,^72^ calculated assuming a population lifetime risk of MDD as 15%,
- OR of 10th PGS decile relative to the first decile,
- OR of 100^th^ centile relative to the 1^st^ centile

Results are reported per cohort and for the weighted mean across cohorts (weighted by effective sample size, *N*_*eff*_). The results reported in the main text come from the joint analysis of 48 cohorts combined based on their within-cohort standardised PGS; the estimated variance explained in liability from the joint analysis was the same as the weighted average of the estimates from individual cohorts. In addition to the evaluation statistics generated for individual cohorts, the joint analysis also allowed evaluation of OR per PGS centiles and their 95% confidence intervals.

#### PGS association in participants with non-European ancestry

We conducted polygenic profiling in three cohorts of African ancestry (48,669 cases and 52,939 controls), two cohorts of Latinx (AMR) ethnicity (1,202 cases and 5,112 controls), three cohorts of East Asian ancestry (6,902 cases and 75,879 controls), and three cohorts of South Asian ancestry (4,862 cases and 28,965 controls) and compared them to the 43 European cohorts. We included studies from China, the USA and the UK that had over 200 cases. Case status was defined based on symptom questionnaires, healthcare records or a combination of both (Table S7). The summary statistics used as a training set excluded 23andMe (because of data sharing restrictions). The available SNP set was limited to those with MAF > 0.10 and imputation INFO score > 0.9, and ambiguous SNPs were excluded. Further, we retained only SNPs with imputation INFO score > 0.9 in the target data sets.

As above, PGS were calculated from the all-ancestries meta-analysis using the p-value P+CT method implemented in PRSice v2^43^ For C+T, the LD estimation was based on the 1000 Genomes Project phase 3 samples (*N*=503) with European ancestry to match the discovery sample. We fixed the LD *r*^*2*^ threshold at 0.1 and we assessed p-value thresholds (1, 0.5, 0.1, 0.05, 0.005, 5e-3, 5e-4, 5e-5, 5e-6, 5e-7, 5e-8), reporting results for the optimal threshold in each data set. The $r_{l}^{2}$ was calculated on the liability scale, using an MDD lifetime prevalence estimate of 0.15, by taking the difference of the R^2^_liability_ of the regression of PGS on the case status with the first ten PCs as covariates, and the R^2^_liability_ of the null model including the first 10 PCs alone. The confidence intervals for the $r_{l}^{2}$ were calculated using bootstrapping with 100 iterations.

#### Tissue and cell-type enrichment analysis

We performed tissue and cell-type enrichment analysis aiming to identify relevant tissues and cell types underlying MDD. First, we analyzed GTEx gene expression data (v8) in 27 human tissues after excluding: 1) tissues with less than 100 donors, 2) non-natural tissues (such as cell lines), and 3) testis tissues (expression outlier).^73^ Second, for the cell-type analysis, we used single-cell RNA sequencing data with over 160K high-quality cells sampled from 19 regions in the entire mouse central nervous system and peripheral nervous system.^17^ We analyzed these data at the cell-type level, including 39 broad cell types (level 4) and 251 refined cell types (level 5, after filtering cell types with fewer than 20 cells). We considered only protein-coding genes with 1:1 orthology between human and mouse for the calculation of expression specificity. Third, we further evaluated the heritability enrichment using single-nucleus RNA sequencing data of over 3 million high-quality nuclei from around 100 dissections across adult human brain.^18^ We analyzed this dataset at the cell-type level including 31 superclusters and 461 cell clusters. For all expression datasets, we calculated a metric of gene expression specificity as previously described^16^; it measures, for each gene, its expression in a specific tissue or cell-type relative to its total expression across all tissues or cell types. As in previous studies,^16,74^ we utilized the genes with the top 10% specificity values in each tissue or cell-type for the enrichment analyses.

We used two primary methods, partitioned LD Score regression (pLSDC)^75^ and MAGMA (v1.08),^45^ to test the enrichment of tissues and cell types in the MDD GWAS results. Our analyses using pLDSC evaluated if the SNPs within 100kb regions of the top 10% specifically expressed genes were enriched for SNP-based heritability. For each tissue or cell-type, we computed the LD scores for this cell-type-specific annotation and added it to the baseline model of 53 functional annotations. We assessed the enrichment of tissue or cell-types using the coefficient z-scores and computed one-sided p-values. For the analyses using MAGMA, we tested if the top 10% specifically expressed genes in each tissue or cell-type were the most associated genes from the GWAS. As part of this analysis, we filtered SNPs with MAF <1% or with imputation INFO score < 0.9, and mapped SNPs to genes with 35kb upstream and 10kb downstream windows. We first conducted gene-level association tests and then gene-set analyses for the tissue or cell-type specifically expressed genes. For both methods, we used the European samples in the phase 3 of 1000 Genomes Project as the reference panel and reported significance at the 5% false discovery rate within each dataset and method.

#### Gene finding analysis in fastBAT

A gene-based association analysis was conducted using fastBAT^76^ within GCTA version 1.94.0 beta.^46^ After removing SNPs with MAF < 0.01 or with imputation INFO score < 0.8, there were 6,724,173 SNPs remaining for analysis. The European subsample from phase 3 of the 1000 Genomes Project was used as the LD reference panel with the fastBAT default LD cut-off of 0.9 applied. A gene list consisting of 19,878 protein coding genes available from https://figshare.com/articles/dataset/geneMatrix/13335548 was used to map the base pair position of genes using genome build hg19. A total of 18,747 genes were analyzed for association with MDD. A Bonferroni correction (0.05/18,747) was applied, with a p-value < 2.67 ×10^−6^ required for an association with MDD.

##### Fine mapping of SNPs

We identified putative causal variants for MD using PolyFun v1.0.0^47^ and SuSiE v0.11.92.^48^ We restricted the meta-analyzed summary statistics to variants lying outside of the HLA region, with imputation INFO score > 0.6 and MAF > 0.001. We computed prior causal probabilities based on 187 functional enrichments from the baselineLF2.2 model,^47^ using an L2-regularized extension of stratified LD Score regression (S-LDSC) implemented in PolyFun, using LD scores derived from the UK Biobank and provided with PolyFun. We performed fine-mapping of genome-wide significant loci with windows defined by clumping (as described above). We used SuSiE to perform the fine-mapping, assuming a single causal variant in each case (as single-variant fine-mapping does not require population-accurate estimation of LD^47^). We identified variants of interest as having a posterior inclusion probability in the causal set (PIP) > 0.95. We ranked all variants by PIP and defined 95% credible causal sets of variants as the minimum set of variants whose PIPs summed to >=0.95. We mapped credible causal sets to 19,878 protein coding genes. We defined high-confidence genes as those containing all variants within the credible causal set within the gene body, and additionally listed all genes at least partially overlapping the credible causal set. We performed analyses on the full, multiple ancestry meta-analysis, and additionally on the European ancestry subset. Estimates of LD (for clumping and for S-LDSC) used European ancestry reference panels; as such, multiple ancestry analyses should be considered exploratory.

#### Expression-based mapping of SNPs

##### Overview

We used both Transcriptome-wide Association Study (TWAS)^49^ and Summary-based Mendelian Randomisation (SMR)^51^ methods to infer differential gene expression associated with MDD based on the meta-analyzed summary statistics. Both methods test whether genetic variants associated with MDD are also associated with differential expression of nearby genes. TWAS and SMR have different limitations and are therefore complementary. TWAS considers the effect of multiple variants on gene expression and the GWAS phenotype, thereby increasing statistical power to detect associations, whereas SMR only considers the effect of each variant individually. However, TWAS requires multi-variant models predicting gene expression to have been generated in the genotype-expression dataset, which are not available in some cases. In contrast, SMR requires only expression quantitative trait (eQTL) summary statistics, enabling it to use a wider range of genotype-expression datasets, such as eQTL meta-analysis results from eQTLGen^77^ and MetaBrain^78^ consortia. For TWAS and SMR analysis, the European subset of the 1000 Genomes Project, Phase 3 was used as an LD reference.

##### TWAS

TWAS was performed based on a previous MDD TWAS,^79^ using FUSION software with default settings. All gene expression panels were of European ancestry. Gene expression panels relating to the brain include dorsolateral prefrontal cortex (DLPFC) from PsychENCODE, differential expression and splicing in DLPFC from the CommonMind Consortium (CMC) and the 12 brain regions collected in the Genotype-Expression (GTEx) project. We also included panels capturing expression in pituitary, adrenal and thyroid tissues from GTEx, given prior evidence these tissues play a role in MDD.^80,81^ Finally, we included panels capturing gene expression in blood from GTEx, the Netherlands Twin Registry (NTR) and the Young Finns Study (YFS) due to their increased sample size, the moderate correlation between cis-eQTLs across tissues,^82^ and evidence that altered expression in blood could influence risk of MDD.^83,84^ To distinguish associations for a gene captured by multiple panels, we refer to each panel-gene pair as *features*.

To account for multiple testing of genes across panels, we used the transcriptome-wide significance threshold previously estimated using a permutation procedure.^79^ The threshold for transcriptome-wide significance was p = 1.37×10^−6^. A more stringent significance threshold (α = .001; p= 3.69×10^−8^) was applied to distinguish high-confidence associations.

Colocalization of overlapping GWAS and gene expression associations was assessed using *coloc*^50^ as implemented by FUSION. *coloc* is a Bayesian method that estimates the posterior probability that associations within a locus for two outcomes are driven by a shared causal variant (PP4).

Conditional analysis was performed using FUSION to determine whether associations within each locus were independent. FUSION also estimates the proportion of the GWAS association explained by the predicted expression of all features in the locus. Furthermore, TWAS-based fine mapping was carried out using FOCUS^85^ to help identify which features were most likely causal for the association. FOCUS estimates the posterior inclusion probability (PIP) of each feature being causal within a region of association, using the sum of PIPs to define the default 90% credible set, a set of features likely to contain the causal feature.

##### SMR

SMR was run using eQTL meta-analysis summary statistics from European populations for blood from eQTLGen,^77^ and five nervous system tissues from MetaBrain (Basalganglia, Cerebellum, Cortex, Hippocampus and Spinal Cord).^78^ SMR was run using default settings. The HEIDI test is performed alongside SMR to test for effect size heterogeneity between the GWAS and eQTL summary statistics, which would indicate that they are driven by different causal variants. The HEIDI test is a frequentist approach that is analogous to colocalization used to check for shared causal variants underlying TWAS associations.

##### Inferring altered dorsolateral prefrontal cortex protein levels in MDD

TWAS and SMR methods can also be applied to protein quantitative trait loci (pQTL) datasets, inferring whether genetic variation associated with MDD confer altered protein levels. Recently, pQTL data from the dorsolateral prefrontal cortex (DLPFC) has been prepared to perform proteome-wide association study (PWAS),^86^ using genotype-protein data from two datasets, referred to as ROSMAP and Banner et al. We followed the same procedure as the study originally performing PWAS, which included performing PWAS using both ROSMAP and Banner et al. panels, treating the larger ROSMAP panel as the discovery sample, and the Banner et al. panel as a replication sample. Proteins were identified as statistically significant in ROSMAP if pFDR < 0.05 (correcting for all proteins tested) and considered replicated in Banner et al. if pFDR < 0.05 (correcting for number of proteins tested for replication). PWAS was performed using FUSION software with the in-built downstream colocalization analysis using *coloc* (PP4 > 0.8). As in the original PWAS, we used the HEIDI test within SMR to confirm evidence of colocalization based on the ROSMAP dataset (HEIDI *p* > 0.05).

##### Gene associations based on mapping chromatin profiles of brain tissues and cells

The gene-based association study for brain tissue-derived chromatin profiles for four tissues (fetal brain paracentral cortex; adult brain – DLPFC), and cells (astrocytes and neurons - human iPSCs) was performed using H-MAGMA v.1.08.^52,87^ Complementary to TWAS and the *coloc* approaches, H-MAGMA maps functional and regulatory effects of non-coding SNPs based on three-dimensional chromatin data. We tested all four tissue profiles integrated with GWAS association statistics using European genetic-ancestry reference panels. Bonferroni correction (0.05/number of associations = 3.73× 10^−6^) on unique genes and four tissues was applied to define significant associations. The genes were grouped into 1Mb regions based on hg19 position; if many genes were within close proximity to each other, then the region size was bigger than 1Mb (e.g. for the MHC region).

##### Psychiatric Omnilocus Prioritization Score (PsyOPS)

PsyOPS combines multiple methods to identify genes that are likely to be causally implicated in psychiatric disorders.^53^ The method prioritised genes based on three criteria: mutational constraint (gnomAD probability of loss-of-function intolerance [pLI] > 0.99), brain expression (Human Protein Atlas “elevated in brain” designation), and association with neurodevelopmental disorders (from Genomics England gene panels for autism, intellectual disability, and epilepsy). The PsyOPS score is assigned based on prediction from a trained classification model. For each lead variant we selected the gene with the highest PsyOPS score, using nearest gene to break ties.

##### Defining high-confidence genes across all gene-based analysis methods

We define genes as showing a high confidence association with MDD based on the following three criteria from fine mapping, TWAS and PWAS analysis:

- SNP-based fine-mapping: all variants within the 95% credible causal set within the gene body
- TWAS: genes showing strong evidence of association in TWAS based on any gene expression panel (p < 3.69×10^−8^, α=0.001), strong evidence of colocalisation (*coloc* PP4 > 0.8), and being congruent with causal model based on TWAS-based fine-mapping (FOCUS PIP > 0.5)
- PWAS: protein was statistically significant based on both ROSMAP and Banner et al. protein panels, and showed strong evidence of colocalisation based on ROSMAP panel using both *coloc* (PP4 >0.8) and HEIDI (p > 0.05)

Results from the SMR analysis of eQTL data were considered auxiliary as no fine-mapping approaches available. SMR results for high-confidence genes are provided for additional information.

Other gene finding analyses based on the proximity of genes to MDD associated variation are often confounded by LD (linking lead variants to the nearest gene (within 50kb), fastBAT and H-MAGMA), leading to multiple genes within a given locus to be associated. These methods are therefore considered auxiliary, and do not provide sufficient evidence to define high confidence associations. Furthermore, the PsyOPS gene prioritization approach was considered auxiliary given it also links associated variants to genes based on proximity, in addition to other information). Gene lists from auxiliary gene finding analyses were generated using the following criteria:

- Nearest Gene: nearest gene to lead variant in genome-wide significant loci within 50kb.
- fastBAT: gene association p < 2.67 × 10^−6^ (0.05/18,747)
- H-MAGMA: gene association 3.73× 10^−6^, controlling for all gene-panel tests
- PsyOPS: gene with highest PsyOPS score for each lead variant in genome-wide significant loci

##### Gene set enrichment analysis: Synaptic Gene Ontologies (SynGO)

We conducted gene set enrichment tests of Gene Ontology (GO)^88,89^terms for biological processes, molecular mechanisms, and cellular components that have been expertly curated for synapse function using SynGO.^15^ We input genes from the high-confidence gene list (identified with finemapping, TWAS, or PWAS) into the SynGO Portal <https://syngoportal.org/> with “brain expressed” genes as the background set.

##### Drug enrichment analysis

Drug-gene sets were created using Drug Targetor,^19^ which collates information across a range of drug-gene databases, including ChEMBL, PHAROS, PDSP K_i_ database and NCBI PubChem BioAssay. We grouped drug-gene sets into two hierarchical levels based on the Anatomical Therapeutic Chemical (ATC) Classification System: pharmacological subgroup (3rd level) and chemical subgroup (4th level).

Briefly, the Drug Targetor method,^19^ was used to assess the association of individual drug or small molecule related gene sets and to assess drug class enrichment. Gene sets are given in accompanying supplementary files (see key resources table). Analyses were run using MAGMA v1.10^45^ using gene flanks of −35kb 5′ and +10kb 3′.^90^ Drug class enrichment was calculated using area under the curve (AUC) defined by the % of drug class gene-sets vs their rank in all the gene-sets.^91^

### ADDITIONAL INFORMATION

Any additional information required to reanalyze the data reported in this paper is available from the lead contact upon request.

## Supplementary Material

1

2 Figure S1. Path diagram showing genetic loadings of MDD phenotype definitions on a latent MDD factor, related to the STAR Methods
Clin, clinical; EHR, electronic health record; Quest, questionnaire; SelfRep, self-report of MDD diagnosis. Numbers represent standardised loadings with standard errors in brackets from genomic structural equation model, with the loading of the Clinical MDD phenotype constrained to 1.0. Self-directed arrows indicate variance of the MDD factor or residual variances of the MDD phenotypes (“u”).

3

4

5

6

7

8

9

10

11

## Figures and Tables

**Table 1.** Details of diverse ancestry studies included in the current GWAS

Ancestry group	*N* studies	*N* cases	*N* controls	*N*_eff_/2

European	76	525,197	3,362,335	788,603
East Asian	7	18,709	349,619	30,654
South Asian	1	3,748	25,934	6,549
African	8	9,649	122,347	17,077
Hispanic/Latin American	5	22,927	340,403	41,233
Multiple/mixed	12	108,578	163,587	120,342
All ancestries	109	688,808	4,364,225	1,004,459

**Table 2.** Significant drug target enrichments

ATC class	Drug name	# of genes	Q value	Notes

L01AC03	CARBOQUONE	7	1.16 × 10^−4^	cancer compound
G03XC03	LASOFOXIFENE	2	5.64 × 10^−4^	osteoporosis treatment; oestrogen receptor modulator
L02AA04	FOSFESTROL	2	5.64 × 10^−4^	cancer causing and block synthesis of testosterone
G03GB01	CYCLOFENIL	4	1.39 × 10^−3^	gonadal stimulant
N03AX16	PREGABALIN	27	1.40 × 10^−3^	neuropathic pain, epilepsy, generalized anxiety disorder
N06AX19	GEPIRONE	2	5.19 × 10^−3^	antidepressant, not marketed
D11AX06	MEQUINOL	4	6.41 × 10^−3^	pigmental drug
N05AX16	BREXPIPRAZOLE	5	6.74 × 10^−3^	antipsychotic, antidepressant
N05AB08	THIOPROPERAZINE	2	0.0131	antipsychotic
N05AC04	PIPOTIAZINE	2	0.0131	antipsychotic
M05BX01	IPRIFLAVONE	14	0.0238	osteoporosis treatment
N06BA13, N06BA07	MODAFINIL	2	0.0337	narcolepsy treatment
D07AB08, S01BA11	DESONIDE	4	0.0337	anti-inflammatory
J01XX08	LINEZOLID	3	0.0337	antibiotic
N05AD04	MOPERONE	3	0.0419	antipsychotic
N05AX15	CARIPRAZINE	6	0.0493	antipsychotic

Table shows the top 16 most significantly enriched drugs based on capture of their targets within the gene-based associations of the current MD GWAS analysis. One topical preparation is not shown. The test for drug enrichment is not directional and may indicate compounds that confer risk of MD or exacerbate depressive symptoms, as well as those that ameliorate risk or depressive symptoms. Q value is false discovery rate, Benjamini-Yekutieli corrected (competitive analysis).

**KEY RESOURCES TABLE:** 

REAGENT or RESOURCE	SOURCE	IDENTIFIER

Deposited data		

Genome-wide summary statistics (exc. 23andMe)	This paper	https://doi.org/10.6084/m9.figshare.27061255
Genome-wide summary statistics (inc. 23andMe)	Psychiatric Genomics Consortium Data Access Committee	mdd23am
Study-level genotypes	Psychiatric Genomics Consortium Data Access Committee	mddw3v01
Study-level summary statistics	Psychiatric Genomics Consortium Data Access Committee	mddw3sum
fastBAT gene association tests	This paper	https://doi.org/10.6084/m9.figshare.27089614
multi-SNP-based conditional and joint (COJO) associations	This paper	https://doi.org/10.6084/m9.figshare.27089614
Hi-C chromatin mapping	This paper	https://doi.org/10.6084/m9.figshare.27089614
LDSC genetic correlation estimates	This paper	https://doi.org/10.6084/m9.figshare.27089614
Principal components plots for genotyped studies	This paper.	https://doi.org/10.6084/m9.figshare.27089614
DrugTargetor enrichment tests	This paper	https://doi.org/10.6084/m9.figshare.27089614
Single cell enrichment results	This paper	https://doi.org/10.6084/m9.figshare.27089614
Polygenic risk score results	This paper	https://doi.org/10.6084/m9.figshare.27089614
Genome-Wide Association Study of Schizophrenia	dbGaP	phs000021
Genome-Wide Association Study of Parkinson Disease	dbGaP	phs000196
High Density SNP Association Analysis of Melanoma	dbGaP	phs000187
Haplotype Reference Consortium v1.1	European Genome-Phenome Archive	EGAD00001002729
GTEx v8	GTEx Portal	https://gtexportal.org
Mouse Brain Atlas	Zeisel et al.^17^	http://mousebrain.org
Human Brain Cell Atlas v1.0	Siletti et al.^18^	https://cellxgene.cziscience.com/collections/283d65eb-dd53-496d-adb7-7570c7caa443
eQTLGen	eQTLGen Consortium	https://www.eqtlgen.org
MetaBrain	MetaBrain	https://www.metabrain.nl
Brain pQTL (ROSMAP, Banner)	AD Knowledge Portal	https://doi.org/10.7303/syn24172458
SynGO	SynGO Portal	https://syngoportal.org/

Software and algorithms		

Analysis workflows	This study	https://doi.org/10.5281/zenodo.11935052
Ricopili 2019_Jun_18.001	Lam et al.^41^	https://doi.org/10.1093/bioinformatics/btz633
Genomic SEM v0.0.5c	Grotzinger et al.^13^	https://github.com/GenomicSEM/GenomicSEM
GCTB 2.0	Zeng et al.^14^	https://cnsgenomics.com/software/gctb/
DENTIST	Chen et al.^42^	https://github.com/Yves-CHEN/DENTIST
PRSice v2	Choi and O’Reilly^43^	https://choishingwan.github.io/PRSice/
LDScore v1.0.1	Bulik-Sullivan et al.^44^	https://github.com/bulik/ldsc
MAGMA v1.08	de Leeuw et al.^45^	https://cncr.nl/research/magma/
GCTA version 1.94.0 beta	Yang et al.^46^	https://yanglab.westlake.edu.cn/software/gcta/
PolyFun v1.0.0	Weissbrod et al.^47^	https://github.com/omerwe/polyfun
SuSiE v0.11.92	Wang et al.^48^	https://stephenslab.github.io/susieR/
FUSION	Gusev et al.^49^	http://gusevlab.org/projects/fusion/
*coloc v5.1*	Giambartolomei et al.^50^	https://github.com/chr1swallace/coloc
smr version 1.3.1	Zhu et al.^51^	https://yanglab.westlake.edu.cn/software/smr/
H-MAGMA v.1.08	Sey et al.^52^	https://github.com/thewonlab/H-MAGMA
PsyOPS	Wainberg et al.^53^	https://github.com/Wainberg/PsyOPS
Drug Targetor	Gaspar et al.^19^	https://drugtargetor.com

## Highlights

- Trans-ancestry GWAS identified 697 variants and 308 genes associated with depression
- Implicates postsynaptic density, neuronal dysregulation, and amygdala involvement
- Findings enriched for antidepressant targets and highlight drug repurposing options
- Polygenic scores predicted depression case-control status across all ancestries

## CONSORTIA

The members of the Major Depressive Disorder Working Group of the Psychiatric Genomics Consortium are Mark J. Adams, Fabian Streit, Xiangrui Meng, Swapnil Awasthi, Brett N. Adey, Karmel W. Choi, V. Kartik Chundru, Jonathan R.I. Coleman, Bart Ferwerda, Jerome C. Foo, Zachary F. Gerring, Olga Giannakopoulou, Priya Gupta, Alisha S.M. Hall, Arvid Harder, David M. Howard, Christopher Hübel, Alex S.F. Kwong, Daniel F. Levey, Brittany L. Mitchell, Guiyan Ni, Vanessa K. Ota, Oliver Pain, Gita A. Pathak, Eva C. Schulte, Xueyi Shen, Jackson G. Thorp, Alicia Walker, Shuyang Yao, Jian Zeng, Johan Zvrskovec, Dag Aarsland, Ky’Era V. Actkins, Mazda Adli, Esben Agerbo, Mareike Aichholzer, Allison Aiello, Tracy M. Air, Thomas D. Als, Evelyn Andersson, Till F.M. Andlauer, Volker Arolt, Helga Ask, Julia Bäckman, Sunita Badola, Clive Ballard, Karina Banasik, Nicholas J. Bass, Aartjan T.F. Beekman, Sintia Belangero, Tim B. Bigdeli, Elisabeth B. Binder, Ottar Bjerkeset, Gyda Bjornsdottir, Sigrid Børte, Emma Bränn, Alice Braun, Thorsten Brodersen, Tanja M. Brückl, Søren Brunak, Mie T. Bruun, Margit Burmeister, Pichit Buspavanich, Jonas Bybjerg-Grauholm, Enda M. Byrne, Jianwen Cai, Archie Campbell, Megan L. Campbell, Adrian I. Campos, Enrique Castelao, Jorge Cervilla, Boris Chaumette, Chia-Yen Chen, Hsi-Chung Chen, Zhengming Chen, Sven Cichon, Lucía Colodro-Conde, Anne Corbett, Elizabeth C. Corfield, Baptiste Couvy-Duchesne, Nick Craddock, Udo Dannlowski, Gail Davies, E.J.C. de Geus, Ian J. Deary, Franziska Degenhardt, Abbas Dehghan, J. Raymond DePaulo, Michael Deuschle, Maria Didriksen, Khoa Manh Dinh, Nese Direk, Srdjan Djurovic, Anna R. Docherty, Katharina Domschke, Joseph Dowsett, Ole Kristian Drange, Erin C. Dunn, William Eaton, Gudmundur Einarsson, Thalia C. Eley, Samar S.M. Elsheikh, Jan Engelmann, Michael E. Benros, Christian Erikstrup, Valentina Escott-Price, Chiara Fabbri, Yu Fang, Sarah Finer, Josef Frank, Robert C. Free, Linda Gallo, He Gao, Michael Gill, Maria Gilles, Fernando S. Goes, Scott Douglas Gordon, Jakob Grove, Daniel F. Gudbjartsson, Blanca Gutierrez, Tim Hahn, Lynsey S. Hall, Thomas F. Hansen, Magnus Haraldsson, Catharina A. Hartman, Alexandra Havdahl, Caroline Hayward, Stefanie Heilmann-Heimbach, Stefan Herms, Ian B. Hickie, Henrik Hjalgrim, Jens Hjerling-Leffler, Per Hoffmann, Georg Homuth, Carsten Horn, Jouke-Jan Hottenga, David M. Hougaard, Iiris Hovatta, Qin Qin Huang, Donald Hucks, Floris Huider, Karen A. Hunt, Nicholas S. Ialongo, Marcus Ising, Erkki Isometsä, Rick Jansen, Yunxuan Jiang, Ian Jones, Lisa A. Jones, Lina Jonsson, Masahiro Kanai, Robert Karlsson, Siegfried Kasper, Kenneth S. Kendler, Ronald C. Kessler, Stefan Kloiber, James A. Knowles, Nastassja Koen, Julia Kraft, Henry R. Kranzler, Kristi Krebs, Theodora Kunovac Kallak, Zoltán Kutalik, Elisa Lahtela, Marilyn Lake, Margit Hørup Larsen, Eric J. Lenze, Melissa Lewins, Glyn Lewis, Liming Li, Bochao Danae Lin, Kuang Lin, Penelope A. Lind, Yu-Li Liu, Donald J. MacIntyre, Dean F. MacKinnon, Brion S. Maher, Wolfgang Maier, Victoria S. Marshe, Gabriela A. Martinez-Levy, Koichi Matsuda, Hamdi Mbarek, Peter McGuffin, Sarah E. Medland, Susanne Meinert, Christina Mikkelsen, Susan Mikkelsen, Yuri Milaneschi, Iona Y. Millwood, Esther Molina, Francis M. Mondimore, Preben Bo Mortensen, Benoit H. Mulsant, Joonas Naamanka, Jake M. Najman, Matthias Nauck, Igor Nenadić, Kasper R. Nielsen, Ilja M. Nolt, Merete Nordentoft, Markus M. Nöthen, Mette Nyegaard, Michael C. O’Donovan, Asmundur Oddsson, Adrielle M. Oliveira, Catherine M. Olsen, Hogni Oskarsson, Sisse Rye Ostrowski, Michael J. Owen, Richard Packer, Teemu Palviainen, Pedro M. Pan, Carlos N. Pato, Michele T. Pato, Nancy L. Pedersen, Ole Birger Pedersen, Wouter J. Peyrot, James B. Potash, Martin Preisig, Michael H. Preuss, Jorge A. Quiroz, Miguel E. Renteria, Charles F. Reynolds III, John P. Rice, Saori Sakaue, Marcos L. Santoro, Robert A. Schoevers, Andrew Schork, Thomas G. Schulze, Tabea S. Send, Jianxin Shi, Engilbert Sigurdsson, Kritika Singh, Grant C.B. Sinnamon, Lea Sirignano, Olav B. Smeland, Daniel J. Smith, Tamar Sofer, Erik Sørensen, Sundararajan Srinivasan, Hreinn Stefansson, Kari Stefansson, Peter Straub, Mei-Hsin Su, André Tadic, Henning Teismann, Alexander Teumer, Anita Thapar, Pippa A. Thomson, Lise Wegner Thørner, Apostolia Topaloudi, Shih-Jen Tsai, Ioanna Tzoulaki, George Uhl, André G. Uitterlinden, Henrik Ullum, Daniel Umbricht, Robert J. Ursano, Sandra Van der Auwera, Albert M. van Hemert, Abirami Veluchamy, Alexander Viktorin, Henry Völzke, G. Bragi Walters, Xiaotong Wang, Agaz Wani, Myrna M. Weissman, Jürgen Wellmann, David C. Whiteman, Derek Wildman, Gonneke Willemsen, Alexander T. Williams, Bendik S. Winsvold, Stephanie H. Witt, Ying Xiong, Lea Zillich, John-Anker Zwart, 23andMe Research Team, China Kadoorie Biobank, Collaborative Group, Estonian Biobank Research Team, Genes & Health Research Team, HUNT All-In Psychiatry, The BioBank Japan Project, VA Million Veteran Program, Ole A. Andreassen, Bernhard T. Baune, Klaus Berger, Dorret I. Boomsma, Anders D. Børglum, Gerome Breen, Na Cai, Hilary Coon, William E. Copeland, Byron Creese, Carlos S. Cruz-Fuentes, Darina Czamara, Lea K. Davis, Eske M. Derks, Enrico Domenici, Paul Elliott, Andreas J. Forstner, Micha Gawlik, Joel Gelernter, Hans J. Grabe, Steven P. Hamilton, Kristian Hveem, Catherine John, Jaakko Kaprio, Tilo Kircher, Marie-Odile Krebs, Po-Hsiu Kuo, Mikael Landé n, Kelli Lehto, Douglas F. Levinson, Qingqin S. Li, Klaus Lieb, Ruth J.F. Loos, Yi Lu, Susanne Lucae, Jurjen J. Luykx, Hermine H.M. Maes, Patrik K. Magnusson, Hilary C. Martin, Nicholas G. Martin, Andrew McQuillin, Christel M. Middeldorp, Lili Milani, Ole Mors, Daniel J. Müller, Bertram Müller-Myhsok, Yukinori Okada, Albertine J. Oldehinkel, Sara A. Paciga, Colin N.A. Palmer, Peristera Paschou, Brenda W.J.H. Penninx, Roy H. Perlis, Roseann E. Peterson, Giorgio Pistis, Renato Polimanti, David J. Porteous, Danielle Posthuma, Jill A. Rabinowitz, Ted Reichborn-Kjennerud, Andreas Reif, Frances Rice, Roland Ricken, Marcella Rietschel, Margarita Rivera, Christian Rück, Giovanni A. Salum, Catherine Schaefer, Srijan Sen, Alessandro Serretti, Alkistis Skalkidou, Jordan W. Smoller, Dan J. Stein, Frederike Stein, Murray B. Stein, Patrick F. Sullivan, Martin Tesli, Thorgeir E. Thorgeirsson, Henning Tiemeier, Nicholas J. Timpson, Monica Uddin, Rudolf Uher, David A. van Heel, Karin J.H. Verweij, Robin G. Walters, Sylvia Wassertheil-Smoller, Jens R. Wendland, Thomas Werge, Aeilko H. Zwinderman, Karoline Kuchenbaecker, Naomi R. Wray, Stephan Ripke, Cathryn M. Lewis, and Andrew M. McIntosh. See supplemental information for consortium member affiliations.
