# Supplementary material for: Trans-ancestry genome-wide study of depression identifies 697 associations implicating cell types and pharmacotherapies
Source: Cell. Author manuscript; Available in PMC 2025 Feb 15. (PMC11829167; doi:10.1016/j.cell.2024.12.002)
Supplement: 1 [file NIHMS2048064-supplement-1.pdf]

Supplemental consortium authorship information

Mark J Adams* 1, Fabian Streit* 2, 3, 4, 5, Xiangrui Meng* 6, Swapnil Awasthi* 7, Brett N Adey 8, Karmel W Choi 9, 10, V Kartik Chundru 11, 12, Jonathan RI Coleman 8, 13, Bart Ferwerda 14, Jerome C Foo 2, 15, 16, 17, Zachary F Gerring 18, Olga Giannakopoulou 6, Priya Gupta 19, 20, Alisha S M Hall 2, 21, Arvid Harder 22, David M Howard 8, Christopher Hübel 8, 23, 24, Alex S F Kwong 1, 25, Daniel F Levey 19, 20, Brittany L Mitchell 18, 26, 27, 28, Guiyan Ni 29, Vanessa K Ota 30, Oliver Pain 31, Gita A Pathak 19, 32, Eva C Schulte 33, 34, 35, 36, 37, Xueyi Shen 1, Jackson G Thorp 18, Alicia Walker 29, Shuyang Yao 22, Jian Zeng 29, Johan Zvrskovec 8, 13, Dag Aarsland 38, Ky'Era V Actkins 39, Mazda Adli 40, 41, Esben Agerbo 24, 42, 43, Mareike Aichholzer 44, Allison Aiello 45, Tracy M Air 46, Thomas D Als 43, 47, 48, Evelyn Andersson 49, Till F M Andlauer 50, 51, Volker Arolt 52, Helga Ask 53, 54, Julia Bäckman 49, Sunita Badola 55, Clive Ballard 56, Karina Banasik 57, Nicholas J Bass 6, Aartjan T F Beekman 58, Sintia Belangero 30, 59, Tim B Bigdeli 60, Elisabeth B Binder 50, 61, 62, Ottar Bjerkeset 63, 64, Gyda Bjornsdottir 65, Sigrid Børte 66, 67, 68, Emma Bränn 69, Alice Braun 7, Thorsten Brodersen 70, Tanja M Brückl 71, Søren Brunak 57, Mie T Bruun 72, Margit Burmeister 73, Pichit Buspavanich 74, 75, Jonas Bybjerg-Grauholm 76, 77, Enda M Byrne 78, Jianwen Cai 79, Archie Campbell 80, 81, Megan L Campbell 82, Adrian I Campos 83, Enrique Castelao 84, Jorge Cervilla 85, 86, 87 Boris Chaumette 88, Chia-Yen Chen 89, Hsi-Chung Chen 90, 91, Zhengming Chen 92, Sven Cichon 93, 94, 95, 96, Lucía Colodro-Conde 18, 97, Anne Corbett 56, Elizabeth C Corfield 53, 98, Baptiste Couvy-Duchesne 99, Nick Craddock 100, Udo Dannlowski 52, Gail Davies 101, EJC de Geus 102, Ian J Deary 101, Franziska Degenhardt 94, 103, Abbas Dehghan 104, 105, J Raymond DePaulo 106, Michael Deuschle 5, 107, Maria Didriksen 108, Khoa Manh Dinh 109, Nese Direk 110, Srdjan Djurovic 111, 112, Anna R Docherty 113, 114, 115, Katharina Domschke 116, Joseph Dowsett 108, Ole Kristian Drange 63, 117, 118, 119, Erin C Dunn 10, 120, 121, William Eaton 122, Gudmundur Einarsson 65, Thalia C Eley 8, Samar S M Elsheikh 123, Jan Engelmann 124, Michael E Benros 77, 125, 126, Christian Erikstrup 109, Valentina Escott-Price 100, Chiara Fabbri 8, 127, Yu Fang 73, Sarah Finer 128, Josef Frank 2, Robert C Free 129, Linda Gallo 130, He Gao 131, Michael Gill 132, Maria Gilles 5, 107, Fernando S Goes 106, Scott Douglas Gordon 18, 26, Jakob Grove 43, 47, 48, 133, Daniel F Gudbjartsson 65, 134, Blanca Gutierrez 85, 86, 87, Tim Hahn 52, Lynsey S Hall 1, 100, Thomas F Hansen 57, 135, 136, Magnus Haraldsson 137, 138, Catharina A Hartman 139, Alexandra Havdahl 53, 140, Caroline Hayward 141, Stefanie Heilmann-Heimbach 94, Stefan Herms 93, 94, Ian B Hickie 142, Henrik Hjalgrim 143, Jens Hjerling-Leffler 144, Per Hoffmann 93, 94, Georg Homuth 145, Carsten Horn 146, Jouke-Jan Hottenga 102, David M Hougaard 76, 77, Iiris Hovatta 147, Qin Qin Huang 12, Donald Hucks 39, Floris Huider 102, Karen A Hunt 148, Nicholas S Ialongo 122, Marcus Ising 149, Erkki Isometsä 150, Rick Jansen 58, Yunxuan Jiang 151, Ian Jones 100, Lisa A Jones 152, Lina Jonsson 153, Masahiro Kanai 154, 155, 156, Robert Karlsson 22, Siegfried Kasper 157, Kenneth S Kendler 158, Ronald C Kessler 159, Stefan Kloiber 123, 149, 160, 161, James A Knowles 162, Nastassja Koen

82, Julia Kraft 7, Henry R Kranzler 163, 164, Kristi Krebs 165, Theodora Kunovac Kallak 166, Zoltán Kutalik 167, 168, 169, Elisa Lahtela 170, Marilyn Lake 171, Margit Hørup Larsen 108, Eric J Lenze 172, Melissa Lewins 1, Glyn Lewis 6, Liming Li 173, 174, Bochao Danae Lin 175, Kuang Lin 92, Penelope A Lind 18, 26, 27, 28, Yu-Li Liu 176, Donald J MacIntyre 1, Dean F MacKinnon 106, Brion S Maher 122, Wolfgang Maier 177, Victoria S Marshe 123, 178, Gabriela A Martinez-Levy 179, Koichi Matsuda 180, 181, Hamdi Mbarek 102, Peter McGuffin 8, Sarah E Medland 18, 26, 182, 183, Susanne Meinert 52, 184, Christina Mikkelsen 108, 185, Susan Mikkelsen 109, Yuri Milaneschi 58, Iona Y Millwood 92,Esther Molina 86, 87, 186, Francis M Mondimore 106, Preben Bo Mortensen 24, 42, 43, Benoit H Mulsant 123, 160, Joonas Naamanka 147, Jake M Najman 187, Matthias Nauck 188, 189, Igor Nenadić 190, Kasper R Nielsen 191, Ilja M Nolte 192, Merete Nordentoft 77, 125, 126, Markus M Nöthen 94, Mette Nyegaard 43, 76, 193, Michael C O'Donovan 100, Asmundur Oddsson 65, Adrielle M Oliveira 194, Catherine M Olsen 195, 196, Hogni Oskarsson 197, Sisse Rye Ostrowski 108, 198, Michael J Owen 100, Richard Packer 199, Teemu Palviainen 170, Pedro M Pan 194, Carlos N Pato 200, Michele T Pato 200, Nancy L Pedersen 22, Ole Birger Pedersen 70, 198, Wouter J Peyrot 58, James B Potash 106, Martin Preisig 84, Michael H Preuss 201, 202, Jorge A Quiroz 203, Miguel E Renteria 18, 27, 28, Charles F Reynolds III 204, John P Rice 172, Saori Sakaue 154, 155, 205, Marcos L Santoro 206, Robert A Schoevers 207, 208, Andrew Schork 43, 209, Thomas G Schulze 2, 34, 106, 210, 211, 212, Tabea S Send 107, Jianxin Shi 213, Engilbert Sigurdsson 214, Kritika Singh 39, Grant C B Sinnamon 215, Lea Sirignano 2, 5, Olav B Smeland 119, 216, Daniel J Smith 217, Tamar Sofer 218, Erik Sørensen 108, Sundararajan Srinivasan 219, Hreinn Stefansson 65, Kari Stefansson 65, 138, Peter Straub 39, Mei-Hsin Su 220, André Tadic 124, 221, Henning Teismann 222, Alexander Teumer 223, Anita Thapar 100, 224, Pippa A Thomson 81, Lise Wegner Thørner 108, Apostolia Topaloudi 225, Shih-Jen Tsai 226, 227, Ioanna Tzoulaki 104, 105, 228, George Uhl 229, André G Uitterlinden 230, Henrik Ullum 108, 198, 231, Daniel Umbricht 232, Robert J Ursano 233, Sandra Van der Auwera 223, Albert M van Hemert 234, Abirami Veluchamy 219, Alexander Viktorin 22, Henry Völzke 235, G Bragi Walters 65, Xiaotong Wang 236, Agaz Wani 237, Myrna M Weissman 238, Jürgen Wellmann 222, David C Whiteman 195, Derek Wildman 237, Gonneke Willemsen 102, Alexander T Williams 199, Bendik S Winsvold 67, 68, 239, Stephanie H Witt 2, 5, 240, Ying Xiong 22, Lea Zillich 2, John-Anker Zwart 66, 67, 68, 23andMe Research Team 151, China Kadoorie Biobank, Collaborative Group 241, Estonian Biobank Research Team 165, Genes & Health Research Team 242, HUNT All-In Psychiatry 243, The BioBank Japan Project 244, VA Million Veteran Program 245, Ole A Andreassen 119, 216, 246, Bernhard T Baune 247, 248, 249, Klaus Berger 222, Dorret I Boomsma 102, 250, Anders D Børglum 43, 47, 48, Gerome Breen 8, 13, Na Cai 251, 252, 253, Hilary Coon 113, 115, William E Copeland 254, Byron Creese 56, Carlos S Cruz-Fuentes 179, Darina Czamara 71, Lea K Davis 39, 255, Eske M Derks 18, Enrico Domenici 256, Paul Elliott 104, 105, 228, 257, Andreas J Forstner 94, 96, 258, Micha Gawlik 259, Joel Gelernter 19, 20, 260, Hans J Grabe 223, Steven P Hamilton 261, Kristian Hveem 67, 262,

263, Catherine John 199, 264, Jaakko Kaprio 170, Tilo Kircher 190, Marie-Odile Krebs 265, Po-Hsiu Kuo 90, 266, Mikael Landén 22, 153, Kelli Lehto 165, Douglas F Levinson 267, Qingqin S Li 268, Klaus Lieb 124, Ruth J F Loos 185, 201, 269, 270, 271, Yi Lu 22, Susanne Lucae 149, Jurjen J Luykx 58, 175, 272, Hermine HM Maes 158, 220, 273, Patrik K Magnusson 22, Hilary C Martin 12, Nicholas G Martin 18, 26, Andrew McQuillin 6, Christel M Middeldorp 78, 274, Lili Milani 165, Ole Mors 43, 275, Daniel J Müller 123, 160, 161, 276, Bertram Müller-Myhsok 50, 277, 278, Yukinori Okada 154, 279, 280, Albertine J Oldehinkel 139, Sara A Paciga 281, Colin NA Palmer 219, Peristera Paschou 225, Brenda WJH Penninx 58, Roy H Perlis 9, 10, 282, Roseann E Peterson 60, Giorgio Pistis 84, Renato Polimanti 19, 32, David J Porteous 81, Danielle Posthuma 283, 284, Jill A Rabinowitz 285, Ted Reichborn-Kjennerud 53, Andreas Reif 44, Frances Rice 100, 224, Roland Ricken 7, Marcella Rietschel 2, Margarita Rivera 86, 87, 286, Christian Rück 49, Giovanni A Salum 287, Catherine Schaefer 288, Srijan Sen 73, 289, Alessandro Serretti 290, 291, Alkistis Skalkidou 166, Jordan W Smoller 9, 292, 293, Dan J Stein 82, Frederike Stein 294, Murray B Stein 295, 296, 297, 298, Patrick F Sullivan 22, 299, Martin Tesli 300, Thorgeir E Thorgeirsson 65, Henning Tiemeier 301, 302, Nicholas J Timpson 25, 303, Monica Uddin 237, Rudolf Uher 304, David A van Heel 148, Karin JH Verweij 305, Robin G Walters 92, Sylvia Wassertheil-Smoller 306, Jens R Wendland 55, Thomas Werge 77, 198, 209, 307, 308, Aeilko H Zwinderman 14

Karoline Kuchenbaecker* 6, 92, Naomi R Wray* 29, 236, 309, Stephan Ripke* 7, 293, Cathryn M Lewis* 8, 310, Andrew M McIntosh* 1, 811,

***** shared first and last authors

Author affiliations

1, Division of Psychiatry, University of Edinburgh, Edinburgh, UK
2, Department of Genetic Epidemiology in Psychiatry, Central Institute of Mental Health, Medical Faculty Mannheim,
Heidelberg University, Mannheim, BW, DE
3, Hector Institute for Artificial Intelligence in Psychiatry, Central Institute of Mental Health, Medical Faculty Mannheim,
Heidelberg University, Mannheim, BW, DE
4, Department for Psychiatry and Psychotherapy, Central Institute of Mental Health, Medical Faculty Mannheim, Heidelberg
University, Mannheim, BW, DE
5, German Center for Mental Health (DZPG), Partner Site Mannheim - Heidelberg - Ulm, DE
6, Division of Psychiatry, University College London, London, UK

7, Department of Psychiatry and Psychotherapy, Charité – Universitätsmedizin Berlin, Berlin, BE, DE
8, Social, Genetic and Developmental Psychiatry Centre, King's College London, London, UK
9, Department of Psychiatry, Massachusetts General Hospital, Boston, MA, US
10, Department of Psychiatry, Harvard Medical School, Boston, MA, US
11, Department of Clinical and Biomedical Sciences, Faculty of Health and Life Sciences, University of Exeter, Exeter, UK
12, Human Genetics, Wellcome Sanger Institute, Hinxton, UK
13, NIHR Maudsley Biomedical Research Centre, King's College London, London, UK
14, Epidemiologie en Data Science (EDS), Amsterdam UMC, location University of Amsterdam, Amsterdam, NL
15, Institute for Psychopharmacology, Central Institute of Mental Health, Medical Faculty Mannheim, Heidelberg University,
Mannheim, BW, DE
16, Department of Psychiatry, College of Health Sciences, University of Alberta, Edmonton, AB, CA
17, Neuroscience and Mental Health Institute, University of Alberta, Edmonton, AB, CA
18, Brain & Mental Health Program, QIMR Berghofer Medical Research Institute, Brisbane, QLD, AU
19, Department of Psychiatry, Yale University School of Medicine, New Haven, CT, US
20, Department of Psychiatry, Veterans Affairs Connecticut Healthcare System, West Haven, CT, US
21, Department of Clinical Medicine, Aarhus University, Aarhus, DK
22, Department of Medical Epidemiology and Biostatistics, Karolinska Institutet, Stockholm, SE
23, Department of Pediatric Neurology, Charité – Universitätsmedizin Berlin, Berlin, BE, DE
24, National Centre for Register-based Research, Aarhus University, Aarhus, DK
25, MRC Integrative Epidemiology Unit, University of Bristol, Bristol, UK
26, Mental Health and Neuroscience, QIMR Berghofer Medical Research Institute, Brisbane, QLD, AU
27, School of Biomedical Sciences, Queensland University of Technology, Brisbane, QLD, AU
28, School of Biomedical Sciences, The University of Queensland, Brisbane, QLD, AU
29, Institute for Molecular Bioscience, University of Queensland, Brisbane, QLD, AU
30, Morphology and Genetics, Universidade Federal de Sao Paulo, Sao Paulo, SP, BR
31, Maurice Wohl Clinical Neuroscience Institute, Department of Basic and Clinical Neuroscience, King's College London,
London, UK
32, Veterans Affairs Connecticut Healthcare System, West Haven, CT, US

33, Department of Psychiatry and Psychotherapy, University Hospital, LMU Munich, Munich, BY, DE
34, Institute of Psychiatric Phenomics and Genomics, University Hospital, LMU Munich, Munich, BY, DE
35, Department of Psychiatry and Psychotherapy, University Hospital Bonn, Medical Faculty, University of Bonn, Bonn, DE
36, Institute of Human Genetics, University Hospital Bonn, Medical Faculty, University of Bonn, Bonn, DE
37, German Center for Mental Health (DZPG), Partner Site Munich - Augsburg, DE
38, Old Age Psychiatry, King's College London, London, UK
39, Department of Medicine, Division of Genetic Medicine, Vanderbilt University Medical Center, Nashville, TN, US
40, Department of Psychiatry and Psychotherapy, Charité – Universitätsmedizin Berlin, Campus Charité Mitte (CCM),
Berlin, BE, DE
41, Center for Psychiatry, Psychotherapy and Psychosomatic Medicine, Fliedner Klinik Berlin, Berlin, BE, DE
42, Centre for Integrated Register-based Research, Aarhus University, Aarhus, DK
43, iPSYCH, The Lundbeck Foundation Initiative for Integrative Psychiatric Research, Aarhus, DK
44, Department of Psychiatry, Psychosomatic Medicine and Psychotherapy, Goethe University Frankfurt - University
Hospital, Frankfurt am Main, DE
45, Department of Epidemiology, Columbia University Mailman School of Public Health, New York, NY, US
46, Discipline of Psychiatry, University of Adelaide, Adelaide, SA, AU
47, Department of Biomedicine and Centre for Integrative Sequencing, iSEQ, Aarhus University, Aarhus, DK
48, Center for Genomics and Personalized Medicine, Aarhus University, Aarhus, DK
49, Department of Clinical Neuroscience, Karolinska Institutet, Stockholm, SE
50, Department of Translational Research in Psychiatry, Max Planck Institute of Psychiatry, Munich, BY, DE
51, Department of Neurology, Klinikum rechts der Isar, Technical University of Munich, Munich, BY, DE52, Institute for Translational Psychiatry, University of Münster, Münster, NRW, DE
53, PsychGen Centre for Genetic Epidemiology and Mental Health, Norwegian Institute of Public Health, Oslo, OSL, NO
54, PROMENTA Research Center, Department of Psychology, University of Oslo, Oslo, OSL, NO
55, Research and Development, Takeda Pharmaceutical Company Limited, Cambridge, MA, US
56, Faculty of Health and Life Sciences, University of Exeter, Exeter, UK

57, Novo Nordisk Foundation Center for Protein Research, Faculty of Health and Medical Sciences, University of
Copenhagen, Copenhagen, CPH, DK
58, Department of Psychiatry, Amsterdam Public Health and Amsterdam Neuroscience, Amsterdam UMC, Vrije Universiteit
Amsterdam, Amsterdam, NL
59, Laboratory of Integrative Neuroscience, Universidade Federal de Sao Paulo, Sao Paulo, SP, BR
60, Department of Psychiatry and Behavioral Sciences, Institute for Genomics in Health, State University of New York
Downstate Health Sciences University, Brooklyn, NY, US
61, Department of Psychiatry and Behavioral Sciences, Emory University School of Medicine, Atlanta, GA, US
62, Department Genes and Environment, Max Planck Institute of Psychiatry, Munich, BY, DE
63, Department of Mental Health, Faculty of Medicine and Health Sciences, Norwegian University of Science and
Technology (NTNU), Trondheim, TRD, NO
64, Faculty of Nursing and Health Sciences, NORD University, Levanger, NO
65, deCODE Genetics / Amgen, Reykjavik, IS
66, Institute of Clinical Medicine, Faculty of Medicine, University of Oslo, Oslo, OSL, NO
67, HUNT Center for Molecular and Clinical Epidemiology, Department of Public Health and Nursing, Faculty of Medicine
and Health Sciences, Norwegian University of Science and Technology, Trondheim, TRD, NO
68, Department of Research and Innovation, Division of Clinical Neuroscience, Oslo University Hospital, Oslo, OSL, NO
69, Institute of Environmental Medicine, Unit of Integrative Epidemiology, Karolinska Institutet, Stockholm, SE
70, Department of Clinical Immunology, Zealand University Hospital, Køge, DK
71, Department Genes and Environment, Max Planck Institute of Psychiatry, Munich, BY, DE
72, Department of Clinical Immunology, Odense University Hospital, Odense, DK
73, Michigan Neuroscience Institute, University of Michigan, Ann Arbor, MI, US
74, Department of Psychiatry and Psychotherapy, Gender Research in Medicine, Institute of Sexology and Sexual
Medicine, Charité – Universitätsmedizin Berlin, Berlin, BE, DE
75, Department of Psychiatry, Psychotherapy and Psychosomatics, Brandenburg Medical School Theodor Fontane,
Neuruppin, BB, DE

76, Center for Neonatal Screening, Department for Congenital Disorders, Statens Serum Institut, Copenhagen, CPH, DK
77, iPSYCH, The Lundbeck Foundation Initiative for Integrative Psychiatric Research, Copenhagen, CPH, DK
78, Child Health Research Centre, University of Queensland, Brisbane, QLD, AU
79, Department of Biostatistics , University of North Carolina at Chapel Hill , Chapel Hill, NC, US
80, Centre for Medical Informatics, Usher Institute, University of Edinburgh, Edinburgh, UK
81, Centre for Genomic & Experimental Medicine, Institute for Genetics and Cancer, University of Edinburgh, Edinburgh,
UK
82, SAMRC Unit on Risk & Resilience in Mental Disorders, Department of Psychiatry and Neuroscience Institute, University
of Cape Town, Cape Town, SA
83, Statistical genetics, Institute for Molecular Bioscience, The University of Queensland, Brisbane, QLD, AU
84, Department of Psychiatry, Lausanne University Hospital and University of Lausanne, Prilly, VD, CH
85, Department of Psychiatry, Faculty of Medicine, University of Granada, Granada, ES
86, Instituto de Investigación Biosanitaria, Ibs Granada, Granada, ES
87, Institute of Neurosciences ´Federico Olóriz´, Biomedical Research Centre (CIBM), University of Granada, Granada, ES
88, Université de Paris Cité, INSERM U1266, Institute of Psychiatry and Neuroscience of Paris, GHU Paris Psychiatry and
Neuroscience, Paris, FR
89, Biogen, Cambridge, MA, US
90, Department of Psychiatry, National Taiwan University Hospital,, TW
91, School of Medicine, National Taiwan University College of Medicine, Taipei, TW
92, Nuffield Department of Population Health, University of Oxford, Oxford, UK
93, Human Genomics Research Group, Department of Biomedicine, University of Basel, Basel, CH
94, Institute of Human Genetics, University of Bonn, School of Medicine & University Hospital Bonn, Bonn, DE
95, Institute of Medical Genetics and Pathology, University Hospital Basel, University of Basel, Basel, CH
96, Institute of Neuroscience and Medicine (INM-1), Research Center Juelich, Juelich, DE
97, School of Psychology, University of Queensland, Brisbane, QLD, AU
98, Nic Waals Institute, Lovisenberg Diakonale Hospital, Oslo, OSL, NO

99, Centre for Advanced Imaging, University of Queensland, Saint Lucia, QLD, AU100, Centre for Neuropsychiatric Genetics and Genomics, Cardiff University, Cardiff, UK
101, The Lothian Birth Cohorts, University of Edinburgh, Edinburgh, UK
102, Department of Biological Psychology & Amsterdam Public Health Research Institute, Vrije Universiteit Amsterdam,
Amsterdam, NL
103, Department of Child and Adolescent Psychiatry, Psychosomatics and Psychotherapy, University Hospital Essen,
University of Duisburg-Essen, Duisburg, DE
104, MRC Centre for Environment and Health, School of Public Health, Imperial College London, London, UK
105, Imperial College Dementia Research Institute, Imperial College London, London, UK
106, Department of Psychiatry and Behavioral Sciences, Johns Hopkins University School of Medicine, Baltimore, MD, US
107, Department of Psychiatry and Psychotherapy, Research Group Stress Related Disorders, Central Institute of Mental
Health, Medical Faculty Mannheim, Heidelberg University, Mannheim, BW, DE
108, Department of Clinical Immunology, Copenhagen University Hospital, Rigshospitalet, Copenhagen, CPH, DK
109, Department of Clinical Immunology, Aarhus University Hospital, Aarhus, DK
110, Department of Psychiatry, Istanbul University, Istanbul, TR
111, Department of Medical Genetics, Oslo University Hospital, Oslo, OSL, NO
112, NORMENT, Department of Clinical Science, University of Bergen, Bergen, NO
113, Psychiatry, University of Utah School of Medicine, Salt Lake City, UT, US
114, Center for Genomic Medicine, Salt Lake City, UT, US
115, Huntsman Mental Health Institute, Salt Lake City, UT, US
116, Department of Psychiatry and Psychotherapy, Medical Center, University of Freiburg, Faculty of Medicine, University
of Freiburg, Freiburg, DE
117, Division of Mental Health Care, St. Olavs Hospital, Trondheim University Hospital, Trondheim, TRD, NO
118, Department of Psychiatry, Sørlandet Hospital, Kristiansand, AG, NO
119, NORMENT, Institute of Clinical Medicine, University of Oslo, Oslo, OSL, NO
120, Center for Genomic Medicine, Massachusetts General Hospital, Boston, MA, US
121, Department of Sociology, Purdue University, West Lafayette, IN, US
122, Department of Mental Health, Johns Hopkins, Baltimore, MD, US
123, Centre for Addiction and Mental Health, Toronto, ON, CA

124, Department of Psychiatry and Psychotherapy, University Medical Center of the Johannes Gutenberg University Mainz,
Mainz, DE
125, Mental Health Center Copenhagen, Mental Health Services Capital Region of Denmark, Copenhagen, CPH, DK
126, Faculty of Health Science, Department of Clinical Medicine, University of Copenhagen, Copenhagen, CPH, DK
127, Department of Biomedical and Neuromotor Sciences, University of Bologna, Bologna, IT
128, Wolfson Institute of Population Health, Queen Mary University of London, London, UK
129, School of Computing and Mathematical Sciences, University of Leicester, Leicester, UK
130, Department of Psychology, San Diego San Diego State University, San Diego, CA, US
131, Department of Epidemiology and Biostatistics, Imperial College London, London, UK
132, Discipline of Psychiatry, School of Medicine, Trinity College Dublin, Dublin, IE
133, Bioinformatics Research Centre, Aarhus University, Aarhus, DK
134, School of Engineering, University of Iceland, Reykjavik, IS
135, Danish Headache Centre, Department of Neurology, Rigshospitalet, Glostrup, DK
136, Neurogenomics Group, Translational Research Centre, Rigshospitalet Copenhagen University Hospital, Glostrup, DK
137, Landspitali-University Hospital, Reykjavik, IS
138, Faculty of Medicine, University of Iceland, Reykjavik, IS
139, Department of Psychiatry, University of Groningen, University Medical Center Groningen, Groningen, NL
140, Nic Waals Institute, Lovisenberg Diaconal Hospital, Oslo, OSL, NO
141, MRC Human Genetics Unit, Institute for Genetics and Cancer, University of Edinburgh, Edinburgh, UK
142, Brain and Mind Centre, University of Sydney, Sydney, NSW, AU
143, Department of Epidemiology Research, Statens Serum Institut, Copenhagen, CPH, DK
144, Department of Medical Biochemistry and Biophysics, Karolinska Institutet, Stockholm, SE
145, Interfaculty Institute for Genetics and Functional Genomics, Department of Functional Genomics, University Medicine
Greifswald, Greifswald, MV, DE
146, Roche Pharmaceutical Research and Early Development, Pharmaceutical Sciences, Roche Innovation Center Basel,
F. Hoffmann-La Roche Ltd, Basel, CH
147, SleepWell Research Program and Department of Psychology and Logopedics, University of Helsinki, Helsinki, FI
148, Blizard Institute, Barts and the London School of Medicine and Dentistry, Queen Mary University of London, London,

UK
149, Max Planck Institute of Psychiatry, Munich, BY, DE150, Department of Psychiatry, University of Helsinki, Helsinki, FI
151, 23andMe Research Team, 23andMe, Inc., Sunnyvale, CA, US
152, Department of Psychological Medicine, University of Worcester, Worcester, UK
153, Institution of Neuroscience and Physiology, University of Gothenburg, Gothenburg, SE
154, Department of Statistical Genetics, Osaka University Graduate School of Medicine, Suita, JP
155, Program in Medical and Population Genetics, Broad Institute of Harvard and MIT, Cambridge, MA, US
156, Center for Computational and Integrative Biology, Massachusetts General Hospital, Boston, MA, US
157, Center for Brain Research, Department of Molecular Neuroscience, Medical University of Vienna, Vienna, AT
158, Department of Psychiatry, Virginia Commonwealth University, Richmond, VA, US
159, Department of Health Care Policy, Harvard Medical School, Boston, MA, US
160, Department of Psychiatry, University of Toronto, Toronto, ON, CA
161, Department of Pharmacology & Toxicology, University of Toronto, Toronto, ON, CA
162, Department of Genetics, Rutgers University, Piscataway, NJ, US
163, Department of Psychiatry, Perelman School of Medicine, University of Pennsylvania, Philadelphia, PA, US
164, Mental Illness Research, Education and Clinical Center, Crescenz VA Medical Center, Philadelphia, PA, US
165, Estonian Genome Centre, Institute of Genomics, University of Tartu, Tartu, EE
166, Department of Women's and Children's Health, Uppsala University, Uppsala, SE
167, Department of Epidemiology and Health Systems, Center for Primary Care and Public Health, Lausanne, VD, CH
168, Department of Computational Biology, University of Lausanne, Lausanne, VD, CH
169, Swiss Institute of Bioinformatics, Lausanne, VD, CH
170, Institute for Molecular Medicine Finland - FIMM, University of Helsinki, Helsinki, FI
171, SAMRC Unit on Risk & Resilience in Mental Disorders, Department of Psychiatry and Neuroscience Institute,
University of Cape Town, Cape Town, SA
172, Department of Psychiatry, Washington University School of Medicine in St. Louis, St. Louis, MO, US
173, Department of Epidemiology and Biostatistics, School of Public Health, Peking University, Beijing, CN

174, Peking University Center for Public Health and Epidemic Preparedness & Response, Peking University, Beijing, CN
175, Department of Psychiatry and Neuropsychology, School for Mental Health and Neuroscience, Maastricht University
Medical Centre, Maastricht, NL
176, Center for Neuropsychiatric Research, National Health Research Institutes,, TW
177, Department of Psychiatry and Psychotherapy, University of Bonn, Bonn, DE
178, Center for Translational and Computational Neuroimmunology, Columbia University Medical Center, New York, NY,
US
179, Psychiatric Genetics Department, Instituto Nacional de Psiquiatría Ramón de la Fuente Muñiz (INPRFM), Mexico City,
CDMX, MX
180, Laboratory of Genome Technology, Human Genome Center, Institute of Medical Science, The University of Tokyo,
Tokyo, JP
181, Laboratory of Clinical Genome Sequencing, Department of Computational Biology and Medical Sciences, Graduate
School of Frontier Sciences, The University of Tokyo, Tokyo, JP
182, School of Psychology, The University of Queensland, Brisbane, QLD, AU
183, School of Psychology and Counselling, Queensland University of Technology, Brisbane, QLD, AU
184, Institute for Translational Neuroscience, University of Münster, Münster, NRW, DE
185, Novo Nordisk Foundation Center for Basic Metabolic Research, Faculty of Health and Medical Sciences, University of
Copenhagen, Copenhagen, CPH, DK
186, Department of Nursing, Faculty of Health Sciences, University of Granada, Granada, ES
187, School of Public Health, University of Queensland, Brisbane, QLD, AU
188, Institute of Clinical Chemistry and Laboratory Medicine, University Medicine Greifswald, Greifswald, MV, DE
189, DZHK (German Centre for Cardiovascular Research), Partner Site Greifswald, Greifswald, MV, DE
190, Department of Psychiatry, University of Marburg, Marburg, HE, DE
191, Department of Clinical Immunology, Aalborg University Hospital, Aalborg, DK
192, Department of Epidemiology, University of Groningen, University Medical Center Groningen, Groningen, NL
193, Department of Health, Science and Technology, Aalborg University, Aalborg, DK
194, Department of Psychiatry, Universidade Federal de Sao Paulo, Sao Paulo, SP, BR

195, Population Health, QIMR Berghofer Medical Research Institute, Brisbane, QLD, AU
196, The Fraser Institute, Faculty of Medicine, University of Queensland, Brisbane, QLD, AU
197, Directorate of Health, Iceland, Reykjavik, IS
198, Department of Clinical Medicine, University of Copenhagen, Copenhagen, CPH, DK
199, Department of Population Health Sciences, University of Leicester, Leicester, UK
200, Department of Psychiatry, Rutgers University, Piscataway, NJ, US201, Charles Bronfman Institute for Personalized Medicine, Icahn School of Medicine at Mount Sinai, New York, NY, US
202, Department of Environmental Medicine and Public Health, Icahn School of Medicine at Mount Sinai, New York, NY, US
203, Translational Medicine, Roche, New York, NY, US
204, Psychiatry, University of Pittsburgh Medical Centre, Pittsburgh, PA, US
205, Divisions of Genetics and Rheumatology, Department of Medicine, Brigham and Women's Hospital, Harvard Medical
School, Boston, MA, US
206, Department of Biochemistry, Universidade Federal de Sao Paulo, Sao Paulo, SP, BR
207, Department of Psychiatry, University Medical Center Groningen, Groningen, NL
208, Research School of Behavioural and Cognitive Neurosciences (BCN), University of Groningen, Groningen, NL
209, Institute of Biological Psychiatry, Mental Health Center Sct. Hans, Mental Health Services Capital Region of Denmark,
Copenhagen, CPH, DK
210, Department of Psychiatry and Psychotherapy, University Medical Center Göttingen, Goettingen, NI, DE
211, Human Genetics Branch, NIMH Division of Intramural Research Programs, Bethesda, MD, US
212, Department of Psychiatry and Behavioral Sciences, SUNY Upstate Medical University, Syracuse, NY, USA
213, Division of Cancer Epidemiology and Genetics, National Cancer Institute, Bethesda, MD, US
214, Faculty of Medicine, Department of Psychiatry, University of Iceland, Reykjavik, IS
215, School of Medicine and Dentistry, James Cook University, Townsville, QLD, AU
216, Division of Mental Health and Addiction, Oslo University Hospital, Oslo, OSL, NO
217, Center for Clinical Brain Sciences, University of Edinburgh, Edinburgh, UK
218, Beth Israel Deaconess Medica, Harvard Medical School, Boston, MA, US
219, Division of Population Health and Genomics, Ninewells Hospital and School of Medicine, University of Dundee,
Dundee, UK
220, Virginia Institute for Psychiatric and Behavioral Genetics, Virginia Commonwealth University, Richmond, VA, US

221, Department of Psychiatry, Psychotherapy and Psychosomatics, Dr. Fontheim Mentale Gesundheit, Liebenburg, DE
222, Institute of Epidemiology and Social Medicine, University of Münster, Münster, NRW, DE
223, Department of Psychiatry and Psychotherapy, University Medicine Greifswald, Greifswald, MV, DE
224, Wolfson Centre for Young People's Mental Health, Division of Psychological Medicine and Clinical Neurosciences,
Cardiff University, Cardiff, UK
225, Department of Biological Sciences, Purdue University, West Lafayette, IN, US
226, Institute of Brain Science & Division of Psychiatry, National Yang-Ming University,, TW
227, Department of Psychiatry, Taipei Veterans General Hospital, Taipei, ROC, TW
228, Imperial College BHF Centre for Research Excellence, Imperial College London, London, UK
229, University of Maryland School of Medicine and VA Maryland Healthcare System, Baltimore, MD, US
230, Department of Internal Medicine, Erasmus University Medical Center Rotterdam, Rotterdam, NL
231, Management Section, Statens Serum Institut, Copenhagen, CPH, DK
232, Xperimed LLC, Basel, CH
233, Department of Psychiatry, Uniformed Services University of the Health Sciences, Bethesda, MD, US
234, Department of Psychiatry, Leiden University Medical Center, Leiden, NL
235, Institute for Community Medicine, University Medicine Greifswald, Greifswald, MV, DE
236, Department of Psychiatry, University of Oxford, Oxford, UK
237, Genomics Program, University of South Florida College of Public Health, Tampa, FL, US
238, Columbia University Vagelos College of Physicians and Surgeons, New York, NY, US
239, Department of Neurology, Oslo University Hospital, Oslo, OSL, NO
240, Center for Innovative Psychiatric and Psychotherapeutic Research, Central Institute of Mental Health, Medical Faculty
Mannheim, Heidelberg University, Mannheim, BW, DE
241, China Kadoorie Biobank Collaborative Group
242, Genes & Health Research Team
243, HUNT All-In Psychiatry
244, The BioBank Japan Project
245, VA Million Veteran Program
246, KG Jebsen Centre for Neurodevelopmental Research, University of Oslo, Oslo, OSL, NO
247, Department of Psychiatry, University of Münster, Münster, NRW, DE
248, Department of Psychiatry, University of Melbourne, Melbourne, VIC, AU

249, Florey Institute of Neuroscience and Mental Health, University of Melbourne, Melbourne, VIC, AU
250, Department of Complex Trait Genetics, CNCR, Vrije Universiteit Amsterdam, Amsterdam, NL
251, Helmholtz Pioneer Campus, Helmholtz Zentrum München, Neuherberg, DE
252, Computational Health Centre, Helmholtz Zentrum München, Neuherberg, DE
253, School of Medicine, Technical University of Munich, Munich, BY, DE254, Department of Psychiatry, University of Vermont, Burlington, VT, US
255, Department of Medicine, Icahn School of Medicine at Mount Sinai, New York, NY, US
256, Department of Cellular, Computational and Integrative Biology, Università degli Studi di Trento, Trento, IT
257, Imperial College Biomedical Research Centre, Imperial College London, London, UK
258, Center for Human Genetics, University of Marburg, Marburg, HE, DE
259, Department of Psychiatry, Psychosomatics and Psychotherapy, Julius-Maximilians-Universität Würzburg, Würzburg,
DE
260, Department of Genetics, Department of Neuroscience, Yale University School of Medicine, New Haven, CT, US
261, Psychiatry, Kaiser Permanente Northern California, San Francisco, CA, US
262, HUNT Research Center, Department of Public Health and Nursing, Faculty of Medicine and Health Sciences,
Norwegian University of Science and Technology (NTNU), Trondheim, NO
263, Department of Research, Innovation and Education, St. Olavs Hospital, Trondheim University Hospital, Trondheim,
TRD, NO
264, University Hospitals of Leicester NHS Trust, Leicester, UK
265, Pathophysiology of Psychiatric Diseases, INSERM, Univ Paris Cité, GHU Paris, Paris, FR
266, Institute of Epidemiology and Preventive Medicine & Department of Public Health, National Taiwan University,, TW
267, Department of Psychiatry & Behavioral Sciences, Stanford University, Stanford, CA, US
268, Neuroscience Therapeutic Area, Janssen Research and Development, LLC, Titusville, NJ, US
269, Mindich Child Health and Development Institute, Icahn School of Medicine at Mount Sinai, New York, NY, US
270, Department of Environmental Medicine and Public Health, Icahn School of Medicine at Mount Sinai, New York, NY, US
271, MRC Metabolic Diseases Unit, University of Cambridge Metabolic Research Laboratories, Wellcome-MRC Institute of
Metabolic Science, Addenbrooke's Hospital, Cambridge, UK

272, Bipolar Disorders Outpatient Clinic, GGZ InGeest, Amsterdam, NL
273, Department of Human and Molecular Genetics, Virginia Commonwealth University, Richmond, VA, US
274, Child and Youth Mental Health Service, Children's Health Queensland Hospital and Health Service, Brisbane, QLD,
AU
275, Psychosis Research Unit, Aarhus University Hospital-Psychiatry, Aarhus, DK
276, Department of Psychiatry, Psychosomatics and Psychotherapy, University Hospital of Würzburg, Würzburg, DE
277, Munich Cluster for Systems Neurology (SyNergy), Munich, BY, DE
278, University of Liverpool, Liverpool, UK
279, Department of Genome Informatics, Graduate School of Medicine, The University of Tokyo, Tokyo, JP
280, Laboratory for Systems Genetics, RIKEN Center for Integrative Medical Sciences, Yokohama, JP
281, Human Genetics and Computational Biomedicine, Pfizer Global Research and Development, Groton, CT, US
282, Centre for Quantitative Health, Massachusetts General Hospital, Boston, MA, US
283, Child and Adolescent Psychiatry, Amsterdam UMC, Vrije Universiteit Amsterdam, Amsterdam, NL
284, Complex Trait Genetics, Vrije Universiteit Amsterdam, Amsterdam, NL
285, Department of Mental Health, Johns Hopkins University, Baltimore, MD, US
286, Department of Biochemistry and Molecular Biology II, Faculty of Pharmacy, University of Granada, Granada, ES
287, Psychiatry, Universidade Federal do Rio Grande do Sul, Porto Alegre, BR
288, Division of Research, Kaiser Permanente Northern California, Oakland, CA, US
289, Eisenberg Family Depression Center, University of Michigan, Ann Arbor, MI, US
290, Department of Medicine and Surgery, Kore University of Enna, Enna, IT
291, Psychiatry, Oasi Research Institute-IRCCS, Troina, IT
292, Psychiatric and Neurodevelopmental Genetics Unit, Massachusetts General Hospital, Boston, MA, US
293, Stanley Center for Psychiatric Research, Broad Institute of MIT and Harvard, Cambridge, MA, US
294, Department of Psychiatry and Psychotherapy, University of Marburg, Marburg, HE, DE
295, Psychiatry Service, Veterans Affairs San Diego Healthcare System, San Diego, CA, US
296, School of Public Health, University of California, San Diego, La Jolla, CA, US
297, Department of Psychiatry, University of California, San Diego, La Jolla, CA, US
298, Psychiatry, Veterans Affairs San Diego Healthcare System, San Diego, CA, US

299, Departments of Genetics and Psychiatry, University of North Carolina at Chapel Hill, Chapel Hill, NC, US
300, Department of Mental Health and Suicide, Norwegian Institute of Public Health, Oslo, OSL, NO
301, Child and Adolescent Psychiatry, Erasmus University Medical Center Rotterdam, Rotterdam, NL
302, Social and Behavioral Science, Harvard T.H. Chan School of Public Health, Boston, MA, US
303, Population Health Sciences, Bristol Medical School, University of Bristol, Bristol, UK
304, Psychiatry, Dalhousie University, Halifax, NS, CA
305, Psychiatry, Amsterdam UMC, location University of Amsterdam, Amsterdam, NL
306, Epidemiology and Population Health, Albert Einstein College of Medicine, Bronx, NY, US307, Institute of Biological Psychiatry, Mental Health Center Sct. Hans, Copenhagen University Hospital, Mental Health
Services, Copenhagen, CPH, DK
308, GLOBE Institute, Lundbeck Foundation Centre for Geogenetics, University of Copenhagen, Copenhagen, CPH, DK
309, Queensland Brain Institute, University of Queensland, Brisbane, QLD, AU
310, Department of Medical & Molecular Genetics, King's College London, London, UK
