# Supplementary figures and images for: Trans-ancestry genome-wide study of depression identifies 697 associations implicating cell types and pharmacotherapies
Source: Cell. Author manuscript; Available in PMC 2025 Feb 15. (PMC11829167; doi:10.1016/j.cell.2024.12.002)

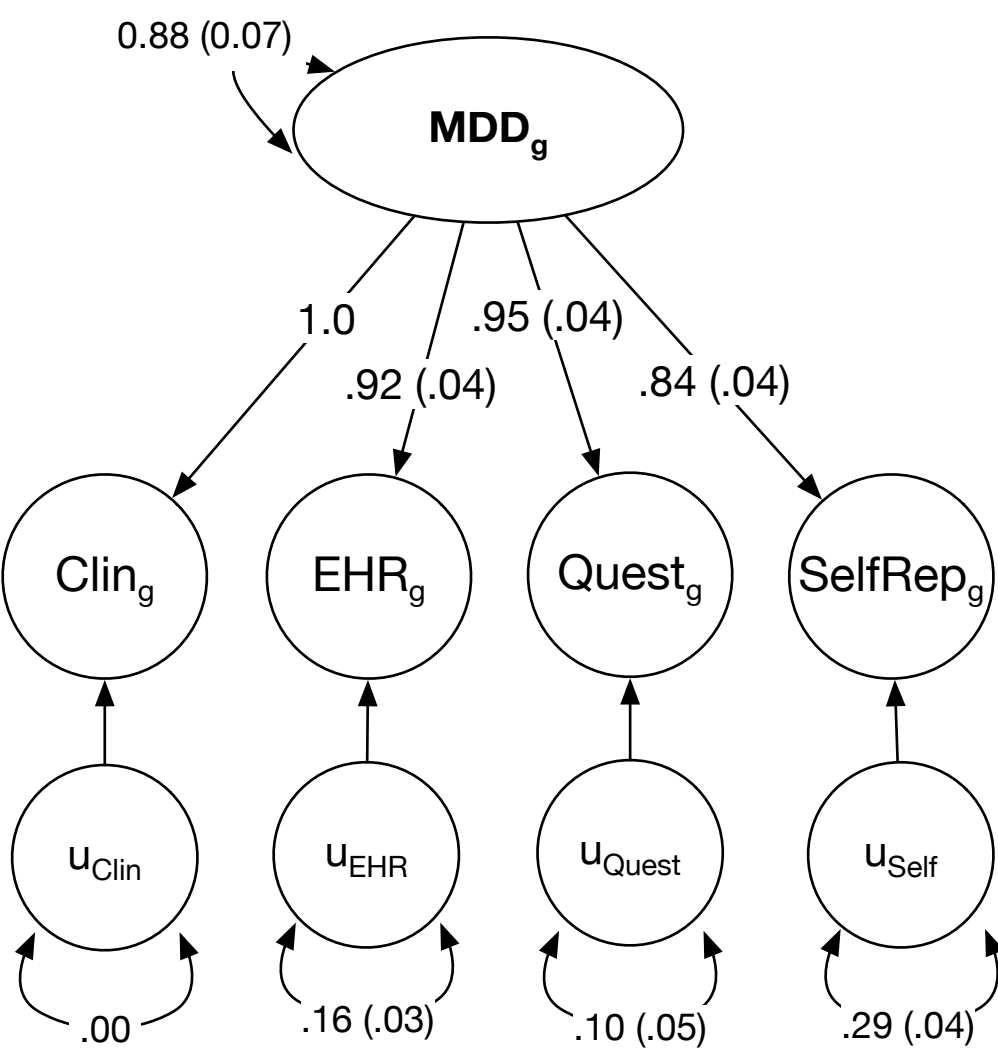

Supplement: 2 — Figure S1. Path diagram showing genetic loadings of MDD phenotype definitions on a latent MDD factor, related to the STAR Methods Clin, clinical; EHR, electronic health record; Quest, questionnaire; SelfRep, self-report of MDD diagnosis. Numbers represent standardised loadings with standard errors in brackets from genomic structural equation model, with the loading of the Clinical MDD phenotype constrained to 1.0. Self-directed arrows indicate variance of the MDD factor or residual variances of the MDD phenotypes (“u”). [file NIHMS2048064-supplement-2.pdf]
