# Supplementary material for: Trans-ancestry genome-wide study of depression identifies 697 associations implicating cell types and pharmacotherapies
Source: Cell. Author manuscript; Available in PMC 2025 Feb 15. (PMC11829167; doi:10.1016/j.cell.2024.12.002)
Supplement: 3 [file NIHMS2048064-supplement-3.pdf]

Methods S2: Supplementary Study Information

Supplementary study information, related to STAR Methods

Table of Contents

Table of Contents 1

PGC wave 1 6

Genotype samples 6

Bonn/Mannheim (BOMA) | Rietschel M | PMID 20673876; 22736164; 16032513; 12177636 | analysis code: boma 6

Edinburgh (UEDIN) | McIntosh AM; Blackwood DHR; Smith D; Adams MJ | PMID 21042317 | analysis code: edi2 8

Max Planck Institute of Psychiatry (MPIP) | Müller-Myhsok B; Binder E | PMID 19107115 | analysis code: gsk2 9

RADIANT | Lewis CM; Breen G; Mors O | PMID 20516156 | analysis code: rad3, rage, rai2, rau2, rde4 9

Sequenced Treatment Alternatives to Relieve Depression (STAR*D) | Hamilton SP | PMID 20038947 | analysis code: stm2 12

PGC wave 2 13

Genotype samples 13

Cognitive Function and Mood Study (CoFaMS) | Baune BT | PMID 27616997 | analysis code: cof3 13

CoLaus¦PsyCoLaus | Preisig M | PMID 19292899 | analysis code: col3 14

Genetics of Recurrent Early-Onset Depression Phase I (GenRED1) | Levinson D | PMID 12707949; 20125088 | analysis code: gens 16

Genetic Predictors of Outcome in Depression (GenPod) / Novel Methods leading to New Medications in Depression and Schizophrenia (NEWMEDS) / Welcome Trust Case-Control Consortium (WTCCC) | Uher R | PMID 18498636; 21263010; 23091423; 17554300 | analysis code: gep3 18

Depression Genes and Networks (DGN) | Levinson D | PMID 24092820 | analysis code: grdg 20

The Netherlands Study of Depression and Anxiety (NESDA) / The Netherlands Twin Register (NTR) | Penninx B; Boomsma D | PMID 33773360; 17254420 | analysis code: nes1 22

Genetics of Recurrent Early-Onset Depression Phase II (GenRED2) | Levinson D | PMID 29700475 | analysis code: grnd 24

Harvard i2b2 (i2b2-TRD) | Perlis RH; Smoller JW | PMID 22472876 | analysis code: i2b3 25

Munich Antidepressant Response Signature (MARS) | Lucae S | PMID 21521612; 19736353; 18586274 | analysis code: mmi2, mmo4 26

Dortmund Health Study Controls (DHS) | Berger K | PMID 18677645 | analysis code: mmo4 26

Pfizer | Paciga SA | PMID 23091423 | analysis code: pfm2 28

QIMR Berghofer Medical Research Institute (QIMR) | Martin NG | PMID 21042317 | analysis code: qi3c, qi6c, qio2 29

Roche | Domenici E | PMID 27304433; 32663909 | analysis code: roc3 31

Rotterdam Study (Rotterdam) | Tiemeier H | PMID 19047526 | analysis code: rot4 32

Study of Health in Pomerania - Life-Events and Gene-Environment Interaction in Depression (SHIP-LEGEND) | Grabe HJ; Völzke H | PMID 25077849; 20167617; 35348705 | analysis code: shp0 34

SHIP-TREND | Grabe HJ; Völzke H | PMID 20167617; 35348705 | analysis code: shpt 34

TwinGene | Kendler K; Magnusson P | PMID 16390897, 23137839 | analysis code: twg2 36

PGC wave 3 38

Genotype samples 38

Janssen | Li QS | PMID 16760927; 26707087; 28086004 | analysis code: janpy 38

Ziekenhuisnetwork Antwerpen (ZNA) | Luykx J | PMID 35896057; 35649338 | analysis code: antpo 41

Pre-, Peri- and POstnatal Stress: Epigenetic Impact on DepressiON (POSEIDON) | Rietschel M; Deuschle M; Streit F; Witt S | PMID 30471571 | analysis code: antpo 42

Study “Establishing the bidirectional link between subclinical arteriosclerosis and depression” (BiDirect) | Berger K | PMID 28348383 | analysis code: bidi 42

Early Prediction of Adolescent Depression (EPAD) (CARD3) | Rice F; Thapar A | PMID 21962850; 34706428 | analysis code: cardm 43

McQuillin controls | McQuillin A | PMID 30859703 | analysis code: cardm 46

The Early Medication Change (EMC) Trial | Lieb K; Tadic A, Engelmann J | PMID 20187947 | analysis code: emcbp 47

Berlin Psychosis Study (BePS) / Berlin Research Initiative for Diagnostics, Genetics and Environmental Factors (BRIDGE-S) | Ripke S | PMID 35396580; 36635663 | analysis code: emcbp 49

FOR2107 | Kircher T; Andlauer T; Forstner AJ; Dannlowski U | PMID 30267149; 31076262 | analysis code: formm 51

Great Smoky Mountains Study (GSMS) | Copeland W; Costello EJ | PMID 27010203; 24342383 | analysis code: gsmse 53

TRD3 European Group for the Study of Resistant Depression (GSRD) | Serretti A; Kasper S; Souery JZD; Montgomery S; Dikeos D; Rujescu D; Mendlewicz J | PMID 30468137 | analysis code: gsrdf-gsrdg-gsrdi-gsrdp 53

PSYDEV controls | Krebs MO | PMID 35396580 | analysis code: gsrdf 55

Gawlik schizophrenia controls (Gawlik controls) | Gawlik M | PMID 35396580 | analysis code: gsdrg 56

Serretti schizophrenia controls (Serretti controls) | Serretti A | PMID 35396580 | analysis code: gsrdi 57

Paschou controls | Paschou P | PMID 29392977 | analysis code: gsrdp 58

Intern Health Study (IHS) | Sen S; Fang Y | PMID 31659322 | analysis code: ihseu; IHS_DIV 59

Incomplete Response in Late-Life Depression (IRL-GRey) | Mueller DJ; Mulsant BH; Reynolds IIIrd C; Lenze EJ] PMID 28068779; 26423282; 26963689 | analysis code: iruts 60

Utah Suicide Genetic Research Study (USGRS) | Coon H; Lewis CM; Docherty A; Shabalin A; DiBlasi E; Stahl E; Mullins E | PMID 30353169 | analysis code: iruts 62

Smoller bipolar disorder controls (Smoller controls) | Smoller JW | PMID 34002096 | analysis code: iruts 63

GeneBaseMDD | Ricken R; Adli M | PMID 30554862 | analysis code: mazdr 64

Reif bipolar disorder controls (Reif controls) | Reif A | PMID 28167838; 27145764 | analysis code: mazdr 65

Epidemiological Study on Mental Health in the Autonomous Community of Andalusia (PISMA-ep) | Cervilla J; Rivera M; Gutierrez B; Molina E | PMID 27237041 | analysis code: mrive 66

MUENS | Baune BT | PMID 29700475 | analysis code: muen2 68

Mater University Study of Pregnancy (MUSPC) | Middeldorp C; Najman J; Wray N | 25519422 | analysis code: muspc 69

Platform for Research Online to investigate Genetics and Cognition in Ageing (PROTECT) | Creese B; Corbett A; Ballard C; Aarsland D | PMID 28128869 | analysis code: prote 70

Thematically Organized Psychosis (TOP) Study | Andreassen O; Djurovic S; Smeland OB | PMID 20185149 | analysis code: topmd 72

Tracking Adolescents' Individual Lives Survey (TRAILS) | Oldehinkel AJ | PMID 25431468; 18263649 | analysis code: trail 73

Yale-Penn (YP) Study | Gelernter J; Polimanti R; Kranzler H | PMID 23958962; 24143882; 24166409; 25555482 | analysis code: yapeu 74

Summary samples 75

23andMe | 23andMe Research Team | PMID 27479909 | analysis code: mdd_23andMe.eur.hg19.v7_2_202012; 23andMe_AFR; 23andMe_EAS; 23andMe_EUR; 23andMe_HIS 75

Australian Genetics of Depression Study (AGDS1) | Martin N; Byrne E; Wray N; Medland S; Mitchell B | PMID 32461290; 34924174 | analysis code: mdd_AGDS.eur.hg19.202012 77

Avon Longitudinal Study of Parents and Children (ALSPAC) | Timpson NJ; Kwong ASF | PMID 22507743; 22507742; 31020050 | analysis code: mdd_ALSPAC.eur.hg19.27022020 79

Airwave Health Monitoring Study (AHMS) | Elliot P; McIntosh A | PMID 25194498 | analysis code: mdd_Airwave.eur.hg19.0820 81

The Biology, Affect, Stress, Imaging and Cognition Study (BASIC) | Skalkidou A; Lu Y; Bränn E; Kunovac Kallak T | PMID 31641004 | analysis code: mdd_BASIC.eur.hg19.202011 82

Biorepository of Vanderbilt University (BioVU) | Davis LK | PMID 20443953; 18500243 | analysis code: mdd_BioVU.eur.hg19.NoCov_SAIGE_051821; BioVU_AFR 84

Copenhagen Hospital Biobank (CHB)/ Danish Blood Donator Study (DBDS) | Thorgeirsson T; Werge T | PMID 33169150; 31182452 | analysis code: mdd_DBDS.eur.hg19.FINAL202103 86

deCODE | Thorgeirsson T | PMID 29700475 | analysis code: mdd_deCODE.eur.hg19.DEPALL_FINAL_WHEAD 88

Estonian Biobank (EstBB) | Lehto K | PMID 24518929; 28401899 | analysis code: mdd_ESTBB.eur.hg19.EstBB 90

Extended Cohort for E-health, Environment and DNA (EXCEED) | Tobin M | PMID 31062032 | analysis code: mdd_EXCEED.eur.hg19.202010 92

FinnGen | Kaprio J | PMID 36653562 | analysis code: mdd_FinnGen.eur.hg19.R5_18032020 94

Generation Scotland: Scottish Family Health Study (GenScot) | McIntosh A; Porteous D | PMID 22786799; 26571028 | analysis code: mdd_GenScot.eur.hg19.SCID_0721a 96

Genetic Epidemiology Research on Aging (GERA) | Schaefer C | PMID 26092718; 26092716 | analysis code: mdd_GERA.eur.hg19.0915a_mds5; GERA_DIV 97

The Trøndelag Health Study (HUNT)| Winsvold B; Hveem K; Zwart JA | PMID 22879362; 30804566; 35578897 | analysis code: mdd_HUNT.eur.hg19.gp_hospital_metacarpa_20190625 100

iPSYCH2012 MDD cohort (iPSYCH2012) | Børglum A; Als T; Mors O; Mortensen PB; Nordentoft M; Werge T; Hougaard DM | PMID 28924187; 29700475 | analysis code: mdd_iPSYCH.eur.hg19.2012_HRC 103

iPSYCH2015i MDD cohort (iPSYCH2015i) | Børglum A; Als T; Mors O; Mortensen PB; Nordentoft M; Werge T; Hougaard DM | 37464041; https://doi.org/10.1101/2020.11.30.20237768 | analysis code: mdd_iPSYCH.eur.hg19.2015i_HRC 106

LifeGene, Karolinska Internet Psychiatry Depression (iCBT) | Pedersen N; Rück C | PMID 30410065 | analysis code: mdd_lgic2.eur.hg19.202011 109

Norwegian Mother, Father and Child Cohort Study (MoBa) | Reichborn-Kjennerud T; Tesli M | PMID 27063603| analysis code: mdd_MoBa.eur.hg19.harvest12, mdd_MoBa.eur.hg19.harvest24, mdd_MoBa.eur.hg19.rotterdam1 111

Million Veteran Program (MVP) | Gelernter J; Levey D | PMID 34045744; 32243820 | analysis code: mdd_MVP.eur.hg19.4_0ICDdep_202106, MVP_DIV 114

Mass General Brigham Biobank (MGBB) | Smoller JW | PMID 26784234 | analysis code: mdd_PBK.eur.hg19.2020 116

PREFECT | Landen M | PMID 33483693 | analysis code: mdd_PREFECT.eur.hg19.run1 117

Scottish Health Research Register (SHARE) | Palmer C; McIntosh AM | PMID 28148535 | analysis code: mdd_SHARE.eur.hg19.godartsshare_842021 118

Swedish Twin Studies of Adults: Genes and Environment (STAGE) | Magnusson P; Lu Y | PMID 23137839; 31747977 | analysis code: mdd_STAGE.eur.hg19.MDDdx_saige 120

Takeda vortioxetine cohort (Takeda) | Wendland JR; Badola S | PMID 25687662; 25575488; 26035185; 26035186 | analysis code: mdd_tkda1.eur.hg19.run1 122

UK Biobank (UKB) - EUR | McIntosh A; Adams M | PMID 30305743 | analysis code: mdd_UKBB.eur.hg19.MD_glm_202012, UKBB_DIV 123

Detroit Neighborhood Health Study (DNHS) | Uddin M | PMID 20439746; 22144187 | analysis code: DNHS 124

Drakenstein Child Health Study (DCHS) | Stein DJ | PMID 25797842 | analysis code: SAFR 126

Jackson Heart Study (JHS) | - | PMID 14632260 | analysis code: JHS 128

Prevention Intervention Research Center (PIRC) 1st Generation Trial (PIRC) | Rabinowitz JA | PMID 24948529 | analysis code: PIRC 129

Women's Health Initiative (WHI) | Wassertheil-Smoller S | PMID 9492970; 14769624; 24572626 | analysis code: WHI_share_AFR, WHI_share_HIS, WHI_DIV 129

BioMe (BioMe) | Loos R | PMID 23588317 | analysis code: BioMe 131

Healthy Life in an Urban Setting (HELIUS) | Verweij KJH; Zwinderman K | PMID 23621920, 29247091 | analysis code: HELIUS 132

Multi-Ethnic Study of Atherosclerosis (MESA) | - | PMID 12397006 | analysis code: MESA 134

Army-STARRS (Army-STARRS) | Ursano RJ; Stein MB | PMID 25338841 | analysis code: Army-STARRS 135

Brazilian High Risk (BHRCS) | Salum G; Pan P; Ota VK; Santoro ML; Oliveira AM; Belangero SI | PMID 25469819 | analysis code: BHRCS 137

Electronic Medical Records and Genomics Network (eMERGE) | - | PMID 25469819 | analysis code: eMERGE 139

Biobank Japan (BBJ) | Okada Y | PMID 28189464; 34594039 | analysis code: BBJ 142

China Kadoorie Biobank (CKB) | Walters RG; Chen Z | PMID 16131516; 37601966 | analysis code: CKB 143

China, Oxford and Virginia Commonwealth University Experimental Research on Genetic Epidemiology cohort (CONVERGE) | Peterson R | PMID 26176920 | analysis code: Converge 145

Taiwan Depression GREAT study | Kuo PH | PMID 36776991; 35321763 | analysis code: Taiwan1; Taiwan2 147

Hispanic Community Health Study / Study of Latinos (HCHS/SOL) | Wassertheil-Smoller S; Dunn E | PMID 20609344; 20609343 | analysis code: HCHS 149

Mexican Adolescent Mental Health Survey (MAMHS) | Cruz-Fuentes C; Martínez-Levy G; Rentería M | PMID 1983355 | analysis code: MAMHS 150

Pregnancy Outcomes, Maternal and Infant Study (PrOMIS) | - | PMID 25620302; 31926482 | analysis code: PrOMIS 150

Genes and Health (GH) | van Heel DA | PMID 31504546 | analysis code: GH 152

QIMR Non-European Cohort | Martin NG; Mitchell BL | 32461290; 34924174; 21042317; 22472876; 26049155 | analysis code: QIMR_DIV 153

Members of contributing groups 155

HUNT All-In Psychiatry 155

Estonian Biobank Research Team 156

Members of the China Kadoorie Biobank collaborative group 156

VA Million Veteran Program: Core Acknowledgement for Publications 157

Conflicts of interest 166

PGC wave 1
Genotype samples

Bonn/Mannheim (BOMA) | Rietschel M | PMID 20673876; 22736164; 16032513; 12177636 | analysis code: boma

Cohort Description:
German psychiatric inpatients (cases) and population-based controls aged 18 years and older were recruited as described below.

Case Ascertainment:
Cases for the Bonn/Mannheim MDD study were ascertained from consecutive admissions to the inpatient units of the Department of Psychiatry and Psychotherapy at the University of Bonn and at the Central Institute of Mental Health in Mannheim, Germany. DSM-IV lifetime diagnoses of MDD were made by a consensus best-estimate procedure based on all available information, including a structured interview (SCID-I), medical records, and the family history method. In addition, the OPCRIT system was used to obtain detailed poly-diagnostic documentation of symptoms. Cases were excluded if they had a lifetime diagnosis of bipolar disorder, hypomania, non-affective psychosis, or depressive symptoms related to a substance use disorder.

Control Ascertainment:
Controls were ascertained from three population-based studies in Germany (Popgen, KORA, and Heinz-Nixdorf-Recall) and included as unscreened controls in the present study.

Genotyping:
Array: Illumina HumanHap550-Quad BeadChip (I550)

Ethics statement:
Study protocols were reviewed and approved in advance by IRBs at participating institutions. Ethics Committee (II) Medical Faculty Mannheim, University of Heidelberg, Department of Psychiatry and Psychotherapy at the University of Bonn; Controls: Ethics Committee of the Medical Faculty of Kiel and by the data protection officer of the University Hospital Schleswig-Holstein (PopGen), Ethics Committee of the Bavarian Medical Association (KORA), Ethics Committee of the Medical Faculty of the University of Essen (Heinz Nixdorf Recall study). All subjects provided written informed consent.

Funding:

**Study**	**Lead investigator**	**Award number**	**Funder**	**Country**
BOMA	M Rietschel	RI 908/11-1	Deutsche Forschungsgemeinschaft	Germany
BOMA	MM Nöthen	NO246/10-1	Deutsche Forschungsgemeinschaft	Germany
BOMA	MM Nöthen	Excellence Cluster ImmunoSensation	Deutsche Forschungsgemeinschaft (DFG)	Germany

BoMa	MM Nöthen, M Rietschel, S Cichon	01ZX1314A/01ZX1614A, 01ZX1314G/01ZX1614G,	BMBF Integrament	Germany
BoMa	MM Nöthen, M Rietschel, S Cichon	01GS08144, 01GS08147	BMBF NGFNplus MooDS	Germany

Edinburgh (UEDIN) | McIntosh AM; Blackwood DHR; Smith D; Adams MJ | PMID 21042317 | analysis code: edi2

Cohort Description:
Cases of MDD were recruited at the same time as individuals with schizophrenia and bipolar disorder from the inpatients and outpatients of the Royal Edinburgh Hospital and associated National Health Service (NHS) facilities.

Case Ascertainment:
Cases were recruited opportunistically from inpatient & outpatient services. Screening for MDD and other conditions was performed using the SADS-L and from structured assessment of medical records. Inclusion criteria: DSM-IV MDD. Exclusion criteria: BIP, NAP, SUD; FHx BIP in a first degree relative.

Control Ascertainment:
Controls were identified from blood donors; which were psychiatrically unscreened. Exclusion criteria included a known current medical or psychiatric diagnosis of medication. Exclusion criteria: Current neurological or psychiatric diagnosis or treatment.

Genotyping:
Array: Affymetrix Genome-Wide Human SNP Array 6.0 (A6.0)

Ethics statement:
The study was approved by the Central Office of Research Ethics Committees in Scotland and all subjects gave informed written consent for the collection of DNA samples for use in genetic studies. Sample collections later received ethical permissions under the project "Family and Population Genetic Studies in Mental Health" Reference 09/MRFE00/81 from Scotland A NHS Research Ethics Committee.

Funding:

Study	Lead investigator	Award number	Funder	Country
EDINBURGH	AM McIntosh	104036/Z/14/Z	Wellcome Trust	UK

Max Planck Institute of Psychiatry (MPIP) | Müller-Myhsok B; Binder E | PMID 19107115 | analysis code: gsk2

Cohort Description:
This sample consisted of German psychiatric in- and outpatients (cases) and population-based controls. Ancestry was estimated using a self-report sheet for nationality, first language, and ethnicity of the subject and of all grandparents. All included patients and controls were of European ancestry.

Case Ascertainment:
All cases were evaluated using the semi-structured Schedules for Clinical Assessment in Neuropsychiatry (SCAN). The SCAN was administered by experienced research assistants who had completed training at World Health Organization Training and Research Centers. Each participant completed a questionnaire covering demography, family and individual history as well as medical history and ancestry. Cases were included in the study if they received a diagnosis of recurrent MDD (≥ 2 separate episodes of MDD) of moderate or severe intensity according to DSM-IV or ICD-10. Cases were excluded from the study if they had experienced mood incongruent psychotic symptoms, a lifetime history of intravenous drug use or diagnosis of drug dependence, depression secondary to alcohol or substance abuse, or depression as clear consequence of medical illnesses or use of medications. Cases with diagnosis of schizophrenia, schizoaffective disorders, and other axis I disorders other than anxiety disorders were excluded from the study.

Control Ascertainment:
Controls were selected randomly from a Munich-based community sample and recruited at the Max-Planck Institute of Psychiatry. They were screened for the presence of anxiety and mood disorders using the CIDI Screener. Only individuals without lifetime mood and anxiety disorders were included as controls.

Genotyping:
Array: Illumina Human Hap550-Quad BeadChip (I550)

Ethics statement:
The study was approved by the Ethics Committee of the Ludwig-Maximilians-University in Munich, Germany.

Funding:
MPIP is funded via in-house institutional funding provided by the Max Planck Society.

RADIANT | Lewis CM; Breen G; Mors O | PMID 20516156 | analysis code: rad3, rage, rai2, rau2, rde4

Cohort Description:
RADIANT cases and controls were ascertained as part of multi-centre studies in depression performed in clinical centres in the UK, Europe, and the US (1 centre).

Case Ascertainment:
Cases for the RADIANT MDD study arise from three multi-center cohorts (DeCC, DeNt, GENDEP) using the same clinical tools. Cases were ascertained from clinical centers in the UK and Europe as well as from one US center. All cases met DSM-IV criteria for MDD after review of all available information by senior study psychiatrists. Cases were interviewed (SCAN and other measures), and consensus diagnoses assigned by research clinicians.

Control Ascertainment:
RADIANT-UK controls were ascertained from the United Kingdom and screened for absence of MDD and mental health disorders using the Past History Schedule.
RADIANT-GER controls consisted of German individuals from the population-based KORA study not overlapping with the ones used for the BOMA study (see separate cohort description).
RADIANT-DEN controls consisted of Danish individuals from the IBP-SGENE Copenhagen (blood bank screening only) with no record of major illnesses or medication use.
RADIANT-Irish controls consisted of Irish ALS GWA controls & opportunistic controls unscreened for psychiatric disorders (PMID 25442119).
RADIANT-US controls consisted of NIMH Clinical Brain Disorders Branch schizophrenia genetics study controls from the United States of America (PMID: 24718902)

Genotyping:

**Subsample**	**Cases/controls**	**Array(s)**
RADIANT-UK (rad3)	Cases	Illumina Human Hap610-Quad BeadChip (I650)
	Controls	Illumina Human Hap610-Quad BeadChip (I650)
RADIANT-DEN (rde4)	Cases	Illumina Human Hap610-Quad BeadChip (I650)
	Controls	
RADIANT-GER (rage)	Cases	Illumina Human Hap610-Quad BeadChip (I650)
	Controls	Illumina Human Hap550-Quad BeadChip (I550)
RADIANT-IRISH (rai2)	Cases	Illumina Human Hap610-Quad BeadChip (I650)
	Controls	Illumina HumanHap550K/610Quad Bead Chips
RADIANT-US (rau2)	Cases	Illumina Human Hap610-Quad BeadChip (I650)
	Controls	Illumina HumanHap550K/610Quad Bead Chips

Ethics statement:
Study protocols were reviewed and approved in advance by IRBs at participating institutions. All subjects provided written informed consent. Ethics permissions were obtained by the South London and Maudsley NHS Trust and Institute of Psychiatry Ethical Committee (Research), 292/03, the South East Multi-centre Research Ethics Committee, MREC (1) 00/19, the South London & Maudsley and IoP Research Ethics Committee, 195/00, and the Institute of Psychiatry Research Ethics Committee, 187/02.

Funding:

**Study**	**Lead investigator**	**Award number**	**Funder**	**Country**

RADIANT-DEN	T Werge	0001-2009-2	Højteknologifonden	Denmark
RADIANT-DEN	T Werge	0001-2009-2	Lundbeck Foundation	Denmark
RADIANT-UK	C Lewis, G Breen	G0701420	MRC	UK
RADIANT-UK	G Breen	G0901245	MRC	UK
RADIANT-UK	G Breen	U01 MH109528	NIMH	UK

Sequenced Treatment Alternatives to Relieve Depression (STAR*D) | Hamilton SP | PMID 20038947 | analysis code: stm2

Cohort Description:
Cases were recruited from participants in the STAR*D trial and controls were Molecular Genetics of Schizophrenia (MGS) subjects as described for GenRED. The STAR*D trial is a large NIMH-sponsored treatment trial involving 4,041 subjects designed to assess the effectiveness of antidepressant treatments in generalizable samples and to investigate outcomes for outpatients with non-psychotic MDD treated with citalopram. To increase the generalizability of the findings, STAR*D utilized broad inclusion criteria and enrolled an ethnically diverse population.

Case Ascertainment:
MDD cases were recruited from participants in the STAR*D trial. Diagnosis was made using the Psychiatric Diagnostic Screening Questionnaire, and depressive symptoms were assessed with the 16-item Quick Inventory of Depressive Symptomatology. Patients with bipolar, psychotic, or obsessive-compulsive disorders were excluded, as were those with primary eating disorders, general medical conditions that contraindicated study medications, substance dependence requiring inpatient detoxification, and clear non-response or intolerance to any protocol antidepressant during current episode or those who were pregnant or breast-feeding.

Control Ascertainment:
An external control sample has been accessed for STAR*D, from the MIGen study of myocardial infarction (PMID: 19198609), accessed through dgGAP (accession # phs000294.v1.p1). https://www.ncbi.nlm.nih.gov/projects/gap/cgi-bin/study.cgi?study_id=phs000294.v1.p1

Genotyping:
Array: Affymetrix Genome-Wide Human SNP Array 6.0 (A6.0)

Ethics statement:
All case subjects signed informed consent for genetic studies. IRB approval was obtained from the institutional review board of the University of California, San Francisco, CA, USA.

Funding:

**Study**	**Lead investigator**	**Award number**	**Funder**	**Country**
STAR*D	SP Hamilton	R01 MH-072802	NIMH	USA

PGC wave 2
Genotype samples

Cognitive Function and Mood Study (CoFaMS) | Baune BT | PMID 27616997 | analysis code: cof3

Cohort Description:
The Cognition and Mood Study (CoFaMS) has the key objective to investigate the clinical, functional, and biological correlates of cognitive dimensions of depression by employing a prospective study design and including a healthy control group. Patients from in- and outpatient settings were recruited between 2015-2018.

Case Ascertainment:
The sampling strategy was opportunistic including inpatients and outpatients. Diagnostic assessment was conducted using SCID and MINI. The main inclusion criteria were a DSM-IV of MDD, age >18 yrs and a current episode of MDD. Exclusion criteria included the diagnoses of BIP, schizophrenia or any other DSM-IV diagnoses as primary; neurological disorders, neurodegenerative disorders such as dementia and MDD related to SUD.

Control Ascertainment:
Controls were recruited from the community through advertisement. Controls were derived from the same community and catchment areas as cases. They were screened for mental disorders using SCID and MINI. Controls were not suitable if they had any life-time psychiatric dx (including MDD).

Genotyping:
Array: Illumina Infinium PsychArray-24 v1.0 (PsychChip)

Ethics statement:
The study was approved by the Human Research Ethics Committees at the University of Adelaide (approval number: H-160-2011) and the Royal Adelaide Hospital Human Research Ethics (approval number: 111230). Study participants were explained all study details in writing and in person before giving informed consent.

Funding:

**Study**	**Lead investigator**	**Award number**	**Funder**	**Country**
CoFaMS - Adelaide	BT Baune	APP1060524	NHMRC	Australia

CoLaus¦PsyCoLaus | Preisig M | PMID 19292899 | analysis code: col3

Cohort Description:
Subjects were selected from individuals of European ancestry from a community survey (CoLaus) carried out in the city of Lausanne, Switzerland. Subjects were randomly selected from a complete list of the Lausanne inhabitants aged 35–75 years. All 35- to 66-year old participants were invited by letters also to participate in the psychiatric evaluation (PsyCoLaus). Sixty-seven percent of the participants of the CoLaus study in the age range between 35–66 years accepted the psychiatric evaluation, which resulted in a sample of 3,719 individuals, of whom 92% were of European ancestry. A subset of European subjects who received full psychiatric assessment and gave consent for genetic testing were selected for genome-wide genotyping. In previous waves of the PGC MDD GWAS, this cohort was referred to as “PsyCoLaus”.

Case Ascertainment:
Psychiatric assessment in the PsyCoLaus sub-study included the semi-structured Diagnostic Interview for Genetic Studies (DIGS), French version. Cases met DSM-IV criteria for MDD.

Control Ascertainment:
Controls were devoid of any psychiatric disorders determined with the DIGS, French version.

Genotyping:
Array: Affymetrix Genome-Wide Human SNP Array 5.0 (A5.0)

Ethics statement:
This research was approved by the Ethics Commission of the Canton of Vaud (www.cer-vd.ch). Reference 16/03; 134-03,134-05bis, 134-05-2to5 addenda 1to4. The approval was renewed for the first (reference 33/09;239/09), the second (reference 26/14; 239/09 addendum 2) and the third (PB_2018-00040; 239/09 addenda 3–4) follow-ups. All participants received a detailed description of the goal and funding of the study and signed a written informed consent.

Funding:

Study	Lead investigator	Award number	Funder	Country
PsyColaus	Martin Preisig	3200B0–105993, 3200B0-118308, 33CSCO-122661, 33CS30-139468, 33CS30-148401, 33CS30_177535	Swiss National Science Foundation	Switzerland
PsyColaus	Julien Vaucher	3247730_204523	Swiss National Science Foundation	Switzerland

PsyColaus	Gérard Waeber, Peter Vollenweider	-	GlaxoSmithKline	USA
PsyColaus	Martin Preisig	-	GlaxoSmithKline	USA
PsyColaus	Peter Vollenweider	-	Faculty of Biology and Medicine of Lausanne	Switzerland
PsyColaus		2018DRI01	Swiss Personalized Health Network	Switzerland

Genetics of Recurrent Early-Onset Depression Phase I (GenRED1) | Levinson D | PMID 12707949; 20125088 | analysis code: gens

Cohort Description:
The GenRED 1 GWAS cohort was designed to study a narrow clinical depression phenotype (recurrent or chronic depression with early age at onset and positive FHx.

Case Ascertainment:
One case subset was recruited for a genome-wide linkage study (one case per family was selected for GWAS but at least one affected relative was directly evaluated) and a second subset was recruited for a case-only cohort with the same inclusion criteria (but with affected relatives evaluated only by family history methods in most cases). Recruitment was opportunistic. Clinical evaluation used the DIGS-3 interview schedule, plus medical records and/or informant report whenever possible. Inclusion criteria: DSM-IV MDD with either 2+ episodes or chronic depression (>3 years' duration); onset < 31 years of age; at least one first-degree relative with recurrent/chronic MDD with onset <41 years. Exclusion criteria: BIP, NAP, mod-severe ID; FHx BIP; if SUD, case was excluded unless at least one MDD onset was preceded by at least 2y of sobriety.

Control Ascertainment:
Controls were drawn from the repository-based Molecular Genetics of Schizophrenia GWAS cohort (MGS, P. Gejman) that was recruited from a large survey research panel using an online clinical screen (based on the CIDI-SF) for psychiatric history. Exclusion criteria: depressed mood or anhedonia for >2 weeks ever; BIP or NAP sx ever; any Dx or Tx for MDD, BIP or NAP.

Genotyping:
Array: Affymetrix Genome-Wide Human SNP Array 6.0 (A6.0)

Ethics statement:
The study was approved by the IRB of each GenRED recruitment site institution (see funding list, below; all controls were recruited by the NorthShore site of the MGS study, see PMID: 19571809). All cases and controls gave written informed consent.

Funding:

**Study**	**Lead investigator**	**Award number**	**Funder**	**Country**
GenRED1 (U Pennsylvania; Stanford University)	D Levinson	5R01-MH-061686	NIMH	USA
GenRED 1 (Columbia University; NY State Psychiatrist Institute)	M Weissman	5R01-MH-060912	NIMH	USA
GenRED 1 (Johns Hopkins University)	JR DePaulo, J Potash	5R01-MH-059552	NIMH	USA
GenRED 1 (University of Iowa)	R Crowe, W Coryell	5R01-MH-059542	NIMH	USA
GenRED1 (Rush University Med Center)	W Scheftner	5R01-MH-059541	NIMH	USA

GenRED1 (University of Pittsburgh)	G Zubenko	5R01-MH-060866	NIMH	USA

Genetic Predictors of Outcome in Depression (GenPod) / Novel Methods leading to New Medications in Depression and Schizophrenia (NEWMEDS) / Welcome Trust Case-Control Consortium (WTCCC) | Uher R | PMID 18498636; 21263010; 23091423; 17554300 | analysis code: gep3

Cohort Description:
Cases were recruited from the multi-center randomized clinical trial "Genetic and clinical predictors of treatment response in depression" (GenPod). The study included patients with MDD recruited in primary care in three centers in the UK (Bristol, Birmingham and Newcastle). Cases from GenPod were genotyped as part of the NEWMEDS project at the University of Geneva Medical School, Geneva, Switzerland. The NEWMEDS project, funded through the European Partnership for Health Innovations in Medicine program of the European Union, is an academia–industry partnership that aimed to discover new drug targets for depression and schizophrenia. NEWMEDS supported the genotyping of multiple cohorts of individuals with MDD and prospectively recorded outcomes of treatment with antidepressants, including GenPod. Control individuals came from the Wellcome Trust Case-Control Consortium (WTCCC) unscreened controls from the UK Blood Bank and 1958 Birth Cohort (NCDS).
Blood-derived DNA samples were genotyped on Illumina Human 660W-Quad bead chips at the University of Geneva Medical School (Geneva, Switzerland) as part of the NEWMEDS project (https://www.imi.europa.eu/projects-results/project-factsheets/newmeds).

Case Ascertainment:
Cases were included if they had an ICD-10 diagnosis of MDD of at least moderate severity as assessed by the Clinical Interview Schedule-Revised (CIS-R) and a Beck Depression Inventory score > 14. A subset of subjects of European ancestry were chosen for genotyping. Individuals were aged between 18–74 years with a mean age of 38.8 years. Exclusion criteria were psychosis, bipolar disorder or major substance or alcohol abuse, and medical contraindications to antidepressants.

Control Ascertainment:
Controls were from the Wellcome Trust Case-Control Consortium, and comprised unscreened controls from the UK Blood Bank and the 1958 Birth Cohort (NCDS), https://www.wtccc.org.uk/ . Genotyping was from an Illumina 1.2M (custom) chip, and access is available through http://www.ebi.ac.uk/ega.

Genotyping:
GenPod array: Ilumina Human Hap610-Quad Bead Chip (I650)
WTCCC array: Illumina 1.2M (custom) chip.

Ethics statement:
Ethical approval for the GenPod study was obtained from the South West Ethics Committee (MREC 02/6/076) as well as research governance approval from Bristol, Manchester and Newcastle Primary Care NHS Trusts. All participants provided written informed consent.
All control participants provided written informed consent.

Funding:

Study	Lead investigator	Award number	Funder	Country
GenPod/Newmeds	G Lewis, M O'Donovan	G0200243	MRC	UK
GenPod/Newm	R Uher	LSHB-CT-2003-	EU 6th	UK

eds		503428	framework	
GenPod/Newmeds	G Lewis	15008	EU IMI-JU	UK
WTCCC	www.wtccc.org.uk	076113, 085475, 090355	Wellcome Trust	UK

Acknowledgments:
This study makes use of data generated by the Wellcome Trust Case-Control Consortium. A full list of the investigators who contributed to the generation of the data is available from www.wtccc.org.uk.

Depression Genes and Networks (DGN) | Levinson D | PMID 24092820 | analysis code: grdg

Cohort Description:
This sample of recurrent MDD cases and controls was recruited for a study of profiles of gene expression in peripheral white blood cells. In PGC MDD Wave 1 this cohort was combined with the GenRED2 cohort (see GenRED2 description) for GWAS analysis as a replication cohort because the two samples were genotyped in the same lab at the same time with the same array. In PGC MDD Wave 2, GenRED2 (with Mayo and GPC controls as described above) and DGN cases/controls were analyzed separately because of ascertainment differences. Here we have continued to analyze them separately.

A survey research company (Knowledge Networks, Menlo Park, CA) recruited prospective recurrent MDD cases and never-depressed controls from participants aged 21–60 from an online survey panel that was recruited on an ongoing basis using random digit dialing of nationally-representative US households. (The same panel was used to recruit the Molecular Genetics of Schizophrenia (MGS) control sample for the NIMH repository, but individuals who were invited to be screened for MGS and who were still members of the panel were not invited to be screened for DGN.) Online screening was carried out using the CIDI-SF depression and alcohol and substance dependence modules. See Supplementary Information for PMID 24092820 for details. Individuals selected as prospective cases or controls were interviewed (SCID) by telephone for DSM-IV diagnosis and final investigator diagnostic review.

Case Ascertainment:
On the online CIDI-SF, prospective cases denied current SUD or lifetime NAP or BIP and endorsed two or more periods of depressed mood and/or anhedonia (or one period of 52 or more weeks), with five total MDD criteria (or four criteria plus either telling a professional or endorsing that depression interfered with functioning). Based on SCID interview and investigator review, individuals selected for inclusion as cases received a final diagnosis of MDD with two or more episodes (or one episode of two or more years) and no lifetime diagnosis of NAP or BIP, and no current substance dependence.

Control Ascertainment:
On online CIDI-SF screening, prospective controls denied ever having two or more weeks of depressed mood or anhedonia (outside of acute bereavement), any history of NAP or BIP, or current substance dependence. Based on SCID interview and investigator review, individuals selected for inclusion as controls continued to meet these same criteria.

Genotyping:
Array: Illumina Omni1-Quad

Ethics statement:
The study protocol was approved by IRBs at Stanford University, Columbia University/NY State Psychiatric Institute, and Johns Hopkins University. Each IRB approved a procedure of obtaining online (digital) consent for clinical screening and (for those selected as prospective cases or controls) to be contacted by the Columbia or Johns Hopkins clinical teams, and then obtaining written informed consent at the time of venipuncture (after having received the consent form by mail, and prior to the telephone SCID interview).

Funding:

**Study**	**Lead investigator**	**Award number**	**Funder**	**Country**
DGN (all sites)	D Levinson	5RC2MH08991	NIMH	USA

		6		

The Netherlands Study of Depression and Anxiety (NESDA) / The Netherlands Twin Register (NTR) | Penninx B; Boomsma D | PMID 33773360; 17254420 | analysis code: nes1

Cohort Description:
The dataset consisted of 3,161 unrelated participants (64.6% females) of North-European ancestry from the Netherlands Study of Depression and Anxiety (NESDA) and from the Netherlands Twin Register (NTR). Detailed descriptions of the rationale, design and methods for both studies are given elsewhere (PMID 33773360, 17254420). Briefly, NESDA is an ongoing cohort study into the long-term course and consequences of depressive and anxiety disorders. In 2004–2007 2,981 participants aged 18 to 65 years were recruited from the community (19%), general practice (54%) and secondary mental health care (27%) and were followed-up during three biannual assessments. The NTR study has been collecting longitudinal data on Dutch twin families since 1991 in over 200,000 young and adult participants (PMID 31666148, 11463154).

Case Ascertainment:
All cases (N=1,527) were drawn from NESDA. Presence of DSM-IV lifetime diagnosis of MDD was assessed using the Composite Interview Diagnostic Instrument (CIDI, version 2.1) administered by specially trained research staff at baseline or one of the biannual follow-up assessments.

Control Ascertainment:
The majority of controls (N=1,320) were drawn from NTR participants who had no reports of MDD, no known first-degree relatives with MDD and a low factor score based on a multivariate analyses of depressive complaints, anxiety, neuroticism and somatic anxiety (PMID 18197199). From NESDA, 314 healthy controls were also selected, consisting of participants without any lifetime psychiatric disorder.

Genotyping:
Array: Affymetrix Genome-Wide Human SNP Array 6.0 (A6.0)

Ethics statement:
The NESDA and NTR studies were approved by the Central Ethics Committee on Research Involving Human Subjects of the VU University Medical Center, Amsterdam, an Institutional Review Board certified by the US Office of Human Research Protections (IRB number IRB-2991 under Federal wide Assurance-3703; IRB/institute codes, NESDA 03-183; NTR 03-180). All subjects provided written informed consent on forms approved by the IRB at the PI's respective institutions.

Funding:

**Study**	**Lead investigator**	**Award number**	**Funder**	**Country**
NESDA	Penninx BWJH	ZonMW Geestkracht grant 10-000-1002	N.W.O.	Netherlands
NTR	Boomsma DI	480-04-004; 480-15-001/674	N.W.O.	Netherlands
NESDA<br>NTR	Penninx BWJH<br>Boomsma DI	R01D0042157-01A,	NIH	USA

		MH081802, Grand Opportunity grants 1RC2 MH089951 and 1RC2 MH089995		

Acknowledgement:
NESDA: Funding for the infrastructure of the NESDA study (www.nesda.nl) was obtained from the Netherlands Organization for Scientific Research (Geestkracht program grant 10-000-1002); the Center for Medical Systems Biology (CSMB, NWO Genomics), Biobanking and Biomolecular Resources Research Infrastructure (BBMRI-NL), VU University's Institutes for Health and Care Research (EMGO+) and Neuroscience Campus Amsterdam, University Medical Center Groningen, Leiden University Medical Center, National Institutes of Health (NIH, R01D0042157-01A, MH081802, Grand Opportunity grants 1RC2 MH089951 and 1RC2 MH089995). Part of the genotyping and analyses were funded by the Genetic Association Information Network (GAIN) of the Foundation for the National Institutes of Health.Computing was supported by BiG Grid, the Dutch e-Science Grid, which is financially supported by NWO.
NTR: We gratefully acknowledge funding from the Netherlands Organization for Scientific Research (NWO) and MagW/ZonMW grants 904-61-090, 985-10-002, 912-10-020, 904-61-193,480-04-004, 463-06-001, 451-04-034, 400-05-717, Addiction-31160008, Middelgroot-911-09-032, Spinozapremie 56-464-14192, Center for Medical Systems Biology (CSMB, NWO Genomics), Biobanking and Biomolecular Resources Research Infrastructure (BBMRI –NL, 184.021.007 and 184.033.111); the KNAW Academy Professor Award (PAH/6635) ; the European Community's Seventh Framework Program (FP7/2007-2013), ENGAGE (HEALTH-F4-2007-201413); the European Science Council (ERC Advanced, 230374), Rutgers University Cell and DNA Repository (NIMH U24 MH068457-06), the Avera Institute, Sioux Falls, South Dakota (USA) and the National Institutes of Health (NIH, R01D0042157-01A, MH081802, Grand Opportunity grants 1RC2 MH089951 and 1RC2 MH089995) and the Genetic Association Information Network (GAIN) of the Foundation for the National Institutes of Health.

Genetics of Recurrent Early-Onset Depression Phase II (GenRED2) | Levinson D | PMID 29700475 | analysis code: grnd

Cohort Description:

See the description of GenRED1 for further details. This cohort included the second wave of recruitment for the case-only phase of the GenRED study. The cohort was limited to cases with recurrent or chronic MDD with early onset and positive FHx. It has not been published separately from the PGC GWAS by Wray et al. (PMID 29700475).

The second phase of GenRED was included as a replication sample (the first phase was a discovery sample). GenRED2 included new cases meeting the same criteria as in GenRED1 (see GenRED1 sample case ascertainment), plus new controls. Dr. Janet Sobell (University of Southern California) contributed post-QC controls from the Mayo DNA Bank which consists of long-term, community medicine patients (Mayo Clinic, Rochester, MN) who were undergoing venipuncture for any reason. Consenting individuals aged 45 years and older completed a brief demographic and psychiatric screening questionnaire. Extensive medical records were screened for evidence of psychiatric illness. Individuals were excluded if they were judged likely to have had a mood or psychotic disorder on the basis of a review of medical records, taking into account the recorded diagnoses and treatment in each case (including major disorders as well as possible proxies for a mood disorder in older nomenclature such as adjustment disorders, depression NOS, anxiety state, etc.). The final subset was selected to roughly match the proportions of self-reported ancestry in the GenRED 1+2 sample.

Note that these 1,305 subjects were genotyped at the same lab (Centrillion Biosciences, Mountain View, CA) and with the same GWAS array (Illumina Omni1-Quad) as the Depression Genes and Networks (DGN) sample collected by a subset of the GenRED investigators, so that these samples were combined for the PGC replication analysis.

Case Ascertainment:

(See GenRED1 case ascertainment. Affective relatives were evaluated by family history methods only in most cases.)

Control Ascertainment:

One subset of controls was contributed by Dr. Janet Sobell (University of Southern California) from the Mayo DNA Bank (PMID: 8357034). Individuals were recruited from long-term, community medicine patients who completed a brief demographic and psychiatric screening questionnaire, and for whom medical records were screened for evidence of psychiatric illness. Individuals were excluded if they were judged to have possibly had a mood or psychotic disorder based on diagnostic terms including older proxies for depression such as adjustment disorders, depression NOS, anxiety state, etc.). The second control subset was contributed by Drs. Carlos Pato and James Knowles from the Genomic Psychiatry Cohort (PMID: 23650244) who were screened for psychiatric history with a self-report questionnaire and who answered negatively to five screening questions for lifetime bipolar disorder, five for lifetime schizophrenia, and four for lifetime MDD.

Genotyping:

Array: Illumina Omni1-Quad (OMEX)

Ethics statement:

For the entire GenRED study, see the statement for GenRED1. Mayo Clinic and GPC control individuals gave informed consent under protocols approved by the IRB of the collecting site as described in the references provided above.

Funding:

See the funding list for GenRED1 except that the University of Pittsburgh was not a collection site for GenRED2.

Harvard i2b2 (i2b2-TRD) | Perlis RH; Smoller JW | PMID 22472876 | analysis code: i2b3

Cohort Description:
Cases were identified using the Partners Healthcare electronic medical record (EMR), and controls were recruited from a healthy volunteer sample (MGH Brain Genomics Superstruct Project).

Case Ascertainment:
Using data available in the EMR, patients who met the following inclusion criteria were identified: a minimum of one ICD-9 code for MDD (ICD-9 296.2x, 296.3x), at least one antidepressant prescription, and at least three outpatient psychiatry visit notes. Patients with both a past antidepressant prescription and at least two electroconvulsive therapy procedures (CPT: 90870, 90871) within one week were also included. Exclusion criteria included a past history of bipolar disorder (ICD-9: 296.1x, 296.4x-296.9x), dementia (ICD-9: 290.x), psychotic disorder (ICD-9: 295.x, 298.x), or past bone marrow transplant. De-identified, discarded blood specimens were obtained for subjects meeting these criteria who had presented for routine blood draw. Genotyping was restricted to individuals who identified as having European ancestry.

Control Ascertainment:
Controls were identified from a healthy volunteer sample, aged 18–35, participating in the MGH Brain Genomics Superstruct Project and were screened to have no history of psychiatric or neurologic illness or treatment with psychoactive drugs.

Genotyping:
Array: Illumina OmniExpress BeadChip (OMEX)

Ethics statement:
The Partners Institutional Review Board approved all aspects of this study.

Funding:

Study	Lead investigator	Award number	Funder	Country

Munich Antidepressant Response Signature (MARS) | Lucae S | PMID 21521612; 19736353; 18586274 | analysis code: mmi2, mmo4

[Berger DHS controls as separate section below]

Cohort Description:
Naturalistic study with inpatients suffering from Major depression from the Max-Planck-Institute of Psychiatry in Munich, Germany, and 3 other Bavarian psychiatric hospitals. It was the purpose of the study to investigate the pharmacogenetics of depression.

Case Ascertainment:
Inpatients; diagnosis by psychiatrist. Clinical interview, MINI, & rater (SAFER & ATRQ). Inclusion criteria: DSM-IV MDD. Exclusion criteria: BIP, SUD, secondary MD, severe medical conditions.

Control Ascertainment:
No controls included. [Berger DHS controls as separate section below]

Genotyping:
mmi2 array: Illumina Human Hap610-Quad Bead Chip (I650)
mmo4 array: Illumina OmniExpress BeadChip (OMEX)

Ethics statement:
Ethics Committee of the Ludwig-Maximilians-University in Munich; Ref.Nr. 318/00

Funding:
The MARS project and genome-wide genotyping was supported by the Bavarian Ministry of Commerce and by the Excellence Foundation for the Advancement of the Max Planck Society.

Dortmund Health Study Controls (DHS) | Berger K | PMID 18677645 | analysis code: mmo4

Cohort Description:
DHS provided controls for the cases in the MARS study described above. Participants in DHS were randomly selected from the city registry of Dortmund, Germany from the age range 25–74 years and invited to participate. The overall aim was to analyze the prevalence and

incidence of different headache types and other chronic diseases in the general population and to evaluate consequences on daily activities of those affected.

Case Ascertainment:
No cases included.

Control Ascertainment:
The CES-D was used in all DHS participants and different lifetime diagnoses by a physician assessed in a medical interview. A CES-D summary score of <10 plus no lifetime diagnosis of depression were used to include DHS controls.

Genotyping:
Array: Illumina HumanOmni2.5-4v1_D.bpm

Ethics statement:
The joint ethics committee of the Westphalian Chamber of Physicians and the University of Muenster approved the study (3VIIBerger).

Funding:
DHS was funded by unrestricted grants of equal share to the University of Muenster, Germany, from the German Migraine and Headache Society and Allmiral, Astra-Zeneca, Berlin-Chemie, Boehringer Ingelheim Pharma, Boots Healthcare, GlaxoSmithKline, Janssen Cilag, McNeil Pharmaceuticals, MSD Sharp & Dohme, and Pfizer.

Pfizer | Paciga SA | PMID 23091423 | analysis code: pfm2

Cohort Description:
The cohort included subjects with moderate to severe major depressive disorder, having participated in one of several clinical trials for compounds under clinical development at Pfizer.

Case Ascertainment:
Clinical trial participants in one of several major depressive disorder clinical trials. MDD patients were ascertained using either the SCID or MINI and scored >21 on the Hamilton Rating Scale for Depression. MDD patients met the criteria for DSM-IV MDD. Exclusion criteria were a diagnosis of bipolar disorder, non-affective psychotic disorder, or substance use disorder.

Control Ascertainment:
Controls consisted of unscreened participants from a genome-wide association study of melanoma.

Genotyping:
Array: Illumina HumanHap550-Quad BeadChip (I550)

Ethics statement:
Samples were collected from participants in one of several clinical trials (A050R-0552, A1650112, A1651002, A1651008, A2721002, A2721008, M20200046, M20200047), for which IRB / EC approval was obtained.

Funding:
Pfizer provided the funding for the clinical trials.

QIMR Berghofer Medical Research Institute (QIMR) | Martin NG | PMID 21042317 | analysis code: qi3c, qi6c, qio2

Cohort Descriptions:
QIMR Berghofer has contributed three cohorts to previous PGC-MDD waves. PGC-MDD wave 1 included two QIMR cohorts, qi3c (MDD2000-QIMR_317) and qi6c (MDD2000-QIMR_610), while the third cohort, qio2 (MDD2000-QIMR_COEX), was introduced in wave 2. All three cohorts were used in this study.

QIMR cohorts were analyzed as three sets qi3c (those genotyped on the Illumina 317K or 370K platforms, denoted MDD200-QIMR_317 in wave 1), qi6c (those genotyped on the Illumina 610K platform, MDD2000-QIMR_610 in wave 1), and qio2 (first contributed to wave 2) genotyped on the Infinium CoreExome, OmniExpress or Omni 2.5 array platforms. Across all three cohorts, cases and controls were drawn from five studies at QIMR: NC/IRPG (Twins and Spouses), SS1 (SSAGA1), TE (Twin89) and AX (Anxiety and Depression).

Case Ascertainment:
QIMR cases were from the population-based Australian Twin Registry. All subjects are of European ancestry. MDD cases and controls were identified through psychiatric questionnaires administered to adult twins and their families recruited through the Australian Twin Registry (ATR, http://www.twins.org.au) under collaborative grant funding to QIMR and the Washington University in St. Louis. The structured interviews were either the shortened Composite International Diagnostic Interview (CIDI) or the SSAGA-OZ interview (a modified version of the Semi-Structured Assessment for the Genetics of Alcoholism), comprehensive psychiatric interviews designed to assess MDD and other psychiatric disorders according to DSM-IV criteria. The interviews were administered by trained telephone interviewers, closely supervised by a clinical psychologist. Only unrelated individuals were included. Particular attention was paid to exclude cases with bipolar spectrum disorders.

Control Ascertainment:
Unrelated controls were selected as genotyped individuals from families in which no individuals qualified for diagnoses of MDD, anxiety disorders or alcohol abuse across QIMR cohorts. If multiple controls were available from a family, the individual with the lowest neuroticism score was preferentially selected, otherwise an individual was selected at random.

Sample Sizes:
Overall, qi3c contained 864 cases and 579 controls while qi6c had 499 cases and 590 controls. The qio2 included 565 cases and 525 controls.

Genotyping:
qi3c (MDD2000-QIMR_317): Illumina 317K or 370K platforms (I317)
qi6c (MDD2000-QIMR_610): Illumina 610K platform (I650)
qio2 (MDD2000-QIMR_COEX): OmniExpress, Omni 2.5 or Infinium CoreExome platforms.

Ethics statement:
Patient consent allows de-identified samples to be sent abroad and used by academic and commercial collaborators and phenotype and genotype data can be used in meta-analyses. All participants provided written informed consent under study protocols approved by the QIMR Human Research Ethics Committee. Ethics approval identifiers for the QIMR studies used to derive cases and controls used across these three cohorts are as follows: P2148, P2162 (NC/IRPG), 34/92 (SSI), P329 (TE), and P642 (AX).

Funding:

Study	Lead investigator	Award number	Funder	Country

qio2	NG Martin	941177, 971232, 3399450 and 443011	National Health and Medical Research Council	Australia
qi3c, qi6c, qio2	AC Heath	AA07535, AA07728, andAA10249	NIAAA	USA
qi3c, qi6c	NG Martin	241944,339462, 389927,389875, 389891,389892, 389938,442915, 442981,496675, 496739,552485, 552498, 613608	National Health and Medical Research Council	Australia

Roche | Domenici E | PMID 27304433; 32663909 | analysis code: roc3

Cohort Description:
Cases were recruited from two randomized, double-blind, placebo-controlled clinical trials (MARIGOLD and ARTDeCo) for the investigation of the safety and efficacy of novel antidepressants as adjunctive therapy in patients with major depressive disorder having inadequate response to ongoing antidepressant treatment. Controls were recruited from a screening study to assess the cognition status in healthy volunteers

Case Ascertainment:
Inclusion criteria: Primary diagnosis of MDD without psychotic features as defined by DSM-IV-TR, on the basis of a structured interview (MINI or SCID-CT); inadequate response to ongoing antidepressant treatment, as defined by protocol. Exclusion criteria: present DSM-IV-TR axis I diagnosis except for anxiety comorbidity; past or present psychotic symptoms, substance use/abuse/dependence, suicidal risk, neurological disorders. Roche cases are described in https://www.clinicaltrials.gov/ct2/show/NCT01457677 and https://clinicaltrials.gov/ct2/show/NCT01437657.

Control Ascertainment:
Opportunistic, healthy volunteers matched to cases (ancestry, education, age, sex). Exclusion criteria: Current or past history of a psychiatric disorder, family history of psychiatric disorders, suspicion or evidence of regular consumption of drugs of abuse or a positive drug test at the screening visit. The controls are described in https://clinicaltrials.gov/ct2/show/NCT01926873.

Genotyping:
Array: Illumina Infinium PsychArray-24 (PsychChip)

Ethics statement:
The institutional review board or ethics committee approved the protocol at each site of the multi-centre clinical trials. For details, see the registered trial descriptions on ClinicalTrials.gov.

Funding:
Investigation sponsor: Hoffman-La Roche

**Study**	**Lead investigator**	**Award number**	**Funder**	**Country**
MARIGOLD	Jorge Quiroz	-	Hoffman-La Roche	International (multi-centre clinical trial)
ARTDeCo	Daniel Umbricht	-	Hoffman-La Roche	International (multi-centre clinical trial)

Rotterdam Study (Rotterdam) | Tiemeier H | PMID 19047526 | analysis code: rot4

Cohort Description:
The design of the Rotterdam Study is that of a prospective cohort study among, initially, 7,983 persons living in the well-defined Ommoord district in the city of Rotterdam in The Netherlands (78% of 10,215 invitees). They were all 55 years of age or over, and the oldest participant at the start was 106 years. From January 1990 onwards, participants were recruited for the Rotterdam Study. In 2000, 3,011 participants (out of 4,472 invitees) who had become 55 years of age or moved into the study district since the start of the study were added to the cohort. In 2006 a further extension of the cohort was initiated in which 3,932 subjects were included, aged 45–54 years, out of 6,057 invited, living in the Ommoord district. By the end of 2008, the Rotterdam Study therefore comprised 14,926 subjects aged 45 years or over.

Case Ascertainment:
During the follow-up rounds, we used a two-step procedure to assess whether participants were going through a depressive episode. First, all participants were screened with the CES-D as part of the home interview. The screen-positive participants (score>=16) were invited for a clinical interview. A psychiatrist or clinical psychologist, each with extensive clinical experience, conducted the interview using the Dutch version of the Present State Examination. This is a semi-structured psychiatric interview included in the Schedules for Clinical Assessment in Neuropsychiatry. Scoring of items is conservative and relies on clinical judgment instead of the participant's answers only. With a computerized diagnostic algorithm based on the item scores, major depressive disorders were classified according to DSM-IV criteria. Exclusion criteria were a diagnosis of bipolar disorder, non-affective psychotic disorder, and depression related to substance use.

Control Ascertainment:
Exclusion criteria for controls were depressive symptoms above the CES-D clinical cut-off of 16 in any of the assessment waves.

Genotyping:
Array: Illumina HumanHap550-Quad BeadChip (I550)

Ethics statement:
The Rotterdam Study has been approved by the Medical Ethics Committee of the Erasmus MC (registration number MEC 02.1015) and by the Dutch Ministry of Health, Welfare and Sport (Population Screening Act WBO, license number 1071272-159521-PG). The Rotterdam Study Personal Registration Data collection is filed with the Erasmus MC Data Protection Officer under registration number EMC1712001.

Funding:

**Study**	**Lead investigator**	**Award number**	**Funder**	**Country**
Rotterdam Study	AG Uitterlinden	175.010.2005.011, 911-03-012	Netherlands Organization of Scientific Research NWO Investments	Netherlands
Rotterdam Study	Henning Tiemeier	016.VICI.170.200	Netherlands Organisation for Health	Netherlands

			Research and Development	

Study of Health in Pomerania - Life-Events and Gene-Environment Interaction in Depression (SHIP-LEGEND) | Grabe HJ; Völzke H | PMID 25077849; 20167617; 35348705 | analysis code: shp0

Cohort Description:
Data from the Study of Health in Pomerania (SHIP) were used. The target population of the SHIP-START cohort comprised all adult German residents in northeastern Germany living in 3 cities and 29 communities, with a total population of 212,157. A two-stage stratified cluster sample of adults aged 20–79 years (baseline) was randomly drawn from local population registries. The net sample (without migrated or deceased persons) comprised 6,265 eligible subjects of whom 4,308 Caucasian subjects participated at baseline (SHIP-START-0) between 1997 and 2001. Follow-up examination (SHIP-START-1) was conducted 5 years after baseline.

Between 2007 and 2010, the "Life-Events and Gene-Environment Interaction in Depression" (LEGEND) study was started based on participants from SHIP-START-0. Of 3,669 subjects from SHIP-START-0 that were invited, 2,400 agreed to participate.

Case Ascertainment:
The lifetime diagnosis of MDD was assessed with the Munich-Composite International Diagnostic Interview (M-CIDI). The M-CIDI is a standardized fully structured instrument for assessing psychiatric disorders over the life span according to DSM-IV criteria. The computerized version of the interview was used by clinically experienced interviewers (psychologists) in a face-to-face situation. Subjects with bipolar disorder or MDD related to suicide were excluded. All interviewers had undergone intensive and continuous training in the diagnostic procedures.

Control Ascertainment:
Control subjects were also taken from the LEGEND sample excluding those subjects that fulfilled the diagnosis of MDD.

Genotyping:
Array: Affymetrix Genome-Wide Human SNP Array 6.0 (A6.0)

Ethics statement:
Written informed consents were obtained from all participants according to the principles of the Declaration of Helsinki. The studies were approved by the Ethics Committee at the University Medicine Greifswald, Germany (approval number BB 39/08)

Funding:

Study	Lead investigator	Award number	Funder	Country
SHIP-LEGEND/TREND	HJ Grabe	DFG: GR 1912/5-1	German Research Foundation	Germany
SHIP-START/TREND	H Völzke	BMBF: grants no. 01ZZ9603, 01ZZ0103, and 01ZZ0403	Federal Ministry of Education and Research	Germany

SHIP-TREND | Grabe HJ; Völzke H | PMID 20167617; 35348705 | analysis code: shpt

Cohort Description:

A separate stratified random sample of adults aged 20–79 years was drawn for the Study of Health in Pomerania - TREND baseline cohort (SHIP-TREND-0) between 2008 and 2011. Sample selection was facilitated by centralization of local population registries in the Federal State of Mecklenburg/West Pomerania. Stratification variables are age, sex and city/county of residence. The target sample size was chosen to obtain a final sample size similar to that of SHIP-START-0. The study comprised 8,016 eligible subjects of whom 4,428 Caucasian subjects participated at baseline SHIP-TREND-0. Examinations were similar to SHIP-START.

Case Ascertainment:
Lifetime diagnosis of MDD was assessed with the M-CIDI that typifies the key diagnostic criteria of MDD from the Diagnostic and Statistical Manual of Mental Disorders IV (DSM-IV). Subjects with bipolar disorder or MDD related to suicide were excluded. As this was general population subjects with bipolar disorder were not included.

Control Ascertainment:
Control subjects were also taken from the SHIP-TREND-0 sample excluding those subjects that fulfilled the diagnosis of MDD.

Genotyping:
Array: Illumina HumanOmni 2.5-Quad

Ethics statement:
Written informed consents were obtained from all participants according to the principles of the Declaration of Helsinki. The studies were approved by the Ethics Committee at the University Medicine Greifswald, Germany (approval number BB 39/08).

Funding:

**Study**	**Lead investigator**	**Award number**	**Funder**	**Country**
SHIP-START/TREND	H Völzke	BMBF: grants no. 01ZZ9603, 01ZZ0103, and 01ZZ0403	Federal Ministry of Education and Research	Germany
SHIP-TREND	H Völzke	BMBF: grants no. 03ZIK012	Federal Ministry of Education and Research	Germany

TwinGene | Kendler K; Magnusson P | PMID 16390897, 23137839 | analysis code: twg2

Cohort Description:
The TwinGene project, conducted between 2004 and 2008, is a population-based Swedish study of twins born between 1911 and 1958 drawn from the Swedish Twin Registry. All subjects in this investigation were independent (i.e., one twin selected per twin pair). The study participants previously participated in a telephone interview called Screening Across the Lifespan Twin Study (SALT), conducted between 1998 and 2002. For the purpose of the current analyses, we kept unrelated individuals (one twin per pair).

Case Ascertainment:
Data from the SALT study were used to identify MDD cases as either reporting antidepressant use or meeting DSM-IV criteria for MDD using the Composite International Diagnostic Interview Short Form (CIDI-SF) criteria. Twingene cases were defined by the presence of MDD using any of the following criteria: having any lifetime diagnosis of MDD (defined by ICD8: 296, 300.4; ICD-9-SWE: 296B, 311; ICD-10 F32, F33 from national patient register) at inpatient care; having any lifetime diagnosis of MDD at outpatient care and prescribed at least one antidepressant (using ATC code N06A from prescription drug register); meeting DSM-IV criteria for MDD using the Composite International Diagnostic Interview Short Form (CIDI-SF; with symptoms scores ≥ 4 of 8) and prescribed at least one antidepressant; meeting DSM-IV criteria for recurrent MDD using CIDI-SF (episodes > 1). We further excluded bipolar disorder from these MDD cases.

Control Ascertainment:
Controls were selected as those who had been screened negative using CIDI-SF and had no record of lifetime MDD diagnosis from inpatient- or outpatient specialist care, and had no prescription record of antidepressants. We also excluded controls with bipolar disorder.

Genotyping:
Array: Illumina OmniExpress BeadChip (OMEX)

Ethics statement:
The study was approved by the Stockholm local ethics committee (DNR: 2007-644-32, 2011/463-32) and all participants gave informed consent

Funding:

Study	Lead investigator	Award number	Funder	Country
Swedish Twin Registry (TwinGene is a substudy)	P Magnusson	2017-00641	Swedish Research Council	Sweden
TwinGene	N Pedersen	EU/QLRT-2001-01254; QLG2-CT-2002-01254	GenomeEUtwin	EU
TwinGene	P Magnusson	20070481	Heart and Lung foundation	Sweden

TwinGene	U de Faire	-	SSF	Sweden
TwinGene	U de Faire	M-2005-1112	Vetenskapsrådet	Sweden

PGC wave 3
Genotype samples

Janssen | Li QS | PMID 16760927; 26707087; 28086004 | analysis code: janpy

Cohort Description:

The Janssen MDD case cohort included unrelated patients with major depressive disorder from 3 clinical trials [N=545, NCT00044681, NCT01640080, and NCT01627782, genotyped using HumanOmni5Exome (Illumina, Inc., San Diego, CA), this was included in PGC wave2 MDD analysis but matched to different control samples in wave 2] and 2 additional clinical studies [N = 291, NCT00566202 and NCT00073203, genotyped using PsychArray, new for wave 3] conducted by Janssen Research & Development, LLC.

The study inclusion/exclusion criteria have been described in detail in prior publications and/or ClinicalTrials.gov. Briefly, all patients met DSM-IV criteria of Major Depressive Disorder. Genetic sampling is either optional or mandatory. Healthy control samples from NINDS Human Genetics Repository (neurologically normal Caucasian control panel NDPT020, NDPT079, NDPT084, NDPT090, NDPT093, NDPT094, NDPT095, NDPT096, NDPT098, and NDPT099, new to wave 3) managed by Coriell Institute for Medical Research (Camden, NJ) were genotyped using PsychArray. Only samples of European ancestry with matching controls were included in this study. Overlapping SNPs between the two arrays were used in the imputation.

Case Ascertainment:

**ClinicalTrials.gov ID**	**N**	**Inclusion Criteria**	**Genotyping Array**	**Status**	**Publication (PMID)**
RIS-INT-93 / NCT00044681	458	Meet criteria of Diagnostic and Statistical Manual of Mental Diseases, 4th edition (DSM-IV), for Major Depressive Disorder with or without psychotic features; history of resistance to therapy with antidepressant medication	HumanOmni5Exome	Case	Rapaport et al., 2006 (16760927)
ESKETIV-TRD-2001 / NCT01640080	26	Meet Diagnostic and Statistical Manual of Mental Disorders diagnostic criteria for recurrent MDD **without psychotic features** (**DSM-IV**, 296.32, or 296.33), based upon clinical assessment and confirmed by the Mini International Psychiatric Interview (MINI); Have an inadequate response to at least 1 antidepressant in the current episode of depression and at least one other inadequate treatment response to an antidepressant either in the current episode or in a previous episode	HumanOmni5Exome	Case	Singh et al., 2015 (26707087)

KETIV-TRD-2002 / NCT01627782	61	Meet Diagnostic and Statistical Manual of Mental Disorders diagnostic criteria for recurrent MDD **without psychotic features** (**DSM-IV**, 296.32, or 296.33), based upon clinical assessment and confirmed by the Mini International Psychiatric Interview (MINI); Have an inadequate response to at least 1 antidepressant in the current episode of depression and at least one other inadequate treatment response to an antidepressant either in the current episode or in a previous episode	HumanOmni5Exome	Case	Johnson et al., 2016 (28086004)
R228060-MDD-201/NCT00073203	254	Study subjects were adult outpatients, 18-65 years of age, inclusive, who met Diagnostic and Statistical Manual of Mental Disorders, 4th Edition, (**DSM-IV**) criteria for moderate or severe major depression **without psychotic features**. Subjects were also required to meet severity criteria of a) at least 23 on the Carroll Depression Scale-Revised (CDS-R), and b) at least 4 on the Clinical Global Impressions of Severity scale (CGI-S), both at screening and at baseline.	PsychArray	Case	
C-2007-007/ NCT00566202	37	Subjects must have a confirmation of a diagnosis of MDD, moderate or severe, at Screening by M.I.N.I. and must have fulfilled **DSM-IV**-TR criteria for MDD **without psychotic features** for greater than 4 weeks and less than 12 months in duration at the Baseline Visit (Visit 2).	PsychArray	Case	
Coriell Controls	926		PsychArray	Control	

Control Ascertainment:
Healthy control samples from NINDS Human Genetics Repository (neurologically normal Caucasian control panel NDPT020, NDPT079, NDPT084, NDPT090, NDPT093, NDPT094, NDPT095, NDPT096, NDPT098, and NDPT099) managed by Coriell Institute for Medical Research (Camden, NJ).

Genotyping:
Arrays: Illumina Omni5MExome BeadChip, PsychArray (PsychChip)

Ethics statement:

All patients providing genetic samples consented to both the main study ICF and genetic testing specific ICF (in the case where participation in genetic study was optional).

Funding:

The studies were funded by Janssen Research & Development, LLC.

Ziekenhuisnetwork Antwerpen (ZNA) | Luykx J | PMID 35896057; 35649338 | analysis code: antpo

Cohort Description:
The goal of this study was to collect patients with varying severe, psychiatric disorders and take their EEG and DNA to run analyses on PRS of EEG in them as well as in healthy controls. Therefore we only included participants admitted for their psychiatric condition and no outpatients, as well as healthy controls.

Case Ascertainment:
Participants were collected from September 2013 until September 2018 at Ziekenhuis Netwerk Antwerpen (ZNA), a large community hospital in Antwerp, Belgium. The study was approved by the Institutional Review Board of ZNA.

In this study, a total of 1,195 adult participants – 1,132 psychiatric patients with various(predominantly mood, psychotic and/or substance use) disorders and 63 healthy controls to obtain a heterogenous sample – were ascertained based on clinician judgment by DSM-IV-TR criteria. Exclusion criteria for all participants were age <18 years, inability to give informed consent for whatever reason, and restlessness that could interfere with EEG measurements.

Control Ascertainment:
Healthy controls were defined as having no current psychiatric episode and never been treated by a mental health service.

Genotyping
Array: Global Screening Array v1.0 (GSA1)

Ethics statement:
We abided by the principles of the Declaration of Helsinki. All participants provided written informed consent.

Funding:

**Study**	**Lead investigator**	**Award number**	**Funder**	**Country**
ZNA	Jurjen J. Luykx	H150	Rudolf Magnus Fellowship; University Medical Center Utrecht	Netherlands

Pre-, Peri- and POstnatal Stress: Epigenetic Impact on DepressiON (POSEIDON) | Rietschel M; Deuschle M; Streit F; Witt S | PMID 30471571 | analysis code: antpo

Cohort Description:
Parents of the families participating in the longitudinal study POSEIDON (Pre-, Peri-, and Postnatal Stress: Epigenetic impact on Depression) for whom genetic data was available. In total, pregnant women were recruited from hospitals in the Rhine-Neckar Region of Germany, about 4–8 weeks prior to delivery. Data was collected during the third trimester of pregnancy (T1), a few days after childbirth (T2), six months postpartum (T3) and 45 months postpartum (T4). The last assessment (T4) took place between August 2014 and January 2017. At T4, dropouts were replaced by families with children of the same age as the original POSEIDON cohort. For this purpose, information about eligible new participants was obtained from the local registries of residents (birth year of the child, parents' names and address). The eligible new participants were informed about the study by mail.

Case Ascertainment:
No cases included.

Control Ascertainment:
Mothers were contacted during pregnancy at the hospital. The subjects replacing dropouts at the last wave of the study were contacted via local registries. Maternal inclusion criteria: 16–45 years old, German-speaking and presumably the child's main caregiver. Maternal exclusion criteria: positive for hepatitis B, hepatitis C or human immunodeficiency virus, current psychiatric disorder requiring inpatient treatment, history or current diagnosis of schizophrenia or psychotic disorder, substance dependency other than nicotine during pregnancy. For the purpose of serving as a control sample for the PGC-MDD group, subjects reporting current MDD or a history of MDD at any of the assessment time points were excluded.

Genotyping
Array: Global Screening Array GSAMD-24v3-0 (GSA3)

Ethics statement:
All participants provided written informed consent before they participated in the study. The study was approved by the institutional research committee (Ethics Committee of the Medical Faculty Mannheim of the University of Heidelberg, reference number 2014-550N-MA).

Funding:

Study	Lead investigator	Award number	Funder	Country
POSEIDON	MR	01EW1810<br>01EW1904	German Federal Ministry of Education and Research (BMBF)	Germany
POSEIDON	MD	-	Dietmar-Hopp Foundation	Germany

Study “Establishing the bidirectional link between subclinical arteriosclerosis and depression” (BiDirect) | Berger K | PMID 28348383 | analysis code: bidi

Cohort Description:

The BiDirect Study examines the association between depression and subclinical arteriosclerosis. Patients hospitalized for an acute episode of depression in one of five psychiatric hospitals in the district of Muenster (Germany), a random sample of the inhabitants of the city of Münster, and patients 2–4 months after an acute coronary syndrome recruited in cardiac rehabilitation facilities in the same area, were recruited into three cohorts, examined in parallel with identical methods. All participants in the three cohorts were aged 35 to 65 years at baseline and overall 2258 individuals were included. The examination program included a medical interview on socioeconomic factors, comorbidities and medication, a depression classification including subtypes, a vascular diagnostic work-up, anthropometric measurements, a neuro-psychological test battery and a magnetic resonance imaging (MRI) of the brain. A battery of interview-based or self-administered questions and scales (e.g., depressive and anxiety symptoms from the M.I.N.I. vs5.0.0, childhood trauma, personality, resilience, and quality of life) was additionally assessed. Blood samples (serum, plasma, EDTA) were collected in each wave and DNA and RNA once. Between 2010 and 2021 four in-person examination waves were performed, with mean follow-up intervals of 2.5 years.

Case Ascertainment:
Trained study psychologists recruited patients (age 35–65 years), hospitalized for a current episode of depression in 5 departments/clinics of psychiatry in the district of Muenster, and performed the MINI Neuropsychiatric Structured Interview (depression parts) and assessed different depression scales (HAM-D and -A), allowing a depression subtype classification. A small proportion (5% of patients) was recruited in outpatient settings. Admission and discharge ICD-10 diagnoses were documented.

Control Ascertainment:
Controls were invited as a random general population sample drawn from the (compulsory) city registry of the City of Muenster among individuals aged 35–65 years. All three cohorts were examined with identical methods.

Genotyping:
Array: Illumina HumanPsychChip_24v1-0 (PsychChip)

Ethics statement:
The joint ethics committee of the Westphalian Chamber of Physicians and the University of Muenster approved the study (2009-391-f-s) approved this study.

Funding:

**Study**	**Lead investigator**	**Award number**	**Funder**	**Country**
BiDirect	Klaus Berger	01ER0816, 01ER1506	German Ministry of Research and Education (BMBF)	Germany

Early Prediction of Adolescent Depression (EPAD) (CARD3) | Rice F; Thapar A | PMID 21962850; 34706428 | analysis code: cardm

Cohort Description:
Data come from the parent generation of The Early Prediction of Adolescent Depression (EPAD) study which is a prospective longitudinal study of the offspring of parents with recurrent depression. Parents all had a history of recurrent unipolar DSM-IV depression confirmed by diagnostic interview at baseline with a timeline of the index parent's previous depressive episodes and the SCAN (Schedules for Clinical Assessment in Neuropsychiatry).

DSM-IV criteria for bipolar disorder, mania/hypomania or psychotic disorder in the parent at interview constituted exclusion criteria.

Case Ascertainment:

Adults all had a history of recurrent unipolar depression and most (78%) were recruited from primary care settings in South Wales, UK. The presence of at least two episodes of DSM-IV major depressive disorder (MDD) in the index parent was confirmed at baseline with a timeline of the index parent's previous depressive episodes and the SCAN (Schedules for Clinical Assessment in Neuropsychiatry). DSM-IV criteria for bipolar disorder, mania/hypomania or psychotic disorder in the parent at interview were exclusion criteria. (N = 165 with genetic data and permission for sharing).

Control Ascertainment:

No controls included.

Genotyping:

Array: Illumina PsychChip_v1-1_15073391_A1-b37 (PsychChip)

Ethics statement:

At all study assessments written informed consent was obtained. The Multi-Centre NHS Research Ethics Committee for Wales reviewed and approved the study. At study assessment 4, ethical review and approval was granted by the School of Medicine Ethics Committee, Cardiff University. Written informed consent including explicit consent for data sharing was obtained at wave 4.

Funding:

**Study**	**Lead investigator**	**Award number**	**Funder**	**Country**
Predicting and preventing adolescent depression	Thapar	JT/06	Jules Thorn Charitable Trust	UK
Early onset depression: characterising development and understanding risks	Rice	MR/R004609/1	Medical Research Council	UK

Acknowledgement:
Clinical and genetic data collection was funded by the Jules Thorn Charitable Trust and the Medical Research Council. Genotyping and data support for genomic data from EPAD were provided by the National Centre for Mental Health.

McQuillin controls | McQuillin A | PMID 30859703 | analysis code: cardm

Cohort Description:
The McQuillin controls were recruited as part of the DNA Polymorphisms in Mental Disorders (DPIM) study. The DPIM sample comprised UK individuals who were ascertained and received clinical diagnoses of a severe mental disorder according to UK National Health Service (NHS) psychiatrists at interview using the categories of the International Classification of Disease version 10, as well as control subjects. All subjects were included only if both parents were of English, Irish, Welsh or Scottish descent and if three out of four grandparents were of the same descent.

Case Ascertainment:
No cases included.

Control Ascertainment:
The control subjects were recruited from centres across the UK that were also recruiting cases. All control subjects were interviewed with the SADS-L to exclude all psychiatric disorders.

Genotyping:
Array: Illumina PsychChip v1-1_15073391_A1 (PsychChip)

Ethics statement:
All volunteers read an information sheet approved by the Metropolitan Multi-centre Research Ethics Committee (approval number MREC/03/11/090) who also approved the project for all NHS hospitals. Written informed consent was obtained from each volunteer.

Funding:

Study	Lead investigator	Award number	Funder	Country
DPIM	McQuillin	-	Stanley Center	US
DPIM	McQuillin	G1000708	MRC	UK

The Early Medication Change (EMC) Trial | Lieb K; Tadic A, Engelmann J | PMID 20187947 | analysis code: emcbp

Cohort Description:
This study investigates about individuals with major depressive disorder (MDD) who had participated between 2009 and 2014 in the "Randomised clinical trial comparing an early medication change (EMC) strategy with treatment as usual (TAU) in patients with Major Depressive Disorders (MDD) - EMC Trial (ClinicalTrials.gov identifier n°: NCT00974155)" and who had agreed to biomarker sampling. The EMC trial was a multi-center, randomized, observer-blinded, controlled clinical trial investigating whether non-improvers after 14 days of an antidepressant treatment with escitalopram are more likely to attain remission (HAMD-17 ≤ 7) on treatment day 56 with an early medication change (EMC: immediate change to venlafaxine followed by an augmentation with lithium after non-response at day 28) compared to patients treated according to current guideline recommendations (TAU: continuing escitalopram for 2 weeks followed by venlafaxine in the case of non-response). 56 percent of the patients were women, mean age was 40 ± 12 years and mean depression severity at baseline was 24 ± 4 assessed by HAMD17 sum score. Blood samples were also obtained weekly as previously described. All blood was withdrawn between 08.00–12.00h in the morning and placed immediately on ice after collection.

Case Ascertainment:
Adult patients (age 18–65 and <60 years at the time of the first depressive episode) treated as in-patient or in a day hospital at one of the three participating centers in Germany with a primary diagnosis of nonpsychotic major depressive disorder (MDD) (DSM-IV) and a sum score ≥18 on the Hamilton Rating Scale for Depression (HAMD-17) at study entry were enrolled between September 2009 and March 2014. Minimal exclusion and broad inclusion criteria were used to maximize generalizability. Patients with a primary diagnosis of bipolar, psychotic, obsessive-compulsive, eating disorder or substance dependence (if it required in-patient detoxification), and female patients who were pregnant or breast-feeding were excluded from the study, as well as those with general medical conditions contraindicating the use of any protocol medication, or a clear history of non-response or intolerance in the current major depressive episode to any protocol antidepressant. Concurrent axis I disorders were assessed by the MINI International Neuropsychiatric Interview, axis II disorders by the Structured Clinical Interview for DSM-IV Axis II Disorders (SCID-II). The severity of depressive symptomatology was assessed weekly by the Hamilton Depression Rating Scale: HAMD-17 by blinded and specially trained raters.

Control Ascertainment:
No controls included.

Genotyping:
Array: InfiniumGlobal Screening Array v2.0 (GSA2)

Ethics statement:
The EMC trial was carried out in accordance with the CONSORT guidelines and was approved by the ethics committee at the Landesärztekammer Rheinland-Pfalz (code: 837.211.09 (6717).

Funding:

**Study**	**Lead investigator**	**Award number**	**Funder**	**Country**
EMC Trial	Lieb	Grant no. 01KG090	German Federal Ministry for	Germany

			Education and Research (BMBF)	

Berlin Psychosis Study (BePS) / Berlin Research Initiative for Diagnostics, Genetics and Environmental Factors (BRIDGE-S) | Ripke S | PMID 35396580; 36635663 | analysis code: emcbp

Cohort Description:
The Berlin Psychosis Study (BePS) and Berlin Research Initiatives for Diagnostics, Genetics and Environmental factors (BRIDGE-S) are case-control cohorts initiated in greater Berlin aiming to facilitate i) the discovery of novel genetic variants associated with schizophrenia and other mental disorders, ii) gene-environment analyses and iii) genotype-informed disease subtyping. The current control sample consists of individuals of European ancestry that were primarily recruited from the general population via local advertisements and participant databases. In addition, all controls who reported any prior psychiatric disorder were removed from the data set. Case status was assigned if subjects have been diagnosed with depression by trained clinicians (self-report). All subjects provided written informed consent and received monetary compensation for their participation. Saliva samples were collected from each participant using OraGene-510 DNA-Self-Collection Kits (Genotek, Ottawa, Ontario, Canada). DNA samples were genotyped on Infinitum Global Screening array-23 (GSA) MD BeadChip (Illumina, San Diego, CA) by ERASMUS Medical Center, Human Genotyping Facility (HuGe-F), Rotterdam, The Netherlands.

Case Ascertainment:
Self-reported diagnoses ("Have you ever been diagnosed with [depression] by a trained clinician, e.g., a physician or psychologist").

Control Ascertainment:
Controls were defined as having no self-reported history of psychiatric illness (diagnosed by a mental health care professional).

Genotyping:
Array: Global Screening Array GSA v1.0–3.0 (intersection of SNPs)

Ethics statement:
Ethical approval for BePS was granted in 2015 (EA1/123/15) and for BRIDGE-S in 2018 (EA1/119/18) by the local ethics committee at Charité Universitätsmedizin, Berlin.

Funding:

**Study**	**Lead investigator**	**Award number**	**Funder**	**Country**
BRIDGE-S (Project number: 445050869)	S Ripke	RI 2846/2-1	DFG	Germany
BePS	S Ripke	23545	1) Brain & Behavior Research Foundation | NARSAD grants 2) Stanley Center, Broad Institute of MIT & Harvard	USA

FOR2107 | Kircher T; Andlauer T; Forstner AJ; Dannlowski U | PMID 30267149; 31076262 | analysis code: formm

Cohort Description:
The FOR2107 cohort is a multi-centre study, recruited through newspaper advertisements and mailing lists from the areas of Marburg and Muenster in Germany. We have characterized individuals across 6 samples with the T1 battery at baseline (first subject in 9/11/2014). We re-evaluate these subjects with the T2 and T3 battery after 2 and 5 years of follow-up at Marburg and Münster Universities, Germany.

Case Ascertainment:
Interview ratings are conducted by trained PhD students at both sites according to detailed SOPs. All raters undergo standardized training, and interviews are regularly supervised and videotaped. Videotaped interviews are regularly rated by all other raters, reliability measures are generated and potential mismatches of ratings are discussed and fine-tuned. Problematic cases are discussed at a weekly conference. Currently, the overall intra-class correlations (ICC) across all videotaped interviews are HAMD (ICC=0.918), HAMA (ICC=0.959), YMRS (ICC=0.829), SANS (ICC=0.845), and SAPS (ICC=0.923).

Rating and questionnaire sets from the probands are generated as PDF files with an individualized barcode for each subject and are handed out in paper form. These forms are then scanned (using Remark Office OMR software) under quality-controlled conditions based on an annotated CFR (aCRF) (1st quality control) and converted to a database-compatible file structure. Through the data import pipeline, all datasets are checked for plausibility (e.g., range of values, missing data) according to comprehensive aCRFs. For each data import, quality control protocols are generated which gives the data quality manager an immediate and detailed feedback and enables to correct data directly (2nd quality control). By implementing a mirrored test server, this process is repeated until the error rate is zero percent (i.e., error reduction loop). Once this value is reached, data sets are released for export and can be downloaded. Pseudonymization is used for data storage for all subjects. Data storage and supervision of data management according to GCP Guidelines lie with the KKS Marburg. Biosamples and corresponding data were sampled, processed and stored in the Marburg Biobank CBBMR. Cases were ascertained in accordance with the DSM-IV.

Control Ascertainment:
Controls did not have a psychiatric diagnosis as determined in line with the DSM-IV.

Genotyping:
Array: Illumina Infinium PsychChip v1 and v3 (PsychChip)

Ethics statement:
Ethics approval was obtained from the ethics committees of the Medical Schools of the Universities of Marburg (approval identifier Study 07/2014) and Münster (approval identifier 2014-422-b-S), respectively, in accordance with the Declaration of Helsinki. All subjects volunteered to participate in the study and provided written informed consent.

Funding:

**Study**	**Lead investigator**	**Award number**	**Funder**	**Country**
FOR2107	Tilo Kircher, Udo Dannlowski	KI588/14-1, KI588/14-2, and KI588/20-1, KI588/22-1 DA 1151/5-1, DA	German Research Council (DFG)	Germany

		1151/5-2		

Great Smoky Mountains Study (GSMS) | Copeland W; Costello EJ | PMID 27010203; 24342383 | analysis code: gsmse

Cohort Description:
The Great Smoky Mountains Study (GSMS) is a prospective, longitudinal study of the development of psychiatric disorders and need for mental health services in rural and urban youth. Three cohorts of children aged 9, 11, and 13 years at intake, were recruited from 11 counties in western North Carolina in 1993. Annual assessments were completed with the children until age 16 and then again at ages 19, 21, 25, and 30. The sample includes participants of European and African ancestry. The study also oversampled for American Indians. This group constitutes only 3% of the area population, but 25% of the study sample. All psychiatric and substance variables were assessed using the Child and Adolescent Psychiatric Assessment interview with participants and a parent up until age 16, and its upward extension, the Young Adult Psychiatric Assessment, with the participant alone thereafter.

Case Ascertainment:
This is a community-representative sample and thus distributions of relevant cases reflect the relative prevalence within the community. Childhood diagnostic status was assessed with items from the structured Child and Adolescent Psychiatric Assessment (CAPA) interview. Responses from this interview were then used to generate DSM diagnoses from the symptoms assessed by computer algorithms. A symptom was counted as present if either parent or child reported it as present in the past three months. A three-month “primary period” was selected to minimize forgetting and recall bias. The reliability and construct validity of CAPA-based diagnoses is good to excellent.

Control Ascertainment:
This is a community-representative sample and thus distributions of relevant controls reflect the relative prevalence within the community. Childhood diagnostic status was assessed with items from the structured Child and Adolescent Psychiatric Assessment (CAPA) interview. Responses from this interview were then used to generate DSM diagnoses from the symptoms assessed by computer algorithms. A symptom was counted as present if either parent or child reported it as present in the past three months. A three-month “primary period” was selected to minimize forgetting and recall bias. The reliability and construct validity of CAPA-based diagnoses is good to excellent.

Genotyping:
Array: Array: Illumina Human660W-Quad v1 DNA Analysis BeadChip

Ethics statement:
Participants in all the studies gave consent for their DNA to be genotyped. The study protocol and consent forms for each assessment were approved by the Duke University Medical Center Institutional Review Board.

Funding:

**Study**	**Lead investigator**	**Award number**	**Funder**	**Country**
GSMS	Costello	R01 DA024413	NIDA	USA

TRD3 European Group for the Study of Resistant Depression (GSRD) | Serretti A; Kasper S; Souery JZD; Montgomery S; Dikeos D; Rujescu D; Mendlewicz J | PMID 30468137 | analysis code: gsrdf-gsrdg-gsrdi-gsrdp

Cohort Description:

The GSRD sample was recruited within a multicenter, cross-sectional study including adult in- and outpatients with major depressive disorder (MDD) according to DSM IV-TR criteria. GSRD-FR: France (gsrdf), GSRD-GER: Germany (gsrdg), GSRD-GR: Greece (gsrdp), GSRD-IT: Italy (gsrdi).

Case Ascertainment:
Diagnosis was confirmed using the Mini International Neuropsychiatric Interview (MINI). Inclusion criteria were: 1) having received at least one antidepressant during the current MDD episode (≥4 weeks at adequate dose) and 2) Montgomery and Åsberg Depression Rating Scale (MADRS) score > 22 at the onset of the current MDD episode. Exclusion criteria were: 1) any primary psychiatric disorder other than MDD, 2) any substance disorder (except nicotine and caffeine) in the previous six months, and 3) any condition that could interfere with the ability to give informed consent or with the assessments required by the study (e.g. linguistic barrier). Depressive symptom severity was assessed using the MADRS at study inclusion and at the onset of the current MDD episode. Information on previous and current antidepressant and other pharmacological treatments during the current MDD episode was collected as well as clinical-demographic characteristics. Antidepressant treatment was naturalistic according to best-clinical practice principles. Other than remission and MADRS improvement, the phenotype "treatment-resistant depression" is available (defined as non-response to at least two antidepressant treatments adequate in terms of duration and dose).

Control Ascertainment:
No controls included.

Genotyping:
Arrays: Illumina Infinium PsychArray 24 BeadChip (PsychChip)

Ethics statement:
All procedures contributing to this work comply with the ethical standards of the relevant national and institutional committees on human experimentation and with the Helsinki Declaration of 1975, as revised in 2008. Written informed consent was obtained from all patients included in this study. All procedures involved in the study were approved by the local ethics committees (coordinating centre (Comité d'Ethique de l'Hôpital Erasme, Bruxelles) approval number: B406201213479).

Funding:
The collection of the GSRD (Group for the Study of Resistant Depression) sample analyzed in this study was supported by an unrestricted grant from Lundbeck. Lundbeck had no further role in the study design, in the collection, analysis, and interpretation of data, in the writing of papers or in the decision to submit them for publication.

PSYDEV controls | Krebs MO | PMID 35396580 | analysis code: gsrdf

Cohort Description:
The cohort is part of the PSYDEV transdiagnosis collection (“Etude familiale et génétique des aspects développementaux des maladies psychiatriques”), a study based in Paris (France) promoted by INSERM (PI MO Krebs).

Case Ascertainment:
No cases included.

Control Ascertainment:
Control participants (N=451, Male N=207 Female N=244, age at recruitment (mean=23.7, range=[18;65])). They were either volunteers recruited from advertisement in paramedical schools, screened using a standardized short medical interview with trained psychiatrists or psychologists (N=301) or from the community and hospital staff by full assessment with DIGS 3 (N=150). Exclusion criteria included personal or family (1st degree) of psychiatric illnesses or addiction and age under 18. The screening was declarative by face-to-face medical interview in addition to self-rated questionnaires.

Genotyping:
DNA from saliva (N=301) or blood (N=150) sample were genotyped using the Illumina PsychArray at the Icahn School of Medicine at Mt. Sinai, New York, NY.

Ethics statement:
All partici-pants provided written informed consent. The study had ethics approval granted by CPP Ile de France IV which permits collaborations with foreign research teams.

Funding:

**Study**	**Lead investigator**	**Award number**	**Funder**	**Country**
PsyDev	Krebs, Marie-Odile	recurrent funding	INSERM U1266, GHU Paris	France
NIH	Sklar, Pamela	U01MH096296	U.S. National Institute of Health	USA

Gawlik schizophrenia controls (Gawlik controls) | Gawlik M | PMID 35396580 | analysis code: gsdrg

Cohort Description:
Controls were recruited as blood donors or by their participation in other studies of of University Hospital Würzburg during which they gave consent to their genetic data being used as a control sample.

Case Ascertainment:
No cases included.

Control Ascertainment:
Inclusion criteria were the absence of any psychiatric disorder, substance abuse as well as severe somatic illnesses stated by self assessment and clinical raters.

Genotyping:
Array: Controls were genotyped on Illumina's PsychChip array (Illumina, San Diego, CA, USA).

Ethics statement:
The ethical committee of Wuerzburg reviewed and approved the study (ID 135/05). All research subjects and, where applicable, their legal guardians provided a written informed consent to participate in the study.

Funding:
Controls were genotyped as part of the Psychiatric Genomics Consortium initiative (PGC3). Collection of the control sample was supported by Universitätsklinik Würzburg.

Serretti schizophrenia controls (Serretti controls) | Serretti A | PMID 35396580 | analysis code: gsrdi

Cohort Description:
Healthy individuals were recruited as controls in a study on chronic psychotic disorders at the Psychiatric Unit of the San Filippo Neri Hospital (Rome, Italy) and at the nursing home (RSA) San Raffaele “Villa dei Fiori" (Rome, Italy), between 2011 and 2012.

Case Ascertainment:
No cases included.

Control Ascertainment:
Subjects were included in the context of a medical screening, no formal psychiatric interview was administered but the absence of major and invalidating psychiatric disorder was recorded.

Genotyping:
Array: Illumina Infinium PsychArray-24 BeadChip (PsychChip)

Ethics statement:
The study was approved by the local Ethics Committee (IRCCS San Raffaele Pisana, Rome number 4/2011 and 8/11, E/38/11), and all participants provided written informed consent.

Funding:
No relevant funding.

Paschou controls | Paschou P | PMID 29392977 | analysis code: gsrdp

Cohort Description:
Community-dwelling individuals were recruited as controls for the Type 2 Diabetes study from multiple centers in Greece.

Case Ascertainment:
No cases included.

Control Ascertainment:
The control inclusion criteria were (a) age > 65 years, (b) HbA1c < 6.5% (48 mmol/mol), (c) FPG < 126 mg/dL, (d) no past history of a T2DM diagnosis as previously described and (e) no prior or current administration of anti-diabetic drugs.

Genotyping:
Array: Illumina Infinium PsychArray-24 BeadChip (PsychChip)

Ethics statement:
All study procedures conformed to the declaration of Helsinki and were approved by the Ethics Committee of the Aristotle University of Thessaloniki.

Funding:

**Study**	**Lead investigator**	**Award number**	**Funder**	**Country**
THALES	Paschou	-	National Strategic Reference Framework (NSRF)	Greece

Intern Health Study (IHS) | Sen S; Fang Y | PMID 31659322 | analysis code: ihseu; IHS_DIV

Cohort Description:
The Intern Health Study is a multi-institutional prospective cohort study that follows training physicians through the first year of residency training (internship). Interns entering residency programs across specialties were sent an email 2–3 months before starting internship (around July 1st each year) and invited to participate in the study. We measured depressive symptoms through the PHQ-9, a self-report component of the primary care evaluation of mental disorders inventory. For each of the nine depressive symptoms included in Diagnostic and Statistical Manual of Mental Disorders, interns indicated whether, during the previous 2 weeks, the symptom had bothered them 'not at all,' 'several days,' 'more than half the days' or 'nearly every day'. Each item yields a score of 0–3, so that the total score ranges from 0 to 27.
From 1 to 2 months before internship, subjects completed an online baseline survey, assessing PHQ-9 depressive symptoms, along with demographic information including age, sex and ethnicity. We then contacted the participants via email at months 3, 6, 9 and 12 of their internship year and asked them to complete online surveys assessing PHQ-9 depressive symptoms during internship.

Case Ascertainment:
PHQ depression cases were defined to be the subjects who had a score of 10 or greater on the PHQ-9 in at least one of the quarterly surveys during internship year.

Control Ascertainment:
PHQ depression controls were defined to be the subjects who had scores of less than 10 on the PHQ-9 in all of the quarterly surveys during internship year.

Genotyping:
Array: Illumina HumanCoreExome v1.0 and v1.1

Ethics statement:
The Institutional Review Board at the University of Michigan (HUM00033029) and the participating hospitals approved the study. All subjects provided informed consent after receiving the complete description of the study.

Funding:

**Study**	**Lead investigator**	**Award number**	**Funder**	**Country**
Intern Health Study (IHSUS)	Srijan Sen	R01MH101459	NIMH	USA

Incomplete Response in Late-Life Depression (IRL-GRey) | Mueller DJ; Mulsant BH; Reynolds IIIrd C; Lenze EJ] PMID 28068779; 26423282; 26963689 | analysis code: iruts

Cohort Description:
The sample includes adults aged 60 or older with DSM-IV-defined major depressive disorder and a score of at least 15 on the Montgomery-Åsberg Depression Rating Scale (MADRS). Participants received protocolized treatment with open-label venlafaxine, up to 300 mg/day for approximately 12 weeks, as part of a three-site clinical trial. Overall, ~52% of individuals reached remission at the end of treatment with an average of 50.4±37.3% change in MADRS score from baseline.

Case Ascertainment:
The study was conducted in three academic centers (University of Pittsburgh, Pennsylvania, USA [coordinating site]; Centre for Addiction and Mental Health, Toronto, Canada, and Washington University, St. Louis, Missouri, USA). From July 2009 to January 2014, we recruited adults age 60 and older who met DSM-IV criteria for a major depressive episode with at least moderate symptoms, as defined by a Montgomery Asberg Depression Rating Scale (MADRS) score ≥15 (ranges 0–60; higher scores indicate greater severity of depression). Exclusion criteria included dementia, bipolar disorder, schizophrenia, current psychotic symptoms, and alcohol or substance abuse or dependence within the past six months. Dementia was based on a view of medical records, cognitive screening, a formal review of dementia criteria, and for unclear cases, an informant's interview.

We started open treatment with venlafaxine extended release (ER) in participants to determine prospectively treatment resistance, defined by failure to remit after at least twelve weeks of treatment with at least four weeks at the highest tolerated dose (minimum of 150 mg/d and maximum 300 mg/d). Remission was defined as a MADRS score ≤10 at both of the final two consecutive visits. The majority of participants were treated for 12–14 weeks but protocol guidelines allowed for a longer trial (up to 24 weeks) if needed to clarify remission status. Those who did not achieve remission with venlafaxine monotherapy were then randomized to the addition of aripiprazole or placebo, while maintaining the final dose of venlafaxine achieved during initial open treatment. Randomization was performed using a permuted block approach to ensure treatment balance within each study site. Treatment was double-blinded; only the database administrator and research pharmacists had knowledge of treatment assignment. In this 12-week randomized phase, aripiprazole or matched placebo tablets were started at 2mg daily and titrated as tolerated to a target dose of 10mg daily that could be increased up to 15mg daily if needed.

To assess stability of remission, participants who achieved remission were then followed for an additional 12 weeks during which the study medication (aripiprazole or placebo) was continued under double-blinded conditions. Adherence was monitored in all phases by self-report and pill counts.

Control Ascertainment:
No controls included.

Genotyping:
Array: Illumina Infinium PsychArray-24 (PsychChip)

Ethics statement:
The study was conducted in three academic centers (University of Pittsburgh, Pennsylvania, USA [coordinating site]; Centre for Addiction and Mental Health, Toronto, Canada, and Washington University, St. Louis, Missouri, USA) after approval by the respective institutional review boards (Research Ethics Board, Centre for Addiction and Mental Health, Protocol number: 035-2009). An independent data and safety monitoring board oversaw the study. All participants provided written, informed consent.

Funding:
This study was supported primarily by the National Institute of Mental Health (grants R01 MH083660, P30 MH90333, and T32 MH019986 to the University of Pittsburgh; grant R01 MH083648 to Washington University; and grant R01 MH083643 to the University of Toronto). Additional funding was provided by the University of Pittsburgh Medical Center Endowment in Geriatric Psychiatry, the Taylor Family Institute for Innovative Psychiatric Research (at Washington University), the Washington University Institute of Clinical and Translational Sciences grant UL1 TR000448 from the National Center for Advancing Translational Sciences, and the Campbell Family Mental Health Research Institute at the Centre for Addiction and Mental Health, Toronto, Ontario, Canada.

Utah Suicide Genetic Research Study (USGRS) | Coon H; Lewis CM; Docherty A; Shabalin A; DiBlasi E; Stahl E; Mullins E | PMID 30353169 | analysis code: iruts

Cohort Description:

The Utah Suicide Genetic Research Study (USGRS) benefits from more than two decades of unprecedented close collaboration with the Utah Department of Health's centralized Office of the Medical Examiner (OME). Since 1999, we have collected de-identified DNA samples from state-wide suicide deaths through the Utah OME. Suicide determination is made by the OME following detailed investigation of the scene and circumstances of the death, review of medical and public records concerning the case, interviews with relatives and friends, and autopsy reports including toxicology (e.g., prescription and recreational drugs, alcohol, nicotine). Suicide determination is given conservatively due to its impact on survivors.

The USGRS includes ongoing accumulation of DNA from whole blood from all state-wide suicide deaths in Utah. In addition, beginning in 2015, we have collected and viably frozen skin biopsies from all OME suicide cases for future studies in cells. DNA from blood is extracted using the Qiagen Autopure LS automated DNA extractor. Protected Health Information (PHI) from cases is used to link each death to data within the Utah Population Database (UPDB) using secure computer servers. Information in the UPDB includes demographics, current medical information on over 11 million individuals, and deep genealogical records dating back to the 1700s. Following linkage to the UPDB information, all PHI is stripped before providing data to the research team. All cases are referenced by anonymous IDs; further contact with family members is not currently possible.

Case Ascertainment:

Cases in this sample are comprised of individuals who died by suicide in cohort with state-wide population-based ascertainment. MDD is determined using electronic health records data.

Control Ascertainment:

No controls included.

Genotyping:

Array: Illumina Infinium PsychArray-24, primarily version 1.2, some 1.0 and 1.1 (PsychChip)

Ethics statement:

Study of de-identified biosamples for the discovery of suicide risk factors is allowed through Utah State Law; we also hold Institutional Review Board approvals from the University of Utah (IRB_00044244, IRB_00133374), Intermountain Healthcare (IRB_1024977, IRB_1051740), and the Utah Department of Health (UDOH_313).

Funding:

Study	Lead investigator	Award number	Funder	Country
USGRS	Coon, H	R01MH122412	NIMH	USA
USGRS	Coon, H	R01MH123489	NIMH	USA
USGRS	Docherty, A	R01MH123619	NIMH	USA

Smoller bipolar disorder controls (Smoller controls) | Smoller JW | PMID 34002096 | analysis code: iruts

Cohort Description:
The sample has been reported as part of the PGC bipolar GWAS (PMID: 34002096, analysis code: usaw5). Controls from the sample were selected for the present analysis.

Case Ascertainment:
No cases included.

Control Ascertainment:

EHR data were obtained from a health care system of more than 4.6 million patients spanning more than 20 years. A rule-based algorithm was used to identify controls for a case-control bipolar disorder cohort. The algorithm defined controls as those age 30 years or older with no ICD-9 codes or history of medications related to a psychiatric or neurological condition. The positive predictive value (PPV) of EHR-based bipolar case or control status calculated against diagnoses from direct semistructured (SCID-IV) interviews of 190 patients by trained clinicians blind to EHR diagnosis. The EHR-based classifications were used to accrue bipolar disorder cases and controls for genetic analyses from anonymized discarded blood samples.

Genotyping:
Array: Samples were genotyped on the Illumina PsychChip Array.

Ethics statement:
The Partners HealthCare System (now called Mass General Brigham) institutional review board approved all aspects of this study.

Funding:

Study	Lead investigator	Award number	Funder	Country
	Jordan Smoller Pamela Sklar	R01 MH-085542	NIMH	USA

GeneBaseMDD | Ricken R; Adli M | PMID 30554862 | analysis code: mazdr

Cohort Description:
The ALIA (antidepressant and lithium augmentation) study is an ongoing prospective cohort study of MDD patients treated with lithium augmentation in the acute treatment of depression. Aim of the study is the pharmacogenetic and molecular prediction of response and side effects of LA in a translational approach. Individuals are clinically well characterized. Patients are recruited at 14 sites across Berlin, Brandenburg and in Leipzig (see: https://www.berliner-wissenschaftsnetz-depression.de). Individual data of the cohort were analyzed on the Charité - Universitätsmedizin Berlin HPC environment.

Case Ascertainment:
Patients were diagnosed by a supervising psychiatrist (MD). Diagnosis of MDD / Major Depressive Episode was confirmed by the Mini-International Neuropsychiatric Interview (MINI; PMID 9881538).

Control Ascertainment:
No controls included.

Genotyping:
Array: Illumina HumanOmniExpress Exome Chip (OMEX)

Ethics statement:
The local ethics committee of Charité - Universitätsmedizin Berlin (EA1/065/09) approved the study.

Funding:
The study was funded by sources of the Mood Disorders Research Unit of Charité - Universitätsmedizin Berlin, Department of Psychiatry and Psychotherapy, Campus Charité Mitte (CCM).

Reif bipolar disorder controls (Reif controls) | Reif A | PMID 28167838; 27145764 | analysis code: mazdr

Cohort Description:
Healthy participants were recruited within the context of the Collaborative Research Center SFB-TRR58, subproject Z02, from 2013 to 2015, which throughout two funding periods (2008–2016) had been designed to provide a recruitment pipeline channeling comprehensively psychometrically and genotypically characterized probands into several subprojects within the SFB-TRR58 for specific research questions (e.g., multilevel characterization of candidate gene variation in anxiety-related phenotypes).

Case Ascertainment:
No cases included.

Control Ascertainment:
Inclusion criteria were Caucasian descent (self-report up to third generation), right-handedness, and fluency in German. Exclusion criteria were manifest or lifetime DSM-IV axis I disorder, severe medical conditions, intake of centrally active medication, excessive consumption of alcohol (more than 15 units per week), nicotine (more than 20 cigarettes per day), and caffeine (more than 4 cups per day), consumption of illegal drugs, and pregnancy. The absence of mental axis I disorder was ascertained using the German version of the Mini International Psychiatric Interview (Sheehan et al. 1998). Participants filled in a set of questionnaires (see below). A venous blood sample (20 ml) was taken for genetic analyses. Participants received 50€ compensation for their participation.

Genotyping:
Array: HumanOmniExpress-12v1-1_B

Ethics statement:
Written informed consent was obtained from all individual participants included in the study. The study was approved by the ethical committees of the Universities of Würzburg and Münster and conformed to the declaration of Helsinki.

Funding:

**Study**	**Lead investigator**	**Award number**	**Funder**	**Country**
“Fear, Anxiety, Anxiety Disorders”	Reif A, Deckert J	TRR SFB 58, Z02	DFG	Germany

Epidemiological Study on Mental Health in the Autonomous Community of Andalusia (PISMA-ep) | Cervilla J; Rivera M; Gutierrez B; Molina E | PMID 27237041 | analysis code: mrive

Cohort Description:

PISMA-ep is a cross-sectional study of a representative sample of the Andalusian population which aims to establish the prevalence of major psychiatric disorders such as schizophrenia and other psychosis, bipolar disorder and major depression disorder in the Andalusian population (South of Spain). PISMA-ep participants were recruited from all the provinces of the Andalusian autonomous community. The sampling method includes a stratification of Andalusia region in provinces and the classification of population in rural, intermediate or urban areas. Individuals recruited were between 18 and 75 years old of both sexes. Information about clinical, psychological, socio-demographic, anthropometric measurements, medical conditions, medication, family psychiatric history, stressful life events, childhood maltreatment, general health state, social support, lifestyle information (smoking, alcohol consumption and physical activity), personality and neurocognitive measures among others, was also collected.

The exclusion criteria for all participants were: being outside the above mentioned age range, having lived in Andalusia for less than a year, having difficulties to communicate fluently in Spanish, being too ill to be able to complete the interview, having a diagnosis of dementia or mental retardation, living in an institution, having moved or not living normally at the address we had been given by the users database, and erroneous data from the users database (for example an incomplete address). The participants excluded were replaced with others matched for age, sex and location.

Case Ascertainment:

Major depressive disorder (MDD) patients were diagnosed following DSM-IV/ICD-10 criteria. The psychiatric interview to identify mental disorder (MDs) diagnoses was performed using the Spanish version of the MINI International Neuropsychiatric Interview (MINI), which generates diagnoses compatible with both Axis I DSM-IV and ICD-10 criteria for 16 common MDs, two additional diagnoses of major depression with melancholia and mood disorder with psychotic symptoms, one Axis II diagnosis (antisocial personality disorder), as well as a suicidal risk estimate.

Control Ascertainment:

Controls were screened for psychiatric disorders using the Spanish version of the MINI International Neuropsychiatric Interview (MINI).

Genotyping:

Array: Illumina Infinium PsychArray-24 v1.1 (PsychChip)

Ethics statement:

Individuals who also agreed to participate in the genetic analysis (n≈4,500) gave specific informed consent and provided a biological sample.

The PISMA-ep study was approved by the Research Ethics Committee of the University Hospital “San Cecilio” (PI-0322/09) and the Research Ethics Committee of the University of Granada (C.0003663).

Funding:

Study	Lead investigator	Award number	Funder	Country
PISMA-ep	Jorge Cervilla	PI322-2009	Consejería de Salud, Junta de	Spain

			Andalucía	
PISMA-ep	Jorge Cervilla	CTS-2010-6682	Consejería de Innovación, Proyecto de Excelencia	Spain
PISMA-ep	Jorge Cervilla	PI18/00467	Institute of Health Carlos III (Co-funded by European Regional Development Fund/European Social Fund “A way to make Europe”/“Investing in your future”	Spain
Med-Psych	Margarita Rivera	22514	NARSAD. Brain & Behavior Research Foundation	USA
Med-Psych	Margarita Rivera	FP7 626235	Marie Curie Research Grants Scheme	EU
PISMA-Med	Margarita Rivera	PI18/00238	Institute of Health Carlos III (Co-funded by European Regional Development Fund/European Social Fund “A way to make Europe”/“Investing in your future”	Spain

MUENS | Baune BT | PMID 29700475 | analysis code: muen2

Cohort Description:
MUENS participants are recruited from a hospital network treating inpatients in the Münster region of Germany. Control participants were drawn from the same geographic area as case participants. Both potential cases and controls (unaffected participants) were interviewed using the Structured Clinical Interview for DSM-IV (SCID) and were asked to complete a questionnaire to assess their psychiatric history. In addition, demographic information (*i.e.*, age, gender and self-identifying race and ethnicity) and medical conditions and disorders were assessed.

Case Ascertainment:
Case status of MDD was ascertained using the SCID interview. Cases reporting any lifetime symptoms indicative of psychosis or mania or major neurological or developmental conditions were excluded.

Control Ascertainment:
Control status was ascertained using the SCID interview. Controls who fulfilled the criteria for MDD or who reported any lifetime symptoms indicative of psychosis or mania or major neurological or developmental conditions were excluded.

Genotyping:
Array: Illumina Infinium PsychArray-24 v2 (PsychChip)

Ethics statement:
The study was approved by the local ethical committee of the University of Münster, and written informed consent was obtained from each patient prior to applying study procedures.

Funding:
No relevant funding.

Mater University Study of Pregnancy (MUSPC) | Middeldorp C; Najman J; Wray N | 25519422 | analysis code: muspc

Cohort Description:
The MUSP study is a population-based study of women recruited early in pregnancy over the period 1981–1983 and their live singleton children to whom they subsequently gave birth. Thirty years after recruitment, the Composite International Diagnostic Interview (CIDI), including the sections on anxiety disorders and depression, was administered to the children and DNA was collected.

Case Ascertainment:
The CIDI was administered to children of the original cohort at 30 years of age in order to diagnose anxiety disorders, depression, and/or additional disorders, such as bipolar disorder. Individuals who fulfilled the criteria for major depressive disorder according to the DSM-IV were considered a case.

Control Ascertainment:
Individuals who participated in the CIDI and did not fulfill the criteria for a major depressive disorder were considered a control.

Genotyping:
Array: Illumina Infinium PsychArray-24 v1.0 (PsychChip)

Ethics statement:
Informed consent was given and ethical approval was gained from the University of Queensland Human Research Ethics Committee 2019/HE002492, Mater Health Services Human Research Ethics Committee HREC/13/MHS/79

Funding:
This project has been funded by The National Health and Medical Research Council and the Australian Research Council, via competitive research grants.

Platform for Research Online to investigate Genetics and Cognition in Ageing (PROTECT) | Creese B; Corbett A; Ballard C; Aarsland D | PMID 28128869 | analysis code: prote

Cohort Description:
PROTECT participants were drawn from the Platform for Research Online to Investigate Genetics and Cognition in Aging (PROTECT), which is led by the University of Exeter and King's College London (https://www.protectstudy.org.uk/). PROTECT is a UK-based 25-year longitudinal online research study which gathers longitudinal data to examine the impact of lifestyle, medical and genetic risk factors of cognitive health and dementia in older adults. Twenty-nine thousand people are currently enrolled on PROTECT but this study focuses on the subset for whom genotype data is available at the time of the data freeze for this analysis. Inclusion criteria for taking part in PROTECT are: 1) age 50 or over; 2) regular access to a computer and the internet; and 3) no diagnosis of dementia. Participants were prospectively recruited from November 2015 through both local and national publicity. Invitations were also sent to persons registered for existing research studies at the Institute of Psychiatry, Psychology and Neuroscience at King's College London.

Case Ascertainment:
Ticked 'depression' in response to: "Have you been diagnosed with one or more of the following mental health problems by a professional, even if you don't have it currently?"; 01 = Depression; 02 = Mania, hypomania, bipolar or manic-depression; 03 = Anxiety, nerves or generalized anxiety disorder; 04 = Social anxiety or social phobia; 05 = Agoraphobia; 06 = Panic attacks; 07 = Obsessive compulsive disorder (OCD); 00 = None of the above; NA = Do not know; DA = Prefer not to answer. And/or PHQ-9 score ≥10.

Control Ascertainment:
Did not tick 'depression' in response to: "Have you been diagnosed with one or more of the following mental health problems by a professional, even if you don't have it currently?"; 01 = Depression; 02 = Mania, hypomania, bipolar or manic-depression; 03 = Anxiety, nerves or generalized anxiety disorder; 04 = Social anxiety or social phobia; 05 = Agoraphobia; 06 = Panic attacks; 07 = Obsessive compulsive disorder (OCD); 00 = None of the above; NA = Do not know; DA = Prefer not to answer. Also did not have a PHQ-9 score ≥10.

Genotyping:
Array: Infinium® Global Screening Array-24 v1.0 with 50k SNP custom content

Ethics statement:
Informed consent was given through an electronic online process and ethical approval was gained through the London Bridge National Research Ethics Committee (Reference: 13/LO/1578).

Funding:

This work was funded in part through the MRC Proximity to Discovery: Industry Engagement Fund (External Collaboration, Innovation and Entrepreneurism: Translational Medicine in Exeter 2 (EXCITEME2) ref. MC_PC_17189) awarded to Dr Creese. This paper represents independent research part funded by the National Institute for Health Research (NIHR) Biomedical Research Centre at South London and Maudsley NHS Foundation Trust and King's College London. This study was supported by the National Institute for Health and Care Research Exeter Biomedical Research Centre. The views expressed are those of the author(s) and not necessarily those of the NIHR or the Department of Health and Social Care. This research was also supported by the NIHR Collaboration for Leadership in Applied Health Research and Care South West Peninsula. Genotyping was performed at deCODE Genetics. The authors would like to acknowledge the use of the research computing facility at King's

College London, Rosalind (https://rosalind.kcl.ac.uk), which is delivered in partnership with the NIHR Biomedical Research Centres at South London & Maudsley and Guy's & St. Thomas' NHS Foundation Trusts, and part-funded by capital equipment grants from the Maudsley Charity (award 980) and Guy's & St. Thomas' Charity (TR130505).

**Study**	**Lead investigator**	**Award number**	**Funder**	**Country**
PROTECT	Byron Creese	MC_PC_17189	Medical Research Council	UK
PROTECT	Anne Corbett	AS-114	Alzheimer's Society	UK
PROTECT	Clive Ballard/Dag Aarsland	-	NIHR	UK

Thematically Organized Psychosis (TOP) Study | Andreassen O; Djurovic S; Smeland OB | PMID 20185149 | analysis code: topmd

Cohort Description:
The cohort comprises participants from the TOP (Thematically Organized Psychosis Study including the BRAIN Network), with patients suffering from severe mental illness recruited from psychiatric hospitals and clinics in Norway, mainly from the Oslo area in the South-Eastern Region, but also some from Bergen (Western Region), and Trondheim (Middle Region) of Norway. The participants contributing to the present PGC MDD analysis are of European ancestry, but participants of other ancestries are also recruited to this cohort. The rationale, design and methods of the TOP study are described in detail elsewhere (PMID 20185149).

Case Ascertainment:
The MDD cases were recruited from psychiatric hospitals or clinics and met criteria for MDD using DSM-IV, diagnosed using SCID or MINI.

Control Ascertainment:
The controls included blood donors and population controls from the Oslo University Hospital and healthy controls screened for psychiatric illness, all recruited from the South-Eastern region of Norway.

Genotyping:
Array: Illumina OmniExpress BeadChip (OMEX), genotyped at deCODE genetics, Reykjavik, Iceland

Ethics statement:
All participants provided written informed consent and the human subjects protocol was approved by the Norwegian Scientific-Ethical Committee and the Norwegian Data Protection Agency.

Funding:

**Study**	**Lead investigator**	**Award number**	**Funder**	**Country**
TOP	Ole Andreassen	223273, 273291, 296030	Research Council of Norway	Norway

Tracking Adolescents' Individual Lives Survey (TRAILS) | Oldehinkel AJ | PMID 25431468; 18263649 | analysis code: trail

Cohort Description:
The dataset consists of 1,448 unrelated 18-20-year olds (49.6% females) from the North of the Netherlands, selected among the general population (81.8%) and former patients of a regional outpatient clinic (18.9%). The rationale, design and methods of the study are described in detail elsewhere (PMID 25431468, 18263649). Briefly, TRAILS is an ongoing cohort study aimed to better understand the determinants of mental (ill-)health and social development during adolescence and young adulthood, as well as the mechanisms underlying the associations between determinants and outcomes. In 2001–2002 2,230 participants aged 10-12 years, selected from municipality registers, were included in the study and measured approximately every 3 years since then. In 2004, the study was extended with 543 same-aged children who visited a psychiatric outpatient clinic before the age of 11, who followed comparable procedures as the population cohort.

Case Ascertainment:
The cohort includes 228 individuals with a lifetime diagnosis of MDD according to the DSM-IV, as assessed with the Composite International Diagnostic Interview (CIDI, version 3.0) at about age 19.

Control Ascertainment:
Controls were determined based on the CIDI 3.0 too. The cohort contains 1220 participants without a lifetime diagnosis of DSM-IV MDD, and 918 participants without a history of any mood or anxiety disorder at age 19.

Genotyping:
Array: Illumina Cyto SNP12v2.

Ethics statement:
The TRAILS study was approved by the Dutch Central Committee on Research Involving Human Subjects (NL22114.042.08), participants were treated in accordance with the Declaration of Helsinki, and written consent was acquired from all participants and their parents.

Funding:

Study	Lead investigator	Award number	Funder	Country
TRAILS	Verhulst FC	100-001-004	NWO	Netherlands
TRAILS	Ormel J	175.010.2003.005	NWO	Netherlands
TRAILS	Oldehinkel AJ	480-07-001	NWO	Netherlands
TRAILS	Hartman CA	CP32	BBMRI-NL	Netherlands

Yale-Penn (YP) Study | Gelernter J; Polimanti R; Kranzler H | PMID 23958962; 24143882; 24166409; 25555482 | analysis code: yapeu

Cohort Description:
Yale-Penn (YP) study includes participants recruited in the eastern US, predominantly in Connecticut and Pennsylvania. They were administered the Semi-Structured Assessment for Drug Dependence and Alcoholism (SSADDA) to derive DSM-IV diagnoses of lifetime substance use disorders and other major psychiatric traits.

Case Ascertainment:
MDD cases were defined using DSM-IV criteria and responses from the SSADDA. Cases had experienced 5+ MDD symptoms for a duration of at least 5 weeks, had clinically significant distress or impairment. Exclusions were made if participants had these symptoms related to drug use or dependence or due to acute bereavement. Participants with symptoms better accounted for by another disorder (schizophrenia or bipolar), pregnancy, or who had experienced manic episodes were also excluded.

Control Ascertainment:
MDD controls were defined using DSM-IV criteria and responses from the SSADDA.

Genotyping:
Array: Illumina HumanOmni1-Quad v1.0

Ethics statement:
The study received IRB approval from all participating institutions and written informed consent was obtained from all study participants. These included the Yale Human Investigation Committee, University of Connecticut Health Center Institutional Review Board, University of Pennsylvania Institutional Review Board, Medical University of South Carolina Institutional Review Board, McLean Hospital Institutional Review Board.

Funding:

**Study**	**Lead investigator**	**Award number**	**Funder**	**Country**
Yale Penn	Joel Gelernter	RC2 DA028909, R01 DA12690, R01 DA12849, R01 DA18432, R01 AA11330, & R01 AA017535	NIH	USA
Yale Penn	Henry Kranzler	R21 DA10242<br>R01 DA18532	NIH	USA

Summary samples

23andMe | 23andMe Research Team | PMID 27479909 | analysis code: mdd_23andMe.eur.hg19.v7_2_202012; 23andMe_AFR; 23andMe_EAS; 23andMe_EUR; 23andMe_HIS

Cohort Description:
23andMe is a company providing direct-to-consumer genetic testing as a paid service to its customers. 23andMe customers are invited to answer research questionnaires and, subject to participants' informed consent, their health data and genotyping results are included in genetic analyses. Subject to agreements between 23andMe and the PGC, summary finding of genetic analyses were shared with the PGC for inclusion in the current GWAS meta-analysis. The full GWAS summary statistics for the 23andMe discovery data set will be made available through 23andMe to qualified researchers under an agreement with 23andMe that protects the privacy of the 23andMe participants. Datasets will be made available at no cost for academic use. Please visit https://research.23andme.com/collaborate/#dataset-access/ for more information and to apply to access the data.

Case Ascertainment:
Phenotypic status was based on responses to web-based surveys, with individuals that self-reported as having received a clinical diagnosis or treatment for depression classified as cases.

Control Ascertainment:
Control status was based on responses to web-based surveys, and individuals that didn't self-report as having received a clinical diagnosis or treatment for depression were classified as controls.

Genotyping, QC, Imputation:
DNA extraction and genotyping were performed on saliva samples by National Genetics Institute (NGI). Samples were genotyped on five different genotyping platforms. Samples that failed to reach 98.5% call rate were re-analyzed. Individuals whose analyses failed repeatedly were recontacted by 23andMe customer service to provide additional samples. Imputation was conducted with a reference panel that combines the May 2015 release of the 1000 Genomes Phase 3 haplotypes with the UK10K imputation reference panel. Prior to imputation, each chromosome of the reference panel was split into chunks of no more than 300,000 variants, with overlaps of 10,000 variants on each side. A single batch of 10,000 individuals was used to estimate Minimac3 imputation model parameters for each chunk. Phased participant data were generated using Finch, a tool internally developed by 23andMe that implements the Beagle graph-based haplotype phasing algorithm12, and Eagle213. Haplotype graphs were constructed for all participants from a representative sample of genotyped individuals, and then out-of-sample phasing of all genotyped individuals was performed against the appropriate graph. For the X chromosome, separate haplotype graphs were built for the non-pseudoautosomal region and each pseudoautosomal region, and these regions were phased separately. Phased participant data were imputed against the merged reference panel using Minimac3, treating males as homozygous pseudo-diploids for the non-pseudoautosomal region.

Ethics statement:
Participants provided informed consent and volunteered to participate in the research online, under a protocol approved by the external AAHRPP-accredited IRB, Ethical & Independent (E&I) Review Services. As of 2022, E&I Review Services is part of Salus IRB (https://www.versiticlinicaltrials.org/salusirb).

Funding:

23andMe is funded by its investors, shareholders, by providing genotyping and feedback paid service to its subscribers.

Acknowledgement:
We would like to thank the research participants and employees of 23andMe for making this work possible. The following members of the 23andMe Research Team contributed to this study: Stella Aslibekyan, Adam Auton, Elizabeth Babalola, Robert K. Bell, Jessica Bielenberg, Katarzyna Bryc, Emily Bullis, Daniella Coker, Gabriel Cuellar Partida, Devika Dhamija, Sayantan Das, Sarah L. Elson, Nicholas Eriksson, Teresa Filshtein, Alison Fitch, Kipper Fletez-Brant, Pierre Fontanillas, Will Freyman, Julie M. Granka, Karl Heilbron, Alejandro Hernandez, Barry Hicks, David A. Hinds, Ethan M. Jewett,, Katelyn Kukar, Alan Kwong, Keng-Han Lin, Bianca A. Llamas, Maya Lowe, Jey C. McCreight, Matthew H. McIntyre, Steven J. Micheletti, Meghan E. Moreno, Priyanka Nandakumar, Dominique T. Nguyen, Elizabeth S. Noblin, Jared O'Connell, Aaron A. Petrakovitz, G. David Poznik, Alexandra Reynoso, Morgan Schumacher, Anjali J. Shastri, Janie F. Shelton, Jingchunzi Shi, Suyash Shringarpure, Qiaojuan Jane Su, Susana A. Tat, Christophe Toukam Tchakouté, Vinh Tran, Joyce Y. Tung, Xin Wang, Wei Wang, Catherine H. Weldon, Peter Wilton, Corinna D. Wong.
Y.J. and members of 23andMe Research Team are employed by and hold stock or stock options in 23andMe, Inc.

Australian Genetics of Depression Study (AGDS1) | Martin N; Byrne E; Wray N; Medland S; Mitchell B | PMID 32461290; 34924174 | analysis code: mdd_AGDS.eur.hg19.202012

Cohort Description:
The Australian genetics of depression study (AGDS) comprises participants that self-report a diagnosis of depression. There were two methods of recruitment into the study: a media appeal recruiting volunteers with a self-reported diagnosis of depression, and ascertainment through Australian government prescription records. Controls were drawn from the Qskin Study at QIMR Berghofer Medical Research Institute.

Case Ascertainment:
Cases were defined as individuals that met criteria for an MDD diagnosis using the DSM-5 as assessed by the CIDI short-form and excluded individuals that self-reported an additional diagnosis of bipolar disorder or schizophrenia.

Control Ascertainment:
Qskin participants answered the lifestyle questionnaire which included a disease checklist including questions about ever having been diagnosed with psychiatric disorders. Participants of European ancestry who reported not having been given a diagnosis of any psychiatric disorder were selected as controls.

Genotyping, QC, Imputation:
Array(s): Illumina Infinium Global Screening Array

Genotyping was conducted using the Illumina Infinium Global Screening Array platform. Prior to imputation, a common set of high QC markers between the different genotyping batches was obtained. Marker exclusion criteria included: unknown or ambiguous map position and strand alignment in a BLAST search, missingness >5%, p(HWE test)< 10^-6), MAF<1%, GenTrain score <0.6. The Michigan imputation server was used to impute the genotypes using the HRCr1.1 as a reference panel. Individuals were excluded based on a high missingness (missing rate > 3%), inconsistent (and unresolvable) sex, or if deemed ancestry outliers from the European population (6 standard deviations from the first two genetic principal components from 1000 Genomes). Imputed genotype dosages were used for the analyses.

GWAS was carried out in SAIGE (v0.36.3.3) in R 3.6.1 using a generalized linear mixed model to account for population stratification, cryptic relatedness and unobserved genetic confounding. The GWAS was further adjusted for genotyping batch and the first 10 principal components. Variants with MAF<1% and imputation accuracy score <0.7 were excluded from the results.

Ethics statement:
All participants in the AGDS and Qskin cohorts provided informed consent that they had read and understood the study information sheets and to confirm that they would be willing to provide a saliva sample for genotyping and downstream generic analyses. All study protocols were approved by the QIMR Berghofer Medical Research Institute Human Research Ethics Committee - approval numbers P2118, P1309 and P2304.

Funding:

**Study**	**Lead investigator**	**Award number**	**Funder**	**Country**
AGDS	Martin,N<br>Medland, S	1086683,<br>1145645,<br>1078901,<br>1087889	National Health and Medical Research Council	Australia

			(NHMRC)	
Qskin	Whiteman, D<br>Olsen, C	1073898,<br>1058522,<br>1123248	National Health and Medical Research Council (NHMRC)	Australia

Avon Longitudinal Study of Parents and Children (ALSPAC) | Timpson NJ; Kwong ASF | PMID 22507743; 22507742; 31020050 | analysis code: mdd_ALSPAC.eur.hg19.27022020

Cohort Description:
The Avon Longitudinal Study of Parents and Children (ALSPAC) is a longitudinal cohort study that recruited pregnant women residing in the former area of Avon, UK with expected dates of delivery 1st April 1991 to 31st December 1992. The initial cohort consisted of 14,062 live births, but has been increased to 14,901 children who were alive after one year with further recruitment.

This study uses data from the original children, taken from the ages of 18 and 24. The study website contains details of all the data that is available through a fully searchable data dictionary and variable search tool: http://www.bristol.ac.uk/alspac/researchers/our-data. We are extremely grateful to all the families who took part in this study, the midwives for their help in recruiting them, and the whole ALSPAC team, which includes interviewers, computer and laboratory technicians, clerical workers, research scientists, volunteers, managers, receptionists and nurses. Part of this data was collected using REDCap, see the REDCap website for details (https://projectredcap.org/resources/citations/).

Case Ascertainment:
Information about major depression diagnosis was collected in ALSPAC using data from two research clinics: participants were assessed once at age 18, and again at age 24. The major depression phenotype was derived using the Clinical Interview Schedule – Revised (CISR) (PMID 1615114), which measures the presence of psychiatric disorders using ICD-10 diagnostic criteria. The CISR was administered via a computer terminal on both occasions. Any major depression was defined as having met the threshold for depression within the last 2 weeks at either age 18 and/or age 24.

Control Ascertainment:
Controls were those who did meet the criteria for depression (assessed via the CISR) at the clinics.

Genotyping, QC, Imputation:
Array(s): Illumina HumanHap550 quad chip (children), Illumina human660W-quad array (mothers)

ALSPAC children were genotyped using the Illumina HumanHap550 quad chip genotyping platforms by 23andme subcontracting the Wellcome Trust Sanger Institute, Cambridge, UK and the Laboratory Corporation of America, Burlington, NC, US. The resulting raw genome-wide data were subjected to standard quality control methods on 9,915 subjects and 550,000 SNPs. Individuals were excluded on the basis of gender mismatches; minimal or excessive heterozygosity; disproportionate levels of individual missingness (>3%) and insufficient sample replication (IBD < 0.8). Population stratification was assessed by multidimensional scaling analysis and compared with Hapmap II (release 22) European descent (CEU), Han Chinese, Japanese and Yoruba reference populations; all individuals with non-European ancestry were removed by removing samples that clustered outside the CEU HapMap2 population using this multidimensional scaling of genome-wide IBS pairwise distances. SNPs with a minor allele frequency of < 1%, a call rate of < 95% or evidence for violations of Hardy-Weinberg equilibrium (P < 5E-7) were removed. Cryptic relatedness was measured as proportion of identity by descent (IBD > 0.1). Related subjects that passed all other quality control thresholds were retained during subsequent phasing and imputation. 9,115 subjects and 500,527 SNPs passed these quality control filters.

ALSPAC mothers were genotyped using the Illumina human660W-quad array at Centre National de Genotypage (CNG) and genotypes were called with Illumina

GenomeStudio. PLINK (v1.07) was used to carry out quality control measures on an initial set of 10,015 subjects and 557,124 directly genotyped SNPs. SNPs were removed if they displayed more than 5% missingness or a Hardy-Weinberg equilibrium P value of less than 1.0e-06. Additionally, SNPs with a minor allele frequency of less than 1% were removed. Samples were excluded if they displayed more than 5% missingness, had indeterminate X chromosome heterozygosity or extreme autosomal heterozygosity. Samples showing evidence of population stratification were identified by multidimensional scaling of genome-wide identity by state pairwise distances using the four HapMap populations as a reference, and then excluded. Cryptic relatedness was assessed using an IBD estimate of more than 0.125 which is expected to correspond to roughly 12.5% alleles shared IBD or a relatedness at the first cousin level. Related subjects that passed all other quality control thresholds were retained during subsequent phasing and imputation. 9,048 subjects and 526,688 SNPs passed these quality control filters.

We combined 477,482 SNP genotypes in common between the sample of mothers and sample of children. We removed SNPs with genotype missingness above 1% due to poor quality (11,396 SNPs removed) and removed a further 321 subjects due to potential ID mismatches. This resulted in a dataset of 17,842 subjects containing 6,305 duos and 465,740 SNPs (112 were removed during liftover and 234 were out of HWE after combination). We estimated haplotypes using ShapeIT (v2.r644) which utilises relatedness during phasing. We obtained a phased version of the 1000 genomes reference panel (Phase 1, Version 3) from the Impute2 reference data repository (phased using ShapeIT v2.r644, haplotype release date Dec 2013). Imputation of the target data was performed using Impute V2.2.2 against the reference panel (all polymorphic SNPs excluding singletons), using all 2186 reference haplotypes (including non-Europeans). This resulted in 28,699,419 SNPs, with 8,282,911 SNPs with a MAF >0.01 and info score of >0.8.

This gave 8,237 eligible children and 8,196 eligible mothers with available genotype data after exclusion of related subjects using cryptic relatedness measures described previously. Only ALSPAC children were used in the following analyses.

Analysis were conducted using SNPTEST v2.5.2, adjusting for sex and the first 10 principal components of ancestry. Age was not included in the model as ALSPAC participants show little variation in age as they were all recruited at birth between April 1991 and December 1992.

Ethics statement:
Ethical approval was obtained from the ALSPAC Ethics and Law Committee and the local research ethics committees (project number B3118). Consent for biological samples has been collected in accordance with the Human Tissue Act (2004). GWAS data was generated by Sample Logistics and Genotyping Facilities at Wellcome Sanger Institute and LabCorp (Laboratory Corporation of America) using support from 23andMe.

Funding:
The UK Medical Research Council and Wellcome (Grant Ref: 217065/Z/19/Z) and the University of Bristol provide core support for ALSPAC. GWAS data was generated by Sample Logistics and Genotyping Facilities at Wellcome Sanger Institute and LabCorp (Laboratory Corporation of America) using support from 23andMe. A comprehensive list of grant funding is available on the ALSPAC website.

Study	Lead investigator	Award number	Funder	Country
ALSPAC	Timpson, N. J.	217065/Z/19/Z	MRC; Wellcome	UK

Airwave Health Monitoring Study (AHMS) | Elliot P; McIntosh A | PMID 25194498 | analysis code: mdd_Airwave.eur.hg19.0820

Cohort Description:
Airwave - The Airwave Health Monitoring Study is an occupational cohort of employees of 28 police forces from across Great Britain. Full details of the cohort and methods are available in Elliott et al. Environ Res 2014;134:280-285. The study started recruitment in 2006 and now contains 53,280 participants. At the baseline health screening, participants underwent health examination, self-completed a computer questionnaire and blood samples were collected in EDTA tubes for DNA extraction.

Case Ascertainment:
Cases were identified as those self-reporting the diagnosis of depression by a healthcare professional.

Control Ascertainment:
Controls were those who completed the health screening questionnaires and did not self-report a diagnosis of depression.

Genotyping, QC, Imputation:
Genotyping was performed on the Illumina Infinium HumanCoreExome-12v1-1 BeadChip and quality control filters including call rate (>=97%), heterozygosity rate (<=3SD from the mean) were applied on the samples. Duplicated and second-degree relatives were further excluded and 14,062 samples of European ancestry based on principle component analysis remained. Markers were removed for high missing rate (>2%), deviation from Hardy-Weinberg equilibrium (P<1E-5) or minor allele frequency below 1%, resulting in 254,027 high-quality and common markers. Imputation was performed using the Haplotype Reference Consortium (HRC) panel (version r1.1 2016).

Ethics statement:
The study received ethical approval from the National Health Service Multi-Site Research Ethics Committee (MREC/13/NW/0588).

Funding:

Study	Lead investigator	Award number	Funder	Country
Airwave	Paul Elliott	780-TETRA	Home Office	UK
Airwave	Paul Elliott	MR/R023484/1	Medical Research Council	UK
Airwave	Paul Elliott		National Institute for Health Research	UK
Airwave	Paul Elliott	MR/L01632X/1	Medical Research Council	UK

The Biology, Affect, Stress, Imaging and Cognition Study (BASIC) | Skalkidou A; Lu Y; Bränn E; Kunovac Kallak T | PMID 31641004 | analysis code: mdd_BASIC.eur.hg19.202011

Cohort Description:
The population-based, prospective Biology, Affect, Stress, Imaging and Cognition Study (BASIC, www.basicstudie.se) has the aim to investigate the biopsychosocial aetiological processes involved in perinatal depression (PND) and to pinpoint its predictors in order to improve early detection. Participants were recruited from September 2009 to November 2018 at Uppsala University Hospital in Sweden. After inclusion around gestational week 16–18, participants were followed-up with data collection points around gestational week 32, at childbirth, as well as three times postpartum (after 6 weeks, 6 months, and 1 year). In these internet-based surveys, the main outcome is depression symptoms, assessed by the Edinburgh Postnatal Depression Scale. In addition to internet-based surveys with self-report instruments, participants contribute with biological samples, for example, blood samples (maternal and from umbilical cord), biopsies (umbilical cord and placenta) and microbiota samples. A nested case-control subsample also takes part in cognitive and emotional tests, heart rate variability tests and bioimpedance tests.

Case Ascertainment:
The BASIC study is a prospective cohort study of 6,478 pregnancies; about a half were genotyped. This GWAS included 1,003 MDD cases and 1,854 controls. Cases are 1) those who received inpatient or outpatient specialist care from 5 years and 9 months prior to the childbirth until 2018; or 2) received positive diagnosis in the DSRS or MINI interviews; or 3) with self-reported depression. We applied additional criteria because the register data coverage is not lifetime, but pregnancy-related.

Control Ascertainment:
Controls included all genotyped samples that did not meet any of the inclusion criteria. Individuals with a specialist diagnosis of bipolar disorder, schizophrenia, or substance abuse disorder were excluded.

Genotyping, QC, Imputation:
Array: Illumina Global Screening Array-Multi Disease version2 (GSAMD-24v2)

Pre-imputation QC:
3,006 samples with both genotypes and MDD phenotypes (1,051 MDD cases and 1,955 controls) were processed using the RICOPILI pipeline for quality control (QC). After first removing 16,332 SNPs with missingness > 0.05, 4 samples failed sample QC due to any of the following: per-sample call rate <0.98; excessive heterozygosity (FHET outside +/-0.20); sex mismatch. 222,082 out of 759,993 markers failed SNP QC due to any of the following: per-SNP call rate <0.98; invariant; Hardy-Weinberg disequilibrium (p-value<1e-6 in controls and P<1e-10 in cases); difference in call rate between cases and controls > 0.02; minor allele frequency < 0.01 (see page 2 for QC indicators). By projecting the first two principal components (PCs) of the study samples to the reference panel of 1000 Genome global population, we identified 89 samples as non-European ancestral outliers, whose first two PCs exceeded 6 standard deviations from the mean values of the European samples in the reference population. We additionally filtered the rare variants (MAF<1%) before imputation.
Imputation:
We then used the Sanger imputation service to impute the post-QC genotype data to the reference panel of Haplotype Reference Consortium data (HRC1.1). EAGLE2 and IMPUTE2 were used for prephasing and imputing respectively. 40M SNPs were available after the HRC imputation.

Post-imputation QC:

We defined closely related samples with pairwise π_i (i.e. proportion of genome that are shared in identity-by-descent; calculated using --genome option in PLINK) ≥0.2. 56 pairs of related individuals were detected.

Analysis:
We tested for SNP associations in 1,003 MDD cases and 1,854 controls post-QC (i.e., excluding 4 failing sample QC, 89 non-European ancestral outliers and 56 related samples). In addition, we adjusted for the first four PCs to control for the subpopulation stratification. The lambda value was 0.994 (lambda 1000=0.995), indicating limited evidence for genomic inflation due to population structure.

Ethics statement:
Ethics approval was obtained from the Regional Ethical Review Board in Uppsala before commencement of the study (Dnr 2009/171, date: 20090701), with amendments. Amendment 5 was specifically for whole genome analysis.

Funding:
The study was partially supported by the Swedish Research Council (523-2014-2342 and 523-2014-07605), the Marianne and Marcus Wallenberg Foundation (MMW2011.0115), the Swedish Medical Association (SLS-250581), the Uppsala University Hospital (2012-Skalkidou) to A.S.

Study	Lead investigator	Award number	Funder	Country
BASIC	Alkistis Skalkidou	523-2014-2342 and 523-2014-07605	Swedish Research Council	Sweden
BASIC	Alkistis Skalkidou	MMW2011.0115	Marianne and Marcus Wallenberg Foundation	Sweden
BASIC	Alkistis Skalkidou	SLS-250581	Swedish Medical Association	Sweden
BASIC	Alkistis Skalkidou	2012-Skalkidou	Uppsala University Hospital	Sweden

Acknowledgement:
The computations were enabled by resources provided by the Swedish National Infrastructure for Computing (SNIC) at the UPPMAX server partially funded by the Swedish Research Council through grant agreement no. 2018-05973.

Biorepository of Vanderbilt University (BioVU) | Davis LK | PMID 20443953; 18500243 | analysis code: mdd_BioVU.eur.hg19.NoCov_SAIGE_051821; BioVU_AFR

Cohort Description:
A total of 7,757 cases and 24,723 controls of European ancestry (as determined by principal component analysis) and a total of 998 cases and 4,938 controls of African ancestry were identified using international classification of diseases (ICD) codes, version 9 and 10, from the EHR-linked biorepository at Vanderbilt University Medical Center, BioVU.

Case Ascertainment:
Cases were selected using ICD-9 and ICD-10 codes for major depressive disorder. A single instance of an inclusion code and no instances of exclusion codes was required for case assignment using the patient's EHR. This included codes that described episodes that were recurrent, single, in remission or were classified as other depressive episodes. Patients were excluded if they had an ICD code for dementia, bipolar disorder, psychotic disorders, a manic episode without psychotic symptoms, personality disorder, persistent mood disorders, or development disorders such as autism. The full list of inclusion and exclusion ICD codes are described in Supplementary Table S1.

Control Ascertainment:
Patients without any of the aforementioned inclusion or exclusion codes that were used to identify cases were selected as controls.

Genotyping, QC, Imputation:
Individuals in BioVU were genotyped on the MEGA Genotyping Array. A quality control pipeline was applied that removed SNPs with a low genotyping call rate (< 0.95), a minor allele frequency of less than 1%, and a significant deviation from Hardy-Weinberg Equilibrium. Individuals were removed if they were related (pi-hat > 0.2), had low call rates (< 0.98), sex discrepancies, or high heterozygosity (Fhet > 0.2). Imputation was aligned to the Haplotype Reference Consortium reference panel using minimach on the Michigan Imputation Server. A principal component (PCs) analysis (PCA) was performed using FlashPCA2 and was used to stratified individuals by ancestral origin.

Ethics statement:
This study was approved by the Institutional Review Board at Vanderbilt University Medical Center.

Required acknowledgment statement for the use of Vanderbilt resources:
CTSA (SD, Vanderbilt Resources)
The project described was supported by the National Center for Research Resources, Grant UL1 RR024975-01, and is now at the National Center for Advancing Translational Sciences, Grant 2 UL1 TR000445-06. The content is solely the responsibility of the authors and does not necessarily represent the official views of the NIH.

BioVU
The dataset(s) used for the analyses described were obtained from Vanderbilt University Medical Center's BioVU which is supported by numerous sources: institutional funding, private agencies, and federal grants. These include the NIH funded Shared Instrumentation Grant S10RR025141; and CTSA grants UL1TR002243, UL1TR000445, and UL1RR024975. Genomic data are also supported by investigator-led projects that include U01HG004798, R01NS032830, RC2GM092618, P50GM115305, U01HG006378, U19HL065962, R01HD074711; and additional funding sources listed at https://victr.vumc.org/biovu-funding/.

Funding:

Study	Lead investigator	Award number	Funder	Country
Mental health and chronic disease: A psycheMERGE investigation into the shared biology underlying psychiatric disorders and their physical comorbidities	Lea K. Davis	R56MH120736	National Institute of Mental Health	USA
PsycheMERGE: Leveraging electronic health records and genomics for mental health research	Lea K. Davis	R01MH118223	National Institute of Mental Health	USA

Copenhagen Hospital Biobank (CHB)/ Danish Blood Donator Study (DBDS) | Thorgeirsson T; Werge T | PMID 33169150; 31182452 | analysis code: mdd_DBDS.eur.hg19.FINAL202103

Cohort Description:
In total, the summary statistics provided to the meta-analysis were based on the genotypes of individuals with depression (n=13,347) compared with controls not diagnosed with depression (n=145,996). The results were obtained by joint analysis of cases and controls from DBDS and the CHB study on pain and degenerative musculoskeletal diseases (CHB-PDS) conducted according to the approval by the Danish Scientific Ethical Committee (NVK-1700407 and NVK-1803812). To prevent overlap with subjects from the iPSYCH study, only subjects born prior to 1981 were included.

CHB includes leftover EDTA whole blood from samples drawn for blood type testing or red cell antibody screening in approximately 25% of all hospitalized patients and outpatients at Danish hospitals. In February 2020 the biobank counted ∼423 000 unique patients, of which genome-wide genotype data was available for ∼154 000 patients above 18 years of age, selected after approval of study protocols by the relevant authorities (see Ethical issues below). There are no exclusion criteria for CHB except that each patient is included only once, i.e. repetitive blood sampling is currently not an option.

The Danish Blood Donor Study (DBDS; dbds.dk) is a large prospective cohort of blood donors (∼230 000) (http://www.bloddonor.dk) of which 110,000 are included in the DBDS Genomic Cohort, which assesses common single nucleotide polymorphisms (SNPs). The inclusion and exclusion criteria for blood donation and participation in DBDS are the same with 95% of the blood donors who are invited agreeing to participate in DBDS. The deferral rules can be seen at http://www.bloddonor.dk.

Case Ascertainment:
Cases included individuals diagnosed with depression (ICD-10 codes F32*, F33*, F41.2, and ICD-8 codes 296.2, 300.4) according to the National Patient Register (Landspatientregisteret; LPR).

Control Ascertainment:
Controls included all individuals in the CHB-PDS cohort without depression available according to the approval by the Danish Scientific Ethical Committee (NVK-1803812) using linked data from the National Patient Register (Landspatientregisteret; LPR).

Genotyping, QC, Imputation:
All samples were genotyped at deCODE genetics using the Global Screening Array (GSA) by Illumina. Whole genome sequencing, chip-typing, quality control, long-range phasing, and imputation from which the data for this analysis were generated was performed at deCODE genetics. The samples were long-range phased together with over 200,000 genotyped samples from North-western Europe using Eagle2 (PMID: 27694958). Samples and variants with less than 98% yield were excluded. A haplotype reference panel was prepared in the same manner as for the Icelandic samples (PMID: 19165921, 25807286) by phasing whole-genome sequence genotypes of ~15,000 individuals from Scandinavia, the Netherlands, and Ireland using the phased chip data. Graphtyper (PMID: 28945251) was used to call the genotypes which were subsequently imputed into the phased chip data.

Logistic regression was used to test for association between sequence variants and depression. Using the additive model, the expected allele counts were used as a covariate, and the analysis adjusted for sex, whether the individual had been chip-typed and/or sequenced, and the first 20 principal components. LD score regression was used to account for distribution inflation due to cryptic relatedness and population stratification (PMID: 25642630) and used the intercept as correction factor (CF = 1.03). The resulting summary statistics contained results for 7,737,239 markers (MAF < 1*, imputation info > 0.8).

Ethics statement:

Data analysis of CHB-PDS participants within this study was performed under the protocol "Genetics of Pain and Degenerative Musculoskeletal Diseases – a Genome-Wide Association study on repository samples from Copenhagen Hospital Biobank" approved by the Danish Data Protection Agency (P-2019-51) and the National Committee on Health Research Ethics (NVK-18038012).

Data analysis of DBDS participants within this study was performed under the protocol "Genetics of healthy ageing and specific diseases among blood donors - a GWA under the Danish Blood Donor Study" approved by the Danish Data Protection Agency (P-2019-99) and the National Committee on Health Research Ethics (NVK-1700407) under which data on DBDS participants were obtained for this study.

Funding:

**Study**	**Lead investigator**	**Award number**	**Funder**	**Country**

deCODE | Thorgeirsson T | PMID 29700475 | analysis code: mdd_deCODE.eur.hg19.DEPALL_FINAL_WHEAD

Cohort Description:
deCODE genetics (www.decode.com) has, since the year 1996, gathered genotypic and medical data from more than 160,000 volunteer participants in Iceland, comprising well over half of the adult population. Recruitment has been through various projects aimed at specific common disorders, as well as through wider recruitment strategies aimed at studying overall health. Cases (N=20,340) and controls (N=331,321) used in the study of depression were all Icelandic and were recruited from all over Iceland.

Case Ascertainment:
Most of the diagnoses were made by clinicians at the Landspitali University Hospital (1987-2019) and were obtained from electronic health records (ICD-10: F32*, F33*, and F41.2, ICD-9: 296.1, 300.4, and 311). Additional cases (N<1650) were identified through recruitment for studies of anxiety, depression, smoking, and substance use disorders. The recruitment process included random screening of approximately 20,000 individuals using the HADS and the SSQ questionnaires. High scorers were subsequently invited to semi-structured interviews (CIDI, SSAGA) conducted between the years 2000 and 2010 and reviewed by a psychiatrist or a psychologist assigning depression status using ICD or DSM criteria. Subjects ever diagnosed with bipolar disorder were excluded. For patients, diagnoses were assigned according to DSM-III or ICD-10 criteria.

Control Ascertainment:
Controls were recruited as a part of various genetic programs at deCODE. The controls were not specifically screened for psychiatric conditions, but subjects ever diagnosed with depression were excluded.

Genotyping, QC, Imputation:
The Icelandic chip-typed samples (N~166,000) were assayed with the Illumina HumanHap300, HumanCNV370, HumanHap610, HumanHap1M, HumanHap660, Omni-1, Omni 2.5 or Omni Express bead chips at deCODE genetics.

Genotypes of single nucleotide polymorphisms (SNPs) and insertions/deletions (indels) were identified and called jointly by Graphtyper (PMID: 28945251). All samples with a call rate below 98% were excluded from the analysis, and individual SNPs were excluded if there was significant deviation from Hardy-Weinberg equilibrium in the controls, if they produced an excessive inheritance error rate, or if there was substantial difference in allele frequency between chip types. Over 60,000 Icelandic samples have been WGS using GAIIx, HiSeq,X, and NovaSeq Illumina technology. Sample preparation, sequencing methods, alignment and BAM file generation have been described in detail elsewhere (PMID: 25977816). Two types of imputations from sequence data were performed; into SNP-typed individuals after long-range phasing (PMID: 19165921) using a method based on IMPUTE (PMID: 17572673), followed by a familial imputation estimating genotype probabilities for about 290,000 un-typed relatives of SNP-typed individuals.

The details of the association analysis approach have also been described in detail elsewhere (PMID: 25977816). Briefly, logistic regression was used to test for association between SNPs and depression, treating depression status as the response and expected genotype counts from imputation or allele counts from direct genotyping as predictor, adjusting for sex, age (or age at death), county of origin, chip-typed and/or sequence status, and an indicator function for the overlap of the lifetime of the individual with the time span of phenotype collection. Testing was performed using the likelihood ratio statistic. To account for confounding biases such as relatedness and stratification within the case and control sample sets we used LD score regression (PMID: 25642630) to estimate the correction factor for test statistic inflation due to these biases (CF = 1.36). P values were adjusted accordingly. Summary statistics, based on data for 20,340 cases and 331,329 controls, were filtered on

MAF ≥ 1% and imputation information > 0.8, and contained information on ~8.84 million variants.

Ethics statement:
All Icelandic studies were approved by the Icelandic National Bioethics Committee and the Data Protection Authority. All subjects signed informed consents prior to donating samples for the genetic study. Personal identifiers of the patients and biological samples were encrypted by a third-party system overseen by the Icelandic Data Protection Authority. Approval for the study of depression was granted by the Icelandic Data Protection Authority and the National Bioethics Committee of Iceland (Approval no: VSNb2016040011/03.01).

Funding:

Study	Lead investigator	Award number	Funder	Country
painFACT	Thorgeir E. Thorgeirsson	H2020-2020-848099	EC	Iceland

Estonian Biobank (EstBB) | Lehto K | PMID 24518929; 28401899 | analysis code: mdd_ESTBB.eur.hg19.EstBB

Cohort Description:
The Estonian Biobank (EstBB) is a population-based cohort with a rich variety of phenotypic and health-related information collected for each participant. At recruitment, participants have signed a consent to allow follow-up linkage of their electronic health records (EHR), thereby providing a longitudinal collection of phenotypic information. The health data of the participants is continuously updated through periodical linking with records from the national Health Insurance Fund Treatment Bills (from 2004 onwards), Tartu University Hospital (from 2008), and North Estonia Medical Center (from 2005). For every participant, diagnoses are stored in ICD-10 coding and drug dispensing data contains drug ATC codes, prescription status and purchase date (if available).

Case Ascertainment:
The cases were defined as participants with the following ICD-10 codes in their EHRs: F32*, F33* or F41.2, while excluding F20*–F29*, F30* and F31* codes. This resulted in 35,473 cases with European ancestry for the GWAS.

Control Ascertainment:
Among the controls, the participants with F32*, F33*, F41.2, F20*–F29*, F30*, or F31* codes, or N06A* prescriptions (at least 2 purchases), or those without any EHR data were excluded. This resulted in 91,301 controls with European ancestry for the GWAS.

Genotyping, QC, Imputation:
The samples from the Estonian Biobank have been genotyped at the Genotyping Core Facility of the Institute of Genomics, University of Tartu using the Global Screening Array (GSAv1.0, GSAv2.0, and GSAv2.0_EST) from Illumina. Altogether 155,772 samples have been genotyped and PLINK format files exported using GenomeStudio v2.0.4. Individuals were excluded from the analysis if their call-rate was <95% or if the sex defined based on heterozygosity of the X chromosome did not match the sex in the phenotype data. Variants were excluded if the call-rate was < 95% and HWE p-value <1e-4 (autosomal variants only). Variant positions were updated to genome build 37 and all alleles were switched to the TOP strand using tools and reference files provided at https://www.well.ox.ac.uk/~wrayner/strand/. After QC the dataset contained 154,201 samples for imputation. Before imputation variants with MAF<1% and indels were removed. Prephasing was done using the Eagle v2.3 software. The number of conditioning haplotypes Eagle2 uses when phasing each sample was set to: --Kpbwt=20000. Imputation was done using Beagle v.28Sep18.793 with effective population size ne=20,000. An Estonian population specific imputation reference of 2,297 WGS samples was used. The analysis was performed using the SAIGE software, including related individuals and adjusting for the first 10 principal components (PCs) of the genotype matrix, as well as for birth year, birth year squared and sex.

Ethics statement:
At recruitment, participants have signed a consent to allow follow-up linkage of their electronic health records (EHR), thereby providing a longitudinal collection of phenotypic information. Ethical approval was obtained from the Estonian Committee on Bioethics and Human Research (Decision nr: 1.1-12/624).

Funding:

**Study**	**Lead investigator**	**Award number**	**Funder**	**Country**
EstBB	Lili Milani	2014-	European Union	Estonia

		2020.4.01.15-0012	through the European Regional Development Fund	
EstBB	Lili Milani (partner PI)	847776, 964874	European Commission (Horizon2020)	Estonia
EstBB	Kelli Lehto	847776	European Commission (Horizon2020)	Estonia
EstBB	Lili Milani	PRG184	Estonian Research Council	Estonia
EstBB	Kelli Lehto	PSG615	Estonian Research Council	Estonia

Extended Cohort for E-health, Environment and DNA (EXCEED) | Tobin M | PMID 31062032 | analysis code: mdd_EXCEED.eur.hg19.202010

Cohort Description:
EXCEED (Extended Cohort for E-heath, Environment and DNA) is a population-based longitudinal cohort study of 11,000 adults based predominantly in the East Midlands, UK. Recruitment started in 2013 via GP surgeries and smoking cessation clinics and is ongoing with sign up via www.exceed,org.uk. Participants consent to provide a DNA sample, electronic healthcare data linkage and recall for further study by genotype or phenotype. EXCEED has saliva for DNA, serum for metabolomic and proteomic analysis. COVID anti-nucleocapsid and anti-spike serology. Genome-wide genotyping will shortly be available on the entire cohort.

Case Ascertainment:
MDD cases were defined using Clinical Terms Version 3 (CTV3) codes for any unipolar depressive disorder in the primary care records. At least two diagnostic codes were required to reduce the risk of misclassification. MDD cases were excluded if they also had a diagnostic code for bipolar disorders, psychotic disorders, or substance use-related disorders. For the GWAS, MDD cases were excluded based on relatedness (2nd degree relatives or close) and genetic ancestry (European ancestry only), leaving a total of 580 MDD cases.

Control Ascertainment:
Controls were individuals who had no diagnostic codes for depressive disorders, bipolar disorders, psychotic disorders, or substance use-related disorders, and no antidepressant prescription code (CTV3 code: x000f and all child codes). For the GWAS, controls were also excluded base don relatedness and genetic ancestry as above, leaving a total of 2071 controls.

Genotyping, QC, Imputation:
Array(s): Affymetrix UK Biobank Axiom Array
Quality control (QC): Genotype calling was performed using the apt-genotype-axiom command line tool from the Analysis Power Tools (APT) suite of applications. Following the workflow set out in the "Axiom Genotyping Solution Data Analysis User Guide" (last accessed: October 14th, 2022), samples were excluded based on Dish QC (DQC) <0.82 and sample call rate <97% (calculated following an initial round of genotype calling based on a subset of high-quality probe sets). Additional samples were excluded based on sample call rate <95%, ancestry-adjusted heterozygosity rate >6 standard deviations from the overall mean heterozygosity rate, a mismatch between clinical sex and genotypic sex, and duplicates (IBD score >0.8). For variant QC, we initially used the SNPolisher R package, a package that includes a set of functions used to evaluate the quality of the genotype clusters for each variant and then classifies the variant according to the cluster properties. We excluded variants that were categorized as "CallRateBelowThreshold", "OffTargetVariant" or "Other". After this, we excluded variants that showed evidence of a significant plate effect, and variants that had a call rate <95%, minor allele frequency <1% and/or a Hardy-Weinberg P-value <1×10-6.
Data were available for 5216 individuals and 636,586 variants after quality control, with imputation to the Haplotype Reference Consortium (HRC) r1.1 panel. A post-imputation checking tool (https://www.well.ox.ac.uk/~wrayner/tools/Post-Imputation.html) was used to verify the imputed data.

Ethics statement:
At recruitment, participants sign a consent form to allow follow up via electronic healthcare records as well as use of samples and data for further research by approved researchers which could include researchers in other countries and commercial companies developing new treatments. EXCEED has been reviewed and approved by the East Midlands – Leicester Central Research Ethics Committee (13/EM/0226).

Funding:
The study is supported by the University of Leicester, the National Institute for Health and Care Research (NIHR) Leicester Biomedical Research Centre, by Wellcome (202849) and by cohort access fees from studies funded by the Medical Research Council (MRC) BBRSC, NIHR, the UK Space Agency and GSK. It was previously supported by MRC grant G0902313. The views expressed are those of the author(s) and not necessarily those of the NIHR or the Department of Health and Social Care.

Study	Lead investigator	Award number	Funder	Country
EXCEED	Professor Martin D. Tobin	202849	Wellcome Trust	UK
EXCEED	Professor Martin D. Tobin	G0902313	MRC	UK

FinnGen | Kaprio J | PMID 36653562 | analysis code: mdd_FinnGen.eur.hg19.R5_ 18032020

Cohort Description:
Population isolates such as Finland provide benefits in genetic studies because the allelic spectrum of damaging alleles in any gene is often concentrated on a small number of low-frequency variants (0.1% ≤ minor allele frequency < 5%), which survived the founding bottleneck, as opposed to being distributed over a much larger number of ultra--rare variants. While this advantage is well-- established in Mendelian genetics, its value in common disease genetics has been less explored. FinnGen aims to study the genome and national health register data of 500,000 Finns by 2023. Given the relatively high median age of participants (63 years) and dominance of hospital-based recruitment, FinnGen is enriched for many disease endpoints often underrepresented in population-based studies (e.g., rarer immune-mediated diseases and late onset degenerative and ophthalmologic endpoints).

Case Ascertainment:
In Finland, similar to the other Nordic countries, there are nationwide electronic health registers originally established primarily for administrative purposes to monitor the usage of healthcare nationwide and over the lifespan of each Finnish resident. These registers have almost complete coverage of major health-related events, such as hospitalizations, prescription drug purchases (not including hospital administered medications), medical procedures or deaths with a history of data collection spanning more than 50 years. Health register based phenotypes ("endpoints") were created by combining data (mainly using International Classification of Diseases (ICD) and Anatomical Chemical Therapeutic (ACT) classification codes) from one or more nationwide heath registers. FinnGen endpoints can be browsed at https://r5.risteys.finngen.fi/. The inclusion criteria for cases were ICD-10 MDD (F32, F33), ICD-9 MDD (296.1, 296.8), or ICD-8 MDD (790.20, 298.0) in Hospital Discharge Registry or Cause of Death Registry.

Control Ascertainment:
The exclusion criteria for controls were any mood disorder in Hospital Discharge Registry or Cause of Death Registry (ICD-10: F30, F31, F32, F33, F34, F38, F39; ICD-9: 296[2-7], 300.4; ICD-8: 296, 300.4, 296.99)

Genotyping, QC, Imputation:
Samples were genotyped with Illumina (Illumina Inc., San Diego, CA, USA) and Affymetrix arrays (Thermo Fisher Scientific, Santa Clara, CA, USA). Genotype calls were made with GenCall and zCall algorithms for Illumina and AxiomGT1 algorithm for Affymetrix data. Chip genotyping data produced with previous chip platforms and reference genome builds were lifted over to build version 38 (GRCh38/hg38) following the protocol described here: dx.doi.org/10.17504/protocols.io.nqtddwn. In sample-wise QC, individuals with ambiguous sex, high genotype missingness (>5%), excess heterozygosity (+-4 standard deviations). In variant-wise QCvariants with high missingness (> 2%), low Hardy–Weinberg Equilibrium (HWE) P-value (< 1e-6) and minor allele count, minor allele count (MAC) < 3 were removed. Chip-genotyped samples were pre-phased with Eagle 2.3.5 (https://data.broadinstitute.org/alkesgroup/Eagle/) with the default parameters, except the number of conditioning haplotypes was set to 20,000.

The population-specific Sequencing Initiative Suomi (SISu) v3 imputation reference panel was developed by using high-coverage (25-30x) whole-genome sequencing (WGS) data for 3,775 Finnish individuals. Briefly, the variant callset was produced with GATK HaplotypeCaller algorithm by following GATK best-practices for variant calling. Genotype-, sample- and variant-wise QC was performed using the Hail framework (https://github.com/hail-is/hail) v0.1 and the resulting high-quality WGS data were phased (see Supplementary Methods). Genotype imputation was carried out by using the SISu v3

reference panel with Beagle 4.1 (version 08Jun17.d8b, https://faculty.washington.edu/browning/beagle/b4_1.html) as described in the following protocol: dx.doi.org/10.17504/protocols.io.nmndc5e. Post-imputation QC involved non-reference concordance analyses, checking expected conformity of the imputation INFO-values distribution, MAF differences between the target dataset and the imputation reference panel and checking chromosomal continuity of the imputed genotype calls. After these steps, variants with imputation INFO score < 0.6 or MAF < 0.0001 were excluded.

Ethics statement:

Patients and control subjects in FinnGen provided informed consent for biobank research, based on the Finnish Biobank Act. Alternatively, separate research cohorts, collected prior the Finnish Biobank Act came into effect (in September 2013) and start of FinnGen (August 2017), were collected based on study-specific consents and later transferred to the Finnish biobanks after approval by Fimea, the National Supervisory Authority for Welfare and Health. Recruitment protocols followed the biobank protocols approved by Fimea.
The Coordinating Ethics Committee of the Hospital District of Helsinki and Uusimaa (HUS) approved the FinnGen study protocol Nr HUS/990/2017.

Funding:

The FinnGen project is funded by two grants from Business Finland (HUS 4685/31/2016 and UH 4386/31/2016) and the following industry partners: AbbVie Inc., AstraZeneca UK Ltd, Biogen MA Inc., Bristol Myers Squibb (and Celgene Corporation & Celgene International II Sarl), Genentech Inc., Merck Sharp & Dohme Corp., Pfizer Inc., GlaxoSmithKline Intellectual Property Development Ltd., Sanofi US Services Inc., Maze Therapeutics Inc., Janssen Biotech Inc, Novartis AG, and Boehringer Ingelheim International GmbH.

**Study**	**Lead investigator**	**Award number**	**Funder**	**Country**
FinnGen	Aarno Palotie	HUS 4685/31/2016	Business Finland	Finland
FinnGen	Aarno Palotie	UH 4386/31/2016	Business Finland	Finland

Generation Scotland: Scottish Family Health Study (GenScot) | McIntosh A; Porteous D | PMID 22786799; 26571028 | analysis code: mdd_GenScot.eur.hg19.SCID_0721a

Cohort Description:
GS:SFHS comprises 24,000 adults (18+) from 7,000 families recruited from the general population of Scotland 2006-2011. 90% of the cohort attended a clinic in Dundee, Glasgow or Aberdeen where samples were taken and various tests carried out. The remainder returned a postal questionnaire and saliva sample for DNA. The study's main aim is to investigate the genetics of common diseases, with a particular focus on mental health.
Further details here: https://pubmed.ncbi.nlm.nih.gov/22786799/

Case Ascertainment:
Volunteers were administered the SCID-IV structured interview by a trained researcher to diagnose serious mood disorders, including MDD. Individuals meeting criteria for bipolar disorder or who self-declared a diagnosis of schizophrenia were excluded.

Control Ascertainment:
Controls were identified as those who answered no to the screening questions from the SCID, or those who screened positive but were subsequently found not to fulfill criteria for a lifetime diagnosis of MDD, bipolar disorder or self-declared schizophrenia.

Genotyping, QC, Imputation:
GWAS data was obtained using the Illumina OmniExpress array, and imputed using the Haplotype Research Consortium (HRC) dataset. Further details of methods here https://pubmed.ncbi.nlm.nih.gov/28270201/

Ethics statement:
Ethical approval for the original data collection was obtained from the Tayside Committee on Medical Research Ethics A (ref 05/S1401/89). Generation Scotland is currently approved as a Research Tissue Bank by the East of Scotland Research Ethics Service (ref 20/ES/0021).

Funding:

**Study**	**Lead investigator**	**Award number**	**Funder**	**Country**
GenScot	AM McIntosh, T-K Clarke, D Porteous	104036/Z/14/Z	Wellcome Trust	UK
GenScot	D Porteous	CZD/16/6	Chief Scientist Office	UK
GenScot	D Porteous	HR03006	Scottish Funding Council	UK

Genetic Epidemiology Research on Aging (GERA) | Schaefer C | PMID 26092718; 26092716 | analysis code: mdd_GERA.eur.hg19.0915a_mds5; GERA_DIV

Cohort Description:

Individual data of the GERA study was accessed via dbGaP under project ID of 18933.

The GERA Cohort is a subsample of the longitudinal, population-based, and multiethnic Kaiser Permanente Research Program on Genes, Environment and Health (RPGEH) cohort. The GERA Cohort was developed from a mailed survey sent to all adult members of Kaiser Permanente Medical Care Plan, Northern California Region (KPNC) who had been members for two years or more in 2007. KPNC is an integrated health care delivery system with a population of about 3.3 million people in northern California. The membership of KPNC is representative of the general population in the 14 county area in which facilities are located, although the membership is underrepresented for the extremes of income at both ends of the spectrum. The RPGEH utilizes the longitudinal electronic health records of KPNC to obtain clinical, laboratory, imaging and pharmacy information on all cohort members, to which personal demographic, behavioral and health characteristics have been added through member surveys. GERA participants were also asked to self-report their race, ethnicity, nationality and religion in order to maximize the diversity of the cohort. As a result, the final cohort was formed by 19% non-European individuals and 81% randomly drawn non-Hispanic white individuals."

All survey respondents were contacted and asked to complete a consent form; those who completed consent forms were asked to provide a saliva sample. Additional participants were added to the RPGEH through inclusion of the Northern California sample of the California Men's Health Study (CMHS) cohort of about 40,000 men from KPNC, ages 45–69 years old at the time of the CMHS survey in 2002–2003. The CMHS participants contributed about 15,400 saliva samples to the RPGEH and were eligible for inclusion in the GERA Cohort. CMHS participants were included according to the same sampling design as for the RPGEH cohort as a whole. Specifically, all minority participants were selected for inclusion in order to maximize representation of minorities in the GERA Cohort, and Non-Hispanic White participants were selected at random to complete the sample of 110,266 GERA Cohort participants.

Participants provided broad consent and a sample of saliva or blood for use in studies of genetic and environmental factors in health and disease. Although the original consent form signed by RPGEH participants provided for sharing of de-identified data with collaborators, it did not provide explicit consent for placement of participants' data in databases with access controlled by NIH or other third parties. To ensure all participants were appropriately consented for placement of data in dbGaP (https://www.ncbi.nlm.nih.gov/projects/gap/cgi-bin/study.cgi?study_id=phs000674.v3.p3), the RPGEH mailed new consent forms that included a section explaining dbGaP to all participants. Approximately 77% of participants returned the signed, updated consent form. After excluding samples that failed genotyping and small numbers of invalid or duplicate results, the total number of appropriately consented participants with data for deposition in dbGaP is 78,419.

Data on the occurrence of health conditions in participants in the GERA Cohort have been derived from summarizing ICD-9 coded diagnoses in Kaiser Permanente's electronic medical records. An algorithm that aggregates specific ICD-9 codes into appropriate diagnostic groups for selected conditions is applied to outpatient and inpatient databases. The criterion for counting a condition as "present" for a participant is the occurrence of two or more diagnoses within a diagnostic category occurring on separate days. Two or more is used as the criterion in order to reduce false positives due to mistakes or rule-out diagnoses. For outpatient visits occurring during the period 1995 to 2006, diagnoses were assigned by the treating physician who endorsed specific diagnoses on an optically scanned list that varied by specialty. Beginning in 2006 with the advent of an integrated, fully electronic medical record, outpatient diagnoses are made by physicians/ providers using a pull down menu. Discharge diagnoses from inpatient stays are specified by physicians and coded by specially trained coders.

Case Ascertainment:
Cases consisted of individuals with electronic medical records (EMR) documenting an ICD-9 diagnosis of MDD on two or more separate days. Cases with either an ICD-9 diagnosis of bipolar disorder or schizophrenia in their EMR were excluded.

Control Ascertainment:
Controls consisted of individuals with no ICD-9 diagnosis of MDD in their EMR. Controls with ICD-9 diagnoses for insomnia, stress, irritable bowel syndrome, bipolar disorder, non-affective psychosis, substance use disorder, post-traumatic stress disorder, any eating disorder or anxiety disorder were excluded.

Genotyping, QC, Imputation:
Array(s): Custom Affymetrix Axiom arrays

Genotyping & QC:
High-density genotyping was conducted at the University of California San Francisco using custom designed Affymetrix Axiom arrays, as described in Hoffmann et al. (PMID 21565264; 21903159). To maximize genome-wide coverage of common and less common variants, four specific arrays were designed for individuals of Non-Hispanic White (EUR), East Asian (EAS), African-American (AFR), and Hispanic/Latinx (HIS) ancestry. There was broad overlap among the SNPs on the arrays, which were designed using a hybrid greedy imputation algorithm (Hoffmann et al., PMID 21903159) applied to genotype information validated by Affymetrix from the 1000 Genomes Project. However, in order to capture low frequency variants specific to particular ancestry groups, SNP content varies between arrays. A more detailed description of the process of genotyping and results is found in Kvale et al., 2015 (PMID 26092718). Description of the analyses of population structure and development of principal components for adjustment of population structure is provided in Banda et al., 2015 (PMID 26092716).

Imputation:
Over 31 million variants were imputed. Imputation was performed on an array-wise basis. Genotypes were first pre-phased with SHAPEIT v2.5, with cryptic relatives included to improve phasing. The 1000 Genomes Project (October 2014 release with 2,504 samples; singletons removed) was used as a cosmopolitan reference panel, and data were imputed with IMPUTE2 v2.3.1 (Hoffmann et al., 2015 (PMID: 26034056)). In general, SNPs in the genotyped dataset were included in the imputed dataset.

Ethics statement:
To ensure all participants were appropriately consented for placement of data in dbGaP, the RPGEH mailed new consent forms that included a section explaining dbGaP to all participants. Approximately 77% of participants returned the signed, updated consent form. After excluding samples that failed genotyping and small numbers of invalid or duplicate results, the total number of appropriately consented participants with data for deposition in dbGaP is 78,419. All study procedures were approved by the Institutional Review Board of the Kaiser Foundation Research Institute (https://bio-protocol.org/exchange/minidetail?type=30&id=9059082).

Funding:
The Resource for Genetic Epidemiology Research on Aging (GERA) Cohort was created by a RC2 "Grand Opportunity" grant that was awarded to the Kaiser Permanente Research Program on Genes, Environment, and Health (RPGEH) and the UCSF Institute of Human Genetics (AG036607; Schaefer/Risch, PIs). In addition to the NIH funding of the RC2 project that supported the genotyping, the RPGEH has been supported by grants from philanthropic foundations, including the Wayne and Gladys Valley Foundation, the Ellison Medical Foundation, and the Robert Wood Johnson Foundation, as well as support from Kaiser

Permanente, for work on disease registries, cohort enrollment, survey collection, and collection of biospecimens.

The Trøndelag Health Study (HUNT)| Winsvold B; Hveem K; Zwart JA | PMID 22879362; 30804566; 35578897 | analysis code: mdd_HUNT.eur.hg19.gp_hospital_metacarpa_20190625

Cohort Description:
The Trøndelag Health Study (HUNT) consists of Four different population-based health surveys conducted in the county of Trøndelag, Norway over approximately 20 years (HUNT1 [1984–1986], HUNT2 [1995–1997], HUNT3 [2006–2008] and HUNT4[2017-2019]). At each survey, the entire adult population (≥20 years) was invited to participate by completing questionnaires, attending clinical examinations and interviews. Participation rates in HUNT1, HUNT2 and HUNT3 were 89.4% (*n*=77,212), 69.5% (*n*=65,237), and 54.1% (*n*=50,807), respectively. Taken together, the study included more than 120,000 different individuals from Nord-Trøndelag County. Biological samples including DNA have been collected for approximately 70,000 participants. The HUNT Study has been described in more detail elsewhere (PMID 22879362; 35578897). For the present study, we included participants from HUNT2 and HUNT3.

Case Ascertainment:
Major depressive disorder (MDD) was defined by the presence of either: 1) “Hospital-based MDD”: at least one hospital contact due to ICD-10 F32 depressive episode or ICD-10 F33 recurrent depressive disorder; or 2) “Lifetime history of MDD” three or more positive answers to the following five questions: "During your life, have there been periods of two consecutive weeks or more when: You felt depressed, sad and down (must always be answered positive to qualify as a case)? You had appetite problems or ate too little? You felt weak (adynamic) or lacked energy? You really reproached yourself and felt worthless? You had problems concentrating or had difficulty making decisions?". These five questions reflect key depressive symptoms and are previously used in a twin study on the reliability and heritability of MDD (PMID 8215812).

Control Ascertainment:
We defined general criteria for controls for all our GWAS on psychiatric phenotypes. These are 1) age >40 years by 2017, 2) no ICD-9 or ICD-10 diagnoses of psychiatric disorders in local hospital registries (separate table, not attached here), 3) no ICPC-2 diagnoses of psychiatric disorders in primary care doctor registries, 4) no self-reported psychiatric disorders in the HUNT questionnaires, 5) no self-reported daily use of antidepressants, relaxants, or sleeping medications in the HUNT questionnaires, and 6) HADS-A and HADS-D <11. Using this definition, 2/3 of the population fulfilled criteria to be included as controls.

Genotyping, QC, Imputation:
Genotyping and QC:
Genotyping was performed with the HumanCoreExome array 24v (n=58,997), 12v1.0 (n=7,872), and 12v1.1 (n=4,992).

In total, DNA from 71,860 HUNT samples was genotyped using one of three different Illumina HumanCoreExome arrays (HumanCoreExome12 v1.0, HumanCoreExome12 v1.1 and UM HUNT Biobank v1.0). Samples that failed to reach a 99% call rate, had contamination > 2.5% as estimated with BAF Regress (PMID 23103226), large chromosomal copy number variants, lower call rate of a technical duplicate pair and twins, gonosomal constellations other than XX and XY, or whose inferred sex contradicted the reported gender, were excluded. Samples that passed quality control were analyzed in a second round of genotype calling following the Genome Studio quality control protocol described elsewhere (PMID 25321409). Genomic position, strand orientation and the reference allele of genotyped variants were determined by aligning their probe sequences against the human genome (Genome Reference Consortium Human genome build 37 and revised Cambridge Reference Sequence of the human mitochondrial DNA; http://genome.ucsc.edu) using BLAT (PMID 22955616). PLINK v1.90 (PMID 25722852) was then used to exclude variants if their probe sequences

could not be perfectly mapped, cluster separation was < 0.3, Gentrain score < 0.15, showed deviations from Hardy Weinberg equilibrium in unrelated samples of European ancestry with p-value < 0.0001), had a call rate < 99%, or another assay with higher call rate genotyped the same variant.

Ancestry of all samples was inferred by projecting all genotyped samples into the space of the principal components of the Human Genome Diversity Project (HGDP) reference panel (938 unrelated individuals; downloaded from http://csg.sph.umich.edu/chaolong/LASER/, PMID 24633160, 18292342) using PLINK. Recent European ancestry was defined as samples that fell into an ellipsoid spanning exclusively European populations of the HGDP panel.

The different arrays were harmonized by reducing to a set of overlapping variants and excluding variants that showed frequency differences > 15% between data sets, or that were monomorphic in one and had MAF > 1% in another data set. The resulting genotype data were phased using Eagle2 v2.3 47 (PMID 27694958).

Imputation:

Imputation was performed on the 69,716 samples of recent European ancestry using Minimac3 (v2.0.1, http://genome.sph.umich.edu/wiki/Minimac3, PMID 27571263) with default settings (2.5 Mb reference based chunking with 500kb windows) and a customized Haplotype Reference consortium release 1.1 (HRC v1.1) for autosomal variants and HRC v1.1 for chromosome X variants (PMID 27548312). The customized reference panel represented the merged panel of two reciprocally imputed reference panels: (1) 2,201 low-coverage whole-genome sequences samples from the HUNT study and (2) HRC v1.1 with 1,023 HUNT WGS samples removed before merging. We excluded imputed variants with Rsq<0.3 or minor allele count <1, resulting in 24.2 million well-imputed variants.

GWAS:

Association analyses were conducted using SAIGE (PMID 30104761), which uses a generalized mixed model to account for sample relatedness and cryptic population structure. A linear mixed regression model was applied to analyze genotyped variants (when available) or imputed variants (dosages) on depression, including the first 10 principal components and batch as covariates. We assumed an additive allelic effect, and included only variants with MAC>3 in the analysis. Analyses for "Hospital-based MDD" (4,640 cases and 17,014 controls) and "Lifetime history of MDD" (7,018 cases and 25,521 controls) were based on non-overlapping samples and were run as separate GWAS before being entered separately in the meta-analysis.

Ethics statement:

Written informed consent was obtained from each study participant. This study was approved by the Regional Committee for Medical and Health Research Ethics (ref. 2015/575 REK midt).

Funding:

**Study**	**Lead investigator**	**Award number**	**Funder**	**Country**
DecipherPain	Bendik S Winsvold	2020034	South-Eastern Norway Regional Health Authority	Norway

Acknowledgement:
The Trøndelag Health Study (HUNT) is a collaboration between HUNT Research Centre (Faculty of Medicine and Health Sciences, Norwegian University of Science and Technology NTNU), Trøndelag County Council, Central Norway Regional Health Authority, and the Norwegian Institute of Public Health. The genotyping was financed by the National Institute of health (NIH), University of Michigan, The Norwegian Research council, and Central Norway Regional Health Authority and the Faculty of Medicine and Health Sciences, Norwegian University of Science and Technology (NTNU). The genotype quality control and imputation has been conducted by the K.G. Jebsen center for genetic epidemiology, Department of public health and nursing, Faculty of medicine and health sciences, Norwegian University of Science and Technology (NTNU).

iPSYCH2012 MDD cohort (iPSYCH2012) | Børglum A; Als T; Mors O; Mortensen PB; Nordentoft M; Werge T; Hougaard DM | PMID 28924187; 29700475 | analysis code: mdd_iPSYCH.eur.hg19.2012_HRC

Cohort Description:
The iPSYCH2012 MDD cohort is part of a population-based case-cohort sample extracted from a baseline cohort consisting of all children born in Denmark between May 1, 1981, and December 31, 2008, who were alive and resided in Denmark on their one-year birthday, and who have a known mother. The samples were linked by using the unique national personal identification number to the Danish Newborn Screening Biobank (DNSB) at Statens Serum Institut (SSI), where DNA was extracted from Guthrie cards, and whole-genome amplification was performed in triplicate. Genotyping of the iPSYCH2012 sample was performed at the Broad Institute of Harvard and MIT (Cambridge, MA, USA) with PsychChip arrays from Illumina according to the manufacturer's instructions. Genotype calling of markers with MAF >0.01 was performed by merging call sets from GenCall and Birdseed , and less frequent variants were called with zCall. Genotyping and data processing were carried out in 23 waves. A previous version of MDD iPSYCH2012, imputed with 1000 genomes has previously been shared with PGC-MDD. All analyses of the iPSYCH sample were performed at the secured national GenomeDK high-performance computing cluster in Denmark.

Case Ascertainment:
MDD cases were identified from the Danish Psychiatric Central Research Register (DPCRR), which includes data on all individuals treated in Denmark at psychiatric hospitals (from 1969 onward) as well as at outpatient psychiatric clinics (from 1995 onward). Subjects were diagnosed with MDD in 2015 or earlier by a psychiatrist according to ICD-10. For controls, we selected a random sample excluding those with an MDD diagnosis. All individuals diagnosed with a BP diagnosis were excluded.

Control Ascertainment:
For controls, we selected a random sample excluding those with an MDD and/or BP diagnosis.

Genotyping, QC, Imputation:
Genotyping:
Genotyping in iPSYCH2012 was done using the PsychArray V1.0 (Illumina, San Diego, California). Genotyping of iPSYCH2015i was done using the Global Screening Array v2 (GSA2) with a Multi disease drop in (Illumina, San Diego, California). Since the two samples were genotyped on different platforms, they were QCed and imputed separately. The provided summary are independent analyses of each subsample in order to keep maximum flexibility in subsequent analyses. A previous version of MDD iPSYCH2012, imputed with 1000 genomes has previously been shared with PGC-MDD.

RICOPILI pre-imputation QC:
We performed a near-default RICOPILI QC using the parameters in order below:
First SNPs with a call rate <0.95 were removed. Then sample QC was run keeping individuals with call rate in cases or controls ≥0.95 and an autosomal heterozygosity deviation FHET within +/-0.20 in cases or controls, and flagging individuals where stated sex was not consistent with sex derived from genotypes (implementing corrections from SSI). Subsequently marker QC was run keeping markers with call rate ≥0.98, missing difference ≤0.02 between cases and controls, with MAF≥0.01 (in particular the are not invariant), Hardy-Weinberg equilibrium (HWE) in controls p-value≥1e-06 and HWE in cases p-value≥1e-10. See https://sites.google.com/a/broadinstitute.org/ricopili/preimputation-qc for further details.

Batch QC:
The effects of three batch variables on marker genotypes were tested in iPSYCH2012 (ArrayPlate.ID, PreProc.Plate and wave) and iPSYCH2015i separately (Array.Batch,

ArrayPlate.ID and PreProc.Plate). This was done using relatedness- pruned dataset with ancestry outliers removed in order to avoid removal of markers where batch effects were caused by population structure or cryptic relatedness rather than genotype artefacts.

Pairwise relatedness coefficients (PI_HAT) were estimated with plink using a LD pruned and MAF filtered set of SNPs (snps-only, window size=5000, step size=300, r2<0.05, MAF>0.05). Principal Component Analysis was conducted using the same set of LD pruned and MAF filtered SNPs, with random removal of one member of each pair with a relatedness coefficient higher than 0.2. Eigenvectors were inferred using EIGENSOFT version 6.1.4 on the relatedness-pruned set of individuals, and subsequently projecting all individuals onto those eigenvectors based on their genotypes.

Individuals with all four grandparents born in Denmark were used as a reference for constructing a 3-dimensional ellipsoid using principal components 1, 2 and 3 with a radius of 5 standard deviations from the mean. Individuals located outside this ellipsoid were removed prior to the testing for batch effects.

Each genotyped marker was tested for association with each batch versus the remaining batches pooled. This was done for the following batch-variables: ArrayPlate.ID, PreProc.Plate and wave for iPSYCH2012 and Array.Batch, ArrayPlate.ID and PreProc.Plate for iPSYCH2015i. Batch-association testing was conducted using plink v1.90b4. Weir and Cockerham's FST was also estimated for each marker and batch value.

The exclusion of SNPs strongly associated with any of the batch-variables were based on their minimum P-value across all associations per variable. The cut-off for the wave and Array. Batch was min(p)<2e-10 and for PreProc.Plate and ArrayPlate.ID min(p)<2e-12, based on a Bonferroni correction for the number of markers tested and the number of associations done per batch-variable trying to incorporate the degree of nestedness of these.

HRC imputation and post-imputation QC:
iPSYCH2012 and iPSYCH2015i were imputed using the RICOPILI pipeline with the downloadable version of the Haplotype Reference Consortium. The imputed SNPs were LD-pruned down to a set of roughly 30k markers. Pairs of individuals were identified with PI_HAT >0.2 using plink and one individual of each such pair was excluded at random. PCA was carried out using RICOPILI. A subsample of European ancestry was selected as an ellipsoid in the space of PC1–3 centered and scaled using the mean and 8 standard deviation of the subsample whose parents and grandparents were all known to have been born in Denmark.

GWAS:
Comorbid cases with a diagnosis of bipolar disorder were excluded. Association analyses were conducted using RICOPILI, and analyses were adjusted for PC 1–10 from a PCA using the remaining subsample after removal of ancestry outliers.

Ethics statement:
The iPSYCH study was approved by the Scientific Ethics Committee in the Central Denmark Region (Case No 1-10-72-287-12) and the Danish Data Protection Agency. The Danish Scientific Ethics Committee, in accordance with Danish legislation, has, for this study, waived the need for informed consent in biomedical research based on existing biobanks.

Funding:
The iPSYCH team was supported by grants from the Lundbeck Foundation (R102-A9118, R155-2014-1724, and R248-2017-2003), NIH/NIMH (1U01MH109514-01 and 1R01MH124851-01 to ADB) and the Universities and University Hospitals of Aarhus and Copenhagen. The Danish National Biobank resource was supported by the Novo Nordisk Foundation. High-performance computer capacity for handling and statistical analysis of iPSYCH data on the GenomeDK HPC facility was provided by the Center for Genomics and Personalized Medicine (CGPM) and the Centre for Integrative Sequencing, iSEQ, Aarhus University, Denmark (grant to ADB).

iPSYCH2015i MDD cohort (iPSYCH2015i) | Børglum A; Als T; Mors O; Mortensen PB; Nordentoft M; Werge T; Hougaard DM | 37464041; https://doi.org/10.1101/2020.11.30.20237768 | analysis code: mdd_iPSYCH.eur.hg19.2015i_HRC

Cohort Description:
The iPSYCH2015i (iPS15) sample is an extension of the iPSYCH2012 (iPS12) sample, and is thus a population-based case-control-cohort extracted from the same baseline cohort consisting of all children born in Denmark between May 1, 1981, and December 31, 2008, who were alive and resided in Denmark on their one-year birthday, and who have a known mother (PMID 28924187). The samples were linked by using the unique national personal identification number to the Danish Newborn Screening Biobank (DNSB) at Statens Serum Institute (SSI), where DNA was extracted from Guthrie cards, and whole-genome amplification was performed in triplicate (PMID 21726430, 23358160). Genotyping of the iPSYCH2015i sample was performed at Statens Serum Institut (SSI, Copenhagen, Denmark) using the Global Screening Array v2 with a Multi disease drop in. All analyses of the iPSYCH sample were performed at the secured national GenomeDK high-performance computing cluster in Denmark.

Case Ascertainment:
MDD cases were identified from the Danish Psychiatric Central Research Register (DPCRR) (PMID 21775352), which includes data on all individuals treated in Denmark at psychiatric hospitals (from 1969 onward) as well as at outpatient psychiatric clinics (from 1995 onward). Subjects were diagnosed with MDD in 2015 or earlier by a psychiatrist according to ICD-10. All individuals diagnosed with a bipolar disorder diagnosis were excluded.

Control Ascertainment:
For controls, we selected a random sample excluding those with an MDD diagnosis. All individuals diagnosed with a bipolar disorder diagnosis were excluded.

Genotyping, QC, Imputation:
Array: Illumina Global Screening Array GSAMD-24v2-0 (GSA2)

RICOPILI pre-imputation QC:
We performed a near default RICOPILI QC using the parameters in order below:
First, SNPs with a call rate <0.95 were removed. Then, sample QC was run keeping individuals with call rate in cases or controls ≥0.95 and an autosomal heterozygosity deviation FHET within +/-0.20 in cases or controls, and flagging individuals where stated sex was not consistent with sex derived from genotypes (implementing corrections from SSI). Subsequently marker QC was run keeping markers with call rate ≥ 0.98, missing difference ≤ 0.02 between cases and controls, with MAF≥0.01 (in particular the are not invariant), Hardy-Weinberg equilibrium (HWE) in controls p-value≥1e-06 and HWE in cases p-value≥1e-10. See https://sites.google.com/a/broadinstitute.org/ricopili/preimputation-qc for further details.

Batch QC:

The effects of three batch variables on marker genotypes were tested in iPSYCH2012 (ArrayPlate.ID, PreProc.Plate and wave) and iPSYCH2015i separately (Array.Batch, ArrayPlate.ID and PreProc.Plate). This was done using relatedness- pruned dataset with ancestry outliers removed in order to avoid removal of markers where batch effects were caused by population structure or cryptic relatedness rather than genotype artefacts.

Pairwise relatedness coefficients (PI_HAT) were estimated with PLINK using a LD pruned and MAF filtered set of SNPs (snps-only, window size =5000, step size=300, r2<0.05, MAF>0.05). Principal Component Analysis was conducted using the same set of LD pruned and MAF filtered SNPs, with random removal of one member of each pair with a relatedness coefficient higher than 0.2. Eigenvectors were inferred using EIGENSOFT version 6.1.4 on the relatedness-pruned set of individuals, and subsequently projecting all individuals onto those eigenvectors based on their genotypes.

Individuals with all four grandparents born in Denmark were used as a reference for constructing a 3-dimensional ellipsoid using principal components 1, 2 and 3 with a radius of 5 standard deviations from the mean. Individuals located outside this ellipsoid were removed prior to the testing for batch effects. Each genotyped marker was tested for association with each batch versus the remaining batches pooled. This was done for the following batch-variables: ArrayPlate.ID, PreProc.Plate and wave for iPSYCH2012 and Array.Batch, ArrayPlate.ID and PreProc.Plate for iPSYCH2015i. Batch-association testing was conducted using PLINK v1.90b4. Weir and Cockerham's FST was also estimated for each marker and batch value. The exclusion of SNPs strongly associated with any of the batch-variables were based on their minimum P-value across all associations per variable. The cut-off for the wave and Array.Batch was min(p)<2e-10 and for PreProc.Plate and ArrayPlate.ID min(p)< 2e-12, based on a Bonferroni correction for the number of markers tested and the number of associations done per batch-variable trying to incorporate the degree of nestedness of these. After removing SNPs falling for any of the above cut-off the remaining distribution was evaluated using QQ-plots. The expected min(p) distribution was calculated using the inverse cumulative distribution of N independent distributions as suggested in supplementary of https://doi.org/10.1038/s41593-018-0320-0, N being the number batch-variable values. Reviewing these QQ-plots it is evident that despite filtering by p-value some signal from the batch-variable remains in the dataset.

HRC imputation and post imputation QC:

iPSYCH2012 and iPSYCH2015i were imputed using the RICOPILI pipeline with the downloadable version of the Haplotype Reference Consortium. The imputed SNPs were LD-pruned down to a set of roughly 30k markers. Pairs of individuals were identified with π" >0.2 using PLINK and one individual of each such pair was excluded at random. PCA was carried out using RICOPILI. A subsample of European ancestry was selected as an ellipsoid in the space of PC1–3 centered and scaled using the mean and 8 standard deviation of the subsample whose parents and grandparents were all known to have been born in Denmark.

GWAS:

Co-morbid cases with a diagnosis of bipolar disorder were excluded. Association analyses were conducted using RICOPILI, and analyses were adjusted for PC 1–10 from a PCA using the remaining subsample after removal of ancestry outliers.

Ethics statement:

The iPSYCH study was approved by the Scientific Ethics Committee in the Central Denmark Region (Case No 1-10-72-287-12) and the Danish Data Protection Agency. The Danish Scientific Ethics Committee, in accordance with Danish legislation, has, for this study, waived the need for informed consent in biomedical research based on existing biobanks.

Funding:

The iPSYCH team was supported by grants from the Lundbeck Foundation (R102-A9118, R155-2014-1724, and R248-2017-2003), NIH/NIMH (1U01MH109514-01 and 1R01MH124851-01 to ADB) and the Universities and University Hospitals of Aarhus and Copenhagen. The Danish National Biobank resource was supported by the Novo Nordisk Foundation. High-performance computer capacity for handling and statistical analysis of iPSYCH data on the GenomeDK HPC facility was provided by the Center for Genomics and Personalized Medicine (CGPM) and the Centre for Integrative Sequencing, iSEQ, Aarhus University, Denmark (grant to ADB).

LifeGene, Karolinska Internet Psychiatry Depression (iCBT) | Pedersen N; Rück C | PMID 30410065 | analysis code: mdd_lgic2.eur.hg19.202011

Cohort Description:
Cases: Between 2008 and 2016, adult patients with MDD who started iCBT at the Internet Psychiatry Clinic in Stockholm, a government-funded psychiatric clinic specializing in delivering psychologist-guided iCBT, were asked to participate in the study. The treatment center is part of the public psychiatric care provided by the Stockholm County Council. The patients were asked to donate a blood sample for DNA. The patients had either been referred to the clinic by their general practitioner or via an online self-referral system. The purpose of the cohort was to study was to study treatment outcome in relation to e.g. genetic variation.

Controls: LifeGene is a nation-wide prospective cohort consisting of 50,799 participants whereas approximately 30 000 of them are women. The study was launched in 2009 and was performed in Sweden, participants were recruited between age 18-50 who were randomly selected and then invited to include their household members. A web-based questionnaire collecting information on lifestyle, physical, mental, and social well-being was administered at baseline with annual follow-ups.

Case Ascertainment:
After an online screening, the patients came to the clinic for psychiatric assessments, including a structured diagnostic interview (Mini-International Neuropsychiatric Interview). A psychiatrist or supervised psychiatry resident performed the interview. For enrollment in the study, the patient had to meet the following requirements: fulfill the criteria in the DSM IV-TR for current MDD, be able to read and write in Swedish, and be at least 18-year-old.

The exclusion criteria were any of the following: Severe MDD combined with moderate to high risk of suicide, recent medication changes, comorbid bipolar or other psychotic disorder, unable to participate in concurrent psychotherapy, current alcohol or illicit drug abuse/dependence or communication difficulties that impact treatment.

Control Ascertainment:
Controls were selected as those who self-reported no mental health history or no mental health conditions diagnosed by a physician at baseline or any of the five follow-up.

Genotyping, QC, Imputation:
Cases:
Genotyping of cases was performed at LIFE & BRAIN GmbH (Bonn, Germany) using the Infinium Global Screening Array 1.0 BeadArray (Illumina, Inc., San Diego, CA, USA) and automated workflow according to the manufacturer's instructions. The raw data were analyzed using GenomeStudio 2.0 (Illumina, Inc.) using the Infinium cluster file (GSA-24v1-0_A1_ClusterFile.egt). A reclustering step was performed using the GenTrain 3 algorithm in Genome Studio 2.0. The GWAS data from the 964 iCBT samples were processed using the PGC Ricopili pipeline for quality control and genotype imputation with reference genomes from the 1000 Genomes Project (phase 1 version 3). Eleven samples were excluded due to sample overlap (two pairs), cryptic relatedness (two pairs with pi-hat ≥ 0.2), or poor call rate (three samples). After excluding 49 subjects due to non-European ancestry, the top 20 ancestry principal components (PC) were calculated from the best-guess imputed genotypes. Ten participants who failed to start treatment after inclusion were excluded due to missing phenotype data, resulting in a final sample total of 894.

Controls:
LifeGene controls were genotyped using the Infinium Global Screening Array 1.0 BeadArray (Illumina, Inc., San Diego, CA, USA). QC was performed using the Ricopilli pipeline and imputation was performed using the Sanger Impute Server.

Ethics statement:

The study was approved by the Regional Ethics Board in Stockholm, Sweden, 2009/1089-32/2. All participants provided written informed consent.

Funding:
For Life Gene, start-up funding has been received from Karolinska Institutet, the Stockholm County Council and the Swedish Research Council. Funding has also been obtained from the Torsten and Ragnar Söderbergs Foundation and AFA Försäkringar.

Study	Lead investigator	Award number	Funder	Country
PRiMED: Predicting Response to CBT in Mental Disorders using multimodal data and machine learning	Christian Rück	2018-02487	Swedish research council	Sweden
Predicting health outcomes and labor market marginalization after treatment in common mental disorders using Machine Learning	Christian Rück	2018-00221	Forte	Sweden

Acknowledgement:
The computations were enabled by resources provided by the Swedish National Infrastructure for Computing (SNIC) at the UPPMAX server partially funded by the Swedish Research Council through grant agreement no. 2018-05973.

Norwegian Mother, Father and Child Cohort Study (MoBa) | Reichborn-Kjennerud T; Tesli M | PMID 27063603 | analysis code: mdd_MoBa.eur.hg19.harvest12, mdd_MoBa.eur.hg19.harvest24, mdd_MoBa.eur.hg19.rotterdam1

Cohort Description:
The Norwegian Mother, Father and Child Cohort Study (MoBa) is a population-based pregnancy cohort study conducted by the Norwegian Institute of Public Health. Participants were recruited from all over Norway, at ultrasound examinations around week 17 of pregnancy, from 1999-2008. The women consented to participation in 41% of the pregnancies (https://doi.org/10.1093/ije/dyw029). The children were born between 1999 and 2009. The cohort includes approximately 114,500 children, 95,200 mothers, and 75,200 fathers. Blood samples were obtained from both parents during pregnancy and from mothers and children (umbilical cord) at birth (https://doi.org/10.5324/nje.v24i1-2.1755). Samples of DNA, RNA, whole blood, and plasma are stored in the NIPH Biobank.

Genotyping, QC, imputation, and post-imputation was performed in the current sub-sample of approximately 17,000 trios of which we here use ~ 30,000 unrelated parents (mothers and fathers). Genotype data was linked to the Norwegian Patient Registry comprising ICD-10 diagnoses for contact with specialist health service in the period 2008–2017. Cases have been defined in two different ways: 1: as depressive episode (F32) and/or recurrent depressive disorder (F33), 2: only recurrent depressive disorder (F33). Controls have been defined as individuals without any diagnoses from the F chapter in ICD-10. According to case definition number 1 we have 1,530 cases and 25,909 controls. According to case definition number 2 we have 929 cases and 25,909 controls.

Case Ascertainment:
Cases have been defined in two different ways: 1: as depressive episode (F32) and/or recurrent depressive disorder (F33), 2: only recurrent depressive disorder (F33) according to ICD-10. Diagnostic information has been acquired by linkage of genotype data with the Norwegian Patient Registry.

Control Ascertainment:
Controls have been defined as individuals without any diagnoses from the F chapter in ICD-10. Diagnostic information has been acquired by linkage of genotype data with the Norwegian Patient Registry.

Genotyping, QC, Imputation:
Approximately 17,000 trios from the Norwegian Mother, Father and Child cohort were genotyped in three batches. Genotypes were called using GenomeStudio (Illumina, San Diego, USA) and converted to PLINK format files. The first batch, comprising 20,664 individuals and 542,585 SNPs was genotyped at the NTNU Genomics Core Facility (Trondheim, Oslo) using the Illumina HumanCoreExome (Illumina, San Diego, USA) genotyping array, version 12 1.1. The second batch, comprising 12,874 individuals and 547,644 SNPs was genotyped at the NTNU Genomics Core Facility (Trondheim, Oslo) using the Illumina HumanCoreExome (Illumina, San Diego, USA) genotyping array, version24 1.0. The third batch, comprising 17,949 individuals and 692,367 SNPs, was genotyped at ERASMUS MC (the Netherlands) using the Illumina Global Screening Array (Illumina, San Diego, USA) version 24 1.

PLINK version 1.90 beta 3.36 (https://doi.org/10.1186/s13742-015-0047-8) was used to conduct the quality control, which has previously been described by Helgeland et al (2019;

https://doi.org/10.1038/s41467-019-12308-0). Known problematic SNPs previously reported by the Cohorts for Heart and Aging Research in Genomic Epidemiology (CHARGE) consortium and Psychiatric Genomics Consortium (PGC) were excluded from each batch. Duplicate samples were removed, and each genotyping batch was split into parents and offspring. Quality control was then conducted by genotyping array in parents and offspring separately.

Individuals were excluded if they had a genotyping call rate below 95% or autosomal heterozygosity greater than four standard deviations from the sample mean. SNPs were excluded if they were ambiguous (A / T and C / G), had a genotyping call rate below 98%, minor allele frequency of less than 1%, or Hardy-Weinberg equilibrium P-value less than 1×10^{-6}. Population stratification was assessed, using the HapMap phase 3 release 3 as a reference, by principal component analysis using EIGENSTRAT version 6.1.4. Visual inspection identified a homogenous population of European ethnicity and individuals of non-European ethnicity were removed. Individuals with a genotyping call rate below 98% or autosomal heterozygosity greater than four standard deviations from the sample mean were then removed. A sex check was done by assessing the sex declared in the pedigree with the genetic sex, which was imputed based on the heterozygosity of chromosome X. When sex discrepancies were identified, the individual was flagged. Relatedness was assessed by flagging one individual from each pairwise comparison of identity-by-descent with a pi-hat greater than 0.1.

The parents and offspring datasets were then merged into one dataset per genotyping batch; keeping only the SNPs that passed quality control in both datasets. All individuals passing the genotyping call rate and autosomal heterozygosity measures were included in the merged datasets. Therefore, the merged datasets included individuals previously excluded or flagged as a duplicate, ethnic outlier, having a sex discrepancy, or high level of relatedness. Concordance checks were then conducted on validated duplicates. Duplicate, tri-allelic and discordant (any discordance between the validated duplicates) SNPs were excluded. Individuals and SNPs with a genotyping call rate below 98% in the merged datasets were excluded. The duplicate sample that was removed before the start of the quality control was then excluded. Mendelian errors identified by the assessment of duos and trios were then recoded to missing. Insertions and deletions were also excluded.

After QC the Human Core Exome 12 batch comprised 20,231 individuals and 384,855 SNPs, the Human Core Exome 24 batch comprised 12,757 individuals and 396,189 SNPs, and the Global Screening Array batch comprised 17,742 individuals and 568,275 SNPs. Phasing was conducted using Shapeit 2 release 837 and the duoHMM approach was used to account for the pedigree structure. Imputation was conducted using the Haplotype reference consortium (HRC) release 1-1 as the genetic reference panel. The Sanger Imputation Server was used to perform the imputation with the Positional Burrows-Wheeler Transform (PBWT). The phasing and imputation were conducted separately for each genotyping batch.

Post imputation quality control was performed by initially converting the dosages to best-guess genotypes. Individuals were removed if they had a genotyping call rate less than 99% or were of non-European ethnicity. SNPs with an imputation INFO quality score less than 0.8, genotyping call rate less than 98%, minor allele frequency less than 1%, or a Hardy-Weinberg equilibrium P-value less than 1×10^{-6} were removed. Mendelian errors were set to missing. Relatedness, which was accounted for within generation and genotyping batch during pre-imputation QC as described above, was assessed intergenerationally and across batches by flagging one individual from each pairwise comparison of identity-by-descent with a pi-hat greater than 0.15 (excepting known parent-offspring relationships). Individuals were flagged for removal only if the other member of their pair would otherwise be included in the same analysis. One individual from each pair was flagged at random, except when retaining one individual in a pair would keep more duo/trio data intact than the other, in which case the other member was dropped. After quality control, a core homogeneous sample of European ethnicity (based on PCA of markers overlapping with available HapMap markers) individuals across all batches and arrays were available for use in analysis (totals prior to analysis-specific exclusions for relatedness: $N_{children}$ = 15,208; $N_{mothers}$ = 14,804; $N_{fathers}$ = 15,198).

Ethics statement:

The current study is based on version 11 of the quality-assured data files released for research in 2019. The establishment of MoBa and initial data collection was based on a license from the Norwegian Data Protection Agency and approval from The Regional Committees for Medical and Health Research Ethics. The MoBa cohort is currently regulated by the Norwegian Health Registry Act. The current study was approved by The Regional Committees for Medical and Health Research Ethics (2016/1226). In accordance with REK regulations, individuals who withdraw consent are excluded.

Funding:

The Norwegian Mother, Father and Child Cohort Study is supported by the Norwegian Ministry of Health and Care Services and the Ministry of Education and Research. We are grateful to all the participating families in Norway who take part in this on-going cohort study. We thank the Norwegian Institute of Public Health (NIPH) for generating high-quality genomic data. This research is part of the HARVEST collaboration, supported by the Research Council of Norway (#229624). We also thank the NORMENT Centre for providing genotype data, funded by the Research Council of Norway (#223273), South East Norway Health Authorities and Stiftelsen Kristian Gerhard Jebsen. We further thank the Center for Diabetes Research, the University of Bergen for providing genotype data and performing quality control and imputation of the data funded by the ERC AdG project SELECTionPREDISPOSED, Stiftelsen Kristian Gerhard Jebsen, Trond Mohn Foundation, the Research Council of Norway, the Novo Nordisk Foundation, the University of Bergen, and the Western Norway Health Authorities. This work was performed on the Tjeneste for Sensitive Data (TSD) facilities, owned by the University of Oslo, operated and developed by the TSD service group at the University of Oslo, IT-Department (USIT), using resources provided by Sigma2—the National Infrastructure for High Performance Computing and Data Storage in Norway (UNINETT).

Million Veteran Program (MVP) | Gelernter J; Levey D | PMID 34045744; 32243820 | analysis code: mdd_MVP.eur.hg19.4_0ICDdep_202106, MVP_DIV

Cohort Description:

The Million Veteran Program (MVP) is a national research program to learn how genes, lifestyle, and military exposures affect health and illness. Envisioned as a US Veterans Affairs (VA)-based mega-biobank, the MVP was launched to establish a national, representative, and longitudinal study of Veterans for genomic (and non-genomic) research that combines data from survey instruments, the electronic health record, and biospecimens. Since launching in 2011, over 825,000 Veteran partners have joined one of the world's largest programs on genetics and health. For the present study, MVP samples were used for GWAS discovery (EUR, diverse), and for PGS prediction (EUR, AFR).

Case Ascertainment:

VA medical records were used to identify cases. Participants were considered a case if they had one inpatient visits or one outpatient visit with an MDD ICD code. Exclusion criteria were a diagnosis of bipolar disorder or schizophrenia.

Control Ascertainment:

VA medical records were used to identify controls. Controls include only those without any inpatient or outpatient depression codes. Exclusion criteria were a diagnosis of bipolar disorder or schizophrenia.

Genotyping, QC, Imputation:

Array: Custom MVP 1.0 array based on Applied Biosystems Axiom Biobank Genotyping Array (https://www.sciencedirect.com/science/article/pii/S000292972030080X)

Genotyping, imputation, and quality control within MVP has been previously described. Briefly, samples were genotyped using a 723,305-SNP Affymetrix Axiom Biobank array customized for MVP. MVP genotype data for biallelic SNPs were imputed using Minimac4 and a reference panel from the African Genome Resources (AGR) panel by the Sanger Institute. Indels and complex variants were imputed independently using the 1000 Genomes (1KG) phase 3 panel and merged in an approach similar to that employed by the UK Biobank. Designation of ancestries was based on genetic assignment with comparison to 1KG reference panels. For post-imputation quality control, SNPs with an imputation INFO score <0.3 or a minor allele frequency (MAF)<0.001 were removed from analysis. Variants were excluded if call missingness in the best-guess genotype exceeded 20%. For the first tranche of data, 22,183 SNPs were selected through linkage disequilibrium (LD) pruning using PLINK 2.0, and then Eigensoft was used to conduct principal component analysis on 343,286 MVP samples and 2,504 1000 Genomes samples. The reference population groups (EUR, EAS, AFR, AMR, or SAS) in the 1000 Genomes samples were used to define European American (N=378,614) and African American (N=92,149) groups used in this analysis.

The GENESIS analysis (details see methods) for diverse/admixed ancestries was carried out in the subgroup of the participants defined from 1Kg reference panels as AFR, AMR, EAS, SAS, and ancestry outliers/OTHER.

Ethics statement:

The Central VA Institutional Review Board (IRB) and site-specific IRBs approved the MVP study. All relevant ethical regulations for work with human subjects were followed in the conduct of the study, and written informed consent was obtained from all participants.

Funding:

Study	Lead investigator	Award number	Funder	Country
MVP	Stein Gelernter	I01CX001849	United States Department of Veterans Affairs Office of Research and Development	USA
MVP	Daniel F Levey	1IK2BX005058-01A2	United States Department of Veterans Affairs Office of Research and Development	USA

Mass General Brigham Biobank (MGBB) | Smoller JW | PMID 26784234 | analysis code: mdd_PBK.eur.hg19.2020

Cohort Description:

Major depression (MD) cases and controls for GWAS were ascertained using EHR data from patients in the Mass General Brigham (MGB; formerly Partners Healthcare) Biobank[1] in the MGB health system.

Case Ascertainment:

EHR data before February 2020 were extracted for these participants including ICD-9/10 diagnostic codes. MD cases (n=16080) were defined based on having two or more MD-related diagnostic codes (excluding those with schizophrenia, psychosis, or autism-related codes).

Control Ascertainment:

EHR data before February 2020 were extracted for these participants including ICD-9/10 diagnostic codes. controls (n=5607) were defined based on having zero MD-related diagnostic codes (excluding those with anxiety, PTSD, bipolar, schizophrenia, psychosis, or autism-related codes).

Genotyping, QC, Imputation:

DNA from blood samples were genotyped from MGB Biobank participants on three Illumina arrays (MEGA, MEGAEX, and MEG BeadChip). During QC, SNPs were excluded based on call rate (<95%) and Hardy-Weinberg Equilibrium (p<1e-10), while individuals were excluded based on missing data (>2%), sex assignment errors, heterozygosity (±3 SD), and relatedness (randomly removing one individual from any pair with kinship > 0.2). Genotyping array batches were merged then imputed using the Michigan Imputation server (Minimac4 1.2.1, HRC/1KG reference panel). Imputed data were then converted to best-guess genotypes for all markers with high-quality imputation (R2 > 0.8) and common minor allele frequency (>1%), with participants of European ancestry retained for these analyses.

Ethics statement:

The Institutional Review Board at Mass General Brigham (formerly Partners HealthCare) approved this study.

Funding:

Study	Lead investigator	Award number	Funder	Country

PREFECT | Landen M | PMID 33483693 | analysis code: mdd_PREFECT.eur.hg19.run1

Cohort Description:
Subjects in PREFECT were enrolled from Swedish National Quality Register for electroconvulsive therapy (Q-ECT, http://ect.registercentrum.se) between 2013 and 2017. All Swedish hospitals administering ECT report to Q-ECT, which collects clinical and demographic data including the indication for the current ECT series.

Case Ascertainment:
For analyses of MDD in PREFECT, participants were included if the indication for ECT was a major depressive episode. The "narrow case definition" was a subset of patients in PREFECT consisting of those receiving ECT for a major depressive episode in the context of MDD (ICD-10 codes F320–F323, F328–F333, F338, F339, F412, F530).

Control Ascertainment:
Controls were obtained from the Swedish arm of the Anorexia Nervosa Genetics Initiative (ANGI). ANGI controls were population based (identified by Statistics Sweden, n = 1035) or archived controls from LifeGene (n = 3000), a population-based study of genes, environment, lifestyle, and health. Controls were excluded from the original ANGI study if they reported a history of an eating disorder. For purposes of the current study, we also excluded individuals with a self-reported lifetime history of MDD, bipolar disorder, schizophrenia, or schizoaffective disorder. As expected for an eating disorder cohort, ANGI controls were 98% female. Blood samples were provided at special test centers (LifeGene controls) or the nearest lab (population-based controls) and transported to Karolinska Institutet Biobank where DNA was extracted and stored.

Genotyping, QC, Imputation:
Array(s): Illumina GSA-MD SNP arrays (v1)

DNA was extracted from blood samples and genotyped on Illumina GSA-MD SNP arrays (v1). For QC, samples were excluded for genotype missingness > 0.02 (after first removing SNPs with call rate < 0.95), genotypic sex ambiguity or not matching phenotypic data, or autosomal heterozygosity |F| > 0.2. SNPs were excluded for call rates < 0.99, difference in missingness between cases and controls > 0.005, MAF < 0.01, or deviation from Hardy–Weinberg equilibrium in cases or controls ($P < 10^{-6}$). European ancestry outliers were removed. Samples passing QC were imputed to the HRC r1.1 reference panel using the Sanger Imputation Service using Eagle2 and PBWT for phasing and imputation.

Ethics statement:
All participants provided informed consent, and the study was approved by the ethical review board in Stockholm, Sweden (DNR: 2012/1969-31/1).

Funding:

Study	Lead investigator	Award number	Funder	Country
PREFECT	Mikael Landén	KF10-0039	The Swedish Foundation for Strategic Research	Sweden

Scottish Health Research Register (SHARE) | Palmer C; McIntosh AM | PMID 28148535 | analysis code: mdd_SHARE.eur.hg19.godartsshare_842021

Cohort Description:

The Scottish Health Research Register (SHARE) is an NHS research Scotland initiative currently comprised of a biobank of around 130,000 individuals. Participants provided a sample of blood for genetic analysis and informed consent to link their genetic information to the anonymized electronic NHS electronic health records including hospital discharge, prescribing, primary care, demography and general practitioners data. Participants of European ancestry were included in this study. Participants also registered in the Generation Scotland cohort were excluded from this study before analysis.

Case Ascertainment:

MDD cases were identified from electronic health records. MDD cases were identified using the electronic health records and ICD-10 codes. Inclusion criteria: MDD; Exclusion criteria: Participants with bipolar disorder, manic episode and schizophrenia were excluded from this study.

Control Ascertainment:

Controls were identified from electronic health records. Exclusion criteria for controls comprised any record of MDD, bipolar disorder, manic episode and schizophrenia diagnosis. Cases and controls were matched by age, gender and diabetes status.

Genotyping, QC, Imputation:

Array(s): Either Affymetrix 6.0 (A6) or Illumina 155 Omni Express (OMEX) chips or Illumina Global Screening Array version 2 plus (GSA2/GSA3).

Quality control (QC): Samples were excluded based on the following criteria: samples with a call rate less than 95%, the mismatch between clinical data and genotypic gender, batch effects, sample duplicates (IBD score > 0.8). SNPs were excluded on the basis of monomorphism, Hardy-Weinberg Equilibrium (HWE) p-value less than 1×10-6 and call rate less than 95%. PLINK version 1.09 was used to perform the quality assessment for genotyping data from all platforms. Ancestry outliers were identified by applying a first three standard deviation cut-off in a principal component analysis. The genotype data were imputed against a haplotype reference consortium (HRC version r1.1) reference panel in NCBI build 37. Post-imputation QC checks were applied; monomorphic markers or imputation quality score < 0.3 were excluded. The genomic position of the markers is based on the NCBI human genome build 37. GCTA version 1.94.1 tool was used to perform genome-wide association analysis. Genetic correlation and heritability estimation were performed using the LD score regression tool.

Ethics statement:

The study was approved by NHS Research Ethics Committees and NHS Tissue Bank. All participants gave informed written consent for the collection of DNA samples for use in genetic studies.

Funding:

**Study**	**Lead investigator**	**Award number**	**Funder**	**Country**
SHARE	Colin NA Palmer	099177/Z/12/Z	Wellcome Trust	UK
SHARE	Colin NA	NRS	Chief Scientist	UK

	Palmer	infrastructure funding	Office (Scottish Govt	
Investigator Award	Andrew McIntosh	220857/Z/20/Z	Wellcome	UK
Pathfinder	Andrew McIntosh	MC_PC_17209	MRC	UK
DATAMIND	Ann John	MR/W014386/1	MRC	UK

Swedish Twin Studies of Adults: Genes and Environment (STAGE) | Magnusson P; Lu Y | PMID 23137839; 31747977 | analysis code: mdd_STAGE.eur.hg19.MDDdx_saige

Cohort Description:
STAGE stands for Swedish Twin Studies of Adults: Genes and Environment which was a web-based data collection effort on Swedish twins born between 1959 and 1985 conducted during 2004 to 2006. The scope was broad with questionnaire instruments included for both somatic and mental health issues. Donations of DNA were requested in a separate effort.

Case Ascertainment:
This GWAS included 421 MDD cases diagnosed at inpatient care or outpatient specialist care (ICD-9-SWE: 296B, 311; ICD-10 F32, F33). The exclusion criteria were no lifetime specialist diagnosis for bipolar disorder, schizophrenia or schizoaffective disorder.

Control Ascertainment:
No record of ICD-9-SWE: 296B, 311; ICD-10 F32, F33 diagnoses. The exclusion criteria for controls were, same as for cases, no lifetime specialist diagnosis for bipolar disorder, schizophrenia or schizoaffective disorder.

Genotyping, QC, Imputation:
Genotyping:
Genotypes were generated at the SNP&SEQ Technology Platform at Uppsala University using the Illumina Infinium assay (chip GSAMD-24v1-0_20011747_A1) and GenomeStudio 2.0.3, and delivered in six batches during 2018–2019. Genotypes are encoded on the + strand of the GRCh37/hg19 build of the human reference genome.

Quality control:
Before standardized quality control, samples with less than 10% missing genotype data were examined for discrepancies in observed sex and relatedness using PLINK 1.90, PLINK 2.0, and R 3.6. A total of 86 samples were excluded for having sex chromosome abnormalities, genotypic sex different from register information, or showing unexpected relatedness patterns (*i.e.*, not related to their co-twin, but related to other samples, indicating sample mixup). At this stage, we also merged data from genotyped MZ twin pairs (zygosity confirmed by genotype data) with genotype missingness < 2% in both twins into one sample per pair (N=54). 8,356 genotyped samples were processed using the Ricopili pipeline for quality control. As the pipeline is optimized for QC of case-control studies, zygosity was used as a temporary phenotype. “Cases” and “controls” below thus refers to DZ and MZ twins respectively. After first removing SNPs with missingness>0.05 (N=12,831), 13 samples failed sample QC due to any of the following: per-sample call rate <0.98; excessive heterozygosity (FHET outside +/- 0.2); sex mismatch. 207,658 out of 700,078 markers failed SNP QC due to any of the following: per-SNP call rate < 0.98; minor allele frequency < 1%; Hardy-Weinberg disequilibrium (P < 1e-6 in controls and P < 1e-10 in cases); difference in call rate between cases and controls > 0.02. Finally, unplaced markers were removed, multiallelic sites encoded as multiple markers with the same position but different alleles had the least common variant removed, and markers passing QC and having the same position and alleles were merged into one marker per position, in total removing 581 SNPs, and leaving 491,839 directly genotyped SNPs for analysis. By projecting the first two principal components (PCs) of the study samples to the 1000 Genomes global population reference panel using PLINK 1.9, we identified 28 samples as non-European ancestral outliers (more than 6 standard deviations from the means of European reference samples in PC1 or PC2). Ancestral outliers were excluded from any case-control comparisons in QC, but remained in the clean genotype files if they passed all other filters.

Imputation:

Post-QC genotype files were submitted to the Sanger imputation service for imputation to the Hap- lotype Reference Consortium panel (HRC1.1). EAGLE2 and PBWT were used for phasing and imputing respectively. 40,359,612 SNPs were available after the HRC imputation.

PCA:
A principal component analysis (PCA) was performed in the study sample without an external population reference to generate ancestry covariates for association analyses. The first 20 principal components based on common directly genotyped markers in linkage equilibrium (MAF>=0.05, pairwise R^2<=0.1) were extracted using PLINK 1.9 and 2.0. Known regions of long-range linkage disequilibrium were excluded from the analysis. PCA weights were derived in unrelated individuals only, and then projected on the full sample of twins. The PCA analysis was repeated with non-European ancestral outliers removed.

Relatedness:
Relatedness within and between family IDs (twin pairs) was estimated using the KING algorithm implemented in PLINK 2.0. The estimated kinship parameter has an expected value of 0.25 for DZ twins, and 0.5 for MZ twins. Note that only one individual from each MZ pair is included in the clean genotype files by design (see the next section for datasets including MZ co-twins). Individuals showing relatedness between family IDs were not excluded.
MZ imputation
After QC and imputation, MZ co-twins were imputed from their genotyped sibling, and added to the main genotype dataset. The twins who were imputed in this manner either had their zygosity confirmed by GWAS array data (and were merged in QC preparations) or were imputed based on register information. The latter set of individuals are flagged with IMPUTE_MZ==1 in STR. MZ imputation was performed using bcftools for imputed data and PLINK 1.9 for directly genotyped data. The total sample size including imputed MZ co-twins is 9,701. After imputation, PLINK 2.0 pgen file sets were generated along with the full VCF output for more convenient downstream processing. These files have a light post-imputation quality filter applied compared to the unfiltered VCF data (MAF>=0.005, INFO>0.1), leaving 9,141,508 markers for analysis.

Analysis:
For the GWAS, we have modeled the twin relatedness and corrected for case-control imbalance using SAIGE, adjusting for top 4 PCs.

Ethics statement:
The study was approved by the Stockholm local ethics committee (DNR 03-224; 2018/960-31/2; 2020-00535) and all participants gave informed consent.

Funding:

Study	Lead investigator	Award number	Funder	Country
Swedish Twin Registry (STAGE is a substudy)	P Magnusson	2017-00641	Swedish Research Council	Sweden

Acknowledgement:
The computations were enabled by resources provided by the Swedish National Infrastructure for Computing (SNIC) at the UPPMAX server partially funded by the Swedish Research Council through grant agreement no. 2018-05973.

Takeda vortioxetine cohort (Takeda) | Wendland JR; Badola S | PMID 25687662; 25575488; 26035185; 26035186 | analysis code: mdd_tkda1.eur.hg19.run1

Cohort Description:
Takeda vortioxetine cohort. Subjects with Major Depressive Disorder were recruited as part of the following clinical trials (ClinicalTrials.gov identifier): NCT01564862 (PMID 25687662); NCT01153009 (PMID 25575488), NCT01163266 (PMID 26035185), and NCT01179516 (PMID 26035186).

Case Ascertainment:
Subjects were required to have a diagnosis of recurrent MDD as per DSM-IV or DSM-IV TR. Key exclusion criteria were the presence of any psychiatric disorder other than MDD, alcohol or substance abuse, or presence of a neurological disorder. Details for each study have been published in peer-reviewed journals and on ClinicalTrials.gov.

Control Ascertainment:
No controls included.

Genotyping, QC, Imputation:
Cases were genotyped on the Illumina Omni 5 plus exome array. Controls were genotyped at the Broad Institute using the Illumina Omni Express and Omni 2.5 array, which had 660k overlapping SNPs with the Illumina Omni 5 plus exome array. This merged dataset provided enough SNPs after QC and imputation for inclusion into the MDD meta-analysis. Genotype QC, PCA and imputation was performed using Ricopili software using default parameters for sample and SNP inclusion, and 1KG reference populations for ancestry assignment. The merged sample was split into three separate continental ancestry subsets: European (672 cases / 878 controls), African (265 cases / 928 controls), and American (129 cases / 408 controls), with QC and PCA being recalculated within each subset. Phasing (EAGLE 2.3.5) and Imputation (Minimac3) was run in Ricopili using the Haplotype Reference Consortium (HRC) release 1-1 reference panel. Finally, association analyses were run on imputed dosages using a logistic regression model including all PCs with case/control association $p < 0.1$ included in the model.

Ethics statement:
All subjects in the Takeda vortioxetine cohort provided written informed consent for exploratory DNA analyses. For detailed information, see the individual clinical trials. Studies were conducted under ICH GCP and in accordance with the Declaration of Helsinki. Study protocols and all related forms and amendments were approved by the independent ethics committee of each study center.

Funding:
Studies and genotyping were funded by Takeda Development Center Americas, Deerfield, Illinois, United States of America. For more details, please see the listed publications and trial information on ClinicalTrials.gov.

UK Biobank (UKB) - EUR | McIntosh A; Adams M | PMID 30305743 | analysis code: mdd_UKBB.eur.hg19.MD_glm_202012, UKBB_DIV

Cohort Description:
UK Biobank (UKB) is a population-based study of health in middle-aged and older individuals (*N*=502,616). Eligible participants were aged 40–69 and recruited from 22 assessment centers in the United Kingdom. The present study was conducted under UK Biobank application 4844. Case and control status for major depression were drawn from a combination of sources. Participants were administered touchscreen and online questionnaires about cardinal symptoms of depression, their frequency, and duration, verbal interview for self-reported illnesses, and an online version of the Composite International Diagnostic Interview Short Form. Participants were linked to hospital inpatient and general practitioner diagnoses and prescribing data.

Case Ascertainment:
European Ancestry: Participants were defined as cases if they matched two or more of the following criteria: self report or questionnaire (CIDI) case definition, antidepressants self-declared treatment, one to two GP diagnoses of MDD, three or more episodes of GP antidepressant prescribing. Participants were also defined as cases if they matched one of the following criteria: three or more GP diagnoses of MDD or inpatient diagnoses of MDD. Cases were excluded if they self-reported schizophrenia psychosis, mania, bipolar disorder, personality disorder or had health record diagnoses for schizophrenia, schizotypal, delusional, and other non-mood psychotic disorders or personality disorders.

Diverse ancestry: We used a combination of hospital diagnoses (ICD10 codes) and lifetime CIDI (A. prolonged feelings of depression OR prolonged loss of interest in normal activities AND B. affected more than half of the day during worst episode of depression AND C. the frequency of depressed days during worst episode was at almost every day/every day AND D. these problems interfered with your life/activities (study/employment, childcare and housework, leisure pursuits) somewhat/a lot) to define our cases.

Control Ascertainment:
European Ancestry: Controls were defined as being negative for depression in self-report questionnaires, did not self-declare antidepressant treatments or had two or fewer GP antidepressant prescriptions, and had no GP or hospital inpatient diagnosis of a mood, psychotic, or personality disorder.

Diverse ancestry:
Controls were defined as being negative for depression in self-report questionnaires, had no GP or hospital inpatient diagnosis of a mood, psychotic, or personality disorder.

Genotyping, QC, Imputation:
Array: AXIOM UK BiLEVE Axiom, UK Biobank Axiom. UK Biobank contains genotype data imputed to ~92 million variants.
European Ancestry: We performed QC procedures on SNPs with filters for MAF>0.01 and INFO>0.9. We removed participants who had failed genotype platform QC, who did not cluster genetically as White British, or who overlapped with Psychiatric Genomics Consortium MDD and Generation Scotland participants; and we conducted additional filtering on related individuals. This resulted in 9,096,571 variants for 361,130 individuals for genetic analysis. We calculated principal components for this sample using FlashPCA2. We conducted a genome-wide association study in PLINK2 (v2.00a2.3LM) using a GLM with 'genotype array' and 20 PCs as covariates.

Diverse ancestry: We first performed principal component analysis using the PC-Air method (Reference: PMID 25810074), that accounts for relatedness and captures the population structure. Based on approximation to reference population of European ancestry, we identified 38,598 subjects of diverse ancestry from the UK Biobank cohort. These subjects were included in our GWAS of diverse ancestry. Gender mismatches, missingness/heterozygosity outliers, participants with excessive genetic relatedness, no quality control metrics, individuals that have withdrawn their consent were excluded before the analysis. According to the criteria, we identified 3328 cases and 5638 controls.

Ethics statement:
UK Biobank received ethical approval from the Research Ethics Committee (reference 11/NW/0382).

Funding:

Study	Lead investigator	Award number	Funder	Country
UKBB	McIntosh A	104036/Z/14/Z<br>220857/Z/20/Z	Wellcome Trust	UK

Detroit Neighborhood Health Study (DNHS) | Uddin M | PMID 20439746; 22144187 | analysis code: DNHS

Cohort Description:
The DNHS is a longitudinal epidemiologic study investigating correlates of PTSD and other mental disorders in the city of Detroit. It recruited adults (18 years or above) from the Detroit population. Initially, 1,547 households were randomly drawn from the city of Detroit under a probability sample. Then an interview was conducted with one random individual from each household. Participants underwent a 40-minute assessment consisting of questions on socio-demographic characteristics, major depressive disorder (MDD), post-traumatic stress disorder (PTSD) and generalized anxiety disorder (GAD) (PMID 20439746).

Case Ascertainment:
MDD was scored using the Patient Health Questionnaire (PHQ-9) and DSM-IV criteria.

Control Ascertainment:
Controls screened negative for MDD.

Genotyping, QC, Imputation:
DNA for GWAS analysis was isolated from peripheral blood or saliva. Study participants were genotyped with Illumina HumanOmniExpress array and imputation was conducted based on the 1000 Genomes phase 3 data. Relatedness was estimated using the IBS function in PLINK 1.9. From each pair with relatedness $\pi^{\wedge} > 0.2$, one individual was removed from further analysis, retaining cases where possible. Principal components were calculated based on the smartPCA algorithm in EIGENSTRAT (PMID 31594949).
A total of 58 cases, which were defined at the baseline visit, and 436 controls of African ancestry were included in our MD GWAS for the DNHS cohort. Logistic regressions were implemented by PLINK2 with imputed dosage data, adjusting for sex, age at baseline assessment, and the first 20 PCs.

Ethics statement:

The Institutional Review Board at the University of Michigan and the University of North Carolina Chapel Hill reviewed and approved the study protocol (https://www.ncbi.nlm.nih.gov/projects/gap/cgi-bin/study.cgi?study_id=phs000560.v1.p1).

Funding:
R01MD011728

Drakenstein Child Health Study (DCHS) | Stein DJ | PMID 25797842 | analysis code: SAFR

Cohort Description:
The DCHS is a population-based birth cohort study in the Drakenstein area in Paarl, a peri-urban area 60 km outside Cape Town, South Africa. Data collection occurred at two clinics (maternal data) as well as at a central hospital (newborn outcomes) in the Drakenstein area between March 2012 to March 2015. Participants were enrolled in the DCHS at 20 to 28 weeks' gestation upon presenting for antenatal booking and followed longitudinally throughout pregnancy until at least five years postnatally. Maternal, paternal and child health are investigated through longitudinal measurements of risk factors in seven areas (environmental, infectious, nutritional, genetic, psychosocial, maternal and immunological) that may impact on child health (PMID 25797842; 25228292). More detailed information on the DCHS can be found on the study website (https://health.uct.ac.za/department-paediatrics/research-about-research-department-research-units-mrc-unit-child-and-adolescent-health/drakenstein-child-health-study-dchs). Exclusion criteria for the DCHS were minimal in order to maximise generalisability, and focused primarily on those individuals who did not live in the region (and thus could not be readily followed up) or those who were intending to move out of the district within the first year.

Case Ascertainment:
Depression cases were defined by BDI-II score (no less than 20 points).
Exclusion criteria for both cases and controls consisted of women who had stillbirths, infant deaths, gave birth to twins/triplets, were diagnosed with lifetime bipolar disorder or psychosis.

Control Ascertainment:
Controls were defined with both BDI-II score and EPDS score (BDI-II score of less than 20 points and EPDS score of less than 13 points).
Exclusion criteria for cases and controls consisted of women who had stillbirths, infant deaths, gave birth to twins/triplets, were diagnosed with lifetime bipolar disorder or psychosis.

Genotyping, QC, Imputation:
DNA was extracted from whole blood using the QIAsymphony DSP DNA Midi kit and protocol (Qiagen, Hilden, Germany). Genome-wide SNP genotyping was conducted using either the Infinium PsychArray or Global Screening Array-24 BeadChip (Illumina). Standard quality control of the genome-wide data was performed using PLINK removing individuals with >5% missing data and removing one in each pair of related individuals with an IBD proportion >0.12 (indicating cousins or a closer relation). The DCHS researchers removed SNPs with call rates <95%, MAF <0.05 and deviation from Hardy–Weinberg proportions ($P < 1 \times 10^{-6}$ in controls and $P < 1 \times 10^{-10}$ in cases). To evaluate population stratification, PC eigenvectors of the genetic relationship matrix were calculated by using about 50 000 independent SNPs. SNPs in LD (r2=0.075) were excluded to calculate PCs. Dimensional plots of the PCs were also used to remove outliers. The Faculty of Health Sciences human research ethics committee of the University of Cape Town (UCT) approved this study.

We acquired individual-level genotype imputed data from the DCHS researchers. Logistic regressions were run by PLINK2 adjusting for the first 20 PCs, recruitment site, age of enrolment for controls or age of assessment for cases.

Ethics statement:
The study was approved by the faculty of Health Sciences, Human Research Ethics Committee, University of Cape Town (401/2009), Stellenbosch University (N12/02/0002) and the Western Cape Provincial Health Research committee (2011RP45) (PMID 29942867).

Funding:

Not available.

Jackson Heart Study (JHS) | - | PMID 14632260 | analysis code: JHS

Cohort Description:
Data of the Jackson Heart Study (JHS) was accessed via dbGaP under project ID 18933. The participants with available depression phenotypes from a sub-study of JHS with genotyped data were analysed for the current study (dbGaP study accession: phs001356.v1.p2).

Study designs for the JHS study have been described elsewhere (PMID 14632260; 14632263). In short, the JHS is a large, community-based, observational study, which recruited 5,301 participants from among the non-institutionalized African-American adults from urban and rural areas of the three counties (Hinds, Madison, and Rankin) that make up the Jackson, Mississippi, metropolitan statistical area (MSA). Participants provided extensive medical and social history and had an array of physical and biochemical measurements and diagnostic procedures during a baseline examination (2000–2004) and two follow-up examinations (2005–2008 and 2009–2012).

Case Ascertainment:
For current depression, cases were identified by a 20-item Center for Epidemiologic Studies Depression Scale (CES-D) score of 16 or greater.

Control Ascertainment:
Controls were identified by a 20-item CES-D score of less than 16.

Genotyping, QC, Imputation:
Study participants were genotyped by the Illumina Human Exome BeadChip v1.1 array and then imputed to the 1000 Genomes Phase 3 African reference panel. Samples with genotyping call rate of less than 95% and variants which were successfully genotyped in less than 95% of samples were excluded. Relatedness coefficients were calculated by KING. Related individuals up to 2nd degree relatedness were randomly excluded (kinship > 0.0884). A total of 299 depression cases and 990 controls were included in our GWA for the JHS study. Logistic regressions adjusting for age, gender, recruitment type and first 20 PCs were implemented in PLINK2. Following the analyses, variants with imputation R squared of less than 0.7 or a minor allele count of less than 50 were excluded.

Ethics statement:
The study was approved by the Institutional Review Board of the National Institutes of Health and the study protocol was approved by the Institutional Review Boards of the participating JHS institutions, including the University of Mississippi Medical Center, Jackson State University and Tougaloo College (PMID 26699120).

Funding:
The JHS is supported by contracts from the National Heart, Lung and Blood Institute and the National Institute on Minority Health and Health Disparities and is conducted in collaboration with Jackson State University (HHSN268201800013), Tougaloo College (HHSN268201800014), the Mississippi State Department of Health (HHSN268201800015), and the University of Mississippi Medical Center (HHSN268201800010, HHSN268201800011 and HHSN268201800012).

Prevention Intervention Research Center (PIRC) 1st Generation Trial (PIRC) | Rabinowitz JA | PMID 24948529 | analysis code: PIRC

Cohort Description:

This study was a trial designed and conducted by the Prevention Intervention Research Center at Johns Hopkins University. A total of 2,311 youth entering first grade in 1985 or 1986 in 19 primary public schools, which were selected from five areas to represent the socio-demographic diversity in the northeastern quadrant of Baltimore in 1985, were recruited (PMID 24948529). The trial was initially aimed at assessing the immediate effects of two universal, first-grade preventive interventions (i.e., classroom-centered intervention vs family-school partnership intervention) on the proximal targets of poor achievements, concentration problems, aggression and shy behaviours, which were known as early risk behaviours for later substance use/abuse, affective disorder and conduct disorder. Twenty five years later, 65% of the surviving cohort (n=1,434) participated in a follow-up interview that inquired about their general and mental health, including alcohol, tobacco, and other drug involvement.

Participants were asked about their willingness to donate a blood sample. If unwilling and/or unable to donate blood, they were then asked if they would donate a saliva sample instead.

Case Ascertainment:

Lifetime history of major depressive episode was measured using the Diagnostic Interview Schedule-III-R (DIS-III-R).

Control Ascertainment:

All living study participants at 25-year follow-up who did not fulfill the criteria for a lifetime history of a depressive episode according to the case definition were included as control participants.

Genotyping, QC, Imputation:

DNA was extracted from blood or saliva samples, then quantitated and genotyped using Affymetrix 6.0 microarray (Santa Clara, CA, USA) (PMID 23128154). Genotypes were imputed to the TopMed using the Michigan Imputation Server (PMID 27571263). The resulting variants imputed with an INFO score of less than 0.8 were removed. Genome-wide logistic regressions were conducted by R (version 3.6.1) for 52 cases and 547 controls of African ancestry, adjusting for participant age, sex, intervention status (control vs exposure to intervention) and the first 20 PCs.

Ethics statement:

The study protocol was approved by the institutional review board for protection of human subjects at Johns Hopkins University (PMID 24948529). All procedures followed were in accordance with the ethical standards of the responsible committee on human experimentation (institutional and national) and with the Helsinki Declaration of 1975, as revised in 2000. Informed consent was obtained from all participants included in the study.

Funding:

Work on the PIRC was supported by several National Institute of Health grants over the span of the prospective study: National Institute of Mental Health MH71395 and National Institute on Drug Abuse DA09897, DA04392, and DA019805.

Women's Health Initiative (WHI) | Wassertheil-Smoller S | PMID 9492970; 14769624; 24572626 | analysis code: WHI_share_AFR, WHI_share_HIS, WHI_DIV

Cohort Description:

The WHI study is a long-term national health study in U.S conducted in postmenopausal women, enrolled either in a clinical trial or an observational study (PMID 9492970). We analysed data from 3,492 women with Asian ancestry who were genotyped as part of the WHI

– Population Architecture using Genomics and Epidemiology (PAGE) sub-study. These participants had agreed their data to be included in the database of Genotypes and Phenotypes (dbGaP). The genotype and phenotype data were assessed vid dbGaP study accession phs000200.v12.p3. Depressive symptoms in the past week were assessed in the baseline visit with a 6-item Center for Epidemiological Studies Depression Scale (CES-D) form.

Case Ascertainment:
Based on Smoller et al., definitions (PMID 14769624), participants with a score of 5 or more (6-item CES-D) were considered as depression cases.

Control Ascertainment:
Participants not classified as currently depressed (6-item CES-D), without medical history of depression (2-item Diagnostic Interview Schedule) and not on antidepressant therapy constituted the control group.

Genotyping, QC, Imputation:
The dataset of WHI included in our analyses, has been genotyped with CardioMetaboChip, as part of the NHGRI's PAGE project. Samples and variants with a call rate lower than 95%, typed variants with different missingness rates between case and control group > 0.2 and variants with MAF < 0.05 were excluded from downstream analysis. A logistic regression analysis was performed (PLINK2), adjusting for age, sex, 20 PCs and study subgroup.

Ethics statement:
The study was approved by the ethics committees at the Women's Health Initiative Coordinating Center, Fred Hutchinson Cancer Research Center, and at all 40 clinical centres (PMID 24572626).

Funding:

The WHI program is funded by the National Heart, Lung, and Blood Institute, National Institutes of Health, U.S. Department of Health and Human Services through contracts N01WH22110, 24152, 32100-2, 32105-6, 32108-9, 32111-13, 32115, 32118-32119, 32122, 42107-26, 42129-32, and 44221

BioMe (BioMe) | Loos R | PMID 23588317 | analysis code: BioMe

Cohort Description:
Bio*Me* is an electronic medical record-linked biobank of more than 50,00 participants from the Mount Sinai Health System (PMID 28895531).

Case Ascertainment:
Bio*Me* cases were individuals with a medical depression diagnosis (ICD-9 296.2, 296.3, 296.82, 298.0, 300.4, 301.12, 311; ICD-10 F32, F33, F34.1, F38.1). Participants diagnosed with dementia, bipolar or manic disorder, developmental disorders, intellectual disability, psychotic disorder, personality disorder were excluded from this study.

Control Ascertainment:
Controls were defined as individuals without a medical depression diagnosis. Participants diagnosed with dementia, bipolar or manic disorder, developmental disorders, intellectual disability, psychotic disorder, personality disorder were excluded from this study.

Genotyping, QC, Imputation:
Bio*Me* samples were genotyped with the Infinium Global Screening Array (GSA) BeadChip. Individuals with population-specific heterozygosity rate that surpassed +/- 6 standard deviations of the population-specific mean, along with individuals with a call rate of <95%, individuals with discordant reported and genetic sex and with phenotypically intermediate sex were not considered in the analysis. In cases of duplicates, the sample of each pair with the lower missingness rate in the exomic data was preferentially excluded. Genetic variants exclusions included a call rate <95% and HWE $p < 10^{-5}$.

The resulting dataset was imputed to the 1000Genomes Phase 3 reference panel. The GWAS was performed with a binary mixed model (SAIGE). The first 20 PCs were calculated using PLINK (v1.9) and a genomic relationship matrix (GRM) was calculated using the KING (v1.4) software (-ibs). The PCA and GRM calculations were restricted to common (MAF>0.01), autosomal sites. Additionally, variants with MAF<0.05 and info<0.07 were excluded before the meta-analysis.

Ethics statement:
Study participants were recruited from the Bio*Me* Biobank Program of The Charles Bronfman Institute for Personalized Medicine at Mount Sinai Medical Center from 2007 onward. The Bio*Me* Biobank Program (Institutional Review Board 07-0529) operates under a Mount Sinai Institutional Review Board-approved research protocol. All study participants provided written informed consent.

Funding:
Not available.

Healthy Life in an Urban Setting (HELIUS) | Verweij KJH; Zwinderman K | PMID 23621920, 29247091 | analysis code: HELIUS

Cohort Description:
Healthy Life in an Urban Setting (HELIUS) is a prospective cohort study executed in Amsterdam, characterized by ethnic diversity. HELIUS includes six large groups of inhabitants of Amsterdam, namely, Dutch, African Surinamese, South-Asian Surinamese, Turkish, Moroccan, or Ghanaian background, and one small group with a Javanese Surinamese background. The HELIUS cohort consists of approximately 25,000 participants aged 18–70 years. For most participants, data on social, environmental, and biological determinants were collected, and follow-up data were obtained. Detailed information on the cohort participants and gathered data has previously been published (PMID 23621920; 29247091).

Case Ascertainment:
Participants were defined as cases if they answered YES to both of the following questionnaire items (self-report): "Have you ever had a period of two weeks or longer in your life when you felt sad, empty, down or depressed?" and "Have you ever had a period of two weeks or longer in your life when you had little or no interest or pleasure in things that you typically enjoy?"

Control Ascertainment:
Participants were defined as controls if they answered NO to both of the following questionnaire items (self-report): "Have you ever had a period of two weeks or longer in your life when you felt sad, empty, down or depressed?" and "Have you ever had a period of two weeks or longer in your life when you had little or no interest or pleasure in things that you typically enjoy?"

Genotyping, QC, Imputation:
A cross-selection of 10,285 HELIUS participants was made for genotyping. Whole blood for DNA isolation was collected in EDTA tubes and stored at −80°C in the AMC Biobank. DNA was isolated using the Gentra Puregene Isolation Kit (Qiagen), and quality control procedures were performed to determine the DNA yield and purity.

Genotyping was performed at the Erasmus MC Human Genomic Facility, using the Illumina Global Screening Array 24v1-0 designed for the multiethnic genome-wide content purpose was used. An in-house protocol of the Human Genomic Facility, with Illumina's GenomeStudio software, was used to perform the initial genotyping of the array. Subsequently, a second quality control (QC) was performed for removing the individuals with discordant gender information and when more than 5% called data on markers per individual were missing.

Before imputation a general QC for the autosomal markers was executed removing variations with more than 5% calls missing, minor allele frequencies (MAF) of <1%, violation of the Hardy–Weinberg equilibrium (HWE) ($p \leq 10-5$), and heterozygosity deviations from a mean larger than ± 3 SD.

Imputation was conducted with the Sanger imputation server using the 1000 Genome phase 3 cohort. All markers are reported with respect to the reference allele and coordinates of GRCh37.

Ethics statement:
The HELIUS study has been approved by the Ethical Review Board of the Academic Medical Center Amsterdam. All participants approved by giving written informed consent.

Funding:
The Academic Medical Center (AMC) of Amsterdam and the Public Health Service of Amsterdam (GGD Amsterdam) provided core financial support for HELIUS. The HELIUS study is also funded by research grants of the Dutch Heart Foundation (Hartstichting; grant no. 2010T084), the Netherlands Organization for Health Research and Development (ZonMw;

grant no. 200500003), the European Integration Fund (EIF; grant no. 2013EIF013) and the European Union (Seventh Framework Programme, FP-7; grant no. 278901).

Multi-Ethnic Study of Atherosclerosis (MESA) | - | PMID 12397006 | analysis code: MESA

Cohort Description:
Data of the Multi-Ethnic Study of Atherosclerosis (MESA) was accessed via dbGaP under project ID 18933. The participants with available depression phenotypes and genotype data were analysed for the current study (dbGaP study accession: phs000209.v12.p3).

A detailed description of the study is available elsewhere (PMID 12397006). Briefly, MESA is a study of the characteristics of subclinical cardiovascular disease and the risk factors that predict progression to of the disease. The participants comprise a diverse, population-based sample of 6,814 asymptomatic men and women aged 45–84 recruited from six field centers across the United States: Wake Forest University, Columbia University, Johns Hopkins University, University of Minnesota, Northwestern University and University of California - Los Angeles. Thirty-eight percent of the recruited participants are white, 28% African-American, 22% Hispanic, and 12% Asian, predominantly of Chinese descent.

Each participant received an extensive physical exam and was asked to answer questions about standard coronary risk factors, sociodemographic factors, lifestyle factors, and psychosocial factors. Blood samples were assayed for biochemical risk factors and DNA was extracted and lymphocytes cryopreserved for the study of candidate genes and for genome-wide scanning, expression, and other genetic techniques. The baseline examination occurred July 2000–July 2002 and was followed by four examination periods that were 17–20 months in length. Participants have been contacted every 9–12 months throughout the study to assess clinical morbidity and mortality.

Case Ascertainment:
Current depression was ascertained with the 20-item CES-D questionnaire (score ≥ 16).

Control Ascertainment:
Participants that never fulfilled the criteria for current depression were ascertained as controls.

Genotyping, QC, Imputation:
Array: Affymetrix, MEGA
The TopMed Imputation Server was utilized for genotype imputation (PMID 33568819; 27571263; 25338720). Imputed SNPs were excluded if the estimated imputation accuracy was low (INFO score < 0.7). Rare variants were excluded (MAF < 0.01 or Neff>50 with Neff=2*MAF*(1 - MAF)*N*INFO). SNPs and samples with a call rate < 0.95 were excluded. HWE was not checked for this diverse ancestry sample.

All study subjects from the MESA study were analyzed using a joint mixed model as implemented in the GENESIS R package, regardless of their ethnic background.

Ethics statement:
Informed consent was obtained from participants upon their arrival at the study clinic. The institutional review boards of the six field centers approved the study protocol.

Funding:
The MESA study is supported by contracts N01-HC-95159 through N01-HC-95169 with the National Heart, Lung, and Blood Institute.

Army-STARRS (Army-STARRS) | Ursano RJ; Stein MB | PMID 25338841 | analysis code: Army-STARRS

Cohort Description:
Data from the Army-STARRS, a study conducted in army members in USA, were also assessed in the current analysis. Army STARRS includes the New Soldier Study (NSS) and the Pre/Pst Deployment Study (PPDS). Detailed information about the design of the study have been published previously (PMID 24865195). Depression outcomes were measured with the CIDI screening scales and evaluated for concordance with DSM-IV diagnoses within the Army STARRS clinical reappraisal study (PMID 24318217).

Case Ascertainment:
Cases were individuals with probable lifetime Major Depressive Disorder on the basis of the CIDI screening scales, as noted above

Control Ascertainment:
Controls were individuals without probable lifetime Major Depressive Disorder on the basis of the CIDI screening scales, as noted above.

Genotyping, QC, Imputation:
Samples were genotyped using either the Illumina OmniExpress + Exome array with additional custom content or the Ilumina PsychChip. Quality control (QC) of genotype data used standard protocols as described elsewhere [Stein et al. 2016]. Relatedness testing was carried out with PLINK v1.90 and, for pairs of subjects with π of >0.2, one member of each relative pair was removed at random.

Genotype imputation was performed with a 2-step pre-phasing/imputation approach with a reference multi-ethnic panel from 1000 Genomes Project (August 2012 phase 1 integrated release; 2,186 phased haplotypes with 40,318,245 variants). We removed SNPs that were not present in the 1000 Genomes Project reference panel, had non-matching alleles to 1000 Genome Project reference, or with ambiguous, unresolvable alleles (AT/GC SNPs with minor allele frequency [MAF] > 0.1). A total of 664,457 SNPs for the Illumina OmniExpress array and 360,704 for the Illumina PsychChip entered the imputation.

We performed the following quality control procedures to obtain the genotype data for population assignment and principal components analysis (PCA). We retained autosomal SNPs with missing rate < 0.05; samples with individual-wise missing rate < 0.02; SNPs with missing rate < 0.02; and SNPs with missing rate difference between cases and controls < 0.02. After QC, we merged our study samples with HapMap3 samples. We retained SNPs with MAF $\geq$ 0.01 and performed LD pruning at R^2 > 0.02. Finally, we excluded SNPs in MHC region (Chr 6:25–35Mb) and Chr 8 inversion (Chr 8:7–13 Mb).

Ethics statement:
Recruitment, consent, and data protection procedures were approved by the Human Subjects Committees of the Uniformed Services University of the Health Sciences for the Henry M. Jackson Foundation (the primary grantee), the Institute for Social Research at the University of Michigan (the organization implementing all Army STARRS surveys), and all other collaborating organizations. These procedures were approved by the Human Subjects Committee of all collaborating organizations.

Funding:
Army STARRS was sponsored by the Department of the Army and funded under cooperative agreement number U01MH087981 (2009–2015) with the U.S. Department of Health and Human Services, National Institutes of Health, National Institute of Mental Health (NIH/NIMH) MPIs: Ursano J; Stein MB.

Brazilian High Risk (BHRCS) | Salum G; Pan P; Ota VK; Santoro ML; Oliveira AM; Belangero SI | PMID 25469819 | analysis code: BHRCS

Cohort Description:

The Brazilian High Risk Study for Mental Conditions (BHRCS), conducted at the Universidade Federal do Rio Grande do Sul, used a two-stage design. We first assessed childhood symptoms and family history of psychiatric disorders in a screening interview, collecting information from 9,937 index children at 57 schools in the cities of São Paulo and Porto Alegre, as well as from 45,394 family members. In the second stage, a random subsample (intended to be representative of the community, n = 957) and a high-risk subsample (children at increased risk for mental disorders, based on family risk and childhood symptoms, n = 1554) were selected for further evaluation.

We evaluated those 2,512 subjects using an extensive protocol, involving one 2-h home evaluation with the parents, two 1-h evaluations of the child by a psychologist, and two 1-hour evaluations of the child by a speech pathologist. In those evaluations, saliva samples for genetic studies were collected from the subjects and their biological parents. In addition, 750 children were invited to take part in a neuroimaging study and to provide blood samples for the assessment of peripheral blood biomarkers. Those children have collectively designated the enriched imaging cohort. We have a 3-year follow up with 80% retention and a 6-year follow-up with 75% retention.

Case Ascertainment:

Community-based.

Children: Diagnosis of MDD in any of the three waves (baseline, 3-year or 6-year follow-up) assessed by Development and Well-Being Behavior Assessment (DAWBA) (n=364).

Adults: Diagnosis of MDD assessed by Mini International Neuropsychiatric Interview (MINI) at baseline (n=320).

Control Ascertainment:

Community-based.

Children: No diagnosis of any psychiatric disorder in any of the three waves assessed by Development and Well-Being Behavior Assessment (DAWBA) nor family history of MDD (n=732).

Adults: No diagnosis of any psychiatric disorder assessed by Mini International Neuropsychiatric Interview (MINI) (n=1999).

Genotyping, QC, Imputation:

Genomic DNA was isolated from saliva specimens, obtained through the Oragene DNA Sample Collection kit (Genotek), following the manufacturer's protocol. In instances where saliva-derived DNA was unavailable, genomic DNA extracted from whole blood, using the Gentra Puregene kit (Qiagen), was employed for subsequent genotyping analyses. Genotyping was performed using the Global Screening Array at the Broad Institute. Standard genotyping QC procedures were implemented using PLINK2 software. Single-nucleotide variants (SNV) with a minor allele frequency <0.05%, locus missingness >10%, or Hardy-Weinberg equilibrium p <0.000001 were excluded, as well as samples with genotype missingness >10%.

For imputation, we employed the Michigan server using the 1000 Genomes (phase 3) as the reference panel. Quality control measures included the use of an infoscore threshold set at > 0.7.

Ethics statement:

The study was approved by the ethics committee of the Hospital de Clínicas de Porto Alegre, University of São Paulo and Universidade Federal de São Paulo.

Funding:

Study	Lead investigator	Award number	Funder	Country
National Institute of Developmental Psychiatry for Children and Adolescents	Eurípedes Constantino Miguel Filho	Grant numbers 573974/2008-0 and 465550/2014-2	CNPq	Brazil
National Institute of Developmental Psychiatry for Children and Adolescents	Eurípedes Constantino Miguel Filho	Grant numbers 2008/57896-8, 2014/50917-0 and 2021/12901-9)	Fundação de Amparo à Pesquisa do Estado de São Paulo	Brazil
Coorte de Alto Risco para Transtornos Psiquiátricos: Seguimento de 10 anos	Rodrigo Affonseca Bressan	Grant number (2021/05332-8)	Fundação de Amparo à Pesquisa do Estado de São Paulo	Brazil

Electronic Medical Records and Genomics Network (eMERGE) | - | PMID 25469819 | analysis code: eMERGE

Cohort Description:
Data of the electronic MEdical Records and GEnomics (eMERGE) Network was accessed via dbGaP under project ID 18933. A subset of ~17,000 individuals from eMERGE with phenotype and genotype data available were analysed for the current study (dbGaP study accession: phs000888.v1.p1).

The eMERGE Network is a National Human Genome Research Institute (NHGRI)-funded consortium tasked with developing methods and best practices for utilization of the electronic medical record (EMR) as a tool for genomic research. The network is comprised of 10 sites in the U.S., and each site maintains its own biorepository where DNA specimens are linked to phenotypic data contained within EMRs. More information about eMERGE is available on the network's website: https://emerge-network.org/.

Case Ascertainment:
Cases were those with a lifetime medical diagnosis of depression from EMR. An individual was defined as a case of depression if they received at least four diagnoses of depression on different days, from either outpatient or inpatient departments.

Control Ascertainment:
Controls were defined as individuals without a lifetime medical diagnosis of depression, as recorded in the electronic medical records (EMR), and without any of the following major conditions: diabetes mellitus, cerebrovascular disease, coronary stenosis, kidney disease, pulmonary disease, heart failure, COPD, leukemia, lymphoma, Hodgkin's disease, myeloma, aplastic anemia, liver failure, HIV, hepatitis B, hepatitis C, thrombocytopenia, alcoholism, Parkinson's disease, anorexia, tuberculosis (TB), dementia, hyperthyroidism, chronic diarrhea, malabsorption including celiac disease, inflammatory bowel disease, chronic liver disease with cirrhosis, end-stage kidney disease, systemic lupus erythematosus (SLE), pancreatitis, anemia, diseases of white blood cells, and rheumatoid arthritis.

Genotyping, QC, Imputation:
For this study, although the eMERGE consortium had previously imputed our data using the 1000 Genomes reference panel, we conducted additional imputation using the TopMed reference panel on the TopMed Imputation Server, which has demonstrated improved performance for subjects of diverse ancestry (PMID 33568819; 27571263; 25338720).

Genotyped SNPs were excluded if the call rate differed >0.02 between cases and controls. Imputed SNPs were excluded if the estimated imputation accuracy was low (INFO score < 0.7). Rare variants were excluded (MAF < 0.01 or N_{eff}>50 with N_{eff}=2*MAF*(1 - MAF)*N*INFO). SNPs and samples with a call rate < 0.95 were excluded. HWE was not checked for this diverse ancestry sample. Participants with self-reported ethnicity of non-European origin were included in a joint mixed model, as implemented by the GENESIS R package.

Ethics statement:
Informed consent was obtained from all participants from the different participating sites in the network. More information about ethical considerations and consent forms used in eMERGE can be found on the network's website: https://emerge-network.org/projects-2/ethical-considerations-privacy/.

Funding:
eMERGE Network (Phase I)
The eMERGE Network was initiated and funded by National Human Genome Research Institute (NHGRI), in conjunction with additional funding from NIGMS through the following grants: U01-HG-004610 (Group Health Cooperative/University of Washington); U01-HG-

004608 (Marshfield Clinic Research Foundation and Vanderbilt University Medical Center); U01-HG-04599 (Mayo Clinic); U01HG004609 (Northwestern University); U01-HG-04603 (Vanderbilt University Medical Center, also serving as the Administrative Coordinating Center); U01HG004438 (CIDR) and U01HG004424 (the Broad Institute) serving as Genotyping Centers.

eMERGE Network (Phase II – Year 1)
The eMERGE Network was initiated and funded by NHGRI through the following grants: U01HG006389 (Essentia Institute of Rural Health, Marshfield Clinic Research Foundation and Pennsylvania State University); U01HG006382 (Geisinger Clinic); U01HG006375 (Group Health Cooperative/University of Washington); U01HG006379 (Mayo Clinic); U01HG006380 (Icahn School of Medicine at Mount Sinai); U01HG006388 (Northwestern University); U01HG006378 (Vanderbilt University Medical Center); and U01HG006385 (Vanderbilt University Medical Center serving as the Coordinating Center); U01HG004438 (CIDR) and U01HG004424 (the Broad Institute) serving as Genotyping Centers.

eMERGE Network (Phase II – CERC Survey Project)
The CERC Survey project within the eMERGE Network was initiated and funded by NHGRI with additional funding by the NIH Office of the Director through the following grants: U01HG006828 (Cincinnati Children's Hospital Medical Center/Boston Children's Hospital); U01HG006830 (Children's Hospital of Philadelphia); UOIHG006389 (Essentia Institute of Rural Health, Marshfield Clinic Research Foundation and Pennsylvania State University); U01HG006382 (Geisinger Clinic); U01HG006375 (Group Health Cooperative/University of Washington); 3U01HG006379 (Mayo Clinic); U01HG006380 (Icahn School of Medicine at Mount Sinai); 3U01-HG006388 (Northwestern University); U01HG006378 (Vanderbilt University Medical Center); and 3U01HG0006385 (Vanderbilt University Medical Center serving as the Coordinating Center).

eMERGE Network (Phase II – addition of pediatric sites)
The eMERGE Network was initiated and funded by NHGRI through the following grants: U01HG006828 (Cincinnati Children's Hospital Medical Center/Boston Children's Hospital); U01HG006830 (Children's Hospital of Philadelphia); U01HG006389 (Essentia Institute of Rural Health, Marshfield Clinic Research Foundation and Pennsylvania State University); U01HG006382 (Geisinger Clinic); U01HG006375 (Group Health Cooperative/University of Washington); U01HG006379 (Mayo Clinic); U01HG006380 (Icahn School of Medicine at Mount Sinai); U01HG006388 (Northwestern University); U01HG006378 (Vanderbilt University Medical Center); U01HG006385 (Vanderbilt University Medical Center serving as the Coordinating Center); U01HG004438 (CIDR); and U01HG004424 (the Broad Institute) serving as Genotyping Centers.

eMERGE Network (Phase III)
This phase of the eMERGE Network was initiated and funded by the NHGRI through the following grants: U01HG008657 (Group Health Cooperative/University of Washington); U01HG008685 (Brigham and Women's Hospital); U01HG008672 (Vanderbilt University Medical Center); U01HG008666 (Cincinnati Children's Hospital Medical Center); U01HG006379 (Mayo Clinic); U01HG008679 (Geisinger Clinic); U01HG008680 (Columbia University Health Sciences); U01HG008684 (Children's Hospital of Philadelphia); U01HG008673 (Northwestern University); U01HG008701 (Vanderbilt University Medical Center serving as the Coordinating Center); U01HG008676 (Partners Healthcare/Broad Institute); U01HG008664 (Baylor College of Medicine); and U54MD007593 (Meharry Medical College).

eMERGE Network (Phase IV)
This phase of the eMERGE Network was initiated and funded by the NHGRI through the following grants: U01HG011172 (Cincinnati Children's Hospital Medical Center);

U01HG011175 (Children's Hospital of Philadelphia); U01HG008680 (Columbia University); U01HG011176 (Icahn School of Medicine at Mount Sinai); U01HG008685 (Mass General Brigham); U01HG006379 (Mayo Clinic); U01HG011169 (Northwestern University); U01HG011167 (University of Alabama at Birmingham); U01HG008657 (University of Washington); U01HG011181 (Vanderbilt University Medical Center); U01HG011166 (Vanderbilt University Medical Center serving as the Coordinating Center)

Biobank Japan (BBJ) | Okada Y | PMID 28189464; 34594039 | analysis code: BBJ

Cohort Description:
BioBank Japan (BBJ) is a prospective biobank that collected DNA and serum samples from 12 medical institutions in Japan between 2003 and 2008 and recruited approximately 200,000 participants (PMID: 28189464).

Participants were recruited on the basis of having a disease diagnosis of at least 1 out of 47 target diseases. The mean age of participants at recruitment was 63.0 years of age, and 46.3% of the participants were female. Participants were mainly of Japanese ancestry. Data on medical history, drug prescription reports and biomarkers have also been collected.

Case Ascertainment:
A total of 3,893 MD cases were included in GWAS analysis, who had past medical history of depression based on text-mining of electronic medical records or had been prescribed antidepressants (ATC code: N06A) in BBJ.

Control Ascertainment:
A total of 174,747 participants were included as controls, who had no past medical history of schizophrenia.

Genotyping, QC, Imputation:
Genotyping of participants was performed using Illumina HumanOmniExpressExome BeadChip or a combination of the Illumina HumanOmniExpress and HumanExome BeadChip. Variants with the following criteria were excluded: call rate <99%, *P* value for Hardy–Weinberg equilibrium $<1.0 \times 10^{-6}$, and number of heterozygotes <5. In the GWAS, Eagle was used for haplotype phasing without an external reference panel and Minimac3 was used for imputation with the reference panels of the 1000 Genomes Project Phase 3 version 5 genotype data (n = 2,504) and Japanese whole-genome sequencing data (n = 1,037). Variants with an imputation quality of Rsq < 0.7 were excluded, resulting in 13,530,797 variants analysed in total. GWAS was performed using a generalized linear mixed model implemented in SAIGE (v.0.37), adjusting for age, age^2, sex, age × sex, age^2 × sex and the top 20 PCs.

Ethics statement:
All study participants provided written informed consent by ethics committees of the Institute of Medical Sciences, the University of Tokyo and RIKEN Center for Integrative Medical Sciences.

Funding:
The BioBank Japan Project was supported by the Tailor-Made Medical Treatment program (the BioBank Japan Project) of the Ministry of Education, Culture, Sports, Science, and Technology (MEXT), the Japan Agency for Medical Research and Development (AMED).

China Kadoorie Biobank (CKB) | Walters RG; Chen Z | PMID 16131516; 37601966 | analysis code: CKB

Cohort Description:

The CKB is a large population dataset of more than 510,000 individuals from 10 geographically defined regions of China, with extensive clinical, lifestyle, dietary, medical history and genetic data (PMID 22158673). All the participants were interviewed at the baseline by trained staff, while periodic re-surveys have been conducted in ~25,000 surviving participants. Health outcomes of the participants provided through linkages with established registries and health insurance databases are also available.

A total of 17,723 participants reported that had experienced at least one Composite International Diagnostic Interview (CIDI) -A trigger symptom (i.e., feeling sad/depressed, loss of appetite, loss of interest or feeling worthless) for two or more weeks during the past year and were categorized as having "symptom-based" depression in our analyses. These participants were further assessed for major depression (MD) using the Chinese version of the CIDI-short form by trained clinicians at study clinics.

Case Ascertainment:

Participants were defined as having past year MD if they had felt sad, blue, or depressed for ≥2 weeks during the past 12 months, accompanied by at least 3 of 7 additional symptoms, including weight/appetite change, sleep problems, loss of interest and pleasure, loss of energy or fatigue, concentration problems, feelings of guilt or worthlessness, and thoughts of suicide. In our analysis, 4,500 participants who fulfilled the past year MD CIDI-criteria, had at least one relevant medical record (ICD10 F32, F33, F34.1, F38.1 codes) during the follow-up period or reported at resurvey 2 that have been ever diagnosed by a doctor with depression were classified as having "lifetime diagnosis of MD". Exclusion criteria for both cases and controls were a medical diagnosis for dementia, psychosis, bipolar disorder, mental retardation or pervasive developmental disorders.

Control Ascertainment:

Participants that had never been diagnosed with MDD (either diagnosed by CIDI-A questionnaire, self-reported depression or had a medical diagnosis of depression (F32, F33, F34.1, F38.1)) or with neurasthenia and did not report any MDD symptoms constituted the control group (~70,000) in all our analyses. Exclusion criteria for both cases and controls were a medical diagnosis for dementia, psychosis, bipolar disorder, mental retardation or pervasive developmental disorders.

Genotyping, QC, Imputation:

A total of 102,783 participants have been genotyped using 2 custom-designed Affymetrix Axiom arrays including up to 803,000 variants, optimised for genome-wide coverage in Chinese populations. Stringent quality control (QC) included SNP call rate > 0.98, plate effect $P > 10^{-6}$, batch effect $P > 10^{-6}$, Hardy-Weinberg Equilibrium (HWE) deviations $P > 10^{-6}$ (combined 10df χ^2 test from 10 regions), biallelic, Minor Allele Frequency (MAF) difference from 1000 Genomes East-Asian frequencies < 0.2, sample call rate > 0.95, heterozygosity < mean + 3 standard deviations (SD), no sex chromosomes aneuploidy, genetically-determined sex concordant with database, resulting in genotypes for 532,415 variants present on both array versions. Genotypes were imputed to the 1,000 Genomes Phase 3 reference (EAS MAF > 0) using SHAPEIT version 3 and IMPUTE version 4.

A total of 5,376 symptom-based depression cases (1,305 participants with lifetime diagnosis) and 69,998 controls have been genotyped. A linear mixed model (SAIGE) was implemented for the association with depression, adjusting for age, sex, principal components (PCs) and recruitment region. After filtering variants with effective sample size (N_{eff}) < 50 and poorly imputed variants (INFO<0.7), 10,834,708 variants were included in the downstream analyses.

Ethics statement:

All participants provided written informed consent at each survey visit, allowing access to their medical records. Ethical approval was obtained from the Oxford Tropical Research Ethics Committee, the Ethical Review Committees of the Chinese Center for Disease Control and Prevention, Chinese Academy of Medical Sciences, and the Institutional Review Board (IRB) at Peking University. The Chinese Ministry of Health approved the study at the start in 2004, and also approved electronic linkage to health insurance records in 2011. Raw genotyping data were exported from China to the Oxford CKB International Coordinating Center under Data Export Approvals 2014-13 and 2015-39 from the Office of Chinese Human Genetic Resource Administration.

Funding:

The CKB baseline survey and the first resurvey were supported by the Kadoorie Charitable Foundation (Hong Kong). Long-term follow-up was supported by the Wellcome Trust (212946/Z/18/Z, 202922/Z/16/Z, 104085/Z/14/Z, 088158/Z/09/Z), the National Key Research and Development Program of China (2016YFC0900500, 2016YFC0900501, 2016YFC0900504, 2016YFC1303904), and the National Natural Science Foundation of China (81941018, 82192900, 91843302, 91846303).

DNA extraction and genotyping was funded by GlaxoSmithKline and the UK Medical Research Council (MC-PC-13049, MC-PC-14135). The project is supported by core funding from the UK Medical Research Council (MC_UU_00017/1, MC_UU_12026/2, MC_U137686851), Cancer Research UK (C16077/A29186; C500/A16896), and the British Heart Foundation (CH/1996001/9454) to the Clinical Trial Service Unit and Epidemiological Studies Unit and to the MRC Population Health Research Unit at Oxford University. Computation used the Oxford Biomedical Research Computing (BMRC) facility, a joint development between the Wellcome Centre for Human Genetics and the Big Data Institute supported by Health Data Research UK and the NIHR Oxford Biomedical Research Centre; the views expressed are those of the authors and not necessarily those of the NHS, the NIHR, or the Department of Health.

China, Oxford and Virginia Commonwealth University Experimental Research on Genetic Epidemiology cohort (CONVERGE) | Peterson R | PMID 26176920 | analysis code: Converge

Cohort Description:
The CONVERGE cohort of 10,640 Han Chinese women has been previously described (PMID 26176920, PMID 28002544). CONVERGE collected cases of recurrent major depression from 58 provincial mental health centers and psychiatric departments of general medical hospitals in 45 cities and 23 provinces of China. Controls were recruited from patients undergoing minor surgical procedures at general hospitals (37%) or from local community centers (63%). Participants were clinically interviewed using a computerized assessment system by trained interviewers. All participants were Han Chinese women with four Han Chinese grandparents.

Case Ascertainment:
Cases of recurrent major depression (≥2 episodes) were established with the Composite International Diagnostic Interview (CIDI), which utilized DSM-IV criteria. Cases with medical history of bipolar disorder, psychosis, mental retardation and/or drug or alcohol abuse before their first depressive episode were excluded from the study. Cases were aged between 30 and 60 years.

Control Ascertainment:
Control participants were screened and did not meet criteria for major depression, schizophrenia, or bipolar illness. Cases were aged between 40 and 60 years.

Genotyping, QC, Imputation:
CONVERGE samples underwent whole-genome sequencing, as previously described (PMID 26176920). In brief, after genotyping calling, two rounds of imputation were performed: first without a reference panel and then using the 1000 Genomes Phase 1 Asian haplotypes. Variants with a) a P-value for violation HWE < 10^{-6}, b) information score < 0.9, and c) MAF in CONVERGE < 0.5% were excluded from the GWAS, resulting in a final set of 5,987,610 SNPs. The GWAS was conducted with a mixed-linear model including a genetic relationship matrix (FastLMM version 2.06.20130802) as random effect and PCs from eigen-decomposition of this matrix as fixed effects. We further filtered the publicly available GWAS summary statistics by removing variants with N_{eff} < 50.

Ethics statement:
The study protocol was approved by the Ethical Review Board of Oxford University and the ethics committees of all participating hospitals in China. All participants provided written informed consent.

Funding:
CONVERGE was funded by the Wellcome Trust (WT090532/Z/09/Z, WT083573/ Z/07/Z, WT089269/Z/09/Z) and by NIH grant MH100549. RE Peterson and TB Bigdeli are supported by NIMH R01MH125938 and RE Peterson by The Brain & Behavior Research Foundation NARSAD grant 28632 P&S Fund.

Acknowledgements:
The CONVERGE consortium gratefully acknowledges the support of all partners in hospitals across China. Special thanks to all the CONVERGE collaborators and patients who made this work possible. CONVERGE Consortium: Na Cai, Tim B. Bigdeli, Warren Kretzschmar, Yihan Li, Jieqin Liang, Li Song, Jingchu Hu, Qibin Li, Wei Jin, Zhenfei Hu, Guangbiao Wang, Linmao Wang, Puyi Qian, Yuan Liu, Tao Jiang, Yao Lu, Xiuqing Zhang, Ye Yin, Yingrui Li, Xun Xu, Jingfang Gao, Mark Reimers, Todd Webb, Brien Riley, Silviu Bacanu, Roseann E. Peterson, Yiping Chen, Hui Zhong, Zhengrong Liu, Gang Wang, Jing Sun, Hong Sang, Guoqing Jiang, Xiaoyan Zhou, Yi Li, Yi Li, Wei Zhang, Xueyi Wang, Xiang Fang, Runde Pan, Guodong Miao,

Qiwen Zhang, Jian Hu, Fengyu Yu, Bo Du, Wenhua Sang, Keqing Li, Guibing Chen, Min Cai, Lijun Yang, Donglin Yang, Baowei Ha, Xiaohong Hong, Hong Deng, Gongying Li, Kan Li, Yan Song, Shugui Gao, Jinbei Zhang, Zhaoyu Gan, Huaqing Meng, Jiyang Pan, Chengge Gao, Kerang Zhang, Ning Sun, Youhui Li, Qihui Niu, Yutang Zhang, Tieqiao Liu, Chunmei Hu, Zhen Zhang, Luxian Lv, Jicheng Dong, Xiaoping Wang, Ming Tao, Xumei Wang, Jing Xia, Han Rong, Qiang He, Tiebang Liu, Guoping Huang, Qiyi Mei, Zhenming Shen, Ying Liu, Jianhua Shen, Tian Tian, Xiaojuan Liu, Wenyuan Wu, Danhua Gu, Guangyi Fu, Jianguo Shi, Yunchun Chen, Xiangchao Gan, Lanfen Liu, Lina Wang, Fuzhong Yang, Enzhao Cong, Jonathan Marchini, Huanming Yang, Jian Wang, Shenxun Shi, Richard Mott, Qi Xu, Jun Wang, Kenneth S. Kendler, and Jonathan Flint.

Taiwan Depression GREAT study | Kuo PH | PMID 36776991; 35321763 | analysis code: Taiwan1; Taiwan2

Cohort Description:

MDD patients were included from a family study of mood disorders in Taiwan. Patients aged between 18 to 70 years, who met diagnostic criteria of MDD using the Diagnostic and Statistical Manual of Mental Disorders, fourth edition (DSM-IV) were consecutively referred by psychiatrists in clinical settings. The community-recruited Taiwan Biobank Dataset was used as the control group. The control group was further filtered by excluding subjects who have self-reported bipolar disorder (BPD), postpartum depression, alcoholism or drug addiction, schizophrenia, Parkinson's disease or dementia. Both MDD and control subjects were Han Chinese.

Case Ascertainment:

Patients diagnosed with MDD met the criteria outlined in the DSM-IV, as confirmed by a psychiatrist. We excluded individuals previously diagnosed with schizophrenia, schizoaffective disorder, or substance-induced mood disorders. All participants underwent interviews using the Chinese version of the Schedule for Affective Disorders and Schizophrenia (SADS) to gather comprehensive details on psychiatric diagnostic criteria and symptomatology.

Control Ascertainment:

The community-recruited Taiwan Biobank Dataset was used as the control group. Moreover, subjects in Taiwan Biobank Data who had self-reported diagnosis of MDD were classified into case (MDD) group. Sample collection procedures and detailed information about Taiwan Biobank were described elsewhere (PMID 27798100).

Genotyping, QC, Imputation:

Genotyping for Taiwan MDD cases was obtained using Affymetrix CHB Array with 642,832 genetic variants, Affymetrix TWB1.0 Array with 642,545 variants, Illumina Human Omni Express Exiome Beadchips with 949,974 variants and Affymetrix TWB2.0 array with 689,688 variants. Genotyping for Taiwan Biobank controls was obtained using Affymetrix TWB1.0 array with 646,735 variants and Affymetrix TWB2.0 array with 686,439 variants. Because Affymetrix TWB2.0 is a very unique array specifically designed for Taiwanese individuals and is very different from other platforms, imputation was performed separately.

Imputation was done for Affymetrix TWB2.0 array only, and the other set of the imputation was done with combining all other arrays, including Affymetrix CHB, TWB1.0, and Illumina arrays using common SNPs across platforms. The overlapping variants of Affymetrix TWB2.0 array and all other platforms were around 28,000. Imputation was conducted by Michigan Imputation Server using 1000G phase 3 v5 as a reference panel, Eagle v2.3 for phasing, and EAS population for QC. Samples that did not meet the 95% threshold of call rate, kinship-pairs and outliers in population stratification were removed. Genetic variants with call rate <95%, MAF <0.01, p-value of HWE $<10^{-6}$ were also excluded.

The GWAS was performed using PLINK 1.9 and adjusted for 5 ancestry principal components. The GWA analysis was conducted separately by platforms with (1) Affymetrix TWB2.0 and (2) all other platforms combined together. In the latter, variants significantly associated (P<0.005) with genotyping platforms were excluded from downstream analysis. We also used a stricter imputation threshold for filtering (INFO<0.9 instead of 0.7).

Ethics statement:

The authors assert that all procedures contributing to this work comply with the ethical standards of the relevant national and institutional committees on human experimentation and with the Helsinki Declaration of 1975, as revised in 2008.

Funding:

Taiwan MDD GREAT study was supported by projects from the National Health Research Institutes (NHRI-EX107-10627NI), the Ministry of Science and Technology (MOST 105-2628-B-002-028-MY3, 108-2314-B-002-136-MY3), and the National Taiwan University Career Development Project (104R7883, 108L7860) to P-H.K.

Hispanic Community Health Study / Study of Latinos (HCHS/SOL) | Wassertheil-Smoller S; Dunn E | PMID 20609344; 20609343 | analysis code: HCHS

Cohort Description:
HCHS/SOL is a prospective, multicenter, population-based cohort study of Hispanic/Latinx adults in the United States. It recruited 16,000 Hispanic/Latinox participants from Bronx, Chicago, Miami and San Diego under a two-stage area probability sampling (PMID 20609344). All participants went through thorough baseline examination, which lasts for 7 hours on average. Signed informed consent was obtained when participants arrived at assessment centres, followed by fasting state measurements (e.g. anthropometry, phlebotomy, 2-hour glucose load) and several other measurements (e.g., EGC, seated blood pressure). Participants were also administered with a questionnaire collecting their socio-demographic status, medical history, substance use, wellbeing, etc. (PMID 20609343). The HCHS/SOL study was approved by institutional review boards at participating centres, and written informed consent was obtained from all participants.

Case Ascertainment:
Depressive symptoms were assessed during the baseline assessment with the Andresen version of the 10-item Center for Epidemiology Studies of Depression Scale (CES-D-10), which reflected core symptoms of depression in the past week (PMID 29505938). The CES-D-10 scores were curated into a binary phenotype, where participants with scores of no less than 10 were defined as cases (N = 3,979).

Control Ascertainment:
Participants with scores of 6 or below were defined as controls (N = 6,499).

Genotyping, QC, Imputation:
DNA extracted from blood was genotyped on an Illumina custom array, SOL HCHS Custom 15041502 B3, consisting of the Illumina Omni 2.5M array (HumanOmni2.5-8v1-1) and ~150,000 custom SNPs selected to include ancestry-informative markers, variants characteristic of Amerindian populations, previously identified GWAS hits, and other candidate-gene polymorphisms. Genotype imputation was performed with the 1000 Genomes Project phase 1 reference panel implemented by SNAPEIT2 and IMPUTE2 (PMID 26748518).

Mixed-effect logistic regression models adjusting for log of sampling weight, recruiting centre age, sex, highest education attained, genetic subgroup, and the first 5 PCs were conducted by GENESIS (PMID 29505938; 31329242).

Ethics statement:
IRBs approved at each study site.

Funding:
The Hispanic Community Health Study/Study of Latinos was carried out as a collaborative study supported by contracts from the National Heart, Lung, and Blood Institute (NHLBI) to the University of North Carolina (N01-HC65233), University of Miami (N01-HC65234), Albert Einstein College of Medicine (N01-HC65235), Northwestern University (N01-HC65236), and San Diego State University (N01-HC65237). The following Institutes/Centers/Offices contribute to the HCHS/SOL through a transfer of funds to the NHLBI: National Center on Minority Health and Health Disparities, the National Institute of
Deafness and Other Communications Disorders, the National Institute of Dental and Craniofacial Research, the National Institute of Diabetes and Digestive and Kidney Diseases, the National Institute of Neurological Disorders and Stroke, and the Office of Dietary Supplements.

Mexican Adolescent Mental Health Survey (MAMHS) | Cruz-Fuentes C; Martínez-Levy G; Rentería M | PMID 1983355 | analysis code: MAMHS

Cohort Description:
MAMHS is a multistage probability survey designed to be representative of the population of Mexico City and surrounding municipalities. A detailed description of the study can be found elsewhere (PMID 19837355). Briefly, three thousand and five adolescents aged 12–17 were first evaluated in 2005 with the computer-assisted version of the adolescent World Health Organization Composite International Diagnostic Interview (CIDI). Face-to-face interviews were conducted at homes of the participants by trained personnel.

Case Ascertainment:
Lifetime and past year MDD and MDE measures were obtained via participant reports on CIDI and based on DSM-IV criteria. A hierarchical diagnostic algorithm classified participants as MDE cases if they met criteria for a depressive episode, regardless of comorbid disorders, but classified participants as MDD cases only if their depressive symptoms were better explained by MDD than by a different diagnosis, such as bipolar disorder.

Control Ascertainment:
As a result of the study design, controls derived from the same communities and catchment areas as cases. Following CIDI data analysis, controls were defined as those that did not met MDD/MDE DSM-IV criteria.

Genotyping, QC, Imputation:
DNA was extracted from exfoliated oral cavity cells and afterwards genotyped with the Illumina Global Screening Array (GSA) at Broad Institute
Imputation was carried out using the Michigan Imputation server using Minimac4 and the 1000 g-phase-3-v5 (hg19) full reference panels (PMID 34173322). Variants with imputation INFO of less than 0.7, minor allele frequency of no larger than 0.01, missingness no less than 0.05 or with Hardy-Weinberg P value of no larger than 1×10-6 were excluded. Participants with genotype missing rate of no less than 0.05 or heterozygosity outliers (> 3 standard deviations away from the mean) were excluded, leaving 105 cases and 996 controls of admixed Hispanic/Latinx ancestry for GWA analysis. Mixed-effect model logistic regressions were implemented by SAIGE (PMID 30104761) adjusting for age, sex, and the first 20 PCs.

Ethics statement:
Signed informed consent from parents or legal guardians, together with the assent of the participating adolescent were obtained (PMID 19837355). The Institutional Review Board of the National Institute of Psychiatry Ramón de la Fuente Muñiz granted approval Ref:CEI/C/039/2017.

Funding:
Funding for the Mexican Adolescent Mental Health Survey and DNA collection samples (*Genetic Epidemiological Study of mental disorders in adolescents*) was supported by the grants CONACYT-SEP-SSEDF-2003-CO1-22 and CONACYT-SEP-2004-CO1-46594, granted to C. Benjet and C. Cruz-Fuentes respectively.

Genomewide typing was feasible thanks to resources offered by The Million Veteran Program (MVP) through a collaboration agreement between INPRFM and the Psychiatric Genomic Consortium for PTSD : Cohort Name PGC_NIMH_Cruz_PTSD_GSA.

Pregnancy Outcomes, Maternal and Infant Study (PrOMIS) | - | PMID 25620302; 31926482 | analysis code: PrOMIS

Cohort Description:

The PrOMIS cohort is a prospective cohort aimed at understanding the life course and intergenerational effects of interpersonal violence and other forms of trauma among Peruvian women. Between 2012 and 2015, participants were recruited from prenatal care clinics at the Instituto Nacional Materno Perinatal (INMP) in Lima, Peru. Women eligible for inclusion were those who initiated prenatal care prior to 16 weeks gestation. Women were ineligible if they were younger than 18 years of age, did not speak and read Spanish, or had completed more than 16 weeks gestation. A structured questionnaire including maternal socio-demographic, lifestyle characteristics, medical and reproductive histories, and mental health symptoms was completed by an interview with trained research staff.

Case Ascertainment:
Depression was assessed with the Patient Health Questionnaire (PHQ-9) in a clinical interview, which enquired about depressive symptoms for the 2-week period prior to the interview. The symptom measure PHQ-9 score (0–27) was used as the phenotype.

Control Ascertainment:
Not applicable.

Genotyping, QC, Imputation:
Genotyping was conducted on the Illumina Multi-Ethnic Global Chip. Imputation was conducted with the 1000 Genomes phase 3 data (PMID 26432245). GWAS quality control and imputation was performed to the published PGC procedures (PMID 31594949). PC-related and PC-Air was employed in identifying and excluding related individuals, and calculating principal components (PMID 26748516). There were 1,076 MD cases and 2,328 controls for the MD GWAS. GWA logistic regressions were conducted by PLINK, adjusting for the first 10 PCs.

Ethics statement:
The institutional review boards of the INMP and the Office of the Human Research Administration, Havard T.H. Chan School of Public Health approved all procedures used in the study (PMID 31926482; 25620302).

Funding:
Not available.

Genes and Health (GH) | van Heel DA | PMID 31504546 | analysis code: GH

Cohort Description:
Genes & Health (GH) is a community based, long-term study of health and disease in British Bangladeshi and British Pakistani people in east London (PMID 31504546). GH uses a population-based study design, as well as incorporated genomics analysis, electronic health record (EHR) data, and targeted recall-by-genotype (RbG) studies (PMID 31504546). As of February 2020 GH had 38,899 volunteers, and by 2023 it aims to reach 100,000 volunteers participating in the study.

Bangladeshi and British Pakistani individuals (16 and over) living in or working in east London are invited to take part. Participants are recruited at mosques, libraries, GP surgeries and outpatient clinics by bilingual health researchers. Stage 1 participants all give consent to lifelong EHR linkage and donate saliva samples for genetic tests5. Data in volunteer questionnaires and EHR data was checked for data concordance and data with >99% concordance for gender and year of birth was retained. Data that could not be resolved with manual checking or data with clear data entry errors were removed (PMID 31504546).

Case Ascertainment:
Cases and controls were defined on the participants' electronic medical records. Participants were defined as cases for MD if they had an ICD code for major depression and never had any of the following diagnoses: autism, bipolar disorder, dementia, Korsakoff psychosis, a manic episode, personality disorder, psychotic disorder, psychogenic fatigue, seasonal affective disorder or postpartum depression.

Control Ascertainment:
Cases and controls were defined on the participants' electronic medical records. Controls were those participants without an ICD code for major depressive disorder or any of the exclusion diagnoses and did not have a record of antidepressant prescription.

Genotyping, QC, Imputation:
DNA was extracted from the Oragene (DNA Genotek) saliva system and stored from all Stage 1 volunteers. By late 2019, 50,000 samples from stage 1 volunteers were genotyped on the Illumina Infinium Global Screening Array v3.0 (with an additional 46,662 Multi-Disease variants) (PMID 31504546). Genotypes were imputed to the TOPMED-r2 multi-ancestry imputation panel to genome build hg38 using the Michigan imputation server. Following imputation SNP quality control was performed, filtering to common (MAF > 0.01) biallelic autosomal variants with <10% missingness, imputation quality (INFO score) > 0.7 with no significant (P < 1x10-15) deviation from Hardy-Weinberg equilibrium. Individuals with >10% missing genotypes, PCA outliers (see supplement), with missing covariate (age and gender) information, and those not included in electronic healthcare record linkage were removed..
GWAS are run using REGENIE, accounting for case-control imbalance and substantial within cohort relatedness. For step 1 of REGENIE, genotyped or perfectly imputed (INFO = 1) markers are used, and adjusted for age, sex, and the first 20 genetic principal components. Association testing (step 2) is performed using default parameters, adjusting for the same covariates as step 1. (taken from https://www.researchsquare.com/article/rs-3438851/v1)

Ethics statement:
A favourable ethical opinion for the main Genes & Health research study was granted by NRES Committee London - South East (reference 14/LO/1240) on 16 Sept 2014.

Funding:
Genes & Health is/has recently been core-funded by Wellcome (WT102627, WT210561), the Medical Research Council (UK) (M009017, MR/X009777/1, MR/X009920/1), Higher Education Funding Council for England Catalyst, Barts Charity (845/1796), Health Data Research UK (for London substantive site), and research delivery support from the NHS

National Institute for Health Research Clinical Research Network (North Thames). Genes & Health is/has recently been funded by Alnylam Pharmaceuticals, Genomics PLC; and a Life Sciences Industry Consortium of AstraZeneca PLC, Bristol-Myers Squibb Company, GlaxoSmithKline Research and Development Limited, Maze Therapeutics Inc, Merck Sharp & Dohme LLC, Novo Nordisk A/S, Pfizer Inc, Takeda Development Centre Americas Inc.

Acknowledgements:

We thank Social Action for Health, Centre of The Cell, members of our Community Advisory Group, and staff who have recruited and collected data from volunteers. We thank the NIHR National Biosample Centre (UK Biocentre), the Social Genetic & Developmental Psychiatry Centre (King's College London), Wellcome Sanger Institute, and Broad Institute for sample processing, genotyping, sequencing and variant annotation.

This work uses data provided by patients and collected by the NHS as part of their care and support.

We thank: Barts Health NHS Trust, NHS Clinical Commissioning Groups (City and Hackney, Waltham Forest, Tower Hamlets, Newham, Redbridge, Havering, Barking and Dagenham), East London NHS Foundation Trust, Bradford Teaching Hospitals NHS Foundation Trust, Public Health England (especially David Wyllie), Discovery Data Service/Endeavour Health Charitable Trust (especially David Stables), Voror Health Technologies Ltd (especially Sophie Don), NHS England (for what was NHS Digital) - for GDPR-compliant data sharing backed by individual written informed consent.

Most of all we thank all of the volunteers participating in Genes & Health.

QIMR Non-European Cohort | Martin NG; Mitchell BL | 32461290; 34924174; 21042317; 22472876; 26049155 | analysis code: QIMR_DIV

Cohort Description:
The QIMR Non-European cohort included individuals drawn from five studies at QIMR: NC/IRPG (Twins and Spouses), SS1 (SSAGA1), TE (Twin89) and AX (Anxiety and Depression) and the Australian genetics of depression study (AGDS). Controls were drawn from the Qskin Study at QIMR Berghofer Medical Research Institute. Non-European ancestry was defined using genetic principle components and only individuals that fell outside 6 SD of the 1000G PC1/PC2 centroid were included in this cohort.

Case Ascertainment:

Cases were defined as individuals that met criteria for an MDD diagnosis using either the DSM-5 or DSM-IV criteria through structured interviews or questionnaires. The structured interviews were either the shortened Composite International Diagnostic Interview (CIDI-SF) or comprehensive psychiatric interviews designed to assess MDD. Cases excluded individuals that self-reported an additional diagnosis of bipolar disorder or schizophrenia.

Control Ascertainment:

Unrelated controls were selected from the Qskin participants answered the lifestyle questionnaire which included a disease checklist including questions about ever having been diagnosed with psychiatric disorders. Participants of Non-European ancestry who reported not having been given a diagnosis of bipolar disorder, schizophrenia or ADHD were selected as controls.

Genotyping, QC, Imputation:

Array(s): Illumina Infinium Global Screening Array, Illumina 610K platform (I650), Illumina 317K or 370K platforms (I317)
Genotyping was conducted using various array platforms across the included studies. Quality control procedures were carried out as described in other QIMR cohorts included in this study (AGDS, qi3c, qi6c). The Michigan imputation server was used to impute the genotypes using the HRCr1.1 as a reference panel. Imputed genotype dosages were used for the analyses.

GWAS was carried out in SAIGE (v0.44) using a generalized linear mixed model to account for population stratification, cryptic relatedness and unobserved genetic confounding. The GWAS was further adjusted for genotyping batch, cohort and the first 10 principal components. Variants with MAF<1% and imputation accuracy score <0.7 were excluded from the results.

Ethics statement:

All participants in the QIMR and Qskin cohorts provided informed consent that they had read and understood the study information sheets and to confirm that they would be willing to provide a saliva sample for genotyping and downstream generic analyses. Patient consent allows de-identified samples to be sent abroad and used by academic and commercial collaborators and phenotype and genotype data can be used in meta-analyses. All study protocols were approved by the QIMR Berghofer Medical Research Institute Human Research Ethics Committee - approval numbers P2118, P1309, P2148, P2162 (NC/IRPG), 34/92 (SSI), P329 (TE), P642 (AX) and P2304.

Members of contributing groups

HUNT All-In Psychiatry

Amy E Martinsen[1,2,3], Anne Heidi Skogholt[3], Bendik S Winsvold[1,3,4], Børge Sivertsen[5,6,7], Cristen J Willer[8], Eystein Stordal[6,9], Grete Dyb[10], Gunnar Morken[6,11], Håvard Kallestad[6,11], Ingrid Heuch[1], John-Anker Zwart[1,2,3], Jonas B Nielsen[3,8], Katrine K Fjukstad[12,13], Kristian Hveem[3,14,15], Lars G Fritsche[16], Laurent F Thomas[3,17,18,19], Linda M Pedersen[1], Maiken E Gabrielsen[3], Marit S Indredavik[20], Marit Skrove[21], Oddgeir L Holmen[14], Ole A Andreassen[22,23], Ole Kristian Drange[6,11,24.25], Ottar Bjerkeset[6,26], Sara G Selvik[9], Sigrid Børte[2,3,27], Synne Ø Stensland[27,10], Torunn S Nøvik[28], Wei Zhou[29,30]

1 Department of Research and Innovation, Division of Clinical Neuroscience, Oslo University Hospital, Oslo, Norway
2 Institute of Clinical Medicine, Faculty of Medicine, University of Oslo, Oslo, Norway
3 K. G. Jebsen Center for Genetic Epidemiology, Department of Public Health and Nursing, Faculty of Medicine and Health Sciences, Norwegian University of Science and Technology (NTNU), Trondheim, Norway
4 Department of Neurology, Oslo University Hospital, Oslo, Norway
5 Department of Health Promotion, Norwegian Institute of Public Health, Bergen, Norway
6 Department of Mental Health, Faculty of Medicine and Health Sciences, Norwegian University of Science and Technology (NTNU), Trondheim, Norway
7 Department of Research and Innovation, Helse-Fonna HF, Haugesund, Norway
8 Department of Internal Medicine, Division of Cardiovascular Medicine, University of Michigan, Ann Arbor, MI, 48109, USA
9 Department of Psychiatry, Hospital Namsos, Nord-Trøndelag Health Trust, Namsos, Norway
10 Norwegian Centre for Violence and Traumatic Stress Studies, Oslo, Norway
11 Division of Mental Health Care, St. Olavs Hospital, Trondheim University Hospital, Trondheim, Norway
12 Department of Psychiatry, Nord-Trøndelag Hospital Trust, Levanger Hospital, Levanger, Norway
13 Department of Laboratory Medicine, Children's and Women's Health, Norwegian University of Science and Technology (NTNU), Trondheim, Norway
14 HUNT Research Center, Department of Public Health and Nursing, Faculty of Medicine and Health Sciences, Norwegian University of Science and Technology (NTNU), Trondheim, Norway
15 Department of Research, Innovation and Education, St. Olavs Hospital, Trondheim University Hospital, Trondheim, Norway
16 Center for Statistical Genetics, Department of Biostatistics, University of Michigan, Ann Arbor, MI, 48109, USA
17 Department of Clinical and Molecular Medicine, Norwegian University of Science and Technology (NTNU), Trondheim, Norway
18 BioCore - Bioinformatics Core Facility, Norwegian University of Science and Technology (NTNU), Trondheim, Norway
19 Clinic of Laboratory Medicine, St. Olavs Hospital, Trondheim University Hospital, Trondheim, Norway
20 Department of Clinical and Molecular Medicine, Faculty of Medicine and Health Sciences, Norwegian University of Science and Technology (NTNU), Trondheim, Norway
21 Regional Centre for Child and Youth Mental Health and Child Welfare, Department of Mental Health, Faculty of Medicine and Health Sciences, Norwegian University of Science and Technology (NTNU), Trondheim, Norway
22 Division of Mental Health and Addiction, Oslo University Hospital, Oslo, Norway
23 NORMENT, University of Oslo, Oslo, Norway
24 Department of Psychiatry, Sørlandet Hospital, Kristiansand, Norway
25 NORMENT Centre, Institute of Clinical Medicine, University of Oslo, Oslo, Norway
26 Faculty of Nursing and Health Sciences, NORD University, Levanger, Norway

27 Research and Communication Unit for Musculoskeletal Health (FORMI), Department of Research and Innovation, Division of Clinical Neuroscience, Oslo University Hospital, Oslo, Norway
28 Department of Child and Adolescent Psychiatry, St. Olavs Hospital, Trondheim University Hospital, Trondheim, Norway
29 Department of Computational Medicine and Bioinformatics, University of Michigan, Ann Arbor, MI, 48109, USA
30 Analytic and Translational Genetics Unit, Massachusetts General Hospital, Boston, MA, USA

Estonian Biobank Research Team

Andres Metspalu[1], Tõnu Esko[1], Reedik Mägi[1], Mari Nelis[1], Georgi Hudjashov[1]

1 Estonian Biobank Research Team, University of Tartu, Institute of Genomics, Tartu Estonia, (EstBBresearch@ut.ee)

Members of the China Kadoorie Biobank collaborative group

International Steering Committee: Junshi Chen, Zhengming Chen (PI), Robert Clarke, Rory Collins, Liming Li (PI), Jun Lv, Richard Peto, Robin Walters.

International Co-ordinating Centre, Oxford: Daniel Avery, Maxim Barnard, Derrick Bennett, Lazaros Belbasis, Ruth Boxall, Ka Hung Chan, Yiping Chen, Zhengming Chen, Charlotte Clarke, Johnathan Clarke; Robert Clarke, Huaidong Du, Geoffery Ma, Ahmed Edris Mohamed, Hannah Fry, Simon Gilbert, Pek Kei Im, Andri Iona, Maria Kakkoura, Christiana Kartsonaki, Hubert Lam, Kuang Lin, James Liu, Mohsen Mazidi, Iona Millwood, Sam Morris, Qunhua Nie, Alfred Pozarickij, Maryam Rahmati, Paul Ryder, Dan Schmidt, Becky Stevens, Iain Turnbull, Robin Walters, Baihan Wang, Lin Wang, Neil Wright, Ling Yang, Xiaoming Yang, Pang Yao.

National Co-ordinating Centre, Beijing: Xiao Han, Can Hou, Qingmei Xia, Chao Liu, Jun Lv, Pei Pei, Dianjianyi Sun, Canqing Yu, Lang Pan.

10 Regional Co-ordinating Centres:
Qingdao CDC: Zengchang Pang, Ruqin Gao, Shanpeng Li, Haiping Duan, Shaojie Wang, Yongmei Liu, Ranran Du, Yajing Zang, Liang Cheng, Xiaocao Tian, Hua Zhang, Yaoming Zhai, Feng Ning, Xiaohui Sun, Feifei Li. **Licang CDC:** Silu Lv, Junzheng Wang, Wei Hou. **Heilongjiang Provincial CDC:** Wei Sun, Shichun Yan, Xiaoming Cui. **Nangang CDC:** Chi Wang, Zhenyuan Wu,Yanjie Li, Quan Kang. **Hainan Provincial CDC:** Huiming Luo, Tingting Ou. **Meilan CDC:** Xiangyang Zheng, Zhendong Guo, Shukuan Wu, Yilei Li, Huimei Li. **Jiangsu Provincial CDC:** Ming Wu, Yonglin Zhou, Jinyi Zhou, Ran Tao, Jie Yang, Jian Su. **Suzhou CDC:** Fang Liu, Jun Zhang, Yihe Hu, Yan Lu, Liangcai Ma, Aiyu Tang, Shuo Zhang, Jianrong Jin, Jingchao Liu. **Guangxi Provincial CDC:** Mei Lin, Zhenzhen Lu. **Liuzhou CDC:** Lifang Zhou, Changping Xie, Jian Lan,Tingping Zhu,Yun Liu, Liuping Wei, Liyuan Zhou, Ningyu Chen, Yulu Qin, Sisi Wang. **Sichuan Provincial CDC:** Xianping Wu, Ningmei Zhang, Xiaofang Chen, Xiaoyu Chang. **Pengzhou CDC:** Mingqiang Yuan, Xia Wu, Xiaofang Chen, Wei Jiang, Jiaqiu Liu, Qiang Sun. **Gansu Provincial CDC:** Faqing Chen, Xiaolan Ren, Caixia Dong. **Maiji CDC:** Hui Zhang, Enke Mao, Xiaoping Wang, Tao Wang, Xi zhang. **Henan Provincial CDC:** Kai Kang, Shixian Feng, Huizi Tian, Lei Fan. **Huixian CDC:** XiaoLin Li, Huarong Sun, Pan He, Xukui Zhang. **Zhejiang Provincial CDC:** Min Yu, Ruying Hu, Hao Wang. **Tongxiang CDC**: Xiaoyi Zhang, Yuan Cao, Kaixu Xie, Lingli Chen, Dun

Shen. **Hunan Provincial CDC:** Xiaojun Li, Donghui Jin, Li Yin, Huilin Liu, Zhongxi Fu. **Liuyang CDC:** Xin Xu, Hao Zhang, Jianwei Chen,Yuan Peng, Libo Zhang, Chan Qu.

VA Million Veteran Program: Core Acknowledgement for Publications

February 2023

MVP Program Office

- Sumitra Muralidhar, Ph.D., Program Director

US Department of Veterans Affairs, 810 Vermont Avenue NW, Washington, DC 20420

- Jennifer Moser, Ph.D., Associate Director, Scientific Programs

US Department of Veterans Affairs, 810 Vermont Avenue NW, Washington, DC 20420

- Jennifer E. Deen, B.S., Associate Director, Cohort & Public Relations

US Department of Veterans Affairs, 810 Vermont Avenue NW, Washington, DC 20420

MVP Executive Committee

- Co-Chair: Philip S. Tsao, Ph.D.

VA Palo Alto Health Care System, 3801 Miranda Avenue, Palo Alto, CA 94304

- Co-Chair: Sumitra Muralidhar, Ph.D.

US Department of Veterans Affairs, 810 Vermont Avenue NW, Washington, DC 20420

- J. Michael Gaziano, M.D., M.P.H.

VA Boston Healthcare System, 150 S. Huntington Avenue, Boston, MA 02130

- Elizabeth Hauser, Ph.D.

Durham VA Medical Center, 508 Fulton Street, Durham, NC 27705

- Amy Kilbourne, Ph.D., M.P.H.

VA HSR&D, 2215 Fuller Road, Ann Arbor, MI 48105

- Shiuh-Wen Luoh, M.D., Ph.D.

VA Portland Health Care System, 3710 SW US Veterans Hospital Rd, Portland, OR 97239

- Michael Matheny, M.D., M.S., M.P.H.

VA Tennessee Valley Healthcare System, 1310 24th Ave. South, Nashville, TN 37212

- Dave Oslin, M.D.

Philadelphia VA Medical Center, 3900 Woodland Avenue, Philadelphia, PA 19104

MVP Co-Principal Investigators

- J. Michael Gaziano, M.D., M.P.H.

VA Boston Healthcare System, 150 S. Huntington Avenue, Boston, MA 02130

- Philip S. Tsao, Ph.D.

VA Palo Alto Health Care System, 3801 Miranda Avenue, Palo Alto, CA 94304

MVP Core Operations

- Lori Churby, B.S., Director, MVP Regulatory Affairs

VA Palo Alto Health Care System, 3801 Miranda Avenue, Palo Alto, CA 94304

- Stacey B. Whitbourne, Ph.D., Director, MVP Cohort Management

VA Boston Healthcare System, 150 S. Huntington Avenue, Boston, MA 02130

- Jessica V. Brewer, M.P.H., Director, MVP Recruitment & Enrollment

VA Boston Healthcare System, 150 S. Huntington Avenue, Boston, MA 02130

- Shahpoor (Alex) Shayan, M.S., Director, MVP Recruitment and Enrollment Informatics

VA Boston Healthcare System, 150 S. Huntington Avenue, Boston, MA 02130

- Luis E. Selva, Ph.D., Executive Director, MVP Biorepositories

VA Boston Healthcare System, 150 S. Huntington Avenue, Boston, MA 02130

- Saiju Pyarajan Ph.D., Director, Data and Computational Sciences

VA Boston Healthcare System, 150 S. Huntington Avenue, Boston, MA 02130

- Kelly Cho, M.P.H, Ph.D., Director, MVP Phenomics Data Core

VA Boston Healthcare System, 150 S. Huntington Avenue, Boston, MA 02130

- Scott L. DuVall, Ph.D., Director, VA Informatics and Computing Infrastructure (VINCI)

VA Salt Lake City Health Care System, 500 Foothill Drive, Salt Lake City, UT 84148

- Mary T. Brophy M.D., M.P.H., Director, VA Central Biorepository

VA Boston Healthcare System, 150 S. Huntington Avenue, Boston, MA 02130

- MVP Coordinating Centers
    - MVP Coordinating Center, Boston - J. Michael Gaziano, M.D., M.P.H.
    VA Boston Healthcare System, 150 S. Huntington Avenue, Boston, MA 02130
    - MVP Coordinating Center, Palo Alto – Philip S. Tsao, Ph.D.
    VA Palo Alto Health Care System, 3801 Miranda Avenue, Palo Alto, CA 94304
    - MVP Information Center, Canandaigua – Brady Stephens, M.S.
    Canandaigua VA Medical Center, 400 Fort Hill Avenue, Canandaigua, NY 14424
    - Cooperative Studies Program Clinical Research Pharmacy Coordinating Center, Albuquerque – Todd Connor, Pharm.D.; Dean P. Argyres, B.S., M.S.
    New Mexico VA Health Care System, 1501 San Pedro Drive SE, Albuquerque, NM 87108

MVP Publications and Presentations Committee

- Co-Chair: Themistocles L. Assimes, M.D., Ph. D

VA Palo Alto Health Care System, 3801 Miranda Avenue, Palo Alto, CA 94304

- Co-Chair: Adriana Hung, M.D.; M.P.H

VA Tennessee Valley Healthcare System, 1310 24th Ave. South, Nashville, TN 37212

- Co-Chair: Henry Kranzler, M.D.

Philadelphia VA Medical Center, 3900 Woodland Avenue, Philadelphia, PA 19104

MVP Local Site Investigators

- Samuel Aguayo, M.D., Phoenix VA Health Care System

650 E. Indian School Road, Phoenix, AZ 85012

- Sunil Ahuja, M.D., South Texas Veterans Health Care System

7400 Merton Minter Boulevard, San Antonio, TX 78229

- Kathrina Alexander, M.D., Veterans Health Care System of the Ozarks

1100 North College Avenue, Fayetteville, AR 72703

- Xiao M. Androulakis, M.D., Columbia VA Health Care System

6439 Garners Ferry Road, Columbia, SC 29209

- Prakash Balasubramanian, M.D., William S. Middleton Memorial Veterans Hospital

2500 Overlook Terrace, Madison, WI 53705

- Zuhair Ballas, M.D., Iowa City VA Health Care System

601 Highway 6 West, Iowa City, IA 52246-2208

- Jean Beckham, Ph.D., Durham VA Medical Center

508 Fulton Street, Durham, NC 27705

- Sujata Bhushan, M.D., VA North Texas Health Care System

4500 S. Lancaster Road, Dallas, TX 75216

- Edward Boyko, M.D., VA Puget Sound Health Care System

1660 S. Columbian Way, Seattle, WA 98108-1597

- David Cohen, M.D., Portland VA Medical Center

3710 SW U.S. Veterans Hospital Road, Portland, OR 97239

- Louis Dellitalia, M.D., Birmingham VA Medical Center

700 S. 19th Street, Birmingham AL 35233

- L. Christine Faulk, M.D., Robert J. Dole VA Medical Center

5500 East Kellogg Drive, Wichita, KS 67218-1607

- Joseph Fayad, M.D., VA Southern Nevada Healthcare System

6900 North Pecos Road, North Las Vegas, NV 89086

- Daryl Fujii, Ph.D., VA Pacific Islands Health Care System

459 Patterson Rd, Honolulu, HI 96819

- Saib Gappy, M.D., John D. Dingell VA Medical Center

4646 John R Street, Detroit, MI 48201

- Frank Gesek, Ph.D., White River Junction VA Medical Center

163 Veterans Drive, White River Junction, VT 05009

- Jennifer Greco, M.D., Sioux Falls VA Health Care System

2501 W 22nd Street, Sioux Falls, SD 57105

- Michael Godschalk, M.D., Richmond VA Medical Center

1201 Broad Rock Blvd., Richmond, VA 23249

- Todd W. Gress, M.D., Ph.D., Hershel “Woody” Williams VA Medical Center

1540 Spring Valley Drive, Huntington, WV 25704

- Samir Gupta, M.D., M.S.C.S., VA San Diego Healthcare System

3350 La Jolla Village Drive, San Diego, CA 92161

- Salvador Gutierrez, M.D., Edward Hines, Jr. VA Medical Center

5000 South 5th Avenue, Hines, IL 60141

- John Harley, M.D., Ph.D., Cincinnati VA Medical Center

3200 Vine Street, Cincinnati, OH 45220

- Kimberly Hammer, Ph.D., Fargo VA Health Care System

2101 N. Elm, Fargo, ND 58102

- Mark Hamner, M.D., Ralph H. Johnson VA Medical Center

109 Bee Street, Mental Health Research, Charleston, SC 29401

- Adriana Hung, M.D., M.P.H., VA Tennessee Valley Healthcare System

1310 24th Avenue, South Nashville, TN 37212

- Robin Hurley, M.D., W.G. (Bill) Hefner VA Medical Center

1601 Brenner Ave, Salisbury, NC 28144

- Pran Iruvanti, D.O., Ph.D., Hampton VA Medical Center

100 Emancipation Drive, Hampton, VA 23667

- Frank Jacono, M.D., VA Northeast Ohio Healthcare System

10701 East Boulevard, Cleveland, OH 44106

- Darshana Jhala, M.D., Philadelphia VA Medical Center

3900 Woodland Avenue, Philadelphia, PA 19104

- Scott Kinlay, M.B.B.S., Ph.D., VA Boston Healthcare System

150 S. Huntington Avenue, Boston, MA 02130

- Jon Klein, M.D., Ph.D., Louisville VA Medical Center

800 Zorn Avenue, Louisville, KY 40206

- Michael Landry, Ph.D., Southeast Louisiana Veterans Health Care System

2400 Canal Street, New Orleans, LA 70119

- Peter Liang, M.D., M.P.H., VA New York Harbor Healthcare System

423 East 23rd Street, New York, NY 10010

- Suthat Liangpunsakul, M.D., M.P.H., Richard Roudebush VA Medical Center

1481 West 10th Street, Indianapolis, IN 46202

- Jack Lichy, M.D., Ph.D., Washington DC VA Medical Center

50 Irving St, Washington, D. C. 20422

- C. Scott Mahan, M.D., Charles George VA Medical Center

1100 Tunnel Road, Asheville, NC 28805

- Ronnie Marrache, M.D., VA Maine Healthcare System

1 VA Center, Augusta, ME 04330

- Stephen Mastorides, M.D., James A. Haley Veterans' Hospital

13000 Bruce B. Downs Blvd, Tampa, FL 33612

- Elisabeth Mates M.D., Ph.D., VA Sierra Nevada Health Care System

975 Kirman Avenue, Reno, NV 89502

- Kristin Mattocks, Ph.D., M.P.H., Central Western Massachusetts Healthcare System

421 North Main Street, Leeds, MA 01053

- Paul Meyer, M.D., Ph.D., Southern Arizona VA Health Care System

3601 S 6th Avenue, Tucson, AZ 85723

- Jonathan Moorman, M.D., Ph.D., James H. Quillen VA Medical Center

Corner of Lamont & Veterans Way, Mountain Home, TN 37684

- Timothy Morgan, M.D., VA Long Beach Healthcare System

5901 East 7th Street Long Beach, CA 90822

- Maureen Murdoch, M.D., M.P.H., Minneapolis VA Health Care System

One Veterans Drive, Minneapolis, MN 55417

- James Norton, Ph.D., VA Health Care Upstate New York

113 Holland Avenue, Albany, NY 12208

- Olaoluwa Okusaga, M.D., Michael E. DeBakey VA Medical Center

2002 Holcombe Blvd, Houston, TX 77030

- Kris Ann Oursler, M.D., Salem VA Medical Center

1970 Roanoke Blvd, Salem, VA 24153

- Ana Palacio, M.D., M.P.H., Miami VA Health Care System

1201 NW 16th Street, 11 GRC, Miami FL 33125

- Samuel Poon, M.D., Manchester VA Medical Center

718 Smyth Road, Manchester, NH 03104

- Emily Potter, Pharm.D., VA Eastern Kansas Health Care System

4101 S 4th Street Trafficway, Leavenworth, KS 66048

- Michael Rauchman, M.D., St. Louis VA Health Care System

915 North Grand Blvd, St. Louis, MO 63106

- Richard Servatius, Ph.D., Syracuse VA Medical Center

800 Irving Avenue, Syracuse, NY 13210

- Satish Sharma, M.D., Providence VA Medical Center

830 Chalkstone Avenue, Providence, RI 02908

- River Smith, Ph.D., Eastern Oklahoma VA Health Care System

1011 Honor Heights Drive, Muskogee, OK 74401

- Peruvemba Sriram, M.D., N. FL/S. GA Veterans Health System

1601 SW Archer Road, Gainesville, FL 32608

- Patrick Strollo, Jr., M.D., VA Pittsburgh Health Care System

University Drive, Pittsburgh, PA 15240

- Neeraj Tandon, M.D., Overton Brooks VA Medical Center

510 East Stoner Ave, Shreveport, LA 71101

- Philip Tsao, Ph.D., VA Palo Alto Health Care System

3801 Miranda Avenue, Palo Alto, CA 94304-1290

- Gerardo Villareal, M.D., New Mexico VA Health Care System

1501 San Pedro Drive, S.E. Albuquerque, NM 87108

- Agnes Wallbom, M.D., M.S., VA Greater Los Angeles Health Care System

11301 Wilshire Blvd, Los Angeles, CA 90073

- Jessica Walsh, M.D., VA Salt Lake City Health Care System

500 Foothill Drive, Salt Lake City, UT 84148

- John Wells, Ph.D., Edith Nourse Rogers Memorial Veterans Hospital

200 Springs Road, Bedford, MA 01730

- Jeffrey Whittle, M.D., M.P.H., Clement J. Zablocki VA Medical Center

5000 West National Avenue, Milwaukee, WI 53295

- Mary Whooley, M.D., San Francisco VA Health Care System

4150 Clement Street, San Francisco, CA 94121

- Allison E. Williams, N.D., Ph.D., R.N, Bay Pines VA Healthcare System

10,000 Bay Pines Blvd Bay Pines, FL 33744

- Peter Wilson, M.D., Atlanta VA Medical Center

1670 Clairmont Road, Decatur, GA 30033

- Junzhe Xu, M.D., VA Western New York Healthcare System

3495 Bailey Avenue, Buffalo, NY 14215-1199

- Shing Shing Yeh, Ph.D., M.D., Northport VA Medical Center

79 Middleville Road, Northport, NY 11768

Conflicts of interest

Ole A. Andreassen is a consultant for Cortechs.ai and Presicion Health, and has received speaker's honorarium from Janssen, Lundbeck, Otsuka and Sunovion.

Dr. Kranzler is a member of advisory boards for Dicerna Pharmaceuticals, Sophrosyne Pharmaceuticals, Enthion Pharmaceuticals, and Clearmind Medicine; a consultant to Sobrera Pharmaceuticals; the recipient of research funding and medication supplies for an investigator-initiated study from Alkermes; a member of the American Society of Clinical Psychopharmacology's Alcohol Clinical Trials Initiative, which was supported in the last three years by Alkermes, Dicerna, Ethypharm, Lundbeck, Mitsubishi, Otsuka, and Pear Therapeutics; and a holder of U.S. patent 10,900,082 titled: "Genotype-guided dosing of opioid agonists," issued 26 January 2021.
